# Evaluation and Identification of the Neuroprotective Compounds of Xiaoxuming Decoction by Machine Learning: A Novel Mode to Explore the Combination Rules in Traditional Chinese Medicine Prescription

**DOI:** 10.1155/2019/6847685

**Published:** 2019-07-10

**Authors:** Shilun Yang, Yanjia Shen, Wendan Lu, Yinglin Yang, Haigang Wang, Li Li, Chunfu Wu, Guanhua Du

**Affiliations:** ^1^School of Life Science and Biopharmaceutics, Shenyang Pharmaceutical University, No. 103, Wen hua Road, Shenyang 110016, China; ^2^Beijing Key Laboratory of Drug Targets Identification and Drug Screening, Institute of Materia Medica, Chinese Academy of Medical Sciences and Peking Union Medical College, No. 2, Nan wei Road, Beijing 100050, China

## Abstract

Xiaoxuming decoction (XXMD), a classic traditional Chinese medicine (TCM) prescription, has been used as a therapeutic in the treatment of stroke in clinical practice for over 1200 years. However, the pharmacological mechanisms of XXMD have not yet been elucidated. The purpose of this study was to develop neuroprotective models for identifying neuroprotective compounds in XXMD against hypoxia-induced and H_2_O_2_-induced brain cell damage. In this study, a phenotype-based classification method was designed by machine learning to identify neuroprotective compounds and to clarify the compatibility of XXMD components. Four different single classifiers (AB, kNN, CT, and RF) and molecular fingerprint descriptors were used to construct stacked naïve Bayesian models. Among them, the RF algorithm had a better performance with an average MCC value of 0.725±0.014 and 0.774±0.042 from 5-fold cross-validation and test set, respectively. The probability values calculated by four models were then integrated into a stacked Bayesian model. In total, two optimal models, s-NB-1-LPFP6 and s-NB-2-LPFP6, were obtained. The two validated optimal models revealed Matthews correlation coefficients (MCC) of 0.968 and 0.993 for 5-fold cross-validation and of 0.874 and 0.959 for the test set, respectively. Furthermore, the two models were used for virtual screening experiments to identify neuroprotective compounds in XXMD. Ten representative compounds with potential therapeutic effects against the two phenotypes were selected for further cell-based assays. Among the selected compounds, two compounds significantly inhibited H_2_O_2_-induced and Na_2_S_2_O_4_-induced neurotoxicity simultaneously. Together, our findings suggested that machine learning algorithms such as combination Bayesian models were feasible to predict neuroprotective compounds and to preliminarily demonstrate the pharmacological mechanisms of TCM.

## 1. Introduction

Traditional Chinese medicine (TCM) prescriptions, as a representative of drug combinations, are generally used in combination with multiple medicinal herbs in a certain dose to exert therapeutic effects [1, 2]. In clinical practice, the TCM prescription Xiaoxuming decoction (XXMD) has been an effective prescription for the treatment of stroke for over 1200 years and has been recorded in Beiji Qianjin Yaofang (Essential Prescriptions Worth a Thousand Gold for Emergencies) in Tang Dynasty [3]. XXMD consists of twelve herbs, which are presented in Table 1. In previous studies, it has been shown that prophylactic administration of XXMD for 5 days prior to surgery protected rats against ischemia-reperfusion-induced brain injury [4]. However, due to the complex composition in TCM prescriptions, it is difficult to conduct in-depth studies on the underlying pharmacological mechanisms of XXMD. Therefore, it is essential to clarify the compatibility of various herbs in TCM prescriptions with sufficient approaches. To further explore the therapeutic roles of XXMD, phenotypic-based drug discovery technology was employed in this study.

Regardless of the molecular mechanism of action during the initial stages of drug discovery, phenotypic-based screening, as opposed to target-based screening, has provided new impetus to improve the probability of success in drug discovery [5, 6]. Thus, identifying the components in XXMD by phenotypic-based screening will contribute to elucidating the mechanism of therapeutic effects of XXMD. There is no doubt that traditional analytic techniques, such as experimental screening methods, take up time and resources. To solve these issues, and to enhance screening efficiency, virtual screening (VS) methods, thereby taking machine learning as an example, have been widely adopted [7–9]. However, studies that focus on classification predictions towards phenotypic screening to evaluate and identify classic TCM prescriptions and discovering neuroprotective agents against ischemic stroke are limited.

Ischemic stroke, which is the result of an insufficient blood supply leading to dysfunction of the brain tissues [10], is mainly due to the occurrence of atherosclerosis and thrombosis in the arteries supplying blood to the brain [11]. Tissue plasminogen activator (tPA) is the only thrombolytic agent that is approved by the US Food and Drug Administration (FDA) [12]. However, when a thrombus is destroyed, blood containing a large amount of reactive oxygen species (ROS) will perfuse into the ischemic site, thereby causing cerebral ischemia-reperfusion (I/R) injury. As the most essential ingredient of ROS, H_2_O_2_ is produced in the body's oxidative metabolism and is considered a messenger of intracellular signaling cascades [13]. Large-scale production of H_2_O_2_ will damage the structure and function of biological membranes and organelles (such as mitochondria) in neuronal cells [14, 15]. Moreover, the excessive level of oxidative stress caused by I/R injury results in a vicious cycle of mitochondrial dysfunction, calcium overload, excitatory glutamate excess release, and lipid peroxidation following cerebral I/R injury [16]. This will lead to an imbalance of neuronal cell homeostasis, which subsequently aggravates I/R injury. Thus, to facilitate cerebral tissue repair, identifying novel therapies is of utmost importance.

**Figure 1:**
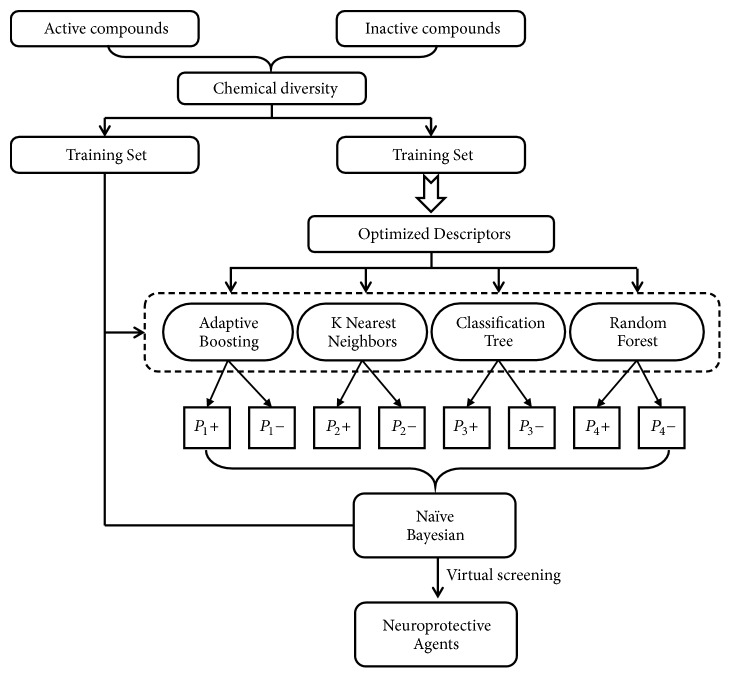
Workflow for classification model building, validation, and virtual screening (VS) as applied to neuroprotective agents.

In this study, a workflow for the classification models, model validations, and their application to virtual screening of neuroprotective agents is presented in Figure 1. Two data sets containing 263 and 116 neuroprotectants were constructed, respectively, and categorized into a training set and test set. The data set containing 263 compounds was used to construct hypoxia-induced neuronal injury models (NIN models), while the data set containing 116 compounds was used to construct H_2_O_2_-induced neuronal injury models (NHN models). Next, data of four single classification models (AB, kNN, CT, and RF) and molecular fingerprint descriptors were integrated to construct stacked naïve Bayesian (s-NB) models. The predictive power of the models was evaluated by five times cross-validation of the training set and validation of the test set. The final two optimal stacked NB classification models, s-NB-1-LPFP4 and s-NB-2-LCFP6, were used for classification of the phenotypic-based active ingredient combinations and screen potential neuroprotectants in XXMD. Furthermore, to verify the results of the two optimal Bayesian classification models, sodium dithionite (Na_2_S_2_O_4_) and hydrogen peroxide (H_2_O_2_) were used to induce chemical hypoxia and oxidative damage, which mimicked the hypoxic phenotype and reperfused phenotype in SH-SY5Y cells [17, 18].

## 2. Materials and Methods

### 2.1. Data Collection and Preparation

#### 2.1.1. Data Collection from XXMD Compounds

Compounds from twelve herbs present in XXMD were collected from the Chinese natural product chemical composition database (National Center for Pharmaceutical Screening, Chinese Academy of Medical Sciences, http://pharmdata.ncmi.cn/cnpc/), TCM-Database@Taiwan Database (http://tcm.cmu.edu.tw), the Traditional Chinese Medicine Systems Pharmacology (TCMSP) Database (http://lsp.nwsuaf.edu.cn/tcmsp.php), and PubChem Compound Database (https://www.ncbi.nlm.nih.gov/pccompound/). A total of 1858 compounds were collected. After removal of duplicate compounds, which were found in more than one herb, 1484 compounds were selected for further studies. Information of XXMD and the twelve herbs obtained from database sources is presented in Table 1.

#### 2.1.2. Collection and Preparation of Training Set and Test Set

**Figure 2:**
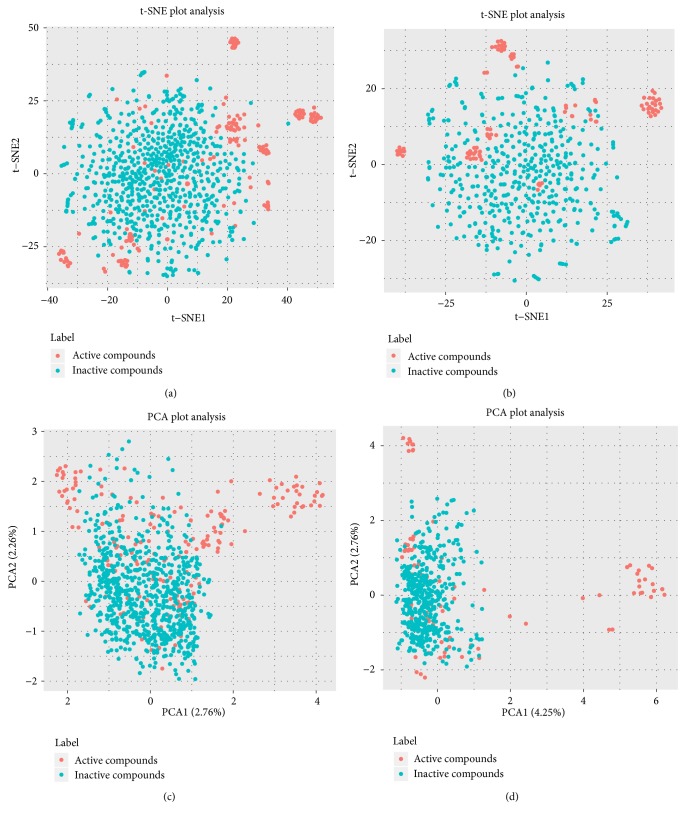
Visual representation of the chemical space of active compounds (red) and inactive compounds (light green) against hypoxia-induced (a and c) and H_2_O_2_-induced (b and d) neurotoxicity. The visualizations of (a) and (b) were generated using t-distributed stochastic neighbor embedding (t-SNE) based on Morgan fingerprints (4096 bits). The visualizations of (c) and (d) were generated using principal component analysis (PCA) based on Morgan fingerprints (4096 bits).

Compounds that were defined as active in the two phenotypes were collected from the ChEMBL database [19, 20], using IC_50_≤10*μ*M in nerve cells as the selection criterion. After eliminating duplicate structures, active datasets were constituted with corresponding neuroprotective compounds. It is noteworthy that compounds collected from the ChEMBL database did not overlap with compounds in the XXMD dataset. Corresponding decoys (defined as inactive) were automatically generated by the DUD-E online database with a ratio of 4:1 to active compounds [21]. Morgan fingerprints (4096 bits, radius = 2) were generated using the RDKit package in Python and were then fed into a t-distributed stochastic neighbor embedding (t-SNE) algorithm and principal component analysis (PCA) to obtain 2-dimensional representations [22–24]. As shown in the t-SNE plot (Figures 2(a) and 2(b)), remarkable colocalization of several active compound clusters was shown, which was well separated from inactive compounds. It should be noted that different active compound clusters may represent different chemotypes, which would require further investigation in the future. Results from PCA plots are presented in Figures 2(c) and 2(d). Considering the sparseness nature of Morgan fingerprints, it is reasonable that the explained variance ratios of PC1 and PC2 are not high. However, when using the top two PCs, some active compounds can still be distinguished from inactive compounds.

Both the active and inactive data sets were randomly distributed into a training set and test set with a ratio of 3:1 as shown in Table 2. In all data sets, neuroprotective compounds and decoys were, respectively, marked as “1” and “0.” Prior to the calculation of molecular descriptors, all compounds required the addition of hydrogen atoms, deprotonation of strong acids, protonation of strong bases, generation of a valid 3D conformation, and energy minimization. Detailed information on the training sets and test sets is presented in Tables S1–S4.

### 2.2. Molecular Descriptors

Molecular descriptors are the basis for the combination of machine learning [25]. Therefore, we used Discovery Studio 2016 (DS 2016) [26] and MOE 2014.9 software [27] to calculate three sets of two-dimensional (2D) descriptors to describe each compound. A total of 256 descriptors calculated by DS 2016 were constituted as the first descriptor set. In addition, the second descriptor set was composed of 185 descriptors calculated by MOE 2014.9. Together, the two molecular descriptor sets constituted the third molecular descriptor set for virtual screening, which contained 441 (256+185) descriptors. Furthermore, in this study, molecular fingerprints involved the SciTegic extended-connectivity fingerprints (FCFP and ECFP) and Daylight-style path-based fingerprints (FPFP and EPFP) were also calculated with DS 2016 [6].

### 2.3. Molecular Descriptors Selection

In this study, Pearson correlation analysis was performed to identify descriptors that were highly correlated with activity [28]. Firstly, descriptors in which values appeared in high frequency of more than 50% were eliminated. Secondly, descriptors in which correlation coefficients had an activity of less than 0.1 were excluded [29]. If the absolute value of the correlation coefficients between two descriptors was higher than 0.9, the descriptor possessing a lower correlation coefficient with activity was deleted. Finally, the remaining descriptors were used for building models. Detailed information on the descriptors is presented in Table S5.

### 2.4. Methods for Model Building

We followed the methods of Fang et al. (2016) [6]. In this study, five different machine learning methods, Adaboost (AB), *k* nearest neighbors (*k*NN), classification tree (CT), random forest (RF), and naïve Bayesian (NB), were used for computational processes. The construction of models was performed using Orange Canvas 3.4.1 [30] (AB, *k*NN, CT, and RF) and DS 2016 (NB). In addition, four models (AB, *k*NN, CT, and RF) exported two probabilities (positive and negative probability) as well as estimated target values (1 or 0).

#### 2.4.1. Single Classifier Model

*(1) Adaboost. *Adaboost (AB) is an iterative algorithm that was designed to get the weighted sum of classifiers by using the lifting method [31, 32]. It tends to tweak the subsequent weak learners in favor of reducing misclassification caused by previous classifiers [33]. Weight coefficients of the N samples in the algorithm were distributed to the same value initialized to 1/N. Next, this subsection was used to train the classifier and calculate the weights of the misclassified samples as the weighted error rate.

*(2) k Nearest Neighbors*. The k nearest neighbors (kNN) algorithm is a nonparametric learning method for classification and is regression-based on the closest training sample in the feature space [34, 35]. The feature selection, the number of nearest neighbors K, and the shape of the distance weighting function determine the performance of the K-NN model. In this method, each molecule is removed from the training set, and the activity value is predicted to have no inverse distance weighted average activity of the most similar molecules. In this study, K was optimized (K=1-10).

*(3) Classification Tree. *Classification tree (CT), a method commonly used in data mining, is designed to illustrate the structure of any particular field [36]. The C4.5 tree in Orange is designed to build classification trees from a set of training data by splitting criterion called normalized information gain [37, 38]. The attribute with the highest normalized information gain is selected to make the decision [6]. The parameters in Orange were adopted using the default settings.

*(4) Random Forest*. Random forest (RF) is an ensemble learning method that stacks multiple decision trees to produce consensus predictions for each tree [39, 40]. RF randomly divides the data in the training set to build individual trees. The arbitrary node of the trees is drawn from the best subset of total descriptors and is selected. Random decision forests correct for the habit of decision trees of overfitting their training set.

#### 2.4.2. Stacked Naïve Bayesian Classification Model

To improve the prediction reliability of a single model, consistent scoring and data fusion are beneficial. In general, stacked models reduce unreliable predicted noise hidden by single classification model [6, 41]. In this study, the probability values calculated by four models were quoted as new descriptors, and the prediction results were integrated with NB models. Stacked-NB classification (s-NB) models were constructed and validated by DS 2016 [29]. The learning process generated a large set of Boolean features from the input descriptors. The weights calculated for each feature using a Laplacian-adjusted probability estimate were summed to provide a probability estimate, which was a relative predictor for the possibility of that sample being from the good subset [42, 43].

### 2.5. Performance Parameters Applied for Model Evaluation

We followed the methods of Fang et al. (2016) [6]. In this study, the quality of the models was assessed by 5-fold cross-validation and test set validation. Measurement parameters included true positives (TP), true negatives (TN), false positives (FP), and false negatives (FN). Subsequently, sensitivity (SE), specificity (SP), positive predictive value (PPV), and Matthews correlation coefficient (MCC) were calculated by (1)–(4). TP indicated number of active compounds predicted to be active; TN indicated the number of inactive compounds predicted to be inactive; FP represented the number of inactive compounds predicted to be active; and FN represented the number of active compounds predicted to be inactive. Similarly, SE represented the accuracy of prediction for active compounds and SP represented the accuracy of prediction for inactive compounds. PPV indicated the overall prediction accuracy for all compounds in the dataset. MCC signified the most important indicator for the quality of binary classification and was calculated to evaluate the predictive power of the model with values ranging from -1 to 1.

(1) SE=TPTP+FN

(2) SP=TNTN+FP

(3) PPV=TPTP+FP

(4) MCC=TP×TN−FN×FPTP+FNTP+FPTN+FNTN+FP

Furthermore, s-NB models measure the accuracy of prediction by calculating the receiver operating characteristic (ROC) and area under the curve (AUC) of the training set and the test set. The ROC curve is a comprehensive indicator that reflects the model sensitivity and specific continuous variables [44]. The ROC curve is based on the true positive rate as the ordinate and the false positive rate as the abscissa.

### 2.6. Cell-Based Neuroprotective Assay

#### 2.6.1. Cell Culture and Treatment

SH-SY5Y cells (human neuroblastoma cell line, Institute of Materia Medica, Chinese Academy of Medical Science, Beijing, China) were cultured in high glucose Dulbecco's Modified Eagle's Medium (DMEM) supplemented with 10% (v/v) fetal bovine serum (FBS, Gibco, USA). Cells were divided into three groups: (1) control group: no treatment, (2) model group: cells were treated with 8 mM Na_2_S_2_O_4_ (Sigma, St. Louis, MO, USA) or 200 *μ*M H_2_O_2_, and (3) treatment group: cells were pretreated with test compounds for 2 h; then 8 mM Na_2_S_2_O_4_ or 200 *μ*M H_2_O_2_ was added, respectively (the oxygen glucose deprivation (OGD) condition was produced by using Na_2_S_2_O_4_, which scavenges O_2_ molecules in solution and reduces the oxygen tension to zero). Test compounds were diluted to four concentrations (0.3*μ*M, 1*μ*M, 3*μ*M, and 10*μ*M).

#### 2.6.2. MTT Assay

We followed the methods of Fang et al. (2016) [6]. The MTT assay was used to assess cell survival. SH-SY5Y cells (5 x 10^3^ cells/well) were seeded in 96-well plates in 100 *μ*L of medium per well and were cultured for 20 hours. When the cell density was roughly 80%, cells were treated with medium containing different concentrations of test compounds for 2 hours; then 200 *μ*M H_2_O_2_ or 8 mM Na_2_S_2_O_4_ was separately added and cells were incubated for 22 hours. MTT reagent (final concentration 1.0 mg/ml) was added 2 hours before the end of the incubation. After 3 hours of incubation, the medium was replaced with 100 *μ*L of dimethyl sulfoxide (DMSO) and the absorption of the solubilized formazan was measured at 570 nm using a microplate reader (Spectra Max M5, Molecular Devices, USA). Cell survival was normalized to the control group, for which the cell survival was set to 100%.

### 2.7. Statistical Methods

Data are presented as the mean ± standard deviation. Statistical analysis was performed using the SPSS statistical package (Version 16.0; SPSS, Chicago, IL, USA) program, and the significance of each group was verified with one-way analysis of variance (ANOVA), followed by a Tukey's multiple comparison post hoc test. P < 0.05 was considered significant.

## 3. Results

### 3.1. Performance of Classification Models

In this study, all single classification models were constructed based on AB, K-NN, CT, and RF algorithms and three descriptor sets. Subsequently, 5-fold cross-validation and test set validation were used to further evaluate the predictive power, as shown in Table 3. Among 12 NIN models, the RF algorithm had a better performance with an average MCC value of 0.725±0.014 and 0.774±0.042 from 5-fold cross validation and test set, respectively. The best single classifier was RF-c1, which was developed by random forest using DS_MOE_2D descriptors. Regarding the 12 NHN models, the best performance was also achieved by the RF algorithm, with an average MCC value of 0.857±0.026 and 0.715±0.043 from 5-fold cross-validation and test set, respectively. The best single classifier was RF-c2, which was developed by RF stacked with a DS_MOE_2D descriptor set.

The RF algorithm was more applicable than other algorithms for the classification of compounds and the prediction of neuroprotective compounds against hypoxic injury and oxidative damage. The average MCC values of each algorithm were calculated with three descriptor combinations (detailed information is presented in Figure S1). The results showed that improving the diversity of descriptors increased the predictability of the models. Therefore, classification models constructed by the DS_MOE_2D descriptor combination were selected for further studies.

**Figure 3:**
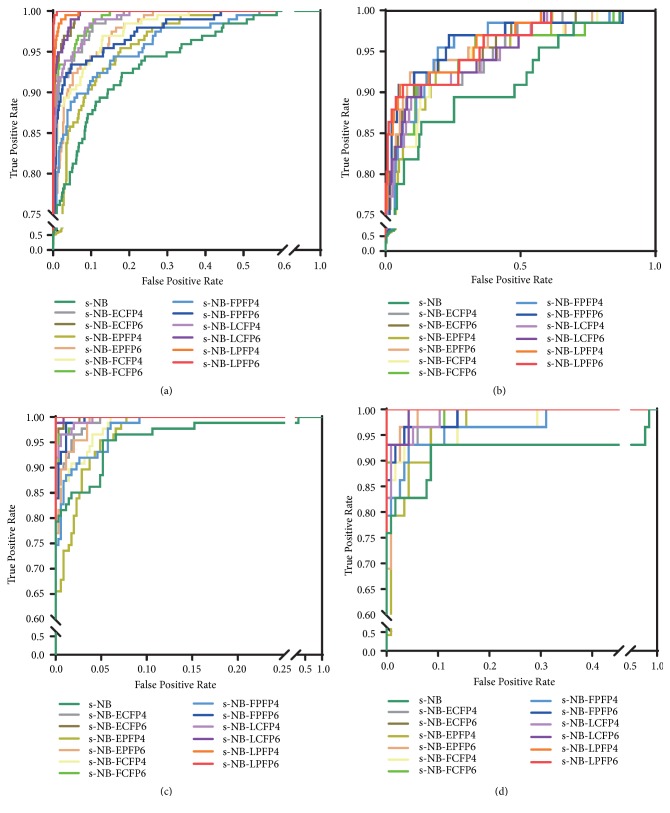
The comparison of average MCC value made by different algorithms (a and c) and different sets of descriptors (b and d) against hypoxia-induced neurotoxicity (a and b) and H2O2-induced neurotoxicity (c and d) on training set and test set.

Based on the results presented above, probability values calculated by four models were integrated into a stacked Bayesian model. Molecular fingerprint descriptors were also integrated into s-NB classification models as replenishment descriptors (detailed information is presented in Table S6). To further compare the predictive performance of the single classification models and the s-NB classification models, the MCC values and AUC values were calculated* via* a ROC plot. As shown in Figure 3, AUC values of the stacked Bayesian model s-NB-1 were 0.932 and 0.913 for the training set and the test set, respectively, and were higher than the corresponding AUC value calculated before replenishing fingerprint descriptors. For the NHN model, the verification obtained the same result.

**Figure 4:**
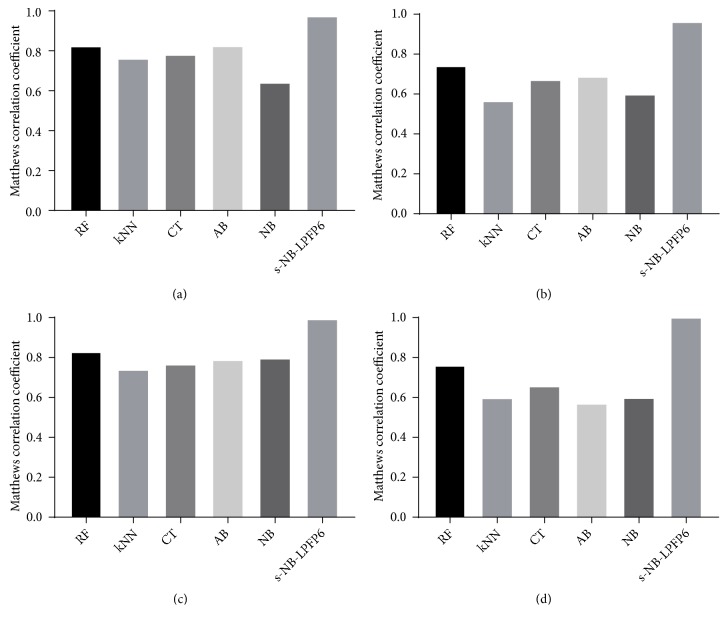
The comparison of MCC value made by four single classifiers and s-NB-LPFP6 model with different sets of descriptors against hypoxia-induced neurotoxicity (a and b) and H_2_O_2_-induced neurotoxicity (c and d) on training set (a and c) and test set (b and d).

As shown in Figure 4, s-NB-1-LPFP6 (MCC = 0.993) revealed superior predictive performance for the training set when compared with four single models (MCC ranged from 0.737 to 0.830); the same results were obtained by verification of the test set. Similarly, the MCC values of the s-NB-2-LPFP6 model were 0.968 and 0874 for the training set and the test set, respectively, and were higher than the values of the corresponding single model MCC.

### 3.2. Virtual Screening of Neuroprotective Agents from XXMD

In this study, we aimed to preliminarily clarify the functional mechanisms of XXMD and to identify potential neuroprotective agents against I/R injury; therefore, virtual screening of the compositions in the XXMD database was performed based on the two optimal classification models (s-NB-1-LPFP6 and s-NB-2-LPFP6). In total, 658 compounds were predicted to be active against ischemia/hypoxia-induced neurotoxicity. Similarly, 615 compounds were predicted to be active against H_2_O_2_-induced neurotoxicity by s-NB-2-LPFP6. A total of 398 compounds were ranked by Bayesian scoring EstPGood (0 ≤ EstPGood ≤ 1) (detailed information is presented in Table S7).

**Figure 5:**
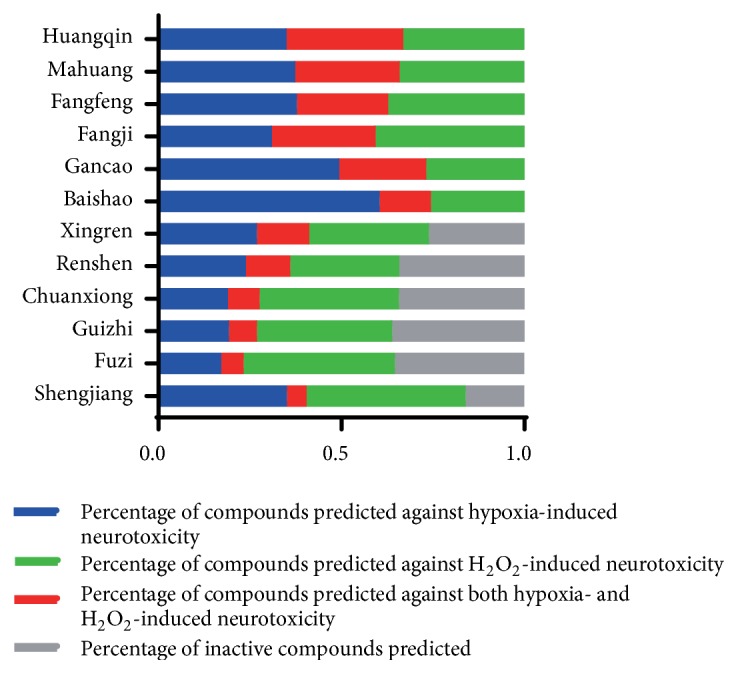
The component analysis of XXMD database. The blue part indicates the percentage of compounds predicted against hypoxia-induced neurotoxicity; the green part indicates the percentage of compounds predicted against H_2_O_2_-induced neurotoxicity; the red part indicates the percentage of compounds predicted against both hypoxia- and H_2_O_2_-induced neurotoxicity and the gray part indicates the percentage of inactive compounds predicted.

Figure 5 shows that each Chinese medicinal herb in XXMD contained antihypoxia and anti-H_2_O_2_ damage compounds. Most were predicted to exert antihypoxia and anti-H_2_O_2_ effects. It could be critical for XXMD to play a therapeutic role in I/R-induced brain injury. Flavonoid glycosides received a higher score in the antihypoxia phenotype, such as narcissoside, rutin, lsoquercitrin, hirsutrin, quercetin derivatives, and kaempferol derivatives. It has been suggested that flavonoid glycoside compounds were the major components in XXMD which acted against hypoxic phenotype in I/R-induced brain injury. Similarly, for the NHN model, alkaloids and sterol compounds in baikal skullcap root and ginseng displayed predictive activity in the anti-H_2_O_2_ phenotypes, such as pancratistatin, menisarine, fangchinoline, normenisarine, and other sterols.

In addition, 398 compounds were clustered into 5 groups by FCFP_6 fingerprint with the cluster ligands module in DS 2016. Clustering is based on the root-mean-square (RMS) difference of the Tanimoto distance for fingerprinting. For each cluster, scaffold novelty as well as probability output was considered. Finally, 10 compounds (Table 4) were identified from the XXMD database for cell-based neuroprotective assays.

In addition, to estimate the model's ability to extrapolate, the Dice similarity between 10 compounds and two predefined sets of compounds against hypoxia-induced and H_2_O_2_-induced injury were calculated by generating Morgan fingerprint using RDKit package in Python. As shown in Table 4, most of the 10 compounds are structurally similar to the representative compounds of the two predefined sets. The results also verify the prediction reliability of the two optimal classification models

### 3.3. Cell-Based Neuroprotective Assay Results

**Figure 6:**
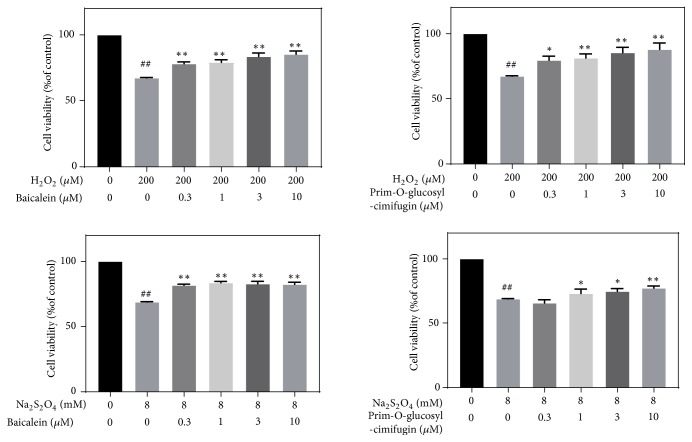
Neuroprotective effects of chemicals on H_2_O_2_-induced and Na_2_S_2_O_4_-induced SH-SY5Y cells. The viability of the untreated cells was set to 100%. The values represent mean (%) ± SD of three individual experiments (n = 3).* ##P < 0.01* versus control groups; *∗P < 0.05* and *∗∗P < 0.01* versus model group.

Cell-based neuroprotective assay results are presented in Table S8. Most of compounds showed a good dose-response relationship at different concentrations.  Figure 6 displays the neuroprotective effects of representative compounds (baicalein and prim-O-glucosylcimifugin) on H_2_O_2_-induced and Na_2_S_2_O_4_-induced SH-SY5Y cells. When compared with the control group, cell survival significantly decreased in the model group using 8 mM Na_2_S_2_O_4_ or 200 *μ*M H_2_O_2_ (P < 0.01). After treatment with baicalein (0.3 *μ*M, 1 *μ*M 3 *μ*M, and 10 *μ*M) or prim-O-glucosylcimifugin (0.3 *μ*M, 1 *μ*M 3 *μ*M, and 10 *μ*M), cell survival was significantly increased.

## 4. Discussion

In this study, compounds in XXMD were analyzed by stacked naïve Bayesian models. The results demonstrated that the existence of the 12 TCMs contained in XXMD was valuable. The synergistic effects in TCM prescriptions could be considered as the material basis for the therapeutic efficacy. Preliminary assay results suggested that a machine learning algorithm, such as combination of Bayesian models, may be feasible to predict neuroprotective compounds and preliminarily demonstrated the pharmacological mechanisms of TCM. The results suggest broadening the selection of stroke treatment methods and further demonstrated the feasibility of applying computer-assisted drug discovery to the analysis of TCM prescriptions.

Through* in silico* prediction studies, in this study, baicalein was identified as a potent neuroprotective agent, with effects on hypoxic and oxidative damage phenotypes. As a natural phenolic flavonoid compound, baicalein has attracted increased attention for antioxidant and anti-inflammatory efficacy [44]. However, studies on the efficacy of baicalein against cerebral I/R injury are limited. The validation of baicalein against two damage phenotypes supports its potential usage in ischemic stroke therapy. Moreover, prim-O-glucosylcimifugin (POG) has the highest content of chromone of* Saposhnikovia divaricate* (Turcz) Schischk (Fangfeng). However, no reports are available on the antioxidation effect of POG or its therapeutic effect on I/R-induced brain injury. In our study, we first identified the potential activity of POG against oxidant damage and hypoxia injury. These findings were subsequently verified by cell-based neuroprotective assays, which indicated that the established virtual screening pipeline could identify novel neuroprotective agents in TCM.

## Figures and Tables

**Table 1:** The total amount of each Chinese medicine compound obtained from the database.

No.	Chinese name	English name	Latin name	Number of compounds
1	Bai Shao	White Peony Root	Paeoniae Radix Alba	41
2	Chuanxiong	Sichuan lovage rhizome	Chuanxiong Rhizoma	242
3	Fangfeng	Divaricate Saposhnikovia Root	Saposhnikovia Radix	107
4	Fang Ji	Fourstamen Stephania Root	Stephaniae Tetrandrae Radix	85
5	Fuzi	Prepared Common Monkshood Daughter Root	Aconiti Lateralis Radix Praeparata	99
6	Gan Cao	Liquorice root	Glycyrrhizae Radix et Rhizoma	393
7	Guizhi	Cassia twig	Cinnamomi Ramulus	130
8	Huang Qin	Baikal Skullcap Root	Scutellariae Radix	128
9	Kuxingren	Bitter Apricot Seed	Armeniacae Semen Amarum	119
10	Ma Huang	Chinese Ephedra Herb	Ephedrae Herba	74
11	Renshen	Ginseng	Ginseng Radix et Rhizoma	272
12	Shengjiang	Fresh ginger	Zingiberis Rhizoma Recens	168
Total				1858
Remove duplicates				1484

**Table 2:** Detailed statistical description of the entire data set.

Model	Training set			Test set		
	Active	Inactive	Total	Active	Inactive	Total
Hypoxia-induced	197	792	989	66	264	330
H_2_O_2_-induced	87	348	435	29	116	145

**Table 3:** Performance of single classification models for the training set (5-fold cross-validation result) and the test set (validation result using external test set) using different combinations of molecular properties.

No.	Model	Descriptors	5-fold cross-validation result				Validation result using external test set			
			SE	SP	PPV	MCC	SE	SP	PPV	MCC
1	RF-a1	32	0.682	0.970	0.912	0.750	0.675	0.975	0.915	0.718
2	RF-b1	53	0.621	0.973	0.903	0.750	0.650	0.981	0.915	0.716
3	RF-c1	79	0.667	0.992	0.927	0.823	0.690	0.980	0.922	0.742
4	K-NN-a1	32	0.682	0.905	0.861	0.710	0.695	0.926	0.880	0.622
5	K-NN-b1	53	0.621	0.936	0.873	0.710	0.599	0.931	0.865	0.557
6	K-NN-c1	79	0.636	0.936	0.876	0.760	0.609	0.931	0.867	0.565
7	Tree-a1	32	0.621	0.943	0.879	0.680	0.635	0.942	0.881	0.609
8	Tree-b1	53	0.667	0.939	0.885	0.566	0.624	0.914	0.856	0.545
9	Tree-c1	79	0.682	0.947	0.894	0.780	0.711	0.943	0.897	0.670
10	AB-a1	32	0.682	0.920	0.873	0.659	0.731	0.900	0.867	0.603
11	AB-b1	53	0.667	0.936	0.882	0.741	0.695	0.902	0.860	0.578
12	AB-c1	79	0.621	0.928	0.867	0.824	0.741	0.941	0.901	0.687
13	NB-a1	32	0.766	0.795	0.790	0.483	0.758	0.746	0.748	0.421
14	NB-b1	53	0.777	0.904	0.879	0.644	0.682	0.894	0.852	0.555
15	NB-c1	79	0.761	0.908	0.879	0.640	0.712	0.905	0.867	0.598
16	RF-a2	26	0.805	0.994	0.956	0.860	0.655	0.991	0.924	0.710
17	RF-b2	50	0.839	0.994	0.963	0.882	0.690	0.983	0.924	0.675
18	RF-c2	65	0.747	0.997	0.947	0.830	0.724	1.000	0.945	0.761
19	K-NN-a2	26	0.793	0.960	0.926	0.766	0.724	0.957	0.910	0.574
20	K-NN-b2	50	0.747	0.945	0.906	0.702	0.724	0.957	0.910	0.585
21	K-NN-c2	65	0.782	0.951	0.917	0.739	0.793	0.957	0.924	0.597
22	Tree-a2	26	0.759	0.971	0.929	0.769	0.655	0.966	0.903	0.601
23	Tree-b2	50	0.805	0.937	0.910	0.726	0.448	0.983	0.876	0.629
24	Tree-c2	65	0.782	0.963	0.926	0.765	0.793	0.966	0.931	0.656
25	AB-a2	26	0.805	0.937	0.910	0.726	0.655	0.957	0.897	0.602
26	AB-b2	50	0.828	0.931	0.910	0.732	0.793	0.948	0.917	0.621
27	AB-c2	65	0.851	0.951	0.931	0.788	0.828	0.974	0.945	0.570
28	NB-a2	26	0.839	0.951	0.929	0.780	0.724	0.871	0.841	0.551
29	NB-b2	50	0.816	0.966	0.936	0.796	0.621	0.914	0.855	0.542
30	NB-c2	65	0.885	0.943	0.931	0.795	0.712	0.905	0.867	0.598

1-15: neuroprotective models against hypoxia-induced neurotoxicity (NIN models).

16-30: neuroprotective models against H_2_O_2_-induced neurotoxicity (NHN models).

a: models built by DS_2D descriptors.

b: models built by MOE_2D descriptors.

c: models built by DS_MOE 2D descriptors.

**Table 4:** Chemical structures of representative compounds predicted by two phenotypic screening models in XXMD.

ID	Name	Structure	Bayesian model		Bayesian model		Most similar compound in training sets
			(s-NB-1-LPFP6)		(s-NB-2-LPFP6)		
			EstPGood	Prediction	EstPGood	Prediction	
PubChemCID 21670038	5-*O*-methylvisammioside	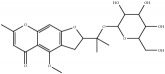	0.993	TRUE	0.133	TRUE	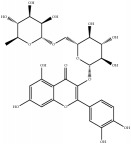
CHEMBL 8260	Baicalein	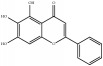	0.999	TRUE	0.349	TRUE	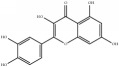
CHEMBL 485818	Baicalein	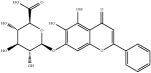	1.000	TRUE	0.397	TRUE	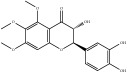
PubChemCID 5281607	Chrysin	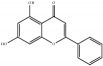	0.994	TRUE	0.183	TRUE	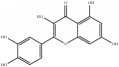
PubChemCID 441960	Cimifugin	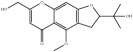	0.881	TRUE	0.183	TRUE	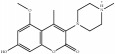
CHEMBL 504256	Fangchinoline	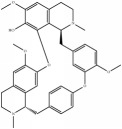	0.828	TRUE	0.727	TRUE	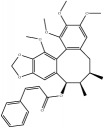
CHEMBL 1734606	Prim-O-glucosylcimifugin	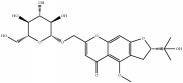	0.996	TRUE	0.101	TRUE	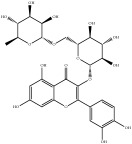
CHEMBL 176045	Tetrandrine	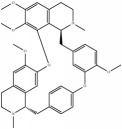	0.754	TRUE	0.787	TRUE	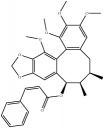
CHEMBL 16171	Wogonin	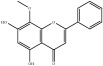	0.994	TRUE	0.416	TRUE	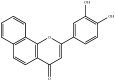
PubChemCID 12004622	Wogonoside	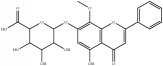	1.000	TRUE	0.441	TRUE	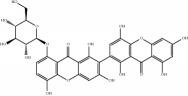

## Data Availability

The data used to support the findings of this study are available from the corresponding author upon request.

## Additional Points

Machine learning models were used to demonstrate the pharmacological mechanisms of classic traditional Chinese medicine prescriptions. Single classifiers and molecular fingerprint descriptors were integrated to improve the predictive power of naïve Bayesian models. The stacked naïve Bayesian models were applied to predict neuroprotective compounds for ischemic stroke. Two compounds gained using above protocols were confirmed by cell-based assays.

## Supplementary Materials

Supplementary Materials The supplementary materials contain the detailed information of the training sets and test sets used in the experiment, the initial evaluation of the predictive power for single algorithms and stacked models, the detailed predictions of compounds contained in XXMD, and* in vitro* detailed assay results.
