# Supplementary material for: Evaluation and Identification of the Neuroprotective Compounds of Xiaoxuming Decoction by Machine Learning: A Novel Mode to Explore the Combination Rules in Traditional Chinese Medicine Prescription
Source: Biomed Res Int. 2019 Jul 10;2019:6847685. doi: 10.1155/2019/6847685 (PMC6652039; doi:10.1155/2019/6847685)
Supplement: Supplementary Materials — The supplementary materials contain the detailed information of the training sets and test sets used in the experiment, the initial evaluation of the predictive power for single algorithms and stacked models, the detailed predictions of compounds contained in XXMD, and in vitro detailed assay results. [file 6847685.f1.zip › Supplementary Materials_BMRI_2778850.docx]

# BioMed Research International

# Supplementary Materials

# Evaluation and identification of the neuroprotective compounds of Xiaoxuming decoction by machine learning: a novel mode to explore the combination rules in traditional Chinese medicine prescription

Shilun Yang,^1,2^ Yanjia Shen,^2^ Wendan Lu,^2^ Yinglin Yang,^2^ Haigang Wang,^2^ Li Li,^2^ Chunfu Wu,^1,*^ and Guanhua Du^1,2,*^

**Figure of Contents**

**Fig. S1** The comparison of average MCC value made by different algorithms (A and B) and different sets of descriptors (C and D) against hypoxia-induced neurotoxicity (A and C) and H2O2-induced neurotoxicity (B and D) on training set and test set.

**Fig. S1**


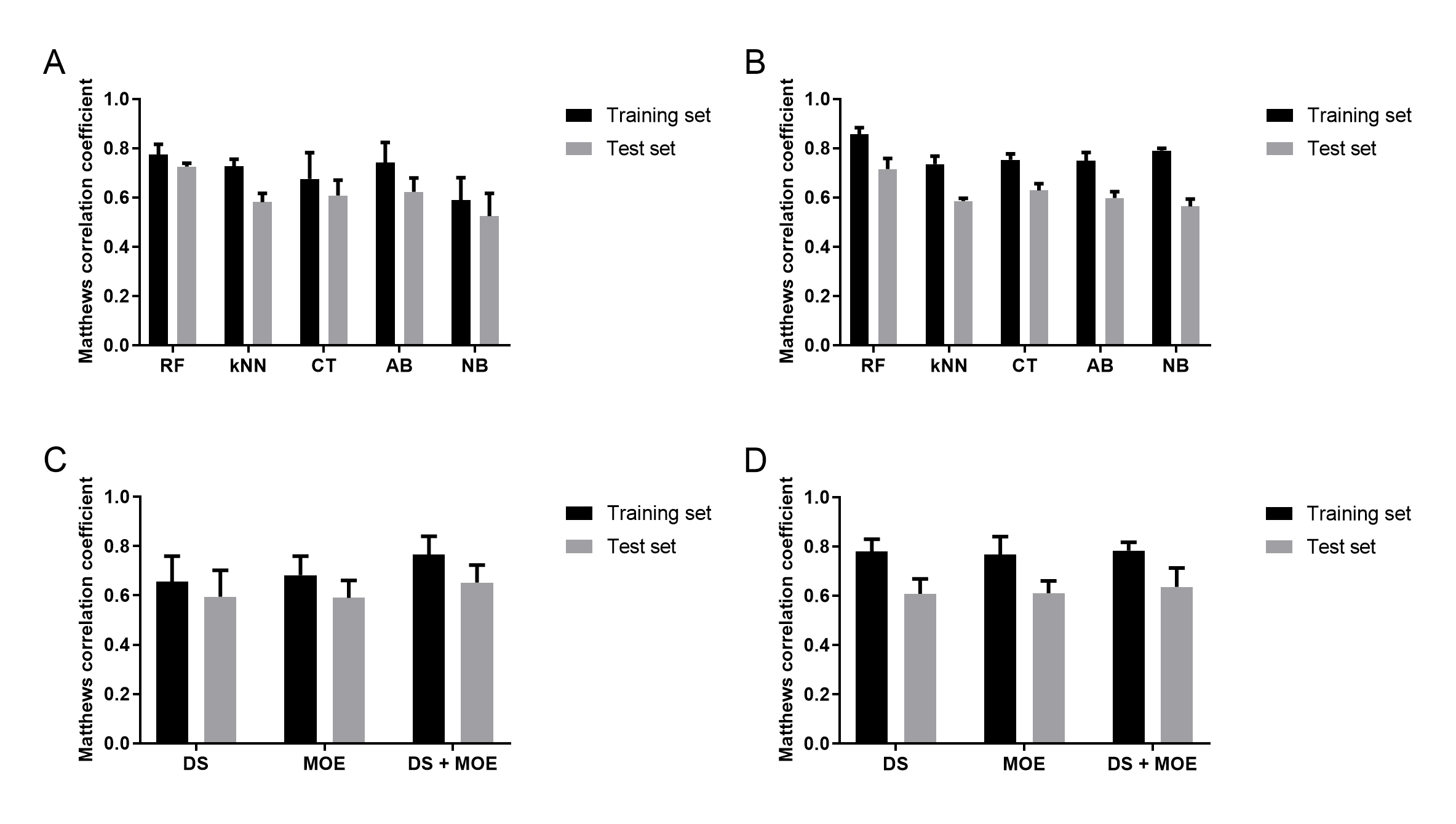


**Table of Contents**

**Table S1.** Structures of the 989 Compounds (in SMILE format) from the training set together with their activities (1 or 0) for ischemia-induced models.

**Table S2.** Structures of the 330 Compounds (in SMILE format) from the test set together with their activities (1 or 0) for glutamate-induced models.

**Table S3.** Structures of the 435 Compounds (in SMILE format) from the training set together with their activities (1 or 0) for H2O2-induced models.

**Table S4.** Structures of the 145 Compounds (in SMILE format) from the test set together with their activities (1 or 0) for H2O2-induced models.

**Table S5.** Molecular descriptors used in this work.

**Table S6.** Performance of the 26 Bayesian classification models for the training set and test set using different combinational of output probabilities and fingerprints.

**Table S7** Detailed prediction results of the 398 compounds predicted active by two phenotypic screening models.

**Table S8.** Neuroprotective effects of compounds on H_2_O_2_ or Na_2_S_2_O_4_-induced neurotoxicity on SH-SY5Y cells.

**Table S1.** Structures of the 989 Compounds (in SMILE format) from the training set together with their activities (1 or 0) for ischemia-induced models.

| ChEMBL ID | Structure | Activity |
| --- | --- | --- |
| CHEMBL1094995 | O1C(C(=O)NC)C(O)C(O)C1n1c2ncnc(Nc3ccc(cc3)CCNC(=O)c3cc(cc(c3)C(C)(C)C)C(C)(C)C)c2nc1 | 1 |
| CHEMBL1094996 | O1[C@H](C(=O)NC)[C@@H](O)[C@@H](O)[C@@H]1n1c2ncnc(Nc3ccc(cc3)CCNC(=O)c3cc(OC)c(OC)c(OC)c3)c2nc1 | 1 |
| CHEMBL1094998 | O1[C@H](C(=O)NC)[C@@H](O)[C@@H](O)[C@@H]1n1c2ncnc(NCc3ccc(cc3)CNC(=O)c3cc(C(C)(C)C)c(O)c(c3)C(C)(C)C)c2nc1 | 1 |
| CHEMBL1094999 | O1[C@H](C(=O)NC)[C@@H](O)[C@@H](O)[C@@H]1n1c2ncnc(NCc3ccc(cc3)CNC(=O)c3cc(cc(c3)C(C)(C)C)C(C)(C)C)c2nc1 | 1 |
| CHEMBL1095000 | O1[C@H](C(=O)NC)[C@@H](O)[C@@H](O)[C@@H]1n1c2ncnc(NCc3ccc(cc3)CNC(=O)c3cc(OC)c(OC)c(OC)c3)c2nc1 | 1 |
| CHEMBL115471 | Clc1nc(NC(COc2ccccc2)C)c2ncn(c2n1)[C@@H]1O[C@H](CO)[C@@H](O)[C@H]1O | 1 |
| CHEMBL116565 | Oc1nc2cc([N+](=O)[O-])c(-n3cc(nc3)COC(=O)Nc3ccc(cc3)C(=O)[O-])cc2nc1C(=O)[O-] | 1 |
| CHEMBL1172243 | O1[C@H]([C@H](O)[C@@H](O)[C@H](O)[C@H]1CO)C1(O)C([O-])=C([C@@H]2O[C@H](CO)[C@@H](O)[C@H](O)[C@H]2O)C(=O)C(C(=O)\C=C\c2ccc(O)cc2)=C1[O-] | 1 |
| CHEMBL1173348 | Fc1cc2c3C(=NNC2=O)c2cc(ccc2Oc3c1)C[NH+]1CC[NH+](CC1)C | 1 |
| CHEMBL117904 | O1[C@H](CO)[C@@H](O)[C@@H](OC)[C@@H]1n1c2ncnc(NC3CCCCC3)c2nc1 | 1 |
| CHEMBL117974 | Clc1nc(NN2CCC(Sc3ccccc3)CC2)c2ncn(c2n1)[C@@H]1O[C@H](CCl)[C@@H](O)[C@H]1O | 1 |
| CHEMBL119625 | O=C1N(c2cc(-n3ccnc3)c([N+](=O)[O-])cc2NC1=O)CC(=O)[O-] | 1 |
| CHEMBL139000 | O1[C@H](CO)[C@@H](O)[C@@H](O)[C@@H]1n1c2ncnc(N[C@@H](Cc3ccccc3)C)c2nc1 | 1 |
| CHEMBL1404 | O(CC(O)C[NH+]1CC[NH+](CC1)CC(=O)Nc1c(cccc1C)C)c1ccccc1OC | 1 |
| CHEMBL145968 | Clc1ccc(SC)cc1NC(=[NH2+])N(C)c1cc(S(=O)C)ccc1 | 1 |
| CHEMBL1482959 | O1c2c(ccc3c2cccc3)C(=O)C=C1c1cc(O)c(O)cc1 | 1 |
| CHEMBL1614652 | S1(=O)(=O)N(CCCC[NH2+]C[C@@H]2Oc3c(CC2)cccc3)C(=O)c2c1cccc2 | 1 |
| CHEMBL164575 | O1C(C(O)CO)C(=O)C(OCCCCCCCCCCCCCCC)=C1[O-] | 1 |
| CHEMBL1760960 | Brc1cc2c(OC(CC23OC(=O)N(Cc2ccc(NC(=O)C)cc2)C3=N)(C)C)cc1 | 1 |
| CHEMBL1782854 | O=C(N[C@@H](CC(C)C)C(=O)N[C@@H](Cc1ccccc1)C(=O)N[C@@H](Cc1c2c([nH]c1)cccc2)C(=O)[O-])[C@H]1[NH2+]Cc2[nH]c3c(c2C1)cccc3 | 1 |
| CHEMBL1818140 | O1c2c(cc(-n3nnc(c3)Cn3ccnc3[N+](=O)[O-])cc2)C(=O)C(O)=C1c1cc(O)c(O)cc1 | 1 |
| CHEMBL188018 | FC(F)(F)c1cc2nc(O)c(nc2cc1-n1cc(nc1)COC(=O)Nc1ccc(cc1)C(=O)[O-])C(=O)[O-] | 1 |
| CHEMBL1951816 | O1c2c(cc(C)c(c2)C)C(=O)C(O)=C1c1cc(OCC)c(OCC)cc1 | 1 |
| CHEMBL1951819 | O1c2c(ccc3c2cccc3)C(=O)C(O)=C1c1cc(OCC)c(OCC)cc1 | 1 |
| CHEMBL1951822 | O1c2c(c3c(cc2)cccc3)C(=O)C(O)=C1c1cc(O)c(O)cc1 | 1 |
| CHEMBL1951823 | O1c2c(cc(C)c(c2)C)C(=O)C=C1c1cc(OC)c(OCc2ccccc2)cc1 | 1 |
| CHEMBL1951824 | O1c2c(cc(C)c(c2)C)C(=O)C(O)=C1c1cc(OC)c(OCc2ccccc2)cc1 | 1 |
| CHEMBL1951826 | O1c2c(ccc3c2cccc3)C(=O)C(O)=C1c1cc(OC)c(OCc2ccccc2)cc1 | 1 |
| CHEMBL1951827 | O1c2c(cc(C)c(c2)C)C(=O)C(O)=C1c1cc(OC)c(O)cc1 | 1 |
| CHEMBL1951830 | O1c2c(cc(C)c(c2)C)C(=O)C(O)=C1c1cc(O)c(OC)cc1 | 1 |
| CHEMBL1951834 | O1c2c(ccc3c2cccc3)C(=O)C=C1c1ccc(N2CCCC2)cc1 | 1 |
| CHEMBL1951835 | O1c2c(cc(C)c(c2)C)C(=O)C=C1c1ccc(N2CCCC2)cc1 | 1 |
| CHEMBL1951836 | O1c2c(cc(C)c(c2)C)C(=O)C(O)=C1c1ccc(N(C)C)cc1 | 1 |
| CHEMBL1951837 | O1c2c(ccc3c2cccc3)C(=O)C(O)=C1c1ccc(N(C)C)cc1 | 1 |
| CHEMBL1951839 | O1c2c(ccc3c2cccc3)C(=O)C=C1c1ccc(N(C)C)cc1 | 1 |
| CHEMBL1951840 | O1c2c(cc(C)c(c2)C)C(=O)C(O)=C1c1ccc(N2CCCC2)cc1 | 1 |
| CHEMBL1951846 | Oc1c2c(ccc1C(=O)\C=C\c1cc(O)c(O)cc1)cccc2 | 1 |
| CHEMBL1951847 | O(Cc1ccccc1)c1ccc(cc1OC)\C=C\C(=O)c1ccc2c(cccc2)c1O | 1 |
| CHEMBL1951863 | O(C(C)C)c1cc(nc2c1cccc2)-c1ccc(N(C)C)cc1 | 1 |
| CHEMBL1951864 | O(C(C)C)c1cc(nc2c1cccc2)-c1ccc(N2CCCC2)cc1 | 1 |
| CHEMBL1951865 | O(C1CCCC1)c1cc(nc2c1cccc2)-c1cc(O)c(O)cc1 | 1 |
| CHEMBL204505 | Clc1ccc(cc1)C(=O)n1c2c(cc(OC)cc2)c(CC(=O)N2CCCC2C(=O)NC(C(OCC)=O)CS)c1C | 1 |
| CHEMBL207440 | S=C(Nc1ccccc1)N[C@@H](CC(C)C)C(=O)N[C@H]1CCOC1O | 1 |
| CHEMBL2115381 | O1[C@H](C(O)CO)C([O-])=C(OCCCCCCCCCCCCCCCCCC)C1=O | 1 |
| CHEMBL213556 | FC(F)(F)C(O)(C(F)(F)F)C1ON=C(C1)C(=O)NC(CC(OC(C)(C)C)=O)C | 1 |
| CHEMBL2170610 | FC(F)(F)c1cc(ccc1C1CCN(CC1)C(=O)C)C(=O)NC(N)=N | 1 |
| CHEMBL2171396 | Fc1ccccc1-n1nc(cc1OC)C(=O)N[C@@H](CC(=O)[O-])c1ccccc1C | 1 |
| CHEMBL2181202 | Oc1cc(C)c(C[C@H]([NH3+])C(=O)N[C@H](CCCNC(=[NH2+])N)C(=O)N[C@@H](Cc2ccccc2)C(=O)N[C@@H](CCCC[NH3+])C(=O)N)c(c1)C | 1 |
| CHEMBL222519 | S(=O)(=O)(N)c1c2c(c3nc(O)c(O)nc3cc2[N+](=O)[O-])ccc1 | 1 |
| CHEMBL226335 | O1c2c(C(=O)C(O[C@@H]3O[C@H](CO[C@@H]4O[C@@H](C)[C@H](O)[C@@H](O)[C@H]4O)[C@@H](O)[C@H](O)[C@H]3O)=C1c1cc(O)c(O)cc1)c(O)cc(O)c2 | 1 |
| CHEMBL229043 | Brc1c2c(cccc2)c(cc1)CCO[C@@H]1CCCC[C@H]1[NH+]1CC(=O)CC1 | 1 |
| CHEMBL229115 | s1cc(c2c1cccc2)CCO[C@@H]1CCCC[C@H]1[NH+]1CCOCC1 | 1 |
| CHEMBL229352 | O1CC[NH+](CC1)[C@@H]1CCCC[C@H]1OCCCc1ccc(OC)cc1OC | 1 |
| CHEMBL229634 | FC(F)(F)c1ccccc1CCO[C@H]1CCCC[C@H]1[NH+]1CCOCC1 | 1 |
| CHEMBL229775 | Brc1cc(ccc1)CCO[C@@H]1CCCC[C@H]1[NH+]1CCOCC1 | 1 |
| CHEMBL229829 | O(CCc1cc2c(cc1)cccc2)[C@@H]1CCCC[C@H]1[NH+](CCOC)CCOC | 1 |
| CHEMBL229830 | O1CC[NH+](CC1)[C@@H]1CCCC[C@H]1OCCCc1cc(OC)c(OC)cc1 | 1 |
| CHEMBL229892 | Brc1ccccc1CCO[C@@H]1CCCC[C@H]1[NH+]1CC(=O)CC1 | 1 |
| CHEMBL229933 | O1CC[NH+](CC1)[C@@H]1CCCC[C@H]1OCCOc1cc2c(cc1)cccc2 | 1 |
| CHEMBL229942 | Clc1cc(Cl)ccc1CCO[C@@H]1CCCC[C@H]1[NH+]1CCOCC1 | 1 |
| CHEMBL232940 | Clc1cc(-c2oc(C(=O)N=C(N)N)c(N)c2)c(OC)cc1 | 1 |
| CHEMBL241401 | Brc1cc2OCOc2cc1\C=[N+](/[O-])\C(C)(C)C | 1 |
| CHEMBL2419402 | O1c2c(C(C)=C(N3CC[NH+](CC3)C)C1=O)c(OC)cc(O)c2 | 1 |
| CHEMBL280974 | Clc1ccc(cc1)[C@@H](O)C[NH+]1CCC(CC1)Cc1ccc(F)cc1 | 1 |
| CHEMBL28564 | Clc1ccc(cc1)C(O)C[NH+]1CCC(CC1)Cc1ccc(F)cc1 | 1 |
| CHEMBL28565 | Clc1ccc(cc1)[C@H](O)C[NH+]1CCC(CC1)Cc1ccc(F)cc1 | 1 |
| CHEMBL293212 | Clc1cc(ccc1)C[NH2+]CCc1ccc(NC(=[NH2+])c2sccc2)cc1 | 1 |
| CHEMBL3099553 | S1SCC[C@H]1CCCCC(=O)N[C@@H](CC(=O)[O-])C(=O)NCC(=O)[O-] | 1 |
| CHEMBL3099554 | S1SCC[C@H]1CCCCC(=O)N[C@@H](Cc1ccc(O)cc1)C(=O)N[C@H](C(=O)[O-])C | 1 |
| CHEMBL3099557 | S1SCC[C@H]1CCCCC(=O)N[C@@H](Cc1ccc(cc1)C(=O)[O-])C(=O)[O-] | 1 |
| CHEMBL3099558 | S1SCC[C@H]1CCCCC(=O)N[C@@H](Cc1ccc(O)cc1)C(=O)[O-] | 1 |
| CHEMBL3099560 | S1SCC[C@H]1CCCCC(=O)N[C@@H](CCC(=O)[O-])C(=O)[O-] | 1 |
| CHEMBL3099561 | S1SCC[C@H]1CCCCC(=O)N[C@H](C(=O)[O-])CS(=O)(=O)[O-] | 1 |
| CHEMBL3099564 | S1SCC[C@H]1CCCCC(=O)NCCS(=O)(=O)[O-] | 1 |
| CHEMBL3099565 | S1SCC[C@H]1CCCCC(=O)NCCOS(=O)(=O)[O-] | 1 |
| CHEMBL3099566 | S1SCC[C@H]1CCCCC(=O)NCCP(=O)([O-])[O-] | 1 |
| CHEMBL3099567 | S1SCC[C@H]1CCCCC(=O)NCCOP(=O)([O-])[O-] | 1 |
| CHEMBL3099568 | S1SCC[C@H]1CCCCC(=O)Nc1ccc(cc1)C(=O)[O-] | 1 |
| CHEMBL3099569 | S1SCC[C@H]1CCCCC(=O)Nc1cc(S(=O)(=O)[O-])ccc1 | 1 |
| CHEMBL3099570 | S1SCC[C@H]1CCCCC(=O)Nc1ccc(S(=O)(=O)[O-])cc1 | 1 |
| CHEMBL3113826 | O=C1[C@H]2C[C@H](O)CC[C@@]2([C@@H]2[C@H]([C@@H]3CC[C@H]([C@@H](CCC(OC)=O)C)[C@]3(CC2)C)[C@@H]1O)C | 1 |
| CHEMBL327073 | Clc1nc(N[C@H](COc2ccccc2)C)c2ncn(c2n1)[C@@H]1O[C@H](CO)[C@@H](O)[C@H]1O | 1 |
| CHEMBL3290184 | Fc1ccc(cc1O)\C=C\C(=O)Nc1c2c(ccc1)cccc2 | 1 |
| CHEMBL331372 | O1[C@H](C(=O)NCC)[C@@H](O)[C@@H](O)[C@@H]1n1c2nc(nc(N)c2nc1)NCCc1ccc(cc1)CCC(=O)[O-] | 1 |
| CHEMBL3322888 | O1c2cc(OC)c-3c(c2OC1)[C@H](N(c1c-3cc(OC)c2cc(OC)c(OC)cc12)C)CC(=O)C | 1 |
| CHEMBL3322889 | O1c2cc(OC)c-3c(c2OC1)[C@@H](N(c1c-3cc(OC)c2cc(OC)c(OC)cc12)C)CO | 1 |
| CHEMBL3322890 | O1c2cc(OC)c-3c(c2OC1)[C@H](N(c1c-3cc(OC)c2cc(OC)c(OC)cc12)C)CO | 1 |
| CHEMBL3322891 | O1c2c3c(-c4cc(OC)c5cc(OC)c(OC)cc5c4N(C)C3CC(=O)CC(O)(C)C)c(OC)cc2OC1 | 1 |
| CHEMBL3397733 | O(Cc1ccc(cc1)CO[N+](=O)[O-])C(=O)[C@H](NC(=O)C1CCC(CC1)C(C)C)Cc1ccccc1 | 1 |
| CHEMBL3398195 | Clc1cc2c(n(cc2C2=C(C(=O)NC2=O)c2n3c(nn2)C=CC=C3)C)cc1 | 1 |
| CHEMBL3600453 | Brc1[nH]c(Br)nc1C[C@H](NC(=O)c1nccnc1)C(=O)N1CCC[C@H]1C(=O)N | 1 |
| CHEMBL3600858 | [O-]\[N+](\C)=C\1/CC[C@@]2([C@@H]3[C@H]([C@@H]4CC[C@H]([C@@H](CCCC(C)C)C)[C@]4(CC3)C)CCC2=C/1)C | 1 |
| CHEMBL3715569 | Clc1c2c(sc1C(=O)N1CCCc3cc(ccc13)C)cccc2 | 1 |
| CHEMBL3716221 | Clc1cc(ccc1Cl)CC=1C(Oc2c(ccc(O)c2)C=1C)=O | 1 |
| CHEMBL3730363 | Clc1ccc(SC(C(=O)Nc2ccc(F)cc2)c2ccccc2)cc1 | 1 |
| CHEMBL373445 | Brc1cc2c(OC(CC23OCC(=O)N(C3)Cc2ccc(NS(=O)(=O)C)cc2)(C)C)cc1 | 1 |
| CHEMBL3735369 | Clc1cccc(Cl)c1OCC([NH2+]Cc1cc2c(cc1)C(N(O)C2(C)C)(C)C)C | 1 |
| CHEMBL3736226 | O(CC([NH2+]Cc1cc2c(cc1)C(N(O)C2(C)C)(C)C)C)c1c(cccc1C)C | 1 |
| CHEMBL374085 | O1c2c(cccc2)C2(OCC(=O)N(C2)Cc2ccc(NC(=O)C)cc2)CC1(C)C | 1 |
| CHEMBL375463 | Clc1cccc(Cl)c1CCO[C@@H]1CCCC[C@H]1[NH+]1CCOCC1 | 1 |
| CHEMBL375464 | s1cc(c2c1cccc2)CCO[C@@H]1CCCC[C@H]1[NH+]1CC(=O)CC1 | 1 |
| CHEMBL3786354 | O(CC(=O)NCCNC(=O)[C@@H](NC(=O)[C@@H]([NH3+])CCCNC(=[NH2+])N)Cc1ccc(O)cc1)c1ccc(cc1)C1=[N+]([O-])C(C)(C)C(N1O)(C)C | 1 |
| CHEMBL3787168 | O(CC(=O)NCCCNC(=O)[C@@H](NC(=O)[C@@H]([NH3+])CCCNC(=[NH2+])N)Cc1ccc(O)cc1)c1ccc(cc1)C1=[N+]([O-])C(C)(C)C(N1O)(C)C | 1 |
| CHEMBL3793387 | Clc1cc(Cl)cc(CNc2cc(O)c(cc2)C(OC2CCCCC2)=O)c1O | 1 |
| CHEMBL3793960 | Clc1cc(Cl)cc(CNc2cc(O)c(cc2)C(OCC)=O)c1O | 1 |
| CHEMBL382194 | Ic1cc(ccc1)CNc1ncnc2n(cnc12)[C@@H]1O[C@H](CO)[C@@H](NC(=O)N)[C@H]1O | 1 |
| CHEMBL3827588 | O(CC(OC)=O)c1ccc(cc1OC)\C=C\C(=O)CC(=O)\C=C\c1cc(OC)c(OC)cc1 | 1 |
| CHEMBL387911 | O(CCc1c2c(ccc1)cccc2)[C@@H]1CCCC[C@H]1[NH+]1CCCC1 | 1 |
| CHEMBL390386 | O=C1CC[NH+](C1)[C@@H]1CCCC[C@@H]1OCCc1c2c(ccc1)cccc2 | 1 |
| CHEMBL423315 | Clc1nc(NN2CCCCC2)c2ncn(c2n1)[C@@H]1O[C@H](CO)[C@@H](O)[C@H]1O | 1 |
| CHEMBL424553 | O1C(C(O)CO)C(=O)C(OCCCCCCCCCCCCCC)=C1[O-] | 1 |
| CHEMBL435073 | O1C(C(=O)N2CC[NH+](CC2)CC(=O)Nc2c3c(ccc2)C(=O)NC3)[C@@H](O)[C@@H](O)C1n1c2ncnc(N)c2nc1 | 1 |
| CHEMBL438826 | Brc1cc2c(OC(CC23OCC[NH+](C3)Cc2ccc(NS(=O)(=O)C)cc2)(C)C)cc1 | 1 |
| CHEMBL452043 | s1nc(OCCC[NH+](CCc2cc(OC)c(OC)cc2)C)nc1-c1ccccc1 | 1 |
| CHEMBL455938 | O1c2c(cccc2)C2(OCC(=O)N(C2)Cc2ccccc2OC)CC1(C)C | 1 |
| CHEMBL455960 | Brc1cc2c(OC(CC23OCC[NH+](C3)Cc2cc(OC)ccc2)(C)C)cc1 | 1 |
| CHEMBL456824 | Brc1cc2c(OC(CC23OCC[NH+](C3)Cc2ccccc2)(C)C)cc1 | 1 |
| CHEMBL457008 | Brc1cc2c(OC(CC23OCC(=O)N(C3)Cc2ccc(N)cc2)(C)C)cc1 | 1 |
| CHEMBL457009 | Brc1cc2c(OC(CC23OCC(=O)N(C3)Cc2ccc(cc2)C)(C)C)cc1 | 1 |
| CHEMBL457011 | Brc1cc2c(OC(CC23OCC(=O)N(C3)Cc2ccc(cc2)C(F)(F)F)(C)C)cc1 | 1 |
| CHEMBL457420 | Brc1cc2c(OC(CC23OCC[NH+](C3)Cc2ccc(OC)cc2)(C)C)cc1 | 1 |
| CHEMBL457638 | Brc1ccc(cc1)CN1CC2(OCC1=O)CC(Oc1c2cccc1)(C)C | 1 |
| CHEMBL457651 | Brc1cc2c(OC(CC23OCC(=O)N(C3)Cc2ccc(OC)cc2)(C)C)cc1 | 1 |
| CHEMBL457652 | Brc1cc2c(OC(CC23OCC(=O)N(C3)Cc2ccccc2OC)(C)C)cc1 | 1 |
| CHEMBL458303 | Brc1cc2c(OC(CC23OCC[NH+](C3)Cc2ccc(cc2)C(F)(F)F)(C)C)cc1 | 1 |
| CHEMBL459767 | Brc1ccc(cc1)C[NH+]1CC2(OCC1)CC(Oc1c2cccc1)(C)C | 1 |
| CHEMBL459768 | O1c2c(cccc2)C2(OCC[NH+](C2)Cc2ccccc2OC)CC1(C)C | 1 |
| CHEMBL460392 | S(=O)(=O)(Nc1ccc(cc1)C[NH+]1CC2(OCC1)CC(Oc1c2cccc1)(C)C)C | 1 |
| CHEMBL464181 | S(=O)(=O)(Nc1ccc(cc1)CN1CC2(OCC1=O)CC(Oc1c2cccc1)(C)C)C | 1 |
| CHEMBL47 | O1c2c(CC[C@]1(CCC[C@@H](CCC[C@@H](CCCC(C)C)C)C)C)c(C)c(O)c(C)c2C | 1 |
| CHEMBL471737 | O(C)c1cc2c(cc1OC)C[C@@H]2C[NH+](CCCN1CCc2cc(OC)c(OC)cc2CC1=O)C | 1 |
| CHEMBL474212 | Clc1c(C)c(c2OC(CN(c2c1C)C(=O)C[NH+](CC)CC)c1ccccc1)C | 1 |
| CHEMBL474213 | O1c2c(N(CC1c1ccccc1)C(=O)C[NH+](CC)CC)c(cc(C)c2C)C | 1 |
| CHEMBL475227 | Clc1c(C)c(c2OCCN(c2c1C)C(=O)C[NH+](CC)CC)C | 1 |
| CHEMBL485818 | O1c2c(c(O)c(O)c(O[C@@H]3O[C@H](C(=O)[O-])[C@@H](O)[C@H](O)[C@H]3O)c2)C(=O)C=C1c1ccccc1 | 1 |
| CHEMBL486256 | S1C[C@H]([NH2+]C[C@H](OC)CSc2ccccc2OC)COc2c1cccc2 | 1 |
| CHEMBL492438 | Clc1cc(ccc1Cl)CC[NH2+]C[C@H](O)COc1ccc(NS(=O)(=O)C)cc1 | 1 |
| CHEMBL493354 | Clc1c(C)c(c2O[C@H](CN(c2c1C)C(=O)C[NH+](CC)CC)c1ccccc1)C | 1 |
| CHEMBL493549 | O1c2c(N(CC1(C(OCC)=O)C)C(=O)C[NH+](CC)CC)c(cc(C)c2C)C | 1 |
| CHEMBL493550 | O1c2c(N(CC1c1ccccc1)C(=O)C[NH+](CC)CC)c(C)c(OC)c(C)c2C | 1 |
| CHEMBL496669 | OCC(NC(=O)C\C=C\C\C=C\C\C=C\CCCCCCCC)C(=O)[O-] | 1 |
| CHEMBL496671 | OCC(NC(=O)\C=C(\C=C\C=C(\C=C\C=1C(CCCC=1C)(C)C)/C)/C)C(=O)[O-] | 1 |
| CHEMBL496869 | OCC(NC(=O)[C@]1(C2CC=C3[C@@H](CCC(=C3)C(C)C)[C@]2(CCC1)C)C)C(=O)[O-] | 1 |
| CHEMBL496877 | O(C(=O)C(NC(=O)CCCCCCC\C=C\C\C=C\CCCCC)c1nnn[n-]1)CC | 1 |
| CHEMBL496878 | O1C(C(O)COC(=O)CCCCCNC(=O)CCCCCCCCCCCCCCCCC)C([O-])=C(O)C1=O | 1 |
| CHEMBL497730 | OC1(\C=C\C(=C\C(=O)NC(C(OCC)=O)CO)\C)C(CC(=O)C=C1C)(C)C | 1 |
| CHEMBL497731 | OC1(\C=C\C(=C\C(=O)NC(C(=O)[O-])CO)\C)C(CC(=O)C=C1C)(C)C | 1 |
| CHEMBL498754 | P(=O)([O-])([O-])C(NC(=O)CCCCCCCCCCCCCCCCC)CO | 1 |
| CHEMBL500432 | OCC(NC(=O)CCCC\C=C\C\C=C\CCCCCCCC)C(=O)[O-] | 1 |
| CHEMBL501217 | O(C(=O)C(NC(=O)CCCCCCCCCCCCCCCCC)CO)CC | 1 |
| CHEMBL501478 | OCC(NC(=O)CCCCCCCCCCCCCCCCC)C(=O)[O-] | 1 |
| CHEMBL506530 | O1c2c(cccc2)C2(OCC[NH+](C2)Cc2ccccc2)CC1(C)C | 1 |
| CHEMBL509130 | S1C[C@H]([NH2+]C[C@H](CC)CSc2ccccc2OC)COc2c1cccc2 | 1 |
| CHEMBL509634 | S1C[C@H]([NH2+]C[C@H](C(C)C)CSc2ccccc2OC)COc2c1cccc2 | 1 |
| CHEMBL509727 | O1c2c(cccc2)C2(OCC(=O)N(C2)Cc2cc(OC)ccc2)CC1(C)C | 1 |
| CHEMBL509974 | Brc1cc2c(OC(CC23OCC[NH+](C3)Cc2ccc(cc2)C)(C)C)cc1 | 1 |
| CHEMBL510870 | O1c2c(cccc2)C2(OCC(=O)N(C2)Cc2ccc(OC)cc2)CC1(C)C | 1 |
| CHEMBL514255 | Brc1cc2c(OC(CC23OCC[NH+](C3)Cc2ccc(N)cc2)(C)C)cc1 | 1 |
| CHEMBL514581 | O1c2c(cccc2)C2(OCC[NH+](C2)Cc2cc(OC)ccc2)CC1(C)C | 1 |
| CHEMBL515266 | O1c2c(c(C)c(O)c(C)c2C)C(NC(=O)C[NH+](CC)CC)CC1c1ccccc1 | 1 |
| CHEMBL515989 | Brc1cc2c(OC(CC23OCC[NH+](C3)Cc2ccccc2OC)(C)C)cc1 | 1 |
| CHEMBL516134 | O1c2c(cccc2)C2(OCC(=O)N(C2)Cc2ccc(cc2)C)CC1(C)C | 1 |
| CHEMBL516779 | Brc1cc2c(OC(CC23OCC(=O)N(C3)Cc2cc(OC)ccc2)(C)C)cc1 | 1 |
| CHEMBL517873 | O1c2c(cccc2)C2(OCC[NH+](C2)Cc2ccc(N)cc2)CC1(C)C | 1 |
| CHEMBL518231 | O1c2c(cccc2)C2(OCC(=O)N(C2)Cc2ccccc2)CC1(C)C | 1 |
| CHEMBL518779 | FC(F)(F)c1ccc(cc1)C[NH+]1CC2(OCC1)CC(Oc1c2cccc1)(C)C | 1 |
| CHEMBL519319 | S(=O)(=O)(N(S(=O)(=O)C)C1=Nc2c(N3C1=NN(c1ccccc1)C3=O)cccc2)C | 1 |
| CHEMBL519718 | S1C[C@H]([NH2+]C[C@H](OCC)CSc2ccccc2OC)COc2c1cccc2 | 1 |
| CHEMBL523584 | OCC(NC(=O)CCCC\C=C\C\C=C\C\C=C\C\C=C\CCCC)C(=O)[O-] | 1 |
| CHEMBL523763 | O1C(C(O)COC(=O)CCCCCNC(=O)CCCCCCC\C=C/C\C=C/CCCCC)C([O-])=C(O)C1=O | 1 |
| CHEMBL524296 | O(C(=O)C(NC(=O)C\C=C\C\C=C\C\C=C\CCCCCCCC)CO)CC | 1 |
| CHEMBL525722 | P(OC)(OC)(=O)C(NC(=O)CCCCCCCCCCCCCCCCC)CO | 1 |
| CHEMBL538387 | O(Cc1ccccc1)c1ccc(cc1OC)\C=[N+](/[O-])\C(C)(C)C | 1 |
| CHEMBL549946 | S1C[C@H]([NH2+]C[C@@H](CSc2c3OCOc3ccc2)C)COc2c1cccc2 | 1 |
| CHEMBL551636 | S1C[C@H]([NH2+]C[C@@H](CSc2cccc(C)c2O)C)COc2c1cccc2 | 1 |
| CHEMBL551637 | S1C[C@H]([NH2+]C[C@@H](CSc2cccc(C)c2CO)C)COc2c1cccc2 | 1 |
| CHEMBL551698 | S1C[C@H]([NH2+]C[C@@H](CSc2cccc(OC)c2O)C)COc2c1cccc2 | 1 |
| CHEMBL552036 | S1C[C@H]([NH2+]C[C@@H](CSc2cccc(OC)c2N)C)COc2c1cccc2 | 1 |
| CHEMBL556069 | Oc1ccc(cc1)C=1NC(=C2N(C=1)C(=O)C(=N2)C)c1ccc(N(C)C)cc1 | 1 |
| CHEMBL558650 | Oc1ccc(cc1)-c1nc(-c2ccc(N(C)C)cc2)c(nc1)N | 1 |
| CHEMBL560159 | S1C[C@H]([NH2+]C[C@@H](CSc2cccc(F)c2N)C)COc2c1cccc2 | 1 |
| CHEMBL560728 | Oc1ccc(cc1)C=1NC(=CN2C=1N=C(C)C2=O)c1ccc(O)cc1 | 1 |
| CHEMBL561222 | S1C[C@H]([NH2+]C[C@@H](CSc2cccc(C)c2N)C)COc2c1cccc2 | 1 |
| CHEMBL561419 | S1C[C@H]([NH2+]C[C@@H](CSc2c3OCCc3ccc2)C)COc2c1cccc2 | 1 |
| CHEMBL561422 | S1C[C@H]([NH2+]C[C@@H](CSc2c3[nH]ncc3ccc2)C)COc2c1cccc2 | 1 |
| CHEMBL561992 | Fc1ccc(cc1)C=1NC(=CN2C=1N=C(C)C2=O)c1ccc(O)cc1 | 1 |
| CHEMBL562237 | S1C[C@H]([NH2+]C[C@@H](CSc2cccc(C(=O)N)c2O)C)COc2c1cccc2 | 1 |
| CHEMBL562299 | S1C[C@H]([NH2+]C[C@@H](CSc2c3occc3ccc2)C)COc2c1cccc2 | 1 |
| CHEMBL563959 | S1C[C@H]([NH2+]C[C@@H](CSc2c3ocnc3ccc2)C)COc2c1cccc2 | 1 |
| CHEMBL564179 | O(C)c1ccc(cc1)C=1NC(=CN2C=1N=C(C)C2=O)c1ccc(O)cc1 | 1 |
| CHEMBL583501 | Brc1cc2c(OC(CC23OC(=O)N(Cc2ccc(NS(=O)(=O)C)cc2)C3=N)(C)C)cc1 | 1 |
| CHEMBL583962 | O1C2(CC(Oc3c2cccc3)(C)C)CN(Cc2ccc(NC(=O)C)cc2)C1=O | 1 |
| CHEMBL61872 | O1c2c(OC1)cc1c(c2)C(=NN(C(=O)C)[C@@H](C1)C)c1ccc(N)cc1 | 1 |
| CHEMBL633 | Ic1cc(cc(I)c1OCC[NH+](CC)CC)C(=O)c1c2c(oc1CCCC)cccc2 | 1 |
| CHEMBL652 | FC(F)(F)COc1ccc(OCC(F)(F)F)cc1C(=O)NCC1[NH2+]CCCC1 | 1 |
| CHEMBL68738 | O1[C@H](CO)[C@@H](O)[C@@H](O)[C@@H]1n1c2ncnc(NC3CCCC3)c2nc1 | 1 |
| CHEMBL91902 | Oc1c(cc(cc1C(C)(C)C)\C=C\C(=O)c1ccccc1O)C(C)(C)C | 1 |
| Decoy001 | S(=O)(=O)(N1CC[NH2+]CC1C#N)c1cc(n(c1)C)C(=O)N | 0 |
| Decoy002 | S=C(N(Cc1cc(OC)c(OC)cc1OC)Cc1occc1)NCCCOC(C)C | 0 |
| Decoy003 | S(=O)(=O)(CCS(=O)([O-])=Nc1ccc(cc1C(=O)[O-])C)C | 0 |
| Decoy004 | Brc1cc(sc1)[C@@H](Oc1cc(C)c(cc1)C)[C@H]([NH3+])C | 0 |
| Decoy005 | Clc1cc(ccc1)\C=N\NC(=O)c1nnn(c1CNc1ccccc1C(=O)N)-c1nonc1N | 0 |
| Decoy006 | O=C1N(C[C@H](C1)c1[nH+]c2c(n1C[C@@H](CC)C)cccc2)[C@@H](C)c1ccccc1 | 0 |
| Decoy007 | O(C(=O)c1ccccc1C(OCCC[C@@H](CCCC)C)=O)CCC[C@H](CCCC)C | 0 |
| Decoy008 | Br[C@H]1C[C@@H](Oc2c(Cl)cc([N+](=O)[O-])cc2Cl)[C@@]1(CC)C | 0 |
| Decoy009 | s1c2cc(F)ccc2nc1N1[C@H](C(C(=O)c2oc(cc2)C)=C([O-])C1=O)c1occc1 | 0 |
| Decoy010 | S(=O)(=O)(C(C[NH+]1CCC[C@@H](C)[C@H]1c1ccc(cc1)C)(C)C)C | 0 |
| Decoy011 | Ic1cc2nc(n(c2cc1)C[C@@H]1CCC[NH+](C1)C)CCCl | 0 |
| Decoy012 | Brc1cc(Cl)c(cc1)-c1nnc(SCC(=O)[O-])n1N | 0 |
| Decoy013 | s1c2c(CCCC2)c(C(=O)N)c1NC(=O)CSc1[nH+]cc(cc1)C(F)(F)F | 0 |
| Decoy014 | O1C[C@]2(CC[NH+](C2)CC(C)C)c2nc(ncc2C1)-c1ccccc1 | 0 |
| Decoy015 | s1c2c(c3c1[nH+]c(SC\C=C/c1ccccc1)nc3N)C[C@](OC2)(CC)C | 0 |
| Decoy016 | S(=O)(=O)(N1C[C@@H](C[C@H](C1)C)C)c1ccc(cc1)C(=O)Nc1ncnc2c1CCC2 | 0 |
| Decoy017 | Brc1c(n(nc1CC)C)C[NH+]([C@@H](C(C)(C)C)C)C | 0 |
| Decoy018 | S(=O)(=O)(NCCCCCCNS(=O)(=O)c1ccc(OCCC)cc1)c1ccc(OCCC)cc1 | 0 |
| Decoy019 | [NH+]1(CCCC[C@H]1CN1CC[NH+](CC1)CC[C@H]1C[C@H](CCC1)C)C | 0 |
| Decoy020 | s1cc(nc1-c1ccc(cc1)C(F)(F)F)COc1ccc([nH+]c1)C | 0 |
| Decoy021 | Brc1ccc(nc1)N1C2=C([C@@H](C(C#N)=C1n1cccc1)c1sccc1C)C(=O)CCC2 | 0 |
| Decoy022 | Ic1cc(-n2c(C)c(cc2C)\C=C/2\Sc3n(c4c(n3)cccc4)C\2=O)ccc1 | 0 |
| Decoy023 | Clc1cc(ccc1)C(=O)N([C@H](\C=C\C)C(=O)NC(C)(C)C)c1cc(Cl)ccc1 | 0 |
| Decoy024 | S(CC(=O)c1ccc(F)cc1)C1=Nc2c(ccc(c2)C(=O)NCCC[NH+]2CCOCC2)C(=O)N1C[C@@H]1OCCC1 | 0 |
| Decoy025 | O(C(=O)CCC(=O)[O-])[C@H]1CC[C@]2([C@@H](CC[C@@]3([C@H]2CC[C@@H]2[C@@H]4[C@@](CC[C@@]23C)(CC[C@H]4C(C)=C)COC(=O)C)C)C1(C)C)C | 0 |
| Decoy026 | s1cc(nc1\N=C(/[O-])\c1c2c(nc(c1)C(C)C)n(nc2C)C(C)(C)C)C(=O)C | 0 |
| Decoy027 | O=C(N[C@@H]1CCc2[nH]ncc2C1)c1cn[nH]c1C1CCCCC1 | 0 |
| Decoy028 | Brc1ccsc1[C@@H](Nc1cc(OCC[NH+](C)C)ccc1)C | 0 |
| Decoy029 | Ic1cc(O[C@H]([C@H]([NH3+])CC)c2sccc2C)ccc1 | 0 |
| Decoy030 | s1cccc1[C@H]1[NH+](CCc2sccc12)Cc1nc(oc1)-c1ccc(F)cc1 | 0 |
| Decoy031 | Fc1ccccc1[C@H]1C[C@H]1N\C(=[NH+]\C)\N[C@@H]1C[C@@H](CCC1)C(F)(F)F | 0 |
| Decoy032 | S1(=O)(=O)C[C@@H](N2[C@@H](c3c(n[nH]c3-c3cc(C)c(cc3[O-])C)C2=O)c2ccc(OCC=C)cc2)CC1 | 0 |
| Decoy033 | Clc1ccc(N2C(=O)[C@H]3[C@H](C4c5c(cccc5)C3(c3c4cccc3)C(=O)C)C2=O)cc1C(=O)[O-] | 0 |
| Decoy034 | Brc1cc(ccc1N(C)C)C1=NC(=O)C2=C3[C@H](SC2=N1)C[C@@H](CC3)C | 0 |
| Decoy035 | S(CCCC)c1nc2n(n1)[C@@H](C(C(OC(C)C)=O)=C(N2)C)c1cc(OC)c(OCCCCCC)cc1 | 0 |
| Decoy036 | S([C@@H](c1ccccc1)c1[nH+]c2c(n1C[C@@H]1OCCC1)cccc2)c1ccccc1 | 0 |
| Decoy037 | O=C(NCC(=O)N([C@@H](CC)C(=O)[O-])C)c1ccc(cc1)C | 0 |
| Decoy038 | s1ccc(C#N)c1N1CC[C@@H]([NH+]2C[C@@H](S[C@H](C2)C)C)C1=O | 0 |
| Decoy039 | Brc1cc(ccc1[C@H](N[NH3+])Cc1scc(n1)C(C)(C)C)C | 0 |
| Decoy040 | Brc1sc(cc1S(=O)(=O)NC1(CCCC1)C)C[NH2+]CCC | 0 |
| Decoy041 | Fc1c(cccc1F)[C@H]1[C@H]2[NH+]3CCC([C@H]2[NH+](C1)C\C=C\c1occc1)CC3 | 0 |
| Decoy042 | Brc1cc2nc(n(c2nc1)-c1cc(Cl)c(cc1)C)CCCl | 0 |
| Decoy043 | Ic1ccc(nc1)N1CCC[NH2+]CC1 | 0 |
| Decoy044 | s1cccc1-c1oc(nc1)C[NH+]1C[C@H](OCC1)c1ccccc1F | 0 |
| Decoy045 | Brc1cc(ccc1)[C@@H](Sc1ncc(Br)cc1)[C@@H]([NH3+])C | 0 |
| Decoy046 | O=C1NC([O-])=NC(=C1)CC(=O)NC[C@@H](CC(C)C)C(=O)[O-] | 0 |
| Decoy047 | O(C(=O)c1ccccc1C(OCCC[C@@H](CCCC)C)=O)CCC[C@H](CCCC)C | 0 |
| Decoy048 | s1cc(c2c1N=CN(CC(=O)N\N=C/1\c3c(N(C)C\1=O)cccc3)C2=O)-c1ccc(cc1)C | 0 |
| Decoy049 | s1cc(c2c1N=C(SCC(=O)Nc1cc(cc(c1)C(OC)=O)C(OC)=O)N(CC=C)C2=O)-c1ccccc1 | 0 |
| Decoy050 | S(CC1=Nc2c(cccc2)C(=O)N1C)C=1n2c3c(nc2NN=1)cccc3 | 0 |
| Decoy051 | S(=O)([O-])(=Nc1ccc(cc1)C#CC[NH3+])CCCOC | 0 |
| Decoy052 | Brc1cc(F)c(cc1)C[NH+]1C[C@@H](NCC[C@H]1C)[C@H](CC)C | 0 |
| Decoy053 | FC(F)(F)C1([NH+]=C(N(CCC)C(=N1)c1ccccc1)C1CCCCC1)C(F)(F)F | 0 |
| Decoy054 | s1c(C)c(C)c([C@H]([NH+]2CCCCC2)c2ccccc2)c1NC(=O)c1occc1 | 0 |
| Decoy055 | S(=O)([O-])(N(CCC#N)C)=NCc1cc(ccc1)Cn1nccc1 | 0 |
| Decoy056 | S(=O)([O-])(=Nc1ccc(cc1)C(=O)N1CCC2(NC(=O)N=C2[O-])CC1)c1cc(C)c(cc1)C | 0 |
| Decoy057 | Brc1cc(F)c(N)cc1C(=O)N(C)[C@@H]1CCS(=O)(=O)C1 | 0 |
| Decoy058 | o1cccc1C(=O)N[C@@H](C(=O)N([C@@H](CC)C)CC(=O)[O-])C | 0 |
| Decoy059 | Ic1cc(Cl)c(N[C@H](C)c2ccc(cc2C)C)cc1 | 0 |
| Decoy060 | o1c2c(nc1-c1ccc(cc1)C(C)C)cc(NC(=O)c1cccnc1)cc2 | 0 |
| Decoy061 | O=C(Nc1ncccc1C(=O)N[C@@H]1CCCc2nc(ncc12)-c1cccnc1)c1cccnc1N | 0 |
| Decoy062 | O=C(NC[C@@H]1[C@@H]2C[C@H](CC1)C2(C)C)C[NH+]1CCCN(CC1)C | 0 |
| Decoy063 | Clc1ccccc1C1=NC(N=C2N1C=CC(=C2)C)(C(F)(F)F)C(F)(F)F | 0 |
| Decoy064 | Brc1ccc(cc1N)-c1nnnn1[C@H]1C[C@@H](CCC1)C | 0 |
| Decoy065 | S([C@@H](C(=O)NCC(C)C)C)c1nc(nc2N(C3CC3)C([O-])=NC(=O)c12)C1CC1 | 0 |
| Decoy066 | Brc1sc(cc1)[C@H](C(=O)N1C[C@H](OCC1)CCC)C | 0 |
| Decoy067 | s1c(ccc1S(=O)(=O)N1Cc2c3[C@H]([NH+](CCc3ccc2)C)C1)C | 0 |
| Decoy068 | Oc1c(O)c(c2c(cc(C)c(-c3c(cc4c(c(\C=[NH+]\Cc5cccnc5)c(O)c(O)c4C(C)C)c3O)C)c2O)c1C(C)C)\C=N\Cc1cccnc1 | 0 |
| Decoy069 | S1[C@H]2[C@H](CCCC2)C(C(OCC)=O)=C1NC(=O)c1c2[nH+]c3c(nc2n(c1N)-c1ccc(OCC)cc1)cccc3 | 0 |
| Decoy070 | Clc1cccc(Cl)c1-c1c(nn(C)c1N)[C@H](C(C)C)C | 0 |
| Decoy071 | O(CCCn1c2c(nc1[C@H](Oc1cc(ccc1)C)CC)cccc2)c1cc(ccc1C)C | 0 |
| Decoy072 | s1cc(nc1C)CN(C(=O)NC(CC)(CC)C(=O)[O-])C | 0 |
| Decoy073 | S(CC(=O)Nc1ccccc1C#N)c1nc([nH]n1)N\N=C\c1cc([N+](=O)[O-])ccc1[O-] | 0 |
| Decoy074 | S(CC(=O)NCC(=O)[O-])c1nc2c(C[C@@H](CC2)CC)c(C(F)(F)F)c1C#N | 0 |
| Decoy075 | O=C1NCC(CC2=N[C@@H]([NH2+][C@H]12)c1cc(ccc1)-c1nnn[n-]1)(C)C | 0 |
| Decoy076 | O=C(NCCC=1CCCCC=1)C[NH2+]C(CC)(CC)C(=O)[O-] | 0 |
| Decoy077 | BrC(CC)(CC)C(=O)NC(=O)N[C@@H]([O-])C(Cl)(Cl)Cl | 0 |
| Decoy078 | S(CC(=O)NCc1ccc(F)cc1)c1nnc(n1N)N\N=C(/C)\c1ccc(OC)cc1 | 0 |
| Decoy079 | Brc1oc(cc1)-c1onc(N)c1-c1ccccc1C | 0 |
| Decoy080 | Clc1ccccc1O[C@H](C)c1nnc(SCC(=O)N)n1CC=C | 0 |
| Decoy081 | Brc1cnn(CCC)c1[C@@H](N[NH3+])Cc1ccccc1Cl | 0 |
| Decoy082 | S(Cc1ccccc1C(=O)NCCOC)c1nnc(n1CC=C)Cc1ccccc1 | 0 |
| Decoy083 | S(=O)(=O)(Nc1cc2c([nH]nc2)cc1)c1cc2NC(Oc2cc1)=O | 0 |
| Decoy084 | S=C(NCc1ccccc1)[N-]\N=C/c1cc(OCc2ccccc2F)ccc1 | 0 |
| Decoy085 | Oc1cc(cc(O)c1)CCCCCCCCCCCCCCCCCCCC | 0 |
| Decoy086 | Brc1cc(sc1)C[C@H]([NH2+]N)C1(CCCC1)CC(C)C | 0 |
| Decoy087 | S1C(S(=O)(=O)Nc2cc(N)c(F)cc2)=C(NC1=O)C | 0 |
| Decoy088 | Brc1ccc(S(=O)(=O)Cc2oc(cn2)-c2ccccc2)cc1 | 0 |
| Decoy089 | S(=O)([O-])(=Nc1ccc(N2CCCCC2)cc1)CC(=O)NC[C@@](O)(C)c1occc1 | 0 |
| Decoy090 | O[C@H]1C[C@@H](O)[C@@H](\C=C/[C@@H](O)CCCCC)[C@H]1C\C=C\CCCCOC | 0 |
| Decoy091 | S(CC)C1=NC(=O)C2=C(NC(=O)C[C@H]2c2ccc(cc2C)C)N1 | 0 |
| Decoy092 | S(=O)([O-])(=Nc1cc(cnc1N1C[C@@H](CCC1)C(OCC)=O)C(=O)[O-])c1ccc(cc1C)C | 0 |
| Decoy093 | S(C[C@@H](Cn1c2c(nc1N1C[C@H](C[C@H](C1)C)C)N(C)C(=O)NC2=O)C)c1nc(cc(n1)C)C | 0 |
| Decoy094 | FC(F)(F)c1ccc(cc1)-c1[nH+]c2n(C=CC=C2)c1NC(CC(C)(C)C)(C)C | 0 |
| Decoy095 | O=C([C@H]1[NH+](Cc2ccccc2)[C@@H]1C(=O)c1ccccc1)c1ccccc1 | 0 |
| Decoy096 | S(CC(=O)Nc1cc(cc(c1)C(F)(F)F)C(F)(F)F)c1nnc(n1N)-c1n[nH]c(c1)-c1ccc(cc1)C | 0 |
| Decoy097 | S(=O)(=O)(Nc1ccc(NC(=O)NC)cc1)c1cc2CCC(=O)Nc2cc1 | 0 |
| Decoy098 | S=C(NCCC[NH+]1CC[NH+](CC1)CCCNC(=S)NCC=C)NCC=C | 0 |
| Decoy099 | Brc1cc(ccc1)C(=O)N(Cc1occc1)c1sc2c(n1)c(F)cc(F)c2 | 0 |
| Decoy100 | S(Cc1ccccc1C(=O)N[C@@H](CC)C)c1nnc(n1CCOC)-c1ccncc1 | 0 |
| Decoy101 | Brc1cc(sc1)[C@H](N(Cc1ccccc1C)C)[C@H]([NH3+])CC | 0 |
| Decoy102 | s1ccnc1-c1ccc(NC(=O)N[C@@H]2CCCC[C@@H]2C)cc1 | 0 |
| Decoy103 | Clc1cc(-n2nc(cc2N)-c2occc2)ccc1Cl | 0 |
| Decoy104 | Brc1ccccc1OCc1nc(Cl)c2c(n1)n(nc2)C | 0 |
| Decoy105 | s1nnc([C@H]2CCCN(C2)C(=O)CCn2nc(C)c(C(=O)C)c2C)c1-c1onc(c1)C | 0 |
| Decoy106 | Ic1ccccc1C[NH2+][C@@](CC)(CO)c1ccccc1 | 0 |
| Decoy107 | S(=O)(=O)(CNC(=O)[C@@H](N(CCCCCC)C(=O)[C@@H](CCCC)CC)C(CC)CC)c1ccc(cc1)C | 0 |
| Decoy108 | Clc1sc(S(=O)([O-])=NCCCC(OCC)=O)cn1 | 0 |
| Decoy109 | O=C(N1Cc2c([nH]nc2C(c2ccccc2)c2ccccc2)CC1)CCCCC[NH3+] | 0 |
| Decoy110 | S(CC(OC)=O)C1=NC=2N([N-]1)[C@H]([C@H]1C(N=2)=CCCC1=O)c1cc(F)cc(F)c1 | 0 |
| Decoy111 | s1c2CCCc2c2c1nc(nc2SC(=S)N(CC)CC)C | 0 |
| Decoy112 | Clc1c(Cl)cc(cc1Cl)C=1Sc2n(N=1)c(nn2)CC | 0 |
| Decoy113 | Clc1cccc(N)c1NS(=O)(=O)c1ccc(cc1)C | 0 |
| Decoy114 | S([C@@H](C(=O)NCc1occc1)C)C1=Nc2c(cccc2)C(=O)N1CCCOC(C)C | 0 |
| Decoy115 | Clc1ccccc1C(=O)N\C(=C/c1sccc1)\C(=O)N\N=C/c1ccccc1F | 0 |
| Decoy116 | O1[C@H](O[C@H]2[C@H](O)[C@@H](O[C@H]3OC[C@@](O)(C)[C@H]([NH2+]C)[C@@H]3O)[C@H](NC(=O)[C@@H](O)CC[NH3+])C[C@@H]2[NH3+])[C@H]([NH3+])CC=C1C[NH2+]CCO | 0 |
| Decoy117 | [S-]C1=Nc2c(ccc(c2)C(=O)N2CCC([NH+]3CCCCC3)(CC2)C(=O)N)C(=O)N1Cc1occc1 | 0 |
| Decoy118 | Clc1ccc(cc1S(=O)(=O)\N=C(/[O-])\NCCC)C(=O)N[C@H](C)c1ccccc1 | 0 |
| Decoy119 | S(Cc1n[nH]c2OC([NH3+])=C(C#N)[C@H](c12)[C@H]1CCCC=C1)c1ccc(cc1)C | 0 |
| Decoy120 | Clc1cnc(Cl)nc1N(Cc1sc(Cl)cc1)C | 0 |
| Decoy121 | O(CCCC)CC[NH+](CCCC)CCOCCOCC[NH+](CCCC)CCOCCCC | 0 |
| Decoy122 | S(=O)(=O)(Nc1ccc(cc1)C(=O)Nc1ccc(N(C)C)cc1)c1cc2NC(=O)Nc2cc1 | 0 |
| Decoy123 | Fc1ccccc1-c1n[nH]cc1C(=O)Nc1cc2CCC(=O)Nc2cc1 | 0 |
| Decoy124 | s1c2c(nc1N(CC(=O)N1[C@@H](CCC[C@H]1C)C)c1ccc(F)cc1)cccc2 | 0 |
| Decoy125 | s1c(ccc1S(=O)(=O)NCc1ccc(NC(=O)N)cc1)CC | 0 |
| Decoy126 | O=C/1C[C@H]2[C@@H](\C\1=C(/NCC\N=C(/C)\C=1[C@@H]3[C@H](CC=1[O-])C3(C)C)\C)C2(C)C | 0 |
| Decoy127 | O=C(N[C@@H](C)c1ccc(cc1)C#N)c1n[nH]c2c1cc(cc2)C | 0 |
| Decoy128 | S(=O)([O-])(=Nc1cnc(nc1N(C)C)N1CCCC1)c1cc(ccc1)C(F)(F)F | 0 |
| Decoy129 | Clc1cccc(F)c1CNn1c(nnc1[S-])CCC | 0 |
| Decoy130 | Clc1c(cccc1Cl)C[C@H]1S/C(/N(C1=O)c1ccccc1)=C(/C(/[O-])=N/c1ccc(Cl)cc1Cl)\C#N | 0 |
| Decoy131 | s1cccc1[C@@H]1Oc2nc(SCc3ccc(cc3)C)nnc2-c2c(N1)cccc2 | 0 |
| Decoy132 | Clc1ccccc1\C=N/NC(=O)[C@H](NC(=O)c1ccccc1)C1=NNC(=O)c2c1cccc2 | 0 |
| Decoy133 | S1CCC2(OCC[C@H](C2)[C@@H](O)c2nn(cc2)C)CC1 | 0 |
| Decoy134 | O=C(NC[C@H](O)c1ccccc1)c1[nH+]c(c2[nH]c3c(c2c1)cccc3)-c1ccccc1 | 0 |
| Decoy135 | Clc1cc(N\C=C(\C#N)/c2ccc(Cl)cc2)c(O)cc1 | 0 |
| Decoy136 | s1cccc1S(=O)(=O)N[C@H](C(=O)NCCC[NH+]1CCN(CC1)c1ccccc1)C | 0 |
| Decoy137 | Brc1ccc(cc1)C(=O)N1CCN(CC1)c1nnc(cc1)-c1ccc(cc1)C | 0 |
| Decoy138 | Brc1ccc(cc1)-c1nc([nH]c1)C[NH+]1CCC(CC1)(CC)CO | 0 |
| Decoy139 | S(=O)(=O)(N)c1ccc(OCCOC(=O)[C@@H]([N-]C2=NS(=O)(=O)c3c2cccc3)C)cc1 | 0 |
| Decoy140 | O(C[C@@H](OC(=O)C=C)C)C[C@H](OC[C@H](OC(=O)C=C)C)C | 0 |
| Decoy141 | S(=O)(=O)(N(CCC(=O)[O-])C)N1CC(=O)NC(=O)C1(C)C | 0 |
| Decoy142 | FC(F)(F)Oc1ccc(NC(=O)N[C@@H]2C=C(C[C@@H](O)[C@@H]2O)C(=O)N[C@H](CC(=O)[O-])C(=O)N)cc1 | 0 |
| Decoy143 | BrC[C@H]1SC(=[NH+]C1)N1CCOCC1(C)C | 0 |
| Decoy144 | Brc1cc2cc(oc2cc1)C(=O)N\N=C\c1cc(n(c1C)C1CCCCC1)C | 0 |
| Decoy145 | FC(F)(F)c1cc(ccc1)-c1nn(c(N)c1C#N)-c1nccc(c1)C(F)(F)F | 0 |
| Decoy146 | ClC1=CC(=Cn2c1nnc2[C@]1([NH3+])CCOC1)C(F)(F)F | 0 |
| Decoy147 | Clc1ccc(NC(=O)c2ccccc2NC(=O)C(=O)N\N=C\c2cn(nc2-c2ccc(OCc3ccccc3)cc2)-c2ccccc2)cc1 | 0 |
| Decoy148 | Clc1ccc(N2C([O-])=C([C@@H](N\C=C\C=3CCCCC=3)CC)C(=O)NC2=O)cc1 | 0 |
| Decoy149 | s1c2c(CC[C@H](C2)C(CC)(C)C)c(C#N)c1NC(=O)CN1C=Nc2n(ncc2C1=O)C | 0 |
| Decoy150 | Ic1c(nc(nc1Cl)CCCC)COC | 0 |
| Decoy151 | o1nc(nc1CCN1CC[NH+](CC1)CCC[NH+](C)C)-c1ccc(cc1)C(C)C | 0 |
| Decoy152 | Fc1cc(ccc1C[NH+]1CC[C@H](OCCC)CCC1)-c1ccncc1 | 0 |
| Decoy153 | BrCCN(S(=O)(=O)N1CCCCCC1)CC | 0 |
| Decoy154 | Clc1cc(N=S(=O)([O-])c2cc(N)ccc2CC)cc(Cl)c1 | 0 |
| Decoy155 | O(C1CC[NH+](CC1)C\C=C\c1ccc(cc1)C#N)c1ccccc1 | 0 |
| Decoy156 | O(C)c1ccc(cc1)C(=O)N([C@H](C(=O)c1c(C)c([nH]c1C)C(OCC)=O)C)CC=C | 0 |
| Decoy157 | Clc1ccc(SCCN(C(=O)C[C@@H]2NC(=O)N=C2[O-])CCO)cc1 | 0 |
| Decoy158 | Clc1ccc(cc1)-c1oc(cc1)\C=N/NC1=N[N-]C(=O)NC1=O | 0 |
| Decoy159 | Brc1ccc(OCc2nc(Cl)c3c(sc(C)c3C)n2)cc1 | 0 |
| Decoy160 | S\1[C@H](CCOC(=O)C)C(=O)N/C/1=N\N=C/c1ccc(OC)c(OC)c1C(=O)[O-] | 0 |
| Decoy161 | s1cc(nc1-c1ccsc1)C(=O)N[C@H]([C@@H](O)C)C(=O)[O-] | 0 |
| Decoy162 | Brc1sc(nc1)NC(=O)c1sc(Cl)nn1 | 0 |
| Decoy163 | O(CC[NH+]([C@H](C[C@@]([NH2+]CCC)(C(OC)=O)C)C)CC)CC | 0 |
| Decoy164 | Clc1cc(S(=O)([O-])=Nc2cc(ccc2)C(C)C)c(N)cc1 | 0 |
| Decoy165 | S(=O)([O-])(=Nc1nc2n(n1)[C@H](CC(=N2)c1ccccc1)c1ccc(F)cc1)c1ccc(cc1)C | 0 |
| Decoy166 | s1c2c(nc1N(C(=O)c1ccc(F)cc1)Cc1[nH+]cccc1)c(ccc2)C | 0 |
| Decoy167 | S1C/C(/N[C@H]1SCC(=O)Nc1ccc(cc1)C)=C/C(=O)NCCC=1C[NH+]=C2C=1C=CC=C2 | 0 |
| Decoy168 | S(CC(=O)c1ccc(cc1C)C)C1=Nc2c(cccc2)C(=O)N1CCCOCC | 0 |
| Decoy169 | s1cc(N)cc1S(=O)([O-])=Nc1cc2c(nc1)cccc2 | 0 |
| Decoy170 | S(OC[C@@](O)(C(=O)c1ccccc1)c1ccccc1)(=O)(=O)CCCCCCCCCCCC | 0 |
| Decoy171 | S([C@H](C)c1oc(nn1)-c1ccc(OC)cc1)c1nnc(n1-c1ccc(cc1)C)N1CCCC1 | 0 |
| Decoy172 | ClCCCC(O[C@H](n1nnc2c1cccc2)c1ccc(cc1)C)=O | 0 |
| Decoy173 | s1c2c(nc1C)CCC[C@H]2[NH+](Cc1cc([N+](=O)[O-])ccc1OC(F)F)C | 0 |
| Decoy174 | Fc1ccc(cc1)[C@@H](NC(=O)/C(/[O-])=N/c1cc(ccc1)C(=O)C)CC(F)(F)F | 0 |
| Decoy175 | S1C[C@@H]2NC(=O)N[C@@H]2[C@@H]1CCCCC(=O)NC[C@@H]1O[C@H](O[C@H]2[C@@H](O)[C@@H](O[C@H]3O[C@@H](CO)[C@@H](O)[C@H]([NH3+])[C@H]3O)[C@H]([NH3+])C[C@H]2[NH3+])[C@H]([NH3+])C[C@@H]1O | 0 |
| Decoy176 | O(C(C)C)c1ccc(cc1)[C@@H](CC[NH2+][C@H](C)c1ccc(OC)cc1)c1ccc(cc1)C | 0 |
| Decoy177 | Brc1cc2n(CC[C@H](C)c3ccccc3)c([nH+]c2cc1)N | 0 |
| Decoy178 | Clc1cccc(N)c1-c1nnnn1[C@@H]1CC[C@H](C[C@H]1C)C | 0 |
| Decoy179 | O1[C@H]2[C@H]3[C@H]4CC[C@@H]5[C@](CC[C@@H]6[C@@]5(CCC6=O)C)(C)[C@@]4(CC[C@@]3(CCC2(C)C)C1)C | 0 |
| Decoy180 | s1c(ccc1C)-c1[nH]nc(c1)C(=O)N1Cc2nc[nH]c2C[C@H]1C(=O)[O-] | 0 |
| Decoy181 | s1cc(cc1)CCNc1nc2c(cc1)c(ccc2)C(=O)[O-] | 0 |
| Decoy182 | S(=O)(=O)(NC[C@@H]1OCCC1)c1cc(ccc1C)-c1nnc(Nc2ccc(OCC(=O)N)cc2)c2c1cccc2 | 0 |
| Decoy183 | O=C1C2=C(Nc3c(N[C@@H]2c2cc(ccc2)C)cccc3)C[C@H](C1)c1ccc(cc1)C | 0 |
| Decoy184 | O(CC[NH+](CCCC)CCCC)C[C@H](CC)C[C@@H](CCCC)CC | 0 |
| Decoy185 | o1cccc1\C=C(\NC(=O)c1ccccc1)/C(=O)NCC(O)CNC(=O)/C(/NC(=O)c1ccccc1)=C\c1occc1 | 0 |
| Decoy186 | S(=O)(=O)(Cc1ccc(cc1)C)c1ncc(n1CCOC)CN(C(C)C)C(=O)CC(C)(C)C | 0 |
| Decoy187 | Brc1cc(C(O[C@@H](C)c2onc(n2)-c2cccnc2)=O)c([O-])cc1 | 0 |
| Decoy188 | O1[C@@H](C[C@@H]2CO[C@@H](C\C(=C\C(OCCCCCCCC)=O)\C)[C@H](O)[C@@H]2O)[C@@H]1[C@H]([C@@H](O)C)C | 0 |
| Decoy189 | o1c(ccc1N1CCCC1)\C=C/1\c2c(nc(N)c(C#N)c2C)C(C#N)=C\1C | 0 |
| Decoy190 | Brc1cc(F)c(cc1)[C@@H]([NH2+]C)c1scc(n1)C(C)(C)C | 0 |
| Decoy191 | Brc1sc(cc1)[C@H]([NH2+]C1CC1)c1sc2CCCCc2n1 | 0 |
| Decoy192 | S(=O)(=O)(N1CCN(CC1)C(OC)=O)c1c[nH]nc1N | 0 |
| Decoy193 | O=C(Nc1ccccc1C[NH2+]C)c1n(ccc1)CC(=O)[O-] | 0 |
| Decoy194 | Brc1ccsc1[C@@H](Nc1cc(ccc1)-c1sccn1)C | 0 |
| Decoy195 | FC(F)Oc1ccccc1-c1nc(on1)C[NH+]1CCC[C@H]1c1ccccc1C | 0 |
| Decoy196 | Clc1ccc(cc1)CCCS(=O)[C@@H](C)c1onc(n1)CCCC | 0 |
| Decoy197 | s1cccc1-c1n(nc2c1[C@@H](N(C2=O)c1sccn1)c1cc(O)ccc1)-c1ccccc1 | 0 |
| Decoy198 | s1c2cc(NC(=S)NC(=O)c3cc([N+](=O)[O-])ccc3N3CCOCC3)ccc2nc1NC(=O)CCC | 0 |
| Decoy199 | Cl[C@@H]([C@@H](C)c1ccncc1)c1cccc(OC)c1OC | 0 |
| Decoy200 | Brc1cc(F)c(cc1)C[C@@H](N[NH3+])[C@@H]1C[C@@H]1c1ccccc1 | 0 |
| Decoy201 | [S-]C=1N([C@H](NC(=O)C=1C#N)c1ccc(cc1)C(C)(C)C)c1ccc(cc1)C(C)C | 0 |
| Decoy202 | S1C[C@@H]2NC(=O)N[C@@H]2[C@@H]1CCCCC(=O)NCCCCCC(=O)NCCCCCNC(=O)[C@@H]([NH3+])CCCNC(=S)N | 0 |
| Decoy203 | Brc1cc(ccc1Cl)[C@H](N[NH3+])Cc1ccc(Br)cc1 | 0 |
| Decoy204 | O(C)c1cc(ccc1)[C@@H]([NH+](C)C)CNC(=O)Nc1ccc(cc1)C(=O)NCC(C)C | 0 |
| Decoy205 | S1\C(=C\c2sc(cc2)C)\C(=O)N(c2ccc(OCC)cc2)C1=S | 0 |
| Decoy206 | s1c(cnc1NN)COc1cc2CCC(=O)Nc2cc1 | 0 |
| Decoy207 | Brc1cc(cc(OC)c1O)\C=N/N1C(=N[N-]C1=S)c1[nH]nc(c1)-c1ccccc1 | 0 |
| Decoy208 | Brc1cc2c(NC(=O)[C@H]2O)cc1N1C[C@H]([NH+](CC1)C)CC | 0 |
| Decoy209 | s1c(-c2nnc([S-])n2C)c(nc1NC(=O)C1CCCCC1)C | 0 |
| Decoy210 | Brc1cc(ccc1)[C@H]1C2=C(N=C(C)C1C(OC)=O)C[C@H](CC2=O)c1occc1 | 0 |
| Decoy211 | O1c2c(OC[C@@H]1C(=O)NN\C=C/1\C([O-])=C(C)C(=O)C=C\1C)cc1c(c2)cccc1 | 0 |
| Decoy212 | BrC=1C=C(c2[nH+]c(C(C)(C)C)c(n2C=1)C(=O)[O-])C | 0 |
| Decoy213 | O1CC(=Cc2c1cccc2)C[NH+](C[C@@H]1CC[NH+](C1)Cc1ccccc1)C | 0 |
| Decoy214 | S1(=O)(=O)C[C@@H](N2C[C@@H]3[NH+](C[C@@H]2C(C)C)CCC3)C=C1 | 0 |
| Decoy215 | FC(F)(F)c1ccc(nc1)/C(=C/c1cc(C)c(OC)c(c1)C)/C#N | 0 |
| Decoy216 | s1nc(C(=O)N)c(N)c1C(=O)N([C@@H](C(=O)NC[C@@H]1OCCC1)c1oc(cc1)C)c1cc(cc(c1)C)C | 0 |
| Decoy217 | O=C(NC12CC3CC(C1)CC(C2)C3)N[C@@H]1CCC[C@H]1C#N | 0 |
| Decoy218 | S(CCCCCCCCCC)c1nc([nH]n1)CCCCCCCC | 0 |
| Decoy219 | s1cc(nc1-c1ncn[n-]1)CC(=O)N(CC(C)C)C[C@@H](O)c1ccc(F)cc1 | 0 |
| Decoy220 | O(CC(=O)N([C@H](\C=C/C)C(=O)NC(CC(C)(C)C)(C)C)CCCCCC)C | 0 |
| Decoy221 | Clc1ccc(cc1)-c1nc(sc1)-c1n(C)c(C)c(S(=O)(=O)N2CCCC[C@H]2C)c1 | 0 |
| Decoy222 | [NH+]1(CCC[C@H]1c1cccnc1-n1c(ccc1C)C)CCCC | 0 |
| Decoy223 | O1c2cc(CC=C)c(cc2OC1)COCOCCOCCOCCCC | 0 |
| Decoy224 | Clc1ncc(S(=O)(=O)N2C[C@@H](Cc3c2cccc3)C)cc1 | 0 |
| Decoy225 | FC(F)(F)c1cc2nc(n(c2cc1)CC[NH+](CC)CC)COc1ccc(cc1C)C | 0 |
| Decoy226 | Brc1oc(cc1)[C@H](O)c1cc2CCC(=O)N(c2cc1)C | 0 |
| Decoy227 | s1c(C(=O)[O-])c(cc1S(=O)(=O)N(CCCC(OC)=O)C)C | 0 |
| Decoy228 | S1\C(=C/c2cc(OC)ccc2)\C(=O)N(C/C(/[O-])=N/c2scc(n2)CC(=O)[O-])C1=O | 0 |
| Decoy229 | S(=O)([O-])(=Nc1ccccc1OCC(=S)N)c1ccccc1 | 0 |
| Decoy230 | [NH+](CC1CC[NH+](CC1)[C@@H]1C[C@H](CC[C@@H]1C#N)CCC)(C)C | 0 |
| Decoy231 | S1C=C(N2C1=NC(=CC2=O)C[NH+](Cc1occc1)CCC)c1ccccc1 | 0 |
| Decoy232 | O(CC)c1cc(O)c(cc1)\C=N\Nc1[nH]c2N=CNC(=O)c2n1 | 0 |
| Decoy233 | S([C@@H](C(=O)N(CCC#N)c1ccccc1)C)c1nnc(n1-c1ccccc1)C[NH+]1CCCCC1 | 0 |
| Decoy234 | s1cccc1C[NH+]1CC[NH+](CC1)[C@@H](C(=O)[O-])c1c2c(n(c1)CC(=O)[O-])cccc2 | 0 |
| Decoy235 | S(=O)(=O)(N1C[C@H]2[NH+](CCc3c2cccc3)[C@@H]1C(C)C)c1ccc(cc1)C | 0 |
| Decoy236 | n1c2n(nc1N)[C@H](C[C@@H](N2)c1ccc(cc1)CC)c1ccc(cc1)CC | 0 |
| Decoy237 | O=C1c2c(N(C)C(=O)C1=C1NNC([O-])=C1\C=N\c1cccnc1)cccc2 | 0 |
| Decoy238 | S(=O)([O-])(=NNC(=O)c1c2c(n(c1)C)cccc2)c1c(C)c(N=S(=O)([O-])C)c(cc1C)C | 0 |
| Decoy239 | Ic1cc2[nH+]c(n(c2cc1)Cc1sc(nc1)C)[C@H](Cl)C | 0 |
| Decoy240 | Clc1ccc(cc1)-c1oc(nn1)CN1C(=O)[C@@]2(NC1=O)CCc1c(C2)cccc1 | 0 |
| Decoy241 | s1c(ncc1C[NH+]1C[C@@H](SCC)CCCC1)-c1ncccc1 | 0 |
| Decoy242 | Fc1ccc(cc1)[C@H]1N(Cc2cccnc2)C(=O)C([O-])=C1C(=O)c1ccc(OCCC)cc1C | 0 |
| Decoy243 | O[C@H]1C[C@@H](O)[C@@H](\C=C/[C@@H](O)CCCCC)[C@H]1C\C=C\CCCCOC | 0 |
| Decoy244 | Ic1ccc(cc1)Cn1cc(cc1)[C@@H]([NH2+]CCC)C(C)C | 0 |
| Decoy245 | Brc1cc(Cl)c(\N=C(/[O-])\C[NH+]([C@H](CC(=O)[O-])C)CC)cc1 | 0 |
| Decoy246 | Clc1ccc(cc1)C(=O)Nc1scc(n1)CC(=O)NCCC=1CCCCC=1 | 0 |
| Decoy247 | Ic1scc(c1)C(=O)N(C)[C@H]1CCC[NH+](C1)C | 0 |
| Decoy248 | s1c2c(nc1COCC(=O)N1CCC(n3nnc4c3C(=O)NN=C4[O-])CC1)cccc2 | 0 |
| Decoy249 | O(CC(=O)NC(=O)NCC(C)C)C(=O)\C=C\C(OCC(=O)NC(=O)NCC(C)C)=O | 0 |
| Decoy250 | Brc1cnc(nc1)Nc1cc(N)ccc1Cl | 0 |
| Decoy251 | Brc1ccc(S[C@@H](C[C@@]([NH2+]CCC)(C(=O)[O-])C)C)nc1 | 0 |
| Decoy252 | S(CCCOc1ccc(F)cc1)c1nc2c(n1CCOC)cccc2 | 0 |
| Decoy253 | S(=O)(=O)(Nc1nc(nc(c1)C)C)c1ccc(NC(=S)NCC)cc1 | 0 |
| Decoy254 | S(=O)(=O)(N)c1ccc(cc1)[C@@H]([NH+](CC(=O)NCc1ccccc1OC(F)(F)F)C)C | 0 |
| Decoy255 | s1c2cc(OC)ccc2nc1N(C(=O)CCSc1ccc(cc1)C)CCC[NH+](C)C | 0 |
| Decoy256 | s1cccc1CSCC(=O)N1CCN(CC1)c1ccc(F)cc1 | 0 |
| Decoy257 | Clc1c(C[NH2+]C2CC2)c(Cl)ccc1S(=O)(=O)N[C@H]1C[C@H]1C | 0 |
| Decoy258 | Clc1sc(Cl)cc1C(=O)C[NH+]1C[C@H](OC[C@H]1CC)C | 0 |
| Decoy259 | Clc1ccc(cc1N[C@@H](c1ccc2c([nH+]c(cc2)C)c1O)c1occc1)C(F)(F)F | 0 |
| Decoy260 | Brc1cc(C)c(SCC(=O)NC(C(=O)[O-])(C)C)cc1 | 0 |
| Decoy261 | Clc1cc(\C=N/NC(=O)Cn2c3c(nc2-c2nonc2N)cccc3)c(O)cc1 | 0 |
| Decoy262 | o1c(nc(C[NH+](C(C)C)C)c1C)-c1ccc(cc1)-c1ccccc1 | 0 |
| Decoy263 | S(Cc1cc(ccc1OC)\C=N\NC(=O)c1nnn(c1C[NH+]1CCCCCC1)-c1nonc1N)c1oc2c(n1)cccc2 | 0 |
| Decoy264 | Clc1cccc(F)c1COc1c(cccc1OC)\C=N\NC(=O)c1nnn(c1CN1CCc2c1cccc2)-c1nonc1N | 0 |
| Decoy265 | S(C/C(/O)=N/CC1(CC1)c1ccccc1)c1[nH]c(C)c(n1)Cc1ccccc1 | 0 |
| Decoy266 | S1\C(=C/c2ccc(OC)cc2)\C(=O)N(CCC(=O)n2c3c(nc2SCC(=O)Nc2ccc(OC)cc2)cccc3)C1=S | 0 |
| Decoy267 | S=C1N=C([O-])[C@@H]2C(OC(N)=C(C#N)[C@@H]2c2ccc([N+](=O)[O-])cc2)=N1 | 0 |
| Decoy268 | O([C@@H](C(=O)C1=C2C(=[NH+][C@@H]1C)C=CC=C2)C)C(=O)C12CC3CC(C1)CC(C2)C3 | 0 |
| Decoy269 | FC(F)Oc1ccc(cc1)C[NH+](Cc1oc(nn1)-c1cc(ccc1)C)C | 0 |
| Decoy270 | s1cc(nc1CNC(=O)C1=C(C)C(=NNC1=O)C)C(=O)[O-] | 0 |
| Decoy271 | Clc1sc(Cl)cc1S(=O)(=O)N(Cc1cc(ccc1)C#N)C | 0 |
| Decoy272 | Ic1cc2[nH+]c(n(c2cc1)-c1ccc(F)cc1)CCCl | 0 |
| Decoy273 | Clc1cc(Cl)c(Cl)cc1OCC(=O)NCC(=O)Nc1cc(F)c(F)cc1 | 0 |
| Decoy274 | O=C(N1CCCC1)C[NH+]1CC[NH+](CC1)[C@@H](C(=O)[O-])c1c2c([nH]c1)cc(NC(=O)CCC(=O)[O-])cc2 | 0 |
| Decoy275 | Brc1cc(sc1)[C@H](Sc1ccc(OC)cc1)[C@@H]([NH3+])C | 0 |
| Decoy276 | S(=O)(=O)(N1CCC(NS(=O)(=O)CC)CC1)c1c[nH]nc1N | 0 |
| Decoy277 | S(C)c1ccccc1NC(=O)CSc1nc([nH]n1)N\N=C(\C)/c1ccccc1 | 0 |
| Decoy278 | Clc1cc(F)ccc1CSC=1N=NC=2[C@@H]([NH+]=C3C=2C=CC=C3)N=1 | 0 |
| Decoy279 | ClCc1sc(S(=O)(=O)N(C)c2ccc(cc2C)C)cc1 | 0 |
| Decoy280 | S(=O)([O-])(=Nc1cn(nc1)CCO)c1cc(ccc1CC)C(=O)[O-] | 0 |
| Decoy281 | Brc1cc(cc(Br)c1O)\C=N/NC(=O)c1nnn(c1C[NH+]1CCCC[C@@H]1C)-c1nonc1N | 0 |
| Decoy282 | O1[C@@]23[C@@H]1C[C@H]1[C@@H]4C[C@@H]5CCCC[C@@]5(C(=O)C)[C@]4(CC[C@H]1[C@]2(CC[C@H](OC(=O)C)C3)C)C | 0 |
| Decoy283 | S=C(N)c1c[nH]nc1NC(=O)c1c2c(oc1)cccc2 | 0 |
| Decoy284 | Cl[C@H](C)c1[nH+]c2cc(ccc2n1-c1ccccc1CC)C | 0 |
| Decoy285 | s1c2CCCc2c2c1nc(nc2[O-])CSc1nc(C)c(cc1C#N)C(=O)C | 0 |
| Decoy286 | Brc1ccc(cc1)-c1[nH+]c2c(n1CCOCC)cccc2 | 0 |
| Decoy287 | Brc1c(nc(nc1Cl)-c1oc(Br)c(Br)c1)C | 0 |
| Decoy288 | S1\C(=C/c2ccc(OCCC)cc2)\C(=O)N(c2ccccc2)C1=S | 0 |
| Decoy289 | S(=O)(=O)(n1nc(N)c(c1)-c1cc(C)c(F)cc1)c1ccc(cc1)C | 0 |
| Decoy290 | S1C=Cn2c1nc(C(=O)N1CCCCCCC1)c2C[NH+]1CCC(=CC1)c1ccccc1 | 0 |
| Decoy291 | O(CC1c2c(-c3c1cccc3)cccc2)C(=O)[C@@H](CCCCCCCCC[NH3+])C(=O)[O-] | 0 |
| Decoy292 | Brc1sc(cc1)[C@H](Oc1cc(ccc1C)C)C[NH3+] | 0 |
| Decoy293 | S=C(Nc1cc(ccc1)C)NCCCNc1nc2c(cc1C#N)cc(OC)cc2 | 0 |
| Decoy294 | Brc1ccccc1\C=C(\S(=O)(=O)c1cc(Cl)ccc1Cl)/C#N | 0 |
| Decoy295 | o1nc(c2nc(nc(NCc3ccc(cc3)C)c12)CC)-c1ccc(cc1)C | 0 |
| Decoy296 | Brc1ccc(cc1)Cc1n2c(nn1)C=C(Cl)N=C2C | 0 |
| Decoy297 | Brc1cc(C)c(NC(=O)C2CCN(CC2)C(=O)c2n(ccc2)-c2cccnc2)cc1 | 0 |
| Decoy298 | S(=O)([O-])(=NCCC(=O)N(C)C)N1CCCC[C@@H]1C(=O)[O-] | 0 |
| Decoy299 | s1c2nc(cc(c2c(N)c1C(=O)Nc1cc(ccc1)C)-c1ccccc1)-c1ccccc1 | 0 |
| Decoy300 | S([C@H]1C[C@]([NH2+]C)(CCC1)C(=O)[O-])c1ncnc2nc[n-]c12 | 0 |
| Decoy301 | OCC1CCN(CC1)C(=O)NC12CC3CC(C1)CC(C2)C3 | 0 |
| Decoy302 | Brc1ccsc1C[NH2+][C@@H](C)c1ccc(OC(F)(F)F)cc1 | 0 |
| Decoy303 | Clc1ccc(Nc2ncnc(Oc3cc(cc(c3)C)C)c2N)cc1C(F)(F)F | 0 |
| Decoy304 | OCC[C@H]1N(CC[NH+](C1)CCC=1C(CCCC=1C)(C)C)C1CC[NH+](CC1)C(C)C | 0 |
| Decoy305 | S([C@H](C(=O)C)C)c1nnc(n1-c1ccc(cc1)C)-c1ccccc1 | 0 |
| Decoy306 | s1cccc1[C@H](NC(=O)NC[C@H]([NH+]1CCc2sccc2C1)C)CC | 0 |
| Decoy307 | Ic1c([nH+]c(nc1NCCC)N1CCCCC1)CC | 0 |
| Decoy308 | S1CCCN=C1N(CC(=O)c1ccc(cc1)C)c1ccccc1C | 0 |
| Decoy309 | Clc1ccccc1NS(=O)(=O)c1ccc(N2S(=O)(=O)CCC2=O)cc1C | 0 |
| Decoy310 | S1\C(=C/c2cc3cc(ccc3nc2N2CCCCC2)C)\C(=O)N(CC)C1=S | 0 |
| Decoy311 | BrC12[C@H]3[C@H](C(c4c1cccc4)c1c2cccc1)C(=O)N(c1cc(Cl)c(cc1)C)C3=O | 0 |
| Decoy312 | Brc1cc(n(c1)C)C(=O)Nc1cc(ccc1)-c1[nH]ncc1 | 0 |
| Decoy313 | Ic1ccc(N[C@@H](C)c2nnc(SCC(=O)N\N=C\c3cc(Br)cc([N+](=O)[O-])c3O)n2C)cc1 | 0 |
| Decoy314 | S1CC(O)=Nc2n(nc(O)c2[C@H]1c1cccnc1)C1CCCCCC1 | 0 |
| Decoy315 | FC(F)(F)c1nn(CCCNC(=O)[C@H]2[C@H]3CC[C@H](C=C3)[C@@H]2C(=O)[O-])c(c1)C1CC1 | 0 |
| Decoy316 | Clc1c(C[NH3+])c(Cl)ccc1S(=O)(=O)N[C@H](CCCCC)C | 0 |
| Decoy317 | O=C(N([C@H](CCCCC)C(=O)NC(CC(C)(C)C)(C)C)CCCCCC)Cc1ccccc1C | 0 |
| Decoy318 | S1\C(=C/c2cc(OCC(=O)N)ccc2)\C(=NC1=O)[N-]c1cc(F)c(F)c(F)c1 | 0 |
| Decoy319 | S(=O)(=O)(N(CC)CC)c1ccc(nc1)NN=S(=O)([O-])c1cc([N+](=O)[O-])ccc1OC | 0 |
| Decoy320 | S(CC(=O)NN1C(=O)C2(NC1=O)CCCCC2)c1nc(cc(n1)N)C | 0 |
| Decoy321 | BrCC1(CCCCC1)CSc1[nH]c2cc(ccc2n1)C | 0 |
| Decoy322 | O=C1N=C(NC2(CCCCC2)[C@@H]1C#N)N1CC[NH+](CC1)CC | 0 |
| Decoy323 | FC(F)(F)c1cc(ccc1)-c1oc(cc1)C(=O)N(C(C)C)C(C)C | 0 |
| Decoy324 | O1[C@H](CO)[C@@H](O)[C@H](O)[C@@H](O)[C@@H]1Oc1c(CCCCCCCCC)c(O[C@@H]2O[C@H](CO)[C@@H](O)[C@H](O)[C@H]2O)c(O)cc1O | 0 |
| Decoy325 | s1cccc1-c1nn2c(N=C(C=C2[O-])c2n[nH]c3c2CCC3)c1C | 0 |
| Decoy326 | s1c2nc(nc([O-])c2cc1C(C)C)CSCC(=O)NCc1ccncc1 | 0 |
| Decoy327 | s1cccc1[C@@H]1[C@@H]2C(=CC[C@@H](C2)C)C(C#N)=C(N)C1(C#N)C#N | 0 |
| Decoy328 | Brc1cc(CCl)c(F)c(S(=O)(=O)N(C)C2CCCC2)c1 | 0 |
| Decoy329 | FC(F)(F)[C@H](NC(=O)C1=CN(C=C(C(=O)NCC(C)C)C1=O)C(C)C)c1ncccc1 | 0 |
| Decoy330 | Brc1ccc(cc1Cl)[C@H](N[NH3+])C[C@@H](CCCC)CC | 0 |
| Decoy331 | S(=O)(=O)(N[C@@H](CCCO)C)c1cc(n(c1)C(C)C)C(=O)[O-] | 0 |
| Decoy332 | s1cccc1[C@@](O)(CNC(=O)/C(/[O-])=N/c1ccc(cc1)C(F)(F)F)C1CC1 | 0 |
| Decoy333 | Brc1sc(cc1)[C@H]([NH2+]CCC)Cc1cccnc1N | 0 |
| Decoy334 | Brc1cc(CN2C=Nc3c(cc(Cl)cc3Cl)C2=O)c(F)cc1 | 0 |
| Decoy335 | Brc1cc(C#N)c(\N=C(/[O-])\c2ccnc(N)c2F)cc1 | 0 |
| Decoy336 | O(C)c1ccc(cc1)[C@H](\[NH+]=C(/C)\C1[C@H]2[C@@H](CC1=O)C2(C)C)C1CC1 | 0 |
| Decoy337 | S(O[C@@H]1[C@H](O)C=C(O[C@@H]1O[C@@H]([C@H](NC(=O)C)C=O)[C@H](O)[C@H](O)CO)C(=O)[O-])(=O)(=O)[O-] | 0 |
| Decoy338 | BrC=1C(=O)N(N=CC=1Br)CCCc1ccccc1 | 0 |
| Decoy339 | [NH+]1(CCCC1)[C@@]([C@@H](NN)c1c(ccnc1N)C)(CC)C | 0 |
| Decoy340 | S(=O)([O-])(=N[C@]1(CCS(=O)(=O)C1)C)N(CCC(=O)[O-])C | 0 |
| Decoy341 | Clc1ccccc1N1C(=O)[C@@](N=C1[O-])(NS(=O)(=O)c1ccc(N)cc1)C(F)(F)F | 0 |
| Decoy342 | Ic1cc2[nH+]c(n(c2cc1)[C@H](CCC)COC)[C@@H](Cl)C | 0 |
| Decoy343 | Clc1cc(CNC[C@@H]([NH+](C)C)c2ccc(F)cc2)c(OCc2cccnc2)cc1 | 0 |
| Decoy344 | O(C(COCCCOC(=O)C=C)COCCCOC(=O)C=C)CCCOC(=O)C=C | 0 |
| Decoy345 | Clc1cccc(Cl)c1Cn1c2c([nH+]c1[C@H](NC(=O)CC)C)cccc2 | 0 |
| Decoy346 | Fc1c(cccc1F)C[NH2+]C[C@]1(O)CCCN(CCc2ccccc2)C1=O | 0 |
| Decoy347 | S(=O)(=O)(N)c1cc(S(=O)([O-])=Nc2cc3nc([nH]c3cc2)CCCC)ccc1 | 0 |
| Decoy348 | s1cc(nc1-c1cc(F)ccc1)CC(=O)N(CC)c1ccccc1 | 0 |
| Decoy349 | O=C1C(=CC(=N/N=C(\[NH-])/C(=[NH+]\N=C2C=C(C(C)(C)C)C(=O)C(=C2)C(C)(C)C)/N)C=C1C(C)(C)C)C(C)(C)C | 0 |
| Decoy350 | O=C(Nc1nccnc1C(=O)[O-])c1nc(n[n-]1)C1CC1 | 0 |
| Decoy351 | s1c2c([n+](C)c1[N-]\N=C\1/CC(C[C@H](C/1)C)(C)C)cccc2 | 0 |
| Decoy352 | OC1=Nc2c(N(/C(/[O-])=[NH+]/[C@H](/C(/O)=N/[C@H](C(=O)[O-])c3ccccc3)C(C)C)[C@H]1C(C)C)cccc2 | 0 |
| Decoy353 | S(CC(=O)Nc1ccc(cc1)-c1oc2c(n1)cc(NC(=O)CSc1[nH]c3c(n1)cccc3)cc2)c1[nH]c2c(n1)cccc2 | 0 |
| Decoy354 | s1cc(nc1-c1ccc(F)cc1)\C=C/C(=O)N1CCC(NS(=O)(=O)c2sccc2)CC1 | 0 |
| Decoy355 | S1\C(=C/c2cc(OCC(=O)[O-])ccc2)\C(=O)N([C@H]([C@H](CC)C)C(=O)[O-])C1=S | 0 |
| Decoy356 | S(CC(=O)Nc1cc(cc(c1)C)C)C1=NN[C@H](N1N)[C@@H]1N=NC2=C1CCC2 | 0 |
| Decoy357 | S([C@H](C(=O)N(Cc1ccccc1)C)C)c1[nH+]c2c(n1CC=C)cccc2 | 0 |
| Decoy358 | S(C)C1=NC(=O)NC(C)=C1C(=O)N[C@@H]1CCC(=O)NC1=O | 0 |
| Decoy359 | Brc1cc2sc(nc2cc1)\N=C(/[O-])\[C@@H](Oc1ccccc1)C | 0 |
| Decoy360 | Ic1cc2nc(n(c2cc1F)-c1ccc(Br)cc1)[C@H](Cl)C | 0 |
| Decoy361 | S(Cc1nc(nc(n1)N)N)c1nnc(n1-c1ccccc1)-c1cc(OC)ccc1 | 0 |
| Decoy362 | O=CN1CCCC[C@@H]1c1cccnc1-n1c(ccc1C)C | 0 |
| Decoy363 | [n+]1(ccn(Cc2ccccc2)c1C)CCCCCCCCCCCCCCCC | 0 |
| Decoy364 | FC(F)(Oc1ccc(cc1)-c1[nH+]c2n(c1)CCC2)[C@@H](F)C(F)(F)F | 0 |
| Decoy365 | S(S\C(=C\C)\C[C@H](O)N(Cc1c[nH+]c(nc1N)C)C=O)\C(=C/C)\C[C@@H](O)N(Cc1c[nH+]c(nc1N)C)C=O | 0 |
| Decoy366 | O(C(=O)N1CCC([NH+]2CCCCC2)CC1)c1cc2c(nc3c(CN4C3=CC([C@@](O)(CC)C(=O)[O-])=C(CO)C4=O)c2CC)cc1 | 0 |
| Decoy367 | Brc1cc2c(ncnc2OCC(=O)N(CC=C)CC=C)cc1 | 0 |
| Decoy368 | Clc1nnc(-n2nc(c3c2N=C(O)C[C@@H]3c2ccccc2OCc2ncccc2)C)cc1 | 0 |
| Decoy369 | s1nnc(C(OCC)=O)c1Sc1n(nnc1C(OCC)=O)-c1ccc(OCC)cc1 | 0 |
| Decoy370 | Brc1cc(\C=N\c2nc3c(n2CC)cccc3)c([O-])cc1 | 0 |
| Decoy371 | [S-]c1nnc(n1CCNC(=O)[C@H]([NH+]1CCc2c(C1)cccc2)C)C | 0 |
| Decoy372 | S1N(C(=S)C2=C1C(Nc1c2cc(OCC)cc1)(C)C)c1ccc(S(=O)([O-])=Nc2nc(ccn2)C)cc1 | 0 |
| Decoy373 | s1c2c(nc1NC(=O)NC1CCCC1)CCN(S(=O)(=O)c1ccccc1)C2 | 0 |
| Decoy374 | Brc1ccc(cc1)-c1c2n(nc1CC)C(=CC=N2)c1sccc1 | 0 |
| Decoy375 | S(=O)([O-])(=Nc1ccc(NS(=O)(=O)[C@@H](C(=O)[O-])C)cc1C)C | 0 |
| Decoy376 | FC(F)(F)c1cc(ccc1)C#CCNC(=O)NCc1ccc(OCc2ccc[nH+]c2)cc1 | 0 |
| Decoy377 | Clc1cccc(-n2c(C)c(cc2C)\C=C/2\C(N3N=C(SC3=NC\2=O)Cc2sccc2)=N)c1C | 0 |
| Decoy378 | Ic1c(nc(nc1[O-])-c1oc(Br)cc1)CCC | 0 |
| Decoy379 | Brc1ccc(cc1)C(=O)[C@@H]([NH+]1C[C@H](OC[C@@H]1CC)C)C | 0 |
| Decoy380 | Br[C@H](c1c(F)cc(F)cc1F)c1oc([N+](=O)[O-])cc1 | 0 |
| Decoy381 | S(=O)(=O)(Nc1ccccc1OC)c1cc(Nc2nc(ncn2)N)ccc1 | 0 |
| Decoy382 | O=C(N\N=C/c1c2c([nH]c1C)cccc2)C12CC3CC(C1)CC(C2)C3 | 0 |
| Decoy383 | SCC[NH+](CCCCOc1ccc(OC)cc1)CCCCOc1ccc(OC)cc1 | 0 |
| Decoy384 | Brc1scc(c1)[C@@H]([NH2+]CC)c1oc2c(c1)cccc2 | 0 |
| Decoy385 | s1cc(nc1N(C(=O)C)c1ccccc1CC)C[NH+](Cc1ccc(OCC)cc1)C | 0 |
| Decoy386 | Br[C@H]1C[C@H](Oc2ccc(cc2C)C(C)(C)C)C1(C)C | 0 |
| Decoy387 | s1c(ccc1C)CN=S(=O)([O-])N(CCC(=O)[O-])C | 0 |
| Decoy388 | Ic1cc(ccc1C)C(=O)Nc1ccc(S(=O)([O-])=Nc2noc(c2)C)cc1 | 0 |
| Decoy389 | [NH3+]N[C@H](C1([NH+]2CCCCC2)CCCC1)c1ccc(nc1)C | 0 |
| Decoy390 | S(=O)([O-])(=Nc1ccccc1CC)c1cc2c(NC=C(C(=O)N[C@@H](CC)C)C2=O)cc1 | 0 |
| Decoy391 | [NH+]1(CCN(CC1)Cn1c2c(nc1)cccc2)C\C=C\c1ccccc1 | 0 |
| Decoy392 | Ic1c([nH+]c(nc1NCCC)N1CCCCC1)CC | 0 |
| Decoy393 | Brc1cc(ccc1)C(=O)N(S(=O)(=O)c1ccccc1)c1ccc(cc1)C | 0 |
| Decoy394 | Ic1ccc(cc1)C[C@@H]([NH2+]N)[C@@H]1C[C@@H]1c1ccccc1 | 0 |
| Decoy395 | S(=O)(=O)(CNC(=O)[C@H](N(C(=O)Cc1ccccc1)CCCCCC)\C=C\CCC)c1ccc(cc1)C | 0 |
| Decoy396 | O([C@@H](\C=C(\CCC=C(C)C)/C)C\C(=C\[C@H](OC(=O)C)C[C@](O)(C=C)C)\C)C(=O)C | 0 |
| Decoy397 | Brc1cnn(C)c1[C@@H]([NH2+]C)c1c2c(nccc2)ccc1 | 0 |
| Decoy398 | Brc1sc(cc1C)C(=O)N1CCC(CC1)C[NH+](C(C)C)C | 0 |
| Decoy399 | Oc1cc(O)cc(O)c1[C@H](N(C(=O)c1ccc([N+](=O)[O-])cc1)C)C(=O)NC(C)(C)C | 0 |
| Decoy400 | Brc1ccc(cc1)[C@@H](Oc1cc(Cl)cnc1)[C@H]([NH3+])CC | 0 |
| Decoy401 | Clc1cc2nc(n(c2cc1F)-c1cc(ccc1)C#N)CCl | 0 |
| Decoy402 | s1ccc(C#CCO)c1CN1C(=O)[C@@H](C)[C@H](C)C1=O | 0 |
| Decoy403 | S(=O)([O-])(=Nc1ccc(cc1)-c1[nH]nc(n1)C)c1ccc(cc1C)C | 0 |
| Decoy404 | s1c2CC[C@H](Cc2cc1C(=O)N\N=C\c1n2c(SC=C2C)nc1C)CC | 0 |
| Decoy405 | Brc1sc(cc1)C(=O)C[NH+](Cc1cc(ccc1)C#N)C | 0 |
| Decoy406 | Ic1cc2[nH+]c(n(c2cc1)[C@@H](CCC)COC)[C@H](Cl)C | 0 |
| Decoy407 | s1c(nnc1\N=C(/[O-])\[C@@H]1CC(=O)N([C@H]1c1sccc1)c1ccc(cc1)C)C(C)C | 0 |
| Decoy408 | Brc1cc([N+](=O)[O-])ccc1C[NH2+][C@@H](C)c1ccc(Cl)cc1 | 0 |
| Decoy409 | O=C1N(C)C(=O)c2c1cc(cc2)C(=O)N[C@@H]1CCCc2n(ncc12)-c1cc(C)c(cc1)C | 0 |
| Decoy410 | Brc1ccc(S(=O)(=O)N(CC2=Cc3c(NC2=O)c(C)c(cc3)C)C[C@@H]2OCCC2)cc1 | 0 |
| Decoy411 | Fc1ccc(cc1)C(=O)C=1[C@H](N(CCO)C(=O)C=1[O-])c1occc1 | 0 |
| Decoy412 | Clc1cc(-n2nnc(C(=O)N3CC[C@H](C[C@@H]3C)C)c2C)ccc1F | 0 |
| Decoy413 | S(CC(=O)N(Cc1ccc(F)cc1)CC[NH+](C)C)c1nncn1-c1cc(C)c(cc1)C | 0 |
| Decoy414 | S1(=O)(=O)C[C@H](\[NH+]=C\C=C\2/N([C@@H]3[C@@H](C=CC=C3)C/2(C)C)C)CC1 | 0 |
| Decoy415 | Fc1cc(ccc1)CN1N=C(C=CC1=O)C(=O)Nc1ccc(cc1)-c1nnc(N2CCC(CC2)C)cc1 | 0 |
| Decoy416 | S1C=2N(N=C1COc1ccccc1)C(=N)\C(=C\c1c3c(n(CCOc4c(cccc4C)C)c1C)cccc3)\C(=O)N=2 | 0 |
| Decoy417 | BrCCCCCCCCC[NH+]1CCN(CC1)CC[NH+](C)C | 0 |
| Decoy418 | Clc1cc([N+](=O)[O-])ccc1N=S(=O)([O-])CCCC(=O)[O-] | 0 |
| Decoy419 | O1CC(=CC1=O)[C@H]1CC[C@]2(O)[C@H]3[C@H](CC[C@]12C)[C@]1(CC[C@H](O[C@@H]2O[C@H](C)[C@@H](O[C@@H]4O[C@H](CO)[C@@H](O)[C@H](O)[C@H]4O)[C@@H](O)C2)C[C@@]1(O)CC3)CO | 0 |
| Decoy420 | Brc1sc(cc1)C[NH2+]CC1(O)CCC(CC1)CC | 0 |
| Decoy421 | o1cccc1\C=C\C[NH+]1CCC2(N(CCc3ccccc3)C(=O)N(Cc3cccnc3)C2=O)CC1 | 0 |
| Decoy422 | S(CC(=O)NCC(=O)Nc1cc(F)c(F)cc1)c1nnc(n1N)C(F)(F)F | 0 |
| Decoy423 | S(=O)(=O)(N(CC1CC1)C(C)C)c1cn(nc1)CCC(=O)[O-] | 0 |
| Decoy424 | o1cccc1C(=O)N1CCCN(CC1)c1nc(nc2onc(c12)-c1ccccc1)CCCC | 0 |
| Decoy425 | s1c(nnc1\N=C(/[O-])\c1ccc(N2C(=O)c3c(cccc3)C2=O)cc1)C(F)(F)F | 0 |
| Decoy426 | o1nc(nc1CN1C(=O)[C@]2(NC1=O)CCc1c(C2)cccc1)-c1ccccc1 | 0 |
| Decoy427 | Brc1c(n(nc1C)CC)C[C@@H]([NH2+]CCC)[C@H]1C[C@H](CC1)C | 0 |
| Decoy428 | ClCCC(=O)N(CCCOC)CC(=O)N(Cc1ccccc1)Cc1sccc1 | 0 |
| Decoy429 | Clc1ccc(cc1S(=O)(=O)N)C(=O)N\N=C(/C)\c1ccc(S(=O)(=O)NCC(=O)[O-])cc1 | 0 |
| Decoy430 | s1c(nnc1SCC(=O)C=1C(=O)NC(=O)N(C=1N)C1CC1)Nc1cc(F)ccc1 | 0 |
| Decoy431 | P(=O)([O-])(CCC(=O)[O-])CC[C@]([NH3+])(C(C)C)C(=O)[O-] | 0 |
| Decoy432 | Brc1cc2[C@@H]3N(N=C(C3)c3sccc3)[C@@H](Oc2cc1)c1ccsc1 | 0 |
| Decoy433 | s1c(nnc1N=S(=O)([O-])C)NC(=O)Cc1cc(ccc1)C(F)(F)F | 0 |
| Decoy434 | Brc1ccccc1[C@@H]1C2C(=NC(=C)C1C#N)C[C@@H](CC2=O)c1ccccc1 | 0 |
| Decoy435 | Brc1ccc(Br)cc1S(=O)(=O)n1c2c(nc1)cccc2 | 0 |
| Decoy436 | S(CCCCCC)CCOCCOCCOC[C@H](O)CSCCCCCC | 0 |
| Decoy437 | s1ccc(C)c1[C@@H]\1[C@@H]2C(=NC(SCC(C)C)=NC2=O)N=C(C)/C/1=C(/OC)\[O-] | 0 |
| Decoy438 | O=C1NC=2N=C(NC(=O)C=2[C@@H]([C@H]1NC(=O)c1ccccc1)c1ccccc1)N1CCc2c(C1)cccc2 | 0 |
| Decoy439 | BrC[C@@H](CCC)C[NH+]1CC(CC1)(CC)CC | 0 |
| Decoy440 | Clc1cc(Cl)cnc1N1CCN(CC1)C(=O)[C@H]1CC1(Cl)Cl | 0 |
| Decoy441 | ClCc1[nH+]c2cc(ccc2n1[C@@H]1C[C@@H](C)[C@@H](CC1)C)C | 0 |
| Decoy442 | o1c(ccc1[C@H]([NH2+]Cc1cn(nc1-c1ccccc1)Cc1ccccc1)C)C | 0 |
| Decoy443 | S(=O)([O-])(=Nc1ccc(cc1)-c1ccccc1)c1cc2NC(=O)Nc2cc1 | 0 |
| Decoy444 | Br[C@@H]1CC[C@@H](Cc2[nH+]c3c(n2CCC)cccc3)[C@H]1C | 0 |
| Decoy445 | Brc1cc(S(=O)(=O)N)ccc1Oc1cc(Br)cnc1 | 0 |
| Decoy446 | S1\C(=C/c2cn(nc2-c2ccccc2)-c2ccccc2)\C(=O)N(C2CCCCC2)C1=S | 0 |
| Decoy447 | S(=O)(=O)(N1CCCCC1)c1cc2[nH+]c(n(c2cc1)C)CCC(=O)NCCCc1n[nH]c(N)c1C#N | 0 |
| Decoy448 | Fc1ccc(cc1)[C@@H]1[NH+]=C2C(C=CC=C2)=C1CCC(=O)N(OC)C | 0 |
| Decoy449 | S(=O)([O-])(=Nc1cc(F)ccc1C)c1ccccc1NCC | 0 |
| Decoy450 | O(C)c1nc2c(cc1CNC(=O)[C@H]([NH3+])[C@@H](CC)C)C(=O)N(C2)C1CCCC1 | 0 |
| Decoy451 | Brc1ccc(cc1C(F)(F)F)-c1oc(cc1)[C@@H]([NH2+]C)C | 0 |
| Decoy452 | Clc1ccc(cc1)-c1sc(CCNS(=O)(=O)c2ccc(cc2C)C)c(n1)C | 0 |
| Decoy453 | Brc1cccnc1N=S(=O)([O-])c1c(C)c(ccc1N)C | 0 |
| Decoy454 | S(=O)(=O)(\N=C\1/NC(OC)=NC(OC)=C/1)c1ccc(NN=C2C(=O)CC(CC2=O)(C)C)cc1 | 0 |
| Decoy455 | S=C1N=C([O-])[C@@H]2C(OC(N)=C(C#N)[C@@H]2c2ccc([N+](=O)[O-])cc2)=N1 | 0 |
| Decoy456 | Fc1ccc(cc1)CN([C@H](C(=O)c1c(C)c([nH]c1C)C(OCC)=O)C)C(=O)c1ccccc1 | 0 |
| Decoy457 | Clc1cc2[nH+]c(n(c2cc1F)[C@@H]1CCC[C@@H]1C)CCCl | 0 |
| Decoy458 | O=C(Nc1c(nc(nc1Nc1ccc(cc1)C)Nc1ccc(cc1)C)C)c1ccc(cc1)C | 0 |
| Decoy459 | Brc1ccc(Br)cc1S(=O)(=O)n1cc[nH+]c1 | 0 |
| Decoy460 | Clc1ccc(Oc2ncnc(NCc3ccc(cc3)C)c2N)cc1C | 0 |
| Decoy461 | S(CC(=O)N1CC[NH+](CC1)Cc1oc(nn1)C(C)C)c1nnc(n1N)N\N=C\c1cc(O)ccc1 | 0 |
| Decoy462 | Fc1cc(ccc1)C[NH+](Cc1n(ccc1)-c1ccc(F)cc1)CCCn1ccnc1 | 0 |
| Decoy463 | Brc1c(n(nc1CC)CC)C[NH+]1CCC(CC1)CCl | 0 |
| Decoy464 | ClC=1C=Cc2n(C=1)c(C)c(n2)Cn1cc[nH+]c1-c1cc(F)ccc1F | 0 |
| Decoy465 | Brc1ccc(cc1)-c1sc(nc1)C[NH2+]C[C@H]1[C@H]2C=C[C@@H](C1)C2 | 0 |
| Decoy466 | Ic1ccc(cc1)[C@@H](Nc1ccc([nH+]c1)N(CC)CC)C | 0 |
| Decoy467 | Clc1cc(F)ccc1[C@@H](Nc1c2nsnc2ccc1Cl)C | 0 |
| Decoy468 | O=C(NCCC)c1ccc(\N=C(/[O-])\C(=O)NCc2ncccc2)cc1 | 0 |
| Decoy469 | s1cccc1CCN(S(=O)(=O)c1ccccc1C#N)C | 0 |
| Decoy470 | Clc1ccc(cc1)-c1noc(c1)C[NH+](CC=C)CC=C | 0 |
| Decoy471 | s1cccc1[C@H](C(=O)N(c1ccccc1)c1scc(n1)C)C | 0 |
| Decoy472 | BrC=1C(=O)N(N=CC=1N[C@H]1[C@H]2O[C@@H](C1)CC2)C | 0 |
| Decoy473 | FC(F)(F)c1nc(ncc1)N1CCC2(CC1)[C@@H](O)C[C@H]2O | 0 |
| Decoy474 | S(=O)(=O)(N1CCC(CC1)c1nnc([S-])n1-c1ccc(cc1)C)c1ccc(cc1)C | 0 |
| Decoy475 | O=C1Nc2c(ccc(C)c2C)[C@]12[NH2+][C@H]([C@@H]1[C@H]2C(=O)N(CCCC)C1=O)CC(=O)N | 0 |
| Decoy476 | Brc1ccccc1Sc1c(nn(c1OC)-c1ccccc1)C | 0 |
| Decoy477 | O1[C@H](CO)[C@@H](O)[C@H](O)[C@@H](O)[C@@H]1O[C@@H]1[C@@H](O)[C@@H](O)CO[C@H]1O[C@H]1CC[C@]2([C@@H](CC[C@@]3([C@@H]2CC=C2[C@@H]4CC(CC[C@@]4(CC[C@]23C)C(=O)[O-])(C)C)C)[C@@]1(CO)C)C | 0 |
| Decoy478 | Brc1cc(Br)cnc1[C@H](N[NH3+])c1cc(ccc1)CC | 0 |
| Decoy479 | Brc1cc2-c3nnc(SC)nc3O[C@H](Nc2cc1)c1ccc(OCC(=O)[O-])cc1 | 0 |
| Decoy480 | Brc1cc([C@H](N[C@H](CC(C)C)C[NH+](C)C)CC)c(O)cc1 | 0 |
| Decoy481 | Brc1sc(S(=O)(=O)N([C@H](C(C)C)C)C)cc1 | 0 |
| Decoy482 | Fc1cc(ccc1CNC(=O)/C(/[O-])=N/c1ccc(cc1)C(=O)C)C#N | 0 |
| Decoy483 | S(=O)(=O)(N[C@@H](CC(=O)[O-])C(=O)[O-])c1c2c3c(ccc2)C(=O)N(c3cc1)CC | 0 |
| Decoy484 | Brc1cc\2c(NC(=O)/C/2=N\NC(=O)c2nnn(c2C[NH+](C)C)-c2nonc2N)cc1 | 0 |
| Decoy485 | S(=O)([O-])(=N[C@@H](C(OC)=O)CO)c1cc(oc1C)C(=O)[O-] | 0 |
| Decoy486 | ClCc1[nH+]c2c(n1[C@H](C(C)C)c1sccc1)cc(F)cc2 | 0 |
| Decoy487 | s1cc(nc1-c1ccccc1)C(=O)N1CC[C@H](SCC1)c1ccccc1C | 0 |
| Decoy488 | Brc1ccccc1[C@@H]([NH2+][C@H](C)c1ccc(cc1O)C)C | 0 |
| Decoy489 | Clc1ccc([N+](=O)[O-])cc1[C@@H]1N2C(=NC(=N1)[N-]c1ccc(cc1)C)N=C(C=C2[O-])C | 0 |
| Decoy490 | Brc1ccccc1[C@H]([NH+](Cc1nnnn1CCCC)CCC)C | 0 |
| Decoy491 | Brc1cc2[nH+]c(n(c2cc1F)C[C@H]1OCCC1)CCCl | 0 |
| Decoy492 | O=C1Nc2n(nc(c2[C@H](C1)c1nc2c(cc1)cccc2[O-])C(C)(C)C)C | 0 |
| Decoy493 | s1cccc1-c1n(nnc1C(=O)N\N=C(\C)/c1ccc(cc1)C#N)-c1nonc1N | 0 |
| Decoy494 | Clc1ccccc1C=1N=C(SCC=2N=C3SC(=NN3C(=O)C=2)CC)N2NC(=O)C=C2N=1 | 0 |
| Decoy495 | ClC=1C=Cc2nc(cn2C=1)C[NH+](Cc1cc(OCC)c(OC(F)F)cc1)C1CC1 | 0 |
| Decoy496 | S(CCc1n(cnc1-c1ccccc1)CC[C@H](O)C(=O)[O-])C | 0 |
| Decoy497 | S(=O)([O-])(=Nc1ccc(cc1)-c1oncc1)c1cc(ccc1C)-c1onc(c1)C | 0 |
| Decoy498 | Brc1cc2c(cc(cc2)[C@@H](N[NH3+])CCCC(C)C)cc1 | 0 |
| Decoy499 | Brc1cc(sc1)C[C@@H](N[NH3+])[C@@H]1[C@H]2[C@@H]1c1c(CC2)cccc1 | 0 |
| Decoy500 | S(c1c2ncccc2ccc1)c1oc(cc1)\C=[NH+]\c1cc(ccc1)C(=O)C | 0 |
| Decoy501 | Brc1c(n(nc1C)CC)C[NH+](C)C1CCC(CC1)(C)C | 0 |
| Decoy502 | S(=O)(=O)(N1CCc2c(C1)cccc2)CCNC(=O)c1ccc(OCCCC)cc1 | 0 |
| Decoy503 | S=P(N\N=C\c1ccc(OCCC(C)C)cc1)(c1ccccc1)c1ccccc1 | 0 |
| Decoy504 | Brc1cc(S(=O)(=O)Nc2cc(cc(c2)C(F)(F)F)C=2NC(=O)[N-]N=2)ccc1OC | 0 |
| Decoy505 | s1c2c(ncnc2SCC)c2c3CC(OCc3c(nc12)C)(C)C | 0 |
| Decoy506 | Brc1ccc(cc1)[C@@H]([C@@H](O)c1sc(cc1)CC)C[NH3+] | 0 |
| Decoy507 | S(=O)(=O)(\N=C(/[O-])\C)c1ccc(NC(=O)[C@@H]2CCCN(C2)c2nnc(-n3nccc3)cc2)cc1 | 0 |
| Decoy508 | Brc1cc(C)c(NC(=O)c2cc(nc3c2cc(Br)cc3C)-c2ccccc2)cc1Cl | 0 |
| Decoy509 | O=C1c2c(cccc2NC(=O)[C@@H](OC(=O)c2c(n(nc2C)-c2ccccc2)C)C)C(=O)c2c1cccc2 | 0 |
| Decoy510 | Brc1ccsc1C[NH2+][C@@H](C)c1ccc(OC(F)(F)F)cc1 | 0 |
| Decoy511 | S=C(N(Cc1ccc(cc1)C(C)C)Cc1occc1)NCCc1cc(OCC)c(OCC)cc1 | 0 |
| Decoy512 | s1cccc1\C=C\c1onc(C)c1S(=O)(=O)N1CCC(CC1)C(=O)Nc1cc([nH+]c2c1cccc2)C | 0 |
| Decoy513 | S(=O)(=O)(N1CCC(OCCC(=O)[O-])CC1)c1[n-]cnc1 | 0 |
| Decoy514 | Clc1cc(ccc1C)-c1oc(cc1)\C=C\C(=O)N/C(/[S-])=N/c1cccnc1Cl | 0 |
| Decoy515 | s1c(NC(=O)c2ccccc2)c(cc1C)[C@@H](Nc1[nH+]ccc(c1)C)c1ncccc1 | 0 |
| Decoy516 | S1C=2C[C@@H](CCC=2N(CC(=O)N(CC2CC2)C)C1=O)CC | 0 |
| Decoy517 | S\1C=CN/C/1=N\S(=O)(=O)c1ccc(NC(=O)c2cc(S(=O)(=O)Nc3ccc(cc3C)C)c(cc2)C)cc1 | 0 |
| Decoy518 | Fc1ccc(cc1)C1=NNC(=[NH+]c2c1cc(OC)cc2)c1ccc(OCC)cc1 | 0 |
| Decoy519 | S1C[C@@H]([NH2+][C@H]2CCS(=O)(=O)C2)c2c(C1)cccc2 | 0 |
| Decoy520 | Clc1ccc(S(=O)(=O)\N=C\2/Sc3cc(F)ccc3N/2CC=C)cc1 | 0 |
| Decoy521 | S(=O)(=O)(N(CCCCCCCCCCCCCCCC)C)c1ccc(nc1)NN | 0 |
| Decoy522 | Fc1ccc(cc1)CCNC(=O)C1=CN(C=C(C(=O)NCC=C)C1=O)Cc1oc(cc1)C | 0 |
| Decoy523 | S(=O)(=O)(\N=C(/[O-])\c1cnn(c1C)-c1ncc(cc1)C(F)(F)F)c1c(C)c(cc(C)c1C)C | 0 |
| Decoy524 | s1c(C)c(cc1C)C[NH+]1C[C@H](CCC1)Cn1nnc2c1nc(nc2[O-])C(C)C | 0 |
| Decoy525 | S(CC(=O)N(CC(=O)Nc1ccccc1C(F)(F)F)C)c1ccccc1F | 0 |
| Decoy526 | s1c2c(nc1CCCC(=O)\C(=C(/[O-])\NCCC)\C#N)cccc2 | 0 |
| Decoy527 | Brc1c(n(nc1C)CC)C[C@@H]([NH3+])Cc1ccc(F)cc1Cl | 0 |
| Decoy528 | O=C(N([C@@H](CCCCC)C(=O)NC(C)(C)C)CCCCCC)[C@H](CC)c1ccccc1 | 0 |
| Decoy529 | s1c2CCCCc2c2c1N[C@@H](NC2=O)c1ccc([NH+](CC)CC)cc1 | 0 |
| Decoy530 | s1c2CCN(Cc2cc1)C(=O)C[NH2+]C(CC)(CC)C(=O)[O-] | 0 |
| Decoy531 | Brc1ccc(cc1)C(=O)COC(=O)c1ccccc1Sc1ccccc1C(OCC(=O)c1ccc(Br)cc1)=O | 0 |
| Decoy532 | o1nc(nc1CCN(CCCC)C(=O)Nc1ccc(cc1)C(C)C)-c1ccc(N(C)C)cc1 | 0 |
| Decoy533 | S\1\C(=C\c2ccsc2)\C(=O)N(/C/1=C(/C(=O)C(C)C)\C#N)c1ccccc1C | 0 |
| Decoy534 | Brc1ccsc1CN1[C@H](C)C(=O)N[C@H](C)C1=O | 0 |
| Decoy535 | Brc1ccc(cc1)C(=O)NNc1ncnc(N2CCN(CC2)C(OCC)=O)c1N | 0 |
| Decoy536 | s1c2c(nc1N(C(=O)CCS(=O)(=O)c1ccc(cc1)C)CCCn1cc[nH+]c1)cccc2 | 0 |
| Decoy537 | s1c2c(nc1NC(=O)C)cc(N=S(=O)([O-])c1cc(ccc1)C)cc2 | 0 |
| Decoy538 | Brc1cc(cc(N[C@@H](C)[C@H]2CCC[NH+](C2)C)c1)C(F)(F)F | 0 |
| Decoy539 | Clc1cccc(F)c1-c1c(nn(C)c1N)C1(CCCC1)C | 0 |
| Decoy540 | S(CC(=O)N[C@H](C)c1ccccc1)c1nnc(n1N)N\N=C(/C)\c1cc(OC)ccc1 | 0 |
| Decoy541 | s1c(nnc1SCCOC)\N=C(/[O-])\CSc1nc([nH]n1)N\N=C\c1c(n(nc1C)C(C)C)C | 0 |
| Decoy542 | Brc1cc(F)ccc1[NH+]1CC([NH2+]C[C@@H]1CC)(CC)CC | 0 |
| Decoy543 | Brc1ccc(cc1)CN(Cc1[nH+]cccc1)C(=O)[C@@H]1CCC=CC1 | 0 |
| Decoy544 | Clc1ccc(O[C@H](C)c2nnc(SCC(=O)NC=3C(=O)N(N(C)C=3C)c3ccccc3)n2-c2ccccc2)cc1 | 0 |
| Decoy545 | S(=O)(=O)(N1CCC(NC(=O)C2CCC(CC2)C)CC1)C(F)(F)F | 0 |
| Decoy546 | Br[C@H]1CC[C@H](C[C@@H]1Cc1ncnn1C(C)C)C(C)(C)C | 0 |
| Decoy547 | Fc1ccc(cc1)[C@H]1C[C@H](N(C1)C(=O)c1n2C=C(C=Cc2nc1)C)C | 0 |
| Decoy548 | Clc1ccc(cc1)CSc1c2c(n(c1)-c1ccc([N+](=O)[O-])cc1)cccc2 | 0 |
| Decoy549 | s1cccc1-c1nn(cc1/C(/[O-])=N/c1sc2c(n1)CC[NH+](C2)C)-c1ccccc1 | 0 |
| Decoy550 | s1c2c(N=C(SCC(=O)NCCc3ccc(S(=O)(=O)N)cc3)N(C3CCN(CC3)C(OCC)=O)C2=O)cc1 | 0 |
| Decoy551 | S1(=O)(=O)N=C(Nc2ccc(cc2)C(=O)Nc2cc(ccc2)C(F)(F)F)c2c1cccc2 | 0 |
| Decoy552 | Brc1cc(cc(Cl)c1OCc1ncccc1)C=O | 0 |
| Decoy553 | O=C(N1CCN(CC1)c1n[nH]c(c1)-c1cccnc1)c1ccc(cc1)C | 0 |
| Decoy554 | S(=O)(=O)(N)c1cc(N=S(=O)([O-])[C@H](C#N)C)cc(F)c1 | 0 |
| Decoy555 | O1c2cc(\C=N\NC(=O)[C@H](NC(=O)c3ccccc3)C3=NNC(=O)c4c3cccc4)c([N+](=O)[O-])cc2OC1 | 0 |
| Decoy556 | Ic1cc2[nH+]c(n(c2cc1F)CCCCC)N | 0 |
| Decoy557 | Clc1ncc(S(=O)(=O)Nc2cn(nc2)C)cc1C | 0 |
| Decoy558 | Brc1ccc(cc1)C=1N(/C(/SC=1)=N\C(=O)c1ccccc1)c1ccccc1 | 0 |
| Decoy559 | S(=O)(=O)(N(C)C)c1ccccc1S(=O)(=O)\N=C(/[O-])\Nc1nc(N2CC[NH2+]CC2)cc(F)n1 | 0 |
| Decoy560 | S=C1N(C[C@@H]2OCCC2)C(N)=C(C2=C1COC(C2)(C)C)C#N | 0 |
| Decoy561 | Brc1cc(Cl)c(N[C@H](CCC)C)c(Cl)c1 | 0 |
| Decoy562 | s1c(SCc2ccc(cc2)C(C)C)nnc1SCC(=O)NCC#C | 0 |
| Decoy563 | Brc1cc(SCc2[nH+]c(cc(n2)NCCC)CC)ccc1 | 0 |
| Decoy564 | s1c2c(nc1NC(=O)c1ccc(OC)cc1)[C@@H](CC(=O)NCCNC(=O)C)[C@]1([C@H](C2)[C@@](CO)(C)[C@H](O)CC1)C | 0 |
| Decoy565 | Clc1cc(F)c(NC(=O)C2CCN(S(=O)(=O)c3scc(c3)-c3nc(on3)CC)CC2)cc1 | 0 |
| Decoy566 | Fc1cc2c(CC[C@@H]2NC(=O)Nc2cc(ccc2C)-c2occn2)cc1 | 0 |
| Decoy567 | Clc1cc(ccc1)-c1nc(on1)C[NH+](Cc1ccc(cc1C)C)C | 0 |
| Decoy568 | Brc1ccc(S(=O)([O-])=N\N=C(/C)\c2cc([N+](=O)[O-])ccc2)cc1 | 0 |
| Decoy569 | Brc1cc([N+](=O)[O-])cc(S(=O)(=O)\N=C\2/NC=C(F)C=C/2)c1 | 0 |
| Decoy570 | Brc1c(n(nc1C(F)(F)F)CCC(=O)NN1C(=Nc2sc3CCCCc3c2C1=O)C)C | 0 |
| Decoy571 | s1cccc1-c1nc(cc([O-])n1)[C@@H]1CCC[NH+](C1)Cc1cnc(nc1)N(C)C | 0 |
| Decoy572 | O=C1N(CC[C@@H]([NH+](CC23CC4CC(C2)CC(C3)C4)C)CC1)CC | 0 |
| Decoy573 | s1c2c(CCC2)c(C(=O)Nc2ccc(OC)cc2)c1NC(=O)C[NH2+]Cc1ccc[nH+]c1 | 0 |
| Decoy574 | Brc1nc(sc1)N[C@@H]1CCC(=O)N(C)C1=O | 0 |
| Decoy575 | O1CC[NH+](CC1)C[C@@H](O)CC=1C(=O)N(C[C@H](O)C[NH+]2CCOCC2)C(=O)N(C[C@@H](O)C[NH+]2CCOCC2)C=1[O-] | 0 |
| Decoy576 | S(CCCC\C=C(/NC(=O)[C@@H]1CC1(C)C)\C(=O)[O-])C[C@H]([NH3+])C(=O)[O-] | 0 |
| Decoy577 | Brc1ccc(cc1)COc1c(cc(cc1OCC)\C=C/1\SC(=S)N(C\1=O)c1cc(Cl)c(F)cc1)CC=C | 0 |
| Decoy578 | S(=O)(=O)(NCCC(F)(F)F)c1cc(cnc1)\C=C\C(=O)[O-] | 0 |
| Decoy579 | O=C1N(N=C(C=C1)C(=O)Nc1ccc(NC(=O)N)cc1)CCC | 0 |
| Decoy580 | Ic1ccc(N2C[C@H](C)[C@@H]([NH2+]CCC)CC2)cc1 | 0 |
| Decoy581 | O=C(N1CCCC1)c1nn(c2c1C[C@@H]([NH+](CC(C)=C)CC)CC2)CC(C)C | 0 |
| Decoy582 | [nH+]1ccn(c1)[C@H]1CCC[C@H]1[NH2+][C@@H]1CCc2cc(N)ccc12 | 0 |
| Decoy583 | O1c2cc(N(CC)CC)ccc2[C@@H](C(C#N)=C1N)c1ccc(OCCCC)cc1 | 0 |
| Decoy584 | S(=O)([O-])(=NC=1C=CC(=O)N(C=1)CCC)[C@H](C(=O)[O-])C | 0 |
| Decoy585 | S(=O)(=O)(NNc1ncnc(N2CCC(CC2)C(OCC)=O)c1N)c1ccc(cc1)C | 0 |
| Decoy586 | n1c(nc(nc1N(C)C)N)CCCCCCCCCCCCCCCCC | 0 |
| Decoy587 | s1c(C)c(CC)c(C(OC)=O)c1NC(=S)Nc1cnn(C)c1C(=O)N | 0 |
| Decoy588 | O=C(N1C[C@]2([NH2+]Cc3c([NH+]=C2NC2CCCC2)cccc3)CC1)c1nn(cc1)CC | 0 |
| Decoy589 | Brc1oc(cc1)C(=O)NC=1C(=O)NC(SCC(=O)NCc2ccccc2)=NC=1N | 0 |
| Decoy590 | S(=O)([O-])(=NCC[NH+]1CCOCC1)N1CCCC[C@@H]1C(=O)[O-] | 0 |
| Decoy591 | s1c2cc(OCC)ccc2nc1N(C(=O)Cc1ccc(cc1C)C)CCC[NH+](C)C | 0 |
| Decoy592 | S(=O)(=O)(CNC(=O)[C@@H](N(CCC)C(=O)\C=C\CCCCC)CCCCC)c1ccc(cc1)C | 0 |
| Decoy593 | O=C1N(C(=C(C(=O)c2ccccc2)C([O-])=C1c1ccccc1)c1ccccc1)c1ccccc1 | 0 |
| Decoy594 | Brc1c([O-])c(Br)cc(\C=N\NC(=O)c2nnn(c2CN2CCc3c2cccc3)-c2nonc2N)c1O | 0 |
| Decoy595 | s1c(C(OCC)=O)c(C)c(C#N)c1NC(=O)CSc1nc([nH]n1)N\N=C\c1ccc([N+](=O)[O-])cc1 | 0 |
| Decoy596 | S(=O)([O-])(=NNC(=O)c1ccc(OCCCCCC)cc1)c1ccc(OCCOC)cc1 | 0 |
| Decoy597 | S(C)c1ccc(cc1)[C@@H]1Nc2c([C@H]3[C@H]1CC=C3)cccc2OCC(C)C | 0 |
| Decoy598 | S1C=2N(NC1=C1C=CC(=O)C=C1)C(=N)\C(=C/c1ccsc1)\C(=O)N=2 | 0 |
| Decoy599 | Brc1cc(C)c(NC(=S)[N-]\N=C/c2ccccc2OCCC)cc1 | 0 |
| Decoy600 | s1ccc(C)c1[C@H]1CC(=O)Nc2n(nc(c12)C)-c1nc(cc(n1)C)C | 0 |
| Decoy601 | Brc1oc(cc1)-c1oc(nn1)NC(=O)c1c(F)cccc1F | 0 |
| Decoy602 | Brc1cc(sc1)C[C@H]([NH2+]C)C1(N(CC)CC)CCCC1 | 0 |
| Decoy603 | Brc1ccccc1C=1C(=O)N=C(NC=1[O-])c1occc1 | 0 |
| Decoy604 | S\1C=C(N/C/1=N/S(=O)(=O)[C@@H](C#N)C)c1ccc(N)cc1F | 0 |
| Decoy605 | Brc1sc(cc1)-c1nc(NN)c2ccsc2n1 | 0 |
| Decoy606 | Brc1c(nc(nc1Cl)-c1scc(Br)c1)-c1ccccc1 | 0 |
| Decoy607 | o1c(ccc1C)CN(C(=O)CN(C(=O)CC(C)(C)C)CCCOC)CCc1ccccc1 | 0 |
| Decoy608 | FC(F)(F)C=1N=C(NC(=O)C=1)c1ccc(cc1)C[NH+](C[C@@H]1OCCOC1)C | 0 |
| Decoy609 | S1C[C@@H](N2[C@]1(CCC2=O)C)C(=O)Nc1ccc(cc1)-c1nc2SC=Cn2c1 | 0 |
| Decoy610 | s1c2c(CCC2)c(C(=O)N)c1NC(=O)CN1C(=O)[C@](NC1=O)(CC)C | 0 |
| Decoy611 | ClCc1[nH+]c2c(n1-c1ccc(cc1)CC)cc(cc2)C | 0 |
| Decoy612 | S=C1N(C2CCCC2)C(=N[N-]1)N1CC[C@H](CCC1)C(C)C | 0 |
| Decoy613 | S1\C(=C/2\c3c(N(Cc4ccccc4)C\2=O)cccc3)\C(=O)N(c2cc(ccc2)C(=O)[O-])C1=S | 0 |
| Decoy614 | O1CC(=CC1=O)[C@H]1CC[C@]2(O)[C@H]3[C@H]([C@]4(CO)[C@@](O)(C[C@@H](O[C@@H]5O[C@H](C)[C@H](O)[C@H](O)[C@@H]5O)C[C@H]4O)CC3)[C@H](O)C[C@]12C | 0 |
| Decoy615 | Clc1ccc(cc1)\C=C/S(=O)([O-])=Nc1cc(ccc1)-c1nccn1CC(=O)[O-] | 0 |
| Decoy616 | Brc1cc([N+](=O)[O-])cnc1N[C@H](C)c1ccccc1Cl | 0 |
| Decoy617 | Brc1c(nc(nc1Cl)-c1ccc(Br)cc1)C(C)C | 0 |
| Decoy618 | O=C(NC[C@@H]1[C@@H]2C[C@H](CC1)C2(C)C)C[NH+]1CCCN(CC1)C | 0 |
| Decoy619 | O=[N+]([O-])c1cc(ccc1[O-])\C=N\Nc1nc(nc(n1)N1CCCCC1)N1CCCCC1 | 0 |
| Decoy620 | S(=O)(=O)(N(Cc1oc(cc1)C)C(C)C)N(CCC(=O)[O-])C | 0 |
| Decoy621 | O=C1N2C(=NC(NCCCc3n[nH]c(N)c3C#N)=C1[N+](=O)[O-])C=CC=C2 | 0 |
| Decoy622 | Clc1cc(N)cc(Cl)c1S(=O)([O-])=Nc1sc(cn1)C | 0 |
| Decoy623 | s1c2cc(S(=O)([O-])=Nc3cn[nH]c3C)ccc2nc1C(C)(C)C | 0 |
| Decoy624 | O=C1N(\N=C/c2cc(n(c2C)-c2ccc(cc2)C)C)C(=O)NC12CCCCC2 | 0 |
| Decoy625 | n1c2c(ccc1\C=C/c1c(n(nc1C)Cc1ccccc1)C)cccc2 | 0 |
| Decoy626 | Fc1ccccc1-c1ccc(cc1)C[C@]1(CCCN(C1)C(=O)Cc1cccnc1)C(=O)N | 0 |
| Decoy627 | S=C1N=C([O-])/C(=C/c2cc([nH]c2C)C)/C(=O)N1c1cc(cc(c1)C)C | 0 |
| Decoy628 | Clc1cc(Cl)c(S(=O)(=O)NCCC(F)(F)F)cc1C[NH2+]C | 0 |
| Decoy629 | Brc1cc(F)c(cc1)Cc1sc([nH+]c1CCC)N | 0 |
| Decoy630 | s1cc(C(=O)N(C(C)C)CC(F)(F)F)c(C)c1S(=O)([O-])=[NH] | 0 |
| Decoy631 | s1c(C[NH3+])c(nc1N1CCc2c1cccc2)C(C)(C)C | 0 |
| Decoy632 | S1[C@H](C=C(C1=N)c1onc(n1)C1CCC(CC1)C(C)C)C | 0 |
| Decoy633 | O1C[C@@H]([NH+](C[C@@H]1C)[C@@H](C[C@@]([NH2+]CCC)(C(=O)[O-])C)C)C | 0 |
| Decoy634 | S(=O)([O-])(=Nc1cc([N+](=O)[O-])ccc1F)CCC(=O)[O-] | 0 |
| Decoy635 | s1cc(nc1N(CC(F)(F)F)C(=O)[C@@H](Cc1ccccc1F)C)C | 0 |
| Decoy636 | S(=O)(=O)(Nc1nc(ccn1)C)c1ccc(NC(=O)\C(=C/c2ccc(OCC(=O)N)cc2)\C#N)cc1 | 0 |
| Decoy637 | Clc1ccc(cc1)-c1c2c(sc1)ncnc2N\N=C\c1ccc(OCC)cc1 | 0 |
| Decoy638 | O=C1c2c([nH]c(C(=O)NNC(=O)C(=O)NC3CC3)c2C)CCC1 | 0 |
| Decoy639 | Clc1c2[nH]c(C[NH+]3CCC(CC3)(CCOc3ccccc3)CO)c(c2ccc1)C | 0 |
| Decoy640 | Brc1ccc(cc1)C1=NN([C@H](C1)c1ccc(Cl)cc1)C=1S[C@@H](CC(=O)[O-])C(=O)N=1 | 0 |
| Decoy641 | O1[C@H](CO)[C@@H](O)[C@H](O)[C@@H](O)[C@@H]1OC(=O)[C@@]12[C@@H](C3=CC[C@@H]4[C@](C[C@@H](O)[C@H]5[C@@](CO)(C)[C@@H](O)[C@H](O)C[C@]45C)(C)[C@@]3(CC1)C)[C@H](O)C(CC2)(C)C | 0 |
| Decoy642 | O1[C@H](C)[C@H](O)[C@H]([NH3+])C[C@@H]1O[C@@H]1c2c(C[C@](O)(C1)C(=O)CO)c(O)c1c(C(=O)c3c(cccc3OC)C1=O)c2O | 0 |
| Decoy643 | O1C(=Cc2c(C1=O)c(O)c1c(OC(=O)[C@H](OC)C[C@@H]1O[C@@H](O)[C@@H](O)[C@@H](O)[C@@H](O)[C@@H](O)CO)c2)C | 0 |
| Decoy644 | Ic1ccc(S(Oc2ccccc2-c2oc3c(n2)cccc3)(=O)=O)cc1 | 0 |
| Decoy645 | [S-]c1nnc(n1CC=C)[C@@H](CCCC)C[C@@H](O)COCC(C)C | 0 |
| Decoy646 | Brc1ccc(cc1)C(=O)Nc1sc2cc(N)ccc2n1 | 0 |
| Decoy647 | S\1\C(=C/c2cccnc2)\C(=O)N/C/1=N\c1cc(C)c(cc1)C | 0 |
| Decoy648 | s1cccc1S(=O)(=O)N1CCN(CC1)c1cc(ccc1)C(F)(F)F | 0 |
| Decoy649 | s1nc2c(n1)cccc2S(=O)(=O)N1CCC[C@@H]1C(=O)Nc1cc(F)c(F)cc1 | 0 |
| Decoy650 | S(=O)(=O)(C)c1ccc(cc1)[C@H]1[NH2+]C[C@@H](CS(=O)C1)C | 0 |
| Decoy651 | O=C1N(c2c(cccc2)[C@]12N(C(=O)CC)[C@H](Cc1c2[nH]c2c1cccc2)C)CCCCC | 0 |
| Decoy652 | S=C1N(C=NN1C[NH+](C[C@@H](Cc1ccc(OC)cc1)C)C)C(C)(C)C | 0 |
| Decoy653 | Ic1ccc(cc1)-c1nc2cc(C#N)c(cc2n1CCC)C#N | 0 |
| Decoy654 | s1c2[nH+]c(sc2cc1C(=O)N(CCC(C)C)C)N(C)C | 0 |
| Decoy655 | O1[C@](O[C@H]2O[C@H](CO)[C@@H](O)[C@H](O)[C@H]2O[C@H]2O[C@@H](CO)[C@@H](O)[C@@H](O)[C@H]2O)(COC(=O)\C=C\c2ccccc2)[C@@H](O)[C@H](O)[C@H]1CO | 0 |
| Decoy656 | Brc1ccc(N)cc1S(=O)(=O)NC[C@H](CO)C | 0 |
| Decoy657 | P(=O)(CCCC)(CCCC)c1ccccc1OCCOCCOc1ccccc1P(=O)(CCCC)CCCC | 0 |
| Decoy658 | Brc1ccc(OC)cc1CSc1ncc(cn1)C | 0 |
| Decoy659 | s1c(C)c(-c2ccc(cc2C)C)c(C(OC(C)C)=O)c1NC(=O)c1c2CC[C@H](Cc2sc1)C(C)(C)C | 0 |
| Decoy660 | S(=O)([O-])(=Nc1ccc(F)cc1C(=O)[O-])c1c2c([nH+]ccc2)ccc1 | 0 |
| Decoy661 | Brc1sc(C)c(c1)C(=O)c1cc(OC)cc(OC)c1 | 0 |
| Decoy662 | s1cc(nc1-c1ccc(F)cc1)CC(=O)NNC(=O)c1cc(C)c(C)c(S(=O)(=O)NC)c1 | 0 |
| Decoy663 | S1\C(=C/c2oc(cc2)-c2ccccc2)\C(=O)N(C/C(/[O-])=N/c2sc3CCCCc3n2)C1=S | 0 |
| Decoy664 | Clc1c(N=S(=O)([O-])c2sc(cc2)C[NH3+])c(Cl)ccc1C | 0 |
| Decoy665 | S1\C(=C/C(=O)Nc2sc3c(CCCC3)c2C#N)\C(=O)[N-]C1=O | 0 |
| Decoy666 | Brc1ccc(cc1)\C=C\1/Oc2c(cc3c(OC[NH+](C3)CC(C)C)c2C)C/1=O | 0 |
| Decoy667 | Brc1cc(C(=O)n2ncc(C#N)c2N)c(F)cc1 | 0 |
| Decoy668 | Brc1cc(ccc1)[C@@H](n1nc(cc1)C(F)(F)F)[C@@H]([NH3+])CC | 0 |
| Decoy669 | Brc1cc(cc(\C=N\CCNC(=O)c2nonc2N)c1O)C | 0 |
| Decoy670 | Fc1ccccc1[C@@H]1[NH+](CCc2c1[nH]c1c2cccc1)Cc1nc([nH]c1C)CC | 0 |
| Decoy671 | Clc1ccc([N+](=O)[O-])cc1C(=O)Nc1n(nc(c1)-c1occc1)-c1nc(cc([O-])n1)-c1ccccc1 | 0 |
| Decoy672 | s1c2c(nc1N(Cc1cc[nH+]cc1)C(=O)Cc1ccccc1)c(F)cc(F)c2 | 0 |
| Decoy673 | s1c(C)c(C)c([C@H]([NH+]2CCCC2)c2ccccc2)c1NC(=O)c1occc1 | 0 |
| Decoy674 | O=C([O-])[C@H]1N(CCc2[nH]cnc12)C(=O)CCc1[nH]nc2c1CCCC2 | 0 |
| Decoy675 | Brc1cc2nc(n(c2cc1)CC[NH+](CC)C)CCl | 0 |
| Decoy676 | s1cc(nc1\N=C(/[O-])\NC(=O)COCC(=O)[O-])C | 0 |
| Decoy677 | O=C(\C(=C\CCC(C)C)\C)CC[C@@H](CCC[C@@H](CC(=O)[O-])C)C | 0 |
| Decoy678 | Ic1c([nH+]c(nc1NCCC)-c1sccc1)CC | 0 |
| Decoy679 | Ic1ccc(Oc2oc3c(n2)cccc3)cc1 | 0 |
| Decoy680 | Clc1cccc(N=S(=O)([O-])c2cc(N)c(F)cc2Cl)c1F | 0 |
| Decoy681 | ClC1=CC(=CN(CC(=O)N2CCc3[nH]c[nH+]c3C2)C1=O)C(F)(F)F | 0 |
| Decoy682 | S(CCCC)c1nc2n(n1)[C@@H](C(C(OCCCC)=O)=C(N2)C)c1cc(OCC)c(OCCCC)cc1 | 0 |
| Decoy683 | s1cc[nH+]c1N1CCC[C@@H]1CNC(=O)CNS(=O)(=O)c1cc(ccc1)C | 0 |
| Decoy684 | S(=O)(=O)(CC[NH+]1CCc2c(cccc2)[C@H]1Cc1ccccc1)CC | 0 |
| Decoy685 | Brc1cc(cc(OC)c1OC)[C@@H]1C2=C(N=C(C)C1C(OC)=O)C[C@H](CC2=O)c1cc(OC)c(OC)cc1 | 0 |
| Decoy686 | S([C@@H](C[C@@]([NH2+]CCC)(C(=O)[O-])C)C)c1nnc(n1C)C | 0 |
| Decoy687 | s1c2n(nc(c2c(N)c1C(=O)N1C[C@@H](CC[C@H]1C)C)C)C | 0 |
| Decoy688 | OC[C@H]1C[NH+](C[C@H]1C[NH+]1CC[NH+](CC1)CC)Cc1ccc(cc1)C(C)(C)C | 0 |
| Decoy689 | O1[C@H]2[C@H](C=C3[C@@](CCC[C@@H]3C)(C2)C)[C@@H](C[NH+]2CCN(CC2)c2ccc(cc2C)C)C1=O | 0 |
| Decoy690 | s1cc(nc1-c1ncccc1)-c1[nH+]c2c(n1CCC)cccc2 | 0 |
| Decoy691 | s1c2cc(NC(=O)CSc3nc([nH]n3)N\N=C\c3ccc(O)cc3[O-])ccc2nc1SCC | 0 |
| Decoy692 | s1cccc1-c1onc(n1)C[NH+](C(C)C)CCCC(=O)[O-] | 0 |
| Decoy693 | s1ccc(N=S(=O)([O-])[C@@H](C(=S)N)C)c1C(OC)=O | 0 |
| Decoy694 | O=C(N([C@@H](C(=O)NC(CC(C)(C)C)(C)C)c1ccc([N+](=O)[O-])cc1)CCCCCC)CCCC | 0 |
| Decoy695 | Brc1cc(F)c(cc1)-c1[nH]c2ccc(nc2n1)N(C)C | 0 |
| Decoy696 | O1COC=2C(c3c(C(=O)C=2C)c([O-])c(NC(=O)\C(=C\C=C/[C@H]([C@@H](O)[C@@H](C)[C@H]2O[C@@H](O)[C@@H](C)[C@@H](O)[C@H]2C(OC)=O)C)\C)c(C)c3OC(=O)C)=C1/C(=C/C(=O)C)/C | 0 |
| Decoy697 | Brc1ccc(nc1)C1=Nc2n(ncc2C2CC2)C(Cl)=C1 | 0 |
| Decoy698 | s1cc(cc1CN=S(=O)([O-])N(C)C)\C=C\C(=O)[O-] | 0 |
| Decoy699 | Clc1ccc(Cl)cc1C(=O)N(Cc1cccnc1)c1sc2c(n1)c(F)cc(F)c2 | 0 |
| Decoy700 | s1cc(nc1C/1=Cc2c(O\C\1=[NH+]/c1sccn1)cccc2)-c1ccccc1 | 0 |
| Decoy701 | Brc1c(n(nc1CC)CC)COc1cc2CCCc2cc1 | 0 |
| Decoy702 | O(C(C)(C)C)c1nc(NC2C[C@@H](C[C@@H](C2)C)C)ccc1N | 0 |
| Decoy703 | Brc1cccnc1[C@H](N[NH3+])c1cc(OC(F)(F)F)ccc1 | 0 |
| Decoy704 | Brc1cc(C(=O)N2Cc3c(nn(C(C)(C)C)c3-n3cccc3)C2)c(Cl)cc1 | 0 |
| Decoy705 | [NH+]1(CCCC[C@@H]1c1ccc(nc1)N(CC)CC)CCCC | 0 |
| Decoy706 | Brc1cc2cc(\C=C(\C#N)/c3scc(n3)-c3ccc(cc3C)C)c(Cl)nc2cc1 | 0 |
| Decoy707 | Brc1ccc(Br)cc1[C@@H]([NH2+]C)c1cc(C)c(cc1)C | 0 |
| Decoy708 | S(=O)(=O)(Nc1ccc(cc1)C(=S)N)c1c(n[nH]c1C)C | 0 |
| Decoy709 | FC(F)(F)c1cc(Oc2c3c(nccc3C)c(NCCC[NH3+])cc2OC)ccc1 | 0 |
| Decoy710 | s1c2c(cc1)[C@@H](N(CC2)C(=O)[C@@H]1OCC[NH2+]C1)C1CC1 | 0 |
| Decoy711 | O(C(=O)C(O)(c1ccccc1)c1ccccc1)CC(=O)NC(=O)NCCC | 0 |
| Decoy712 | Clc1ccc2nsnc2c1N[C@@H](C)c1cc(F)c(F)cc1 | 0 |
| Decoy713 | S([C@@H](C[C@@]([NH2+]C1CC1)(C(=O)[O-])C)C)c1nncn1C | 0 |
| Decoy714 | O=[N+]([O-])/N=C(\N=C\c1c(nn(c1[O-])-c1cc(C)c(cc1)C)C)/[NH-] | 0 |
| Decoy715 | Clc1cc(ccc1F)CN=S(=O)([O-])N(CCC(=O)[O-])C | 0 |
| Decoy716 | Brc1cc(sc1)Cn1c2c(nc1CCCl)c[nH+]cc2 | 0 |
| Decoy717 | s1cc(nc1-c1ccc(cc1)C)[C@H]1NC(=O)c2n[nH]c(c2N1)C | 0 |
| Decoy718 | Clc1ccccc1S(=O)(=O)NCCNc1nc([nH+]c(N2CCCC2)c1)C | 0 |
| Decoy719 | Clc1nc(SC)nc(NN)c1Cc1ccc(OC(C)C)cc1 | 0 |
| Decoy720 | s1c(nc(C)c1C(=O)Nc1cc(F)cc(F)c1)C1=C(C)C(=NN(C)C1=O)C | 0 |
| Decoy721 | O=C1NC(=O)NC(C(=O)N2Cc3c(N(C[C@H]2CC)C)cccc3)=C1N | 0 |
| Decoy722 | Brc1ccc(cc1)C(=O)N([C@H]1C=CS(=O)(=O)C1)c1ccc(cc1)C(C)C | 0 |
| Decoy723 | S(CC(=O)N)c1[nH+]c(c2c(CCC2)c1C#N)-c1ccc(cc1)C(C)(C)C | 0 |
| Decoy724 | S([C@@H]1C[C@@]([NH2+]CC)(CCC1)C(=O)N)C=1NC(=CC(=O)N=1)C | 0 |
| Decoy725 | Clc1cc(N2[C@H](C)C(=O)N[C@H](C(C)(C)C)C2=O)cc(Cl)c1 | 0 |
| Decoy726 | O(C(=O)C[n+]1cc(ccc1)C(=O)Nc1ccccc1)CCCCCCCCCCCC | 0 |
| Decoy727 | O=C(Nc1ccccc1-c1ccncc1)N[C@H]1CCc2[nH]ncc2C1 | 0 |
| Decoy728 | FC(F)(F)Oc1cc(ccc1)-c1cc(-n2c[nH+]cc2C(=O)N(CCCC)CC)ccc1 | 0 |
| Decoy729 | Ic1c(nc(nc1Cl)-c1oc(cc1)[C@H]1C[C@@H]1C)C1CC1 | 0 |
| Decoy730 | S=C(Nc1cccc(OC)c1OC)N(Cc1ccccc1OCC)Cc1occc1 | 0 |
| Decoy731 | BrC12CC3(C[C@@H](C1)C[C@H](C3)C2)C(OCc1ccccc1C#N)=O | 0 |
| Decoy732 | S(=O)([O-])(=N[C@@H]1C=CS(=O)(=O)C1)c1n[nH]cc1C(=O)[O-] | 0 |
| Decoy733 | Clc1ccccc1N([C@@H](\C=C\CCC)C(=O)NCCCC)C(=O)[C@H](CCCC)CC | 0 |
| Decoy734 | s1c(C(=O)C)c(nc1N1[C@@H](C(C(=O)c2n3c(nc2C)C=CC=C3)=C([O-])C1=O)c1ccccc1F)C | 0 |
| Decoy735 | S(=O)([O-])(=N[N-]\C(=N/S(=O)(=O)c1ccc(cc1)C)\c1ccccc1)c1ccccc1 | 0 |
| Decoy736 | O1CCC[C@H]1C[NH+](Cc1ccncc1)C[C@H]1C=NN=C1c1cc(C)c(cc1)C | 0 |
| Decoy737 | s1cc(nc1N(C(=O)C)C)\C=C\C(=O)c1ccc(OCCCCC)cc1 | 0 |
| Decoy738 | S(=O)(=O)(CC1CC1)c1ncc(n1CCOC)C[NH+]1[C@H](CC(=C[C@@H]1CC=C)C)CC=C | 0 |
| Decoy739 | Brc1ccc(cc1)[C@@]([NH3+])(Cc1n(nc(CC)c1Cl)CC)C | 0 |
| Decoy740 | S=C(Nc1cccc(C)c1C)N(Cc1ccc(OCC)cc1)Cc1ccc[nH+]c1 | 0 |
| Decoy741 | S(Cc1cccc(F)c1F)C1=Nc2c(cccc2)C(=O)N1CC=C | 0 |
| Decoy742 | S=C(Nc1ccc(cc1)C)N(Cc1ccc([NH+](CC)CC)cc1)Cc1occc1 | 0 |
| Decoy743 | Fc1cc(OC)c(OC)cc1[C@H](NC(=O)CCNC(=O)C(C)(C)C)C | 0 |
| Decoy744 | S1CC[C@@H](NC(=O)C=2C(=O)NC(=CC=2C)C)c2cc(F)ccc12 | 0 |
| Decoy745 | S(C[C@H](CCCC)CC)c1nnnn1CC[NH2+]CCOC | 0 |
| Decoy746 | Fc1ccccc1\C=C/C(OCC(=O)C12CC3CC(C1)CC(C2)C3)=O | 0 |
| Decoy747 | O=C1N(CCC(=O)[O-])C(=O)[C@H]2[C@H]1C1[C@H]3[C@H](C2C=C1)C(=O)N(CCC(=O)[O-])C3=O | 0 |
| Decoy748 | O1[C@H](CO)[C@H](O)[C@H](O[C@@H]2O[C@H](CO)[C@@H](O)[C@H](O)[C@H]2NC(=O)C)[C@@H](NC(=O)C)[C@H]1Oc1ccc([N+](=O)[O-])cc1 | 0 |
| Decoy749 | S=C(NC(=O)c1c(noc1C)-c1ccccc1)[N-]NC(=O)c1n[nH]c(c1)-c1occc1 | 0 |
| Decoy750 | O1[C@@H](CO)[C@@H](O)[C@@H](O)[C@@H](O)[C@H]1OC(=O)[C@]1([C@H]2[C@]3([C@@]4([C@H](CC2)[C@]2(CC[C@@H]([C@H](C[C@@H](O)[C@H](O)C(O)(C)C)C)[C@]2(CC4)C)C)C3)[C@H](O)C[C@@H]1O)C | 0 |
| Decoy751 | S(=O)([O-])(=Nc1cc(ccc1)C(=O)NCCCO)c1cc(F)c(cc1)C | 0 |
| Decoy752 | Brc1cc(Br)cc(N)c1N=S(=O)([O-])CCC | 0 |
| Decoy753 | FC(F)(F)c1cc(ccc1)-c1nn(c2c1C[NH+](CC2)C)Cc1ccccc1 | 0 |
| Decoy754 | IC1=CNC(=NC1=O)c1ncccn1 | 0 |
| Decoy755 | S1(=O)(=O)N=C([N-][C@@H](CCCC)C(=O)NNC(=O)[C@@H](Oc2ccccc2F)C)c2c1cccc2 | 0 |
| Decoy756 | S(=O)([O-])(=Nc1n(ncc1C(=O)[O-])C)c1[nH]cnc1 | 0 |
| Decoy757 | O=C(N([C@H](CCCCC)C(=O)NC1CCCCC1)CCCCCC)[C@H](CCCC)CC | 0 |
| Decoy758 | S(c1c2ncccc2ccc1)c1oc(cc1)\C=[NH+]\c1cc(ccc1)C(=O)C | 0 |
| Decoy759 | Fc1ccc(cc1)[C@H](Nc1ccccc1C(F)(F)F)c1ccc2c([nH+]ccc2)c1O | 0 |
| Decoy760 | O=C([O-])c1ccccc1NC(=O)C[NH+](CCN(CC(=O)[O-])CC(=O)[O-])CC(=O)[O-] | 0 |
| Decoy761 | Clc1ccccc1[C@H]1C(C(OCC)=O)=C(NC(C)=C1C(OC)=O)COCC[NH2+][C@@H]1O[C@H](CO)[C@@H](O[C@@H]2O[C@H](CO)[C@H](O)[C@H](O)[C@H]2O)[C@H](O)[C@H]1O | 0 |
| Decoy762 | s1cc(c2c1nc(nc2N)-c1ccsc1)-c1ccccc1 | 0 |
| Decoy763 | O(C(=O)[C@@]1(CCC[NH+](C1)Cc1nccn1CC)CCc1ccccc1)CC | 0 |
| Decoy764 | s1c(ccc1[C@@H](Nc1cc(ccc1F)C(=O)[O-])C)CC | 0 |
| Decoy765 | Clc1ccc(cc1)-c1nnc(SCc2[nH+]c3n(c2)C=CC=C3C)n1CC | 0 |
| Decoy766 | Brc1cccnc1SCC[C@@]([NH2+]CC)(C(=O)[O-])C | 0 |
| Decoy767 | O(C[C@H]1CCCN(C1)c1nc([nH+]cc1C)N1C[C@H](CCC1)COCC)CC | 0 |
| Decoy768 | Brc1sc(cc1)C(=O)N1CCCN(CC1)c1ncc(cc1)C(F)(F)F | 0 |
| Decoy769 | Brc1ccc(cc1)-c1noc(C)c1C(=O)N1CCN(CC1)c1ccc(Cl)cc1 | 0 |
| Decoy770 | Brc1cc(Br)cc(C[NH2+][C@@H](CCC)COC)c1OC | 0 |
| Decoy771 | O=C1N2C=C(C=CC2=NC(=C1)C[NH2+][C@H](C(C)C)c1ccc(cc1)C1CCCCC1)C | 0 |
| Decoy772 | O1[C@@H](CC[C@H]1[C@H](C(=O)[O-])C)C[C@@H](OC(=O)[C@H](C)[C@H]1O[C@H](CC1)C[C@@H](O)CC)CC | 0 |
| Decoy773 | Brc1c(n(nc1C)CC)C[C@]([NH3+])(C)c1cc(C)c(cc1)C | 0 |
| Decoy774 | [NH2+](C\C=C\c1ccc(cc1)C#N)C1C[C@@H]2[NH+]([C@H](C1)CC2)Cc1ccccc1 | 0 |
| Decoy775 | Brc1ccccc1-c1oc(nn1)-c1ccccc1Br | 0 |
| Decoy776 | S1(=O)(=O)CCN(C(=O)N[C@@H]2CC[NH+](C2)CC(F)(F)F)[C@@H](C)[C@@H]1C | 0 |
| Decoy777 | Fc1c(F)cc(-n2nc([C@H](CC=C)C)c(c2)C=O)cc1F | 0 |
| Decoy778 | S=C(N)c1c(C)c(nnc1N[C@H](C)c1ccc(cc1)C)C | 0 |
| Decoy779 | Brc1cc([C@H]2Nc3c([C@@H]4[C@@H]2CC=C4)cc(N(C(=O)C)C)cc3)c(O)cc1 | 0 |
| Decoy780 | s1cccc1C1=NN2[C@H](C1)c1c(O[C@@H]2c2ccncc2)c(OC)ccc1 | 0 |
| Decoy781 | Brc1ccc(cc1)C(=O)N([C@H]1C=CS(=O)(=O)C1)c1ccc(cc1)C(C)C | 0 |
| Decoy782 | Brc1cc(sc1)C[C@H]([NH2+]C)C1(N(CC)CC)CCCC1 | 0 |
| Decoy783 | Brc1cc(-n2c3ncc(Br)cc3nc2CCl)c(F)cc1 | 0 |
| Decoy784 | Brc1cc(F)c(cc1)-c1[nH]c2ccc(nc2n1)N(C)C | 0 |
| Decoy785 | S1CCC([NH+]2C[C@@H](CCC2)C2=NN=C[C@@H]2Cc2ccccc2)CC1 | 0 |
| Decoy786 | S(=O)([O-])(=Nc1cc(cc(c1)C(=O)[O-])C(=O)[O-])c1cc2C[C@H](N(c2cc1)C(=O)CC)C | 0 |
| Decoy787 | O=C1CC[C@H](O)C([O-])=C1C[C@H]1[C@](O)(CC[C@@H]1/C(=C/CC=C(C)C)/C)C | 0 |
| Decoy788 | FC(F)(F)c1cc(ccc1)CC(=O)N\N=C\c1cc(n(c1C)-c1ccccc1CC)C | 0 |
| Decoy789 | Brc1cc(S(=O)([O-])=Nc2scnn2)c(nc1)NCCC | 0 |
| Decoy790 | ClCc1[nH+]c2c(n1[C@H](C[C@@H](CC)C)C)c(F)c(F)cc2 | 0 |
| Decoy791 | O=C1NC(=CC([O-])=N1)C(=O)NC[C@@H](CC(C)C)CC(=O)[O-] | 0 |
| Decoy792 | s1c2CCCCc2c2c1nc(nc2[O-])C[NH+](CC=C)C[C@H](O)C[NH+]1CCOCC1 | 0 |

**Table S2.** Structures of the 330 Compounds (in SMILE format) from the test set together with their activities (1 or 0) for glutamate-induced models.

| ChEMBL ID | Structure | Activity |
| --- | --- | --- |
| CHEMBL100367 | S(c1ccc(cc1C(F)(F)F)\C=C\C(=O)N1CC(CCC1)C(=O)[O-])c1cc2OCCOc2cc1 | 1 |
| CHEMBL116903 | O1[C@H](CO)[C@@H](O)[C@@H](O)[C@@H]1n1c2ncnc(N[C@@H]3CCC[C@H]3O)c2nc1 | 1 |
| CHEMBL119709 | Ic1cc(ccc1)CNc1ncnc2n(cnc12)[C@@H]1O[C@H](C(=O)NC)[C@@H](O)[C@H]1O | 1 |
| CHEMBL1254343 | O1[C@H](C)[C@H]([NH3+])CO[C@@H]1C[C@H]1[NH2+]CCc2c1[nH]c1c2cc(OC)cc1 | 1 |
| CHEMBL1294 | O(C)c1cc2c(nccc2[C@H](O)[C@@H]2[NH+]3C[C@@H]([C@H](C2)CC3)C=C)cc1 | 1 |
| CHEMBL163316 | O1c2c(C(=O)C(OC)=C1c1cc(O)c(O)cc1)c(O)cc(O)c2 | 1 |
| CHEMBL1760959 | Brc1cc2c(OC(CC23OC(=O)N(C3)Cc2ccc(NC(=O)C)cc2)(C)C)cc1 | 1 |
| CHEMBL1946837 | Fc1cc(F)ccc1Nc1nc2c(nc(nc2)NC2CCC([NH3+])CC2)n1C1CCCC1 | 1 |
| CHEMBL1951821 | O1c2c(ccc3c2cccc3)C(=O)C(O)=C1c1cc(O)c(O)cc1 | 1 |
| CHEMBL1951828 | O1c2c(ccc3c2cccc3)C(=O)C(O)=C1c1cc(OC)c(O)cc1 | 1 |
| CHEMBL1951829 | O1c2c(ccc3c2cccc3)C(=O)C=C1c1cc(OC)c(O)cc1 | 1 |
| CHEMBL1951833 | O1c2c(ccc3c2cccc3)C(=O)C(O)=C1c1ccc(N2CCCC2)cc1 | 1 |
| CHEMBL1951841 | O1c2c(ccc3c2cccc3)C(=O)C(O)=C1c1cc(O)c(N2CCCC2)cc1 | 1 |
| CHEMBL1951842 | O1c2c(cc(C)c(c2)C)C(=O)C(O)=C1c1cc(O)c(N2CCCC2)cc1 | 1 |
| CHEMBL2028973 | O(CC(=O)N[C@@H](CCCNC(=[NH2+])N)C(=O)[O-])c1ccc(cc1)C1=[N+]([O-])C(C)(C)C(N1O)(C)C | 1 |
| CHEMBL2047750 | O([N+](=O)[O-])C[C@H](O[N+](=O)[O-])CNC(=O)[C@@H](NC(=O)CC[NH3+])Cc1nc[nH]c1 | 1 |
| CHEMBL2112564 | Clc1nc(N[C@@H](CSc2sc3c(n2)cccc3)C)c2ncn(c2n1)[C@@H]1O[C@H](CO)[C@@H](O)[C@H]1O | 1 |
| CHEMBL2144409 | O1CCN(CC1)c1cc(ccc1)-c1nc(ncc1)Nc1ccc(-n2nc(nc2)-c2ccc(nc2)C)cc1 | 1 |
| CHEMBL229125 | O=C1CC[NH+](C1)[C@@H]1CCCC[C@H]1OCCc1c2c(ccc1)cccc2 | 1 |
| CHEMBL229281 | O1CC[NH+](CC1)[C@@H]1CCCC[C@H]1OCCc1cc2c(cc1)cccc2 | 1 |
| CHEMBL229355 | O(CCc1cc2c(cc1)cccc2)[C@@H]1CCCC[C@H]1[NH+]1CCN(CC1)C(=O)C | 1 |
| CHEMBL229828 | Brc1ccccc1CCO[C@@H]1CCCC[C@H]1[NH+]1CCOCC1 | 1 |
| CHEMBL229853 | O=C1CC[NH+](C1)[C@@H]1CCCC[C@H]1OCCCC1CCCCC1 | 1 |
| CHEMBL2391546 | ICC(O)(C)[C@@H]1Oc2c(C1)c1O[C@H]3[C@H](c4cc(OC)c(OC)cc4OC3)C(=O)c1cc2 | 1 |
| CHEMBL257088 | Clc1ccc(Oc2ccc(S(=O)(=O)N(C)C3=CC=CN([O-])C3=O)cc2)cc1 | 1 |
| CHEMBL288365 | S(=O)(=O)(N[C@@H](C(C)C)C(=O)N[C@@H](CC(C)C)C=O)c1ccc(F)cc1 | 1 |
| CHEMBL3099549 | O=C(N[C@@H](CCC(=O)[O-])C(=O)N[C@H](C(=O)N[C@@H](CCC(=O)[O-])C(=O)N[C@H](C(=O)[O-])C)C)C | 1 |
| CHEMBL3099551 | S1SCC[C@H]1CCCCC(=O)N[C@@H](CCC(=O)[O-])C(=O)N[C@H](C(=O)[O-])C | 1 |
| CHEMBL3099555 | S1SCC[C@H]1CCCCC(=O)N[C@@H](CCC(=O)[O-])C(=O)NCCS(=O)(=O)[O-] | 1 |
| CHEMBL3099556 | S1SCC[C@H]1CCCCC(=O)N[C@H](C(=O)N[C@@H](CCC(=O)[O-])C(=O)[O-])C | 1 |
| CHEMBL3099562 | S1SCC[C@H]1CCCCC(=O)N(CC(=O)[O-])CC(=O)[O-] | 1 |
| CHEMBL3219851 | O1c2c(cccc2)C(=O)C(OC(=O)CCCCC(=O)[O-])=C1c1cc(O)c(O)cc1 | 1 |
| CHEMBL326838 | Clc1nc(N[C@@H](COc2ccccc2)C)c2ncn(c2n1)[C@@H]1O[C@H](CO)[C@@H](O)[C@H]1O | 1 |
| CHEMBL331382 | O1[C@H](CO)[C@@H](O)[C@@H](O)[C@@H]1n1c2ncnc(NCc3ccccc3C)c2nc1 | 1 |
| CHEMBL3322887 | O1c2cc(OC)c-3c(c2OC1)[C@@H](N(c1c-3cc(OC)c2cc(OC)c(OC)cc12)C)CC(=O)C | 1 |
| CHEMBL338753 | Clc1cc(Cl)cc2NC(C\C(\c12)=C/C(=O)Nc1ccc(NC(=O)C)cc1)C(=O)[O-] | 1 |
| CHEMBL3667032 | O(CC(OC)=O)c1ccc(cc1OC)\C=C\C(=O)CC(=O)\C=C\c1cc(OC)c(O)cc1 | 1 |
| CHEMBL3735205 | O(CC([NH2+]Cc1cc2c(cc1)C([NH2+]C2(C)C)(C)C)C)c1c(cccc1C)C | 1 |
| CHEMBL3792609 | Clc1cc(Cl)cc(CNc2cc(O)c(cc2)C(OC)=O)c1O | 1 |
| CHEMBL3805744 | Fc1ccc(cc1)-c1nc(C)c(cc1)C(=O)N\N=C/1\c2c(N(C)C\1=O)cccc2 | 1 |
| CHEMBL397881 | O(Cc1ccccc1)c1ccc(cc1OC)\C=[N+](\[O-])/C(C)(C)C | 1 |
| CHEMBL398669 | O1[C@H](CO)[C@@H](O)[C@@H](O)[C@@H]1n1c2ncnc(Nc3ccc(cc3)CCNC(=O)c3cc(C(C)(C)C)c(O)c(c3)C(C)(C)C)c2nc1 | 1 |
| CHEMBL399774 | O1[C@H](CO)[C@@H](O)[C@@H](O)[C@@H]1n1c2ncnc(NCC3CC(N(O)C3(C)C)(C)C)c2nc1 | 1 |
| CHEMBL400746 | O1[C@H](CO)[C@@H](O)[C@@H](O)[C@@H]1n1c2ncnc(Nc3ccc(cc3)CCNC(=O)c3cc4c(cc3)C(N(O)C4(C)C)(C)C)c2nc1 | 1 |
| CHEMBL41003 | O1C(C(O)CO)C(OCCCCCCCCCCCCCCCCCC)=C(O)C1=O | 1 |
| CHEMBL41306 | Clc1ccc(SC)cc1NC(=[NH2+])N(C)c1cc(SC)ccc1 | 1 |
| CHEMBL426744 | SC[C@H](NC(=O)[C@H]1N(CCC1)C(=O)[C@H](C)c1ccc(cc1)CC(C)C)C(OCC)=O | 1 |
| CHEMBL450656 | Oc1ccc(cc1)C[C@@H](NC(=O)C)C(=O)N1CCC[C@@H]1C(=O)N1C[C@@H](O)C[C@@H]1C(=O)NCC(=O)N[C@@H](C(=O)NCC(=O)N)C | 1 |
| CHEMBL455939 | Brc1cc2c(OC(CC23OCC(=O)N(C3)Cc2ccccc2)(C)C)cc1 | 1 |
| CHEMBL458304 | Brc1ccc(cc1)C[NH+]1CC2(OCC1)CC(Oc1c2cc(Br)cc1)(C)C | 1 |
| CHEMBL463980 | O1c2c(cccc2)C2(OCC(=O)N(C2)Cc2ccc(N)cc2)CC1(C)C | 1 |
| CHEMBL465952 | Clc1cccc(Cl)c1C(=O)NCc1cc2OCOc2cc1 | 1 |
| CHEMBL475228 | O1c2c(N(CC1(C)C)C(=O)C[NH+](CC)CC)c(cc(C)c2C)C | 1 |
| CHEMBL475235 | Clc1c(C)c(c2O[C@@H](CN(c2c1C)C(=O)C[NH+](CC)CC)c1ccccc1)C | 1 |
| CHEMBL496670 | O(C(=O)C(NC(=O)\C=C(\C=C\C=C(\C=C\C=1C(CCCC=1C)(C)C)/C)/C)CO)CC | 1 |
| CHEMBL496686 | O(C(=O)C(NC(=O)[C@@]1(C2CC=C3[C@@H](CCC(=C3)C(C)C)[C@]2(CCC1)C)C)CO)CC | 1 |
| CHEMBL501313 | Clc1c(C)c(c2OC(CN(c2c1C)C(=O)C[NH+](CC)CC)(C)C)C | 1 |
| CHEMBL511058 | S1C[C@H]([NH2+]C[C@@H](CSc2ccccc2OC)C)COc2c1cccc2 | 1 |
| CHEMBL514423 | Brc1ccc(cc1)CN1CC2(OCC1=O)CC(Oc1c2cc(Br)cc1)(C)C | 1 |
| CHEMBL516980 | O1c2c(cccc2)C2(OCC[NH+](C2)Cc2ccc(cc2)C)CC1(C)C | 1 |
| CHEMBL525390 | O1c2c(cccc2)C2(OCC[NH+](C2)Cc2ccc(OC)cc2)CC1(C)C | 1 |
| CHEMBL564175 | S1C[C@H]([NH2+]C[C@@H](CSc2cccc(F)c2O)C)COc2c1cccc2 | 1 |
| CHEMBL575803 | O1C2(CC(Oc3c2cccc3)(C)C)C(=N)N(Cc2ccc(NC(=O)C)cc2)C1=O | 1 |
| CHEMBL577782 | S(=O)(=O)(Nc1ccc(cc1)CN1CC2(OC1=O)CC(Oc1c2cccc1)(C)C)C | 1 |
| CHEMBL90286 | O1c2c(ccc(O)c2)C(=O)C=C1c1cc(C(C)(C)C)c(O)c(c1)C(C)(C)C | 1 |
| CHEMBL90863 | O1c2c(ccc(O)c2)C(=O)/C(=C\c2cc(C(C)(C)C)c(O)c(c2)C(C)(C)C)/C1c1cc(C(C)(C)C)c(O)c(c1)C(C)(C)C | 1 |
| Decoy001 | S1C(=NN(C1C(O)C(O)C(O)CO)c1ccccc1)c1ccccc1 | 0 |
| Decoy002 | s1nc2c(n1)cccc2S(=O)([O-])=N[C@H](C(=O)N1CCN(CC1)c1ccccc1)c1ccccc1 | 0 |
| Decoy003 | Clc1ccc([N+](=O)[O-])cc1C(=O)Nc1n(nc(c1)-c1occc1)-c1nc(cc([O-])n1)-c1ccccc1 | 0 |
| Decoy004 | o1cccc1CNC(=O)CN1N=C(c2c(n(nc2)-c2cc(C)c(cc2)C)C1=O)C(C)C | 0 |
| Decoy005 | Clc1ccccc1Cn1c2c(nc1[C@H]1CC(=O)N(C1)c1ccc(Cl)cc1)cccc2 | 0 |
| Decoy006 | Clc1ccccc1OCCCn1c2c([nH+]c1-c1sccc1)cccc2 | 0 |
| Decoy007 | Fc1cc(F)ccc1-c1n[nH]cc1C[NH2+]Cc1c(n(nc1C)-c1ccc(F)cc1)C | 0 |
| Decoy008 | Clc1cc2OC(=O)N(c2cc1)CCC(OCC=1N=C2SC=3CCCCC=3N2C(=O)C=1)=O | 0 |
| Decoy009 | o1nc(-c2ccccc2)c(C[NH+](CC2CC2)C[C@H](O)COC(=O)CCC)c1N(CC)CC | 0 |
| Decoy010 | s1cccc1CN([C@@H]1OC(=O)c2c1cccc2)c1ccc(OC)cc1 | 0 |
| Decoy011 | s1c2CCCc2c2c1nc(nc2[O-])CSc1ncc(S(=O)(=O)N2CCCCCC2)cc1 | 0 |
| Decoy012 | s1c(ncc1C[NH+](C)[C@@H]1[C@@H]2[C@H](OCC2)C1(C)C)-c1sccc1 | 0 |
| Decoy013 | [nH+]1cnc(N(Cc2ccccc2)Cc2ccccc2)c(N)c1Nc1ccc(cc1)C | 0 |
| Decoy014 | FC(F)(F)c1cc(ccc1)-c1nn(c2c1C[NH+](CC2)C)Cc1ccccc1 | 0 |
| Decoy015 | FC(F)(F)C[NH+]1CCC(CC1)C(O[C@H]1C[C@@H]2C[C@H]([C@@H]1C)C2(C)C)=O | 0 |
| Decoy016 | S1C=Cn2cc(nc12)C(=O)N1C[C@@H](CCC1)C(=O)c1cc(F)c(cc1)-c1ccccc1 | 0 |
| Decoy017 | Ic1cc2[nH+]c(n(c2cc1)C[C@H]1CCOC1)[C@H](Cl)C | 0 |
| Decoy018 | Brc1cc(cc(OC)c1[O-])[C@@H]1N(C(=O)C([O-])=C1C(=O)c1cc2OCCOc2cc1)c1sc(C(OC)=O)c(n1)C | 0 |
| Decoy019 | Ic1c(nc(nc1Cl)-c1cc(F)cc(Br)c1)CCC | 0 |
| Decoy020 | Brc1cc(CCl)c(F)c(S(=O)(=O)N(C)C2CCCC2)c1 | 0 |
| Decoy021 | [NH+]1(CCC(CC1)c1nc2n(n1)C=C(C=C2)c1cc(ccc1)C)CC1CC1 | 0 |
| Decoy022 | s1cccc1[C@@H]\1N(CCCn2cc[nH+]c2)C(=O)C(=O)/C/1=C(/[O-])\c1ccccc1 | 0 |
| Decoy023 | O=C1N(c2c(cccc2)[C@@]12[C@@H]1C(=CC[C@@H](C1)C)C(C#N)=C(N)C2(C#N)C#N)CCc1ccccc1 | 0 |
| Decoy024 | O=C1C2=C(Nc3c(N[C@H]2c2ccncc2)cccc3)C[C@@H](C1)c1ccc(N(C)C)cc1 | 0 |
| Decoy025 | S(C[C@H](CS)c1ccccc1)c1n2CCCCCc2nn1 | 0 |
| Decoy026 | Fc1c(C[NH+]2C[C@@H](CCC2)C2=NN=C[C@H]2c2cc(F)ccc2)c(F)ccc1F | 0 |
| Decoy027 | Brc1cc([N+](=O)[O-])ccc1N[C@](C(=O)[O-])(C(F)(F)F)C | 0 |
| Decoy028 | S1CC[NH+](CC12CCCCC2)Cc1n2c(nc1)N=C(C=C2C)C | 0 |
| Decoy029 | S(=O)([O-])(N(C)C1CCCCC1)=N[C@@](C(=O)[O-])(C(F)(F)F)C | 0 |
| Decoy030 | O=C1N=C([O-])[C@@H](N1)Cc1nc(nn1CC(=O)[O-])C1CCCC1 | 0 |
| Decoy031 | O=C(N1CCCCCC1)CCCc1nc2cccnc2n1CCC(C)C | 0 |
| Decoy032 | O(C(C)C)c1ccc(cc1)C(=O)[C@H]1[C@H](N(CCCn2cc[nH+]c2)C(=O)C1=O)c1ccc(cc1)C | 0 |
| Decoy033 | O1[C@](O[C@@H]2O[C@H](COC(=O)Cc3c4c([nH]c3)cccc4)[C@@H](O)[C@@H](O)[C@@H]2O)(CO)[C@H](O)[C@H](O)[C@@H]1CO | 0 |
| Decoy034 | Clc1ccc(cc1)-c1scc(n1)CN([C@@H](C)c1[nH+]cccc1)C | 0 |
| Decoy035 | Brc1cc(F)ccc1N=S(=O)([O-])c1c(C)c(ccc1N)C | 0 |
| Decoy036 | Clc1ccccc1[C@@H]1C(C(OCC)=O)=C(NC(C)=C1C(OC)=O)COCC[NH2+][C@@H]1O[C@H](CO)[C@@H](O[C@@H]2O[C@H](CO)[C@H](O)[C@H](O)[C@H]2O)[C@H](O)[C@H]1O | 0 |
| Decoy037 | S1[C@@H]2C[C@H]3[C@@H]4[C@@H](CC[C@]3(C)[C@@]2(OCCCC)[NH+]=C1N)[C@@]1([C@H](CCCC1)CC4)C | 0 |
| Decoy038 | Clc1ccc(cc1)-c1[nH+]c2n(C=C(C=C2)c2cc(ccc2)C)c1CCC(=O)NC(C)C | 0 |
| Decoy039 | Brc1ccccc1[C@@H](SCC[NH+](CC)CC)[C@H]([NH3+])C | 0 |
| Decoy040 | Clc1c(nn(c1N)-c1ncc(cc1Cl)C(F)(F)F)C(C)C | 0 |
| Decoy041 | s1c2c(CC(OC2)(C)C)c(C(=O)N)c1NC(=O)C(=O)NC(C)C | 0 |
| Decoy042 | Clc1cnc(Cl)nc1N(Cc1sc(Cl)cc1)C | 0 |
| Decoy043 | Clc1ccc(cc1)C(=O)C/C(=C\C(=C/1\SC(=NC\1=O)N1CCCC1)\c1ccc(Cl)cc1)/c1ccccc1 | 0 |
| Decoy044 | s1c(ccc1C)C(=O)/C(/[n+]1cc([O-])ccc1C)=C(/[S-])\NC1CCCCC1 | 0 |
| Decoy045 | O=C(N([C@H](CCCCC)C(=O)NC(CC(C)(C)C)(C)C)C(C)(C)C)[C@H](CCCC)CC | 0 |
| Decoy046 | s1c2cc(ccc2nc1N1[C@H]([C@H](C(=O)c2occc2)C(=O)C1=O)c1ccc(cc1)C(=O)[O-])C(=O)[O-] | 0 |
| Decoy047 | s1c2c(nc1[C@H]1CC=CC[C@H]1C(=O)NCCCO[C@@H](C)c1ccccc1)cccc2 | 0 |
| Decoy048 | S(=O)(=O)(N1Cc2c3[C@@H]([NH+](CCc3ccc2)C)C1)c1ccc(F)cc1 | 0 |
| Decoy049 | Brc1cc2NC(=O)Nc2cc1NC(=O)[C@H](C[NH2+]C)C | 0 |
| Decoy050 | Brc1cc(C)c(N2C3=C([C@H](C(C#N)=C2n2cccc2)c2cc(F)ccc2)C(=O)CCC3)cc1 | 0 |
| Decoy051 | S(=O)(=O)(Nc1ccc(cc1)C(=O)NC1CCCCCC1)\C=C/c1ccccc1 | 0 |
| Decoy052 | Clc1c(Cl)c(Cl)ccc1[C@H](N[C@H]1CC[NH+](C1)C1CC1)C | 0 |
| Decoy053 | Brc1c(n(nc1C)C)C[NH+](Cc1ccc(N)cc1)CCC | 0 |
| Decoy054 | s1cc(nc1[C@H](N1C(=O)c2ccsc2NC1=S)C)C | 0 |
| Decoy055 | O=C(NCCCCC/C(/[O-])=N/c1[nH]nc(n1)-c1ccc(cc1)C(C)(C)C)C | 0 |
| Decoy056 | O=C/1Nc2c(cccc2)\C\1=N/[N-]c1[nH+]c2N(C)C(=O)N(C)C(=O)c2n1C[C@@H](O)COC(C)C | 0 |
| Decoy057 | Brc1cc(-c2nc(nc(Cl)n2)-n2ccnc2)c(F)cc1 | 0 |
| Decoy058 | Clc1ccc(cc1)[C@@H]1OC(=O)C2(CCCCC2)C(=O)[C@@H]1C | 0 |
| Decoy059 | o1cc(cc1)-c1cc(ccc1)C(=O)N[C@@H](CC(=O)N)C1CCCCC1 | 0 |
| Decoy060 | s1c2c(S[C@H]3[C@@H]([C@@H]2c2cc([N+](=O)[O-])ccc2[O-])C(=O)N(c2ccc(OCC)cc2)C3=O)nc1[S-] | 0 |
| Decoy061 | S1[C@H]2N(C(C(=O)[O-])=C(C1)CSc1nnnn1C)C(=O)[C@@H]2NC(=O)Cn1nc(cc1C1CC1)C(F)(F)F | 0 |
| Decoy062 | s1cc(nc1N=S(=O)([O-])CC)-c1ccc(F)cc1N | 0 |
| Decoy063 | s1cc(nc1-c1ccc(cc1)C)COc1ccc(N2CCCC2=O)cc1 | 0 |
| Decoy064 | O=C(N[C@H]1CCC[C@@H](C)[C@H]1C)[C@H](N1C[C@H](C)[C@H]([NH+](C)C)C1)C | 0 |
| Decoy065 | S(CC[C@H]([NH+](CCCc1onc(n1)-c1cc(ccc1)C(F)(F)F)C)C)C | 0 |
| Decoy066 | S(=O)([O-])(=N[C@@H](C(OC\C=C\c1ccccc1)=O)C)c1cn(nc1)C | 0 |
| Decoy067 | Brc1ccsc1CN1C[C@]([NH2+]C[C@@H]1C(C)C)(C)C1CC1 | 0 |
| Decoy068 | S(=O)(=O)(NC)c1cc(ccc1)C(OCC(=O)C=1C(=O)N(C)C(=O)N(Cc2ccccc2)C=1N)=O | 0 |
| Decoy069 | IC1=CN=C(N(C[C@@H](CCCC)CC)C1=O)C | 0 |
| Decoy070 | s1cccc1[C@H]1CC(=O)[C@H]2[C@H]3N(c4c(N=C2C1)cccc4)C(=O)c1c3cccc1 | 0 |
| Decoy071 | O=C(N[C@H](\C(\[O-])=N\N=C\c1c2c(n(c1)CC(=O)N)cccc2)C(C)C)c1ccc(cc1)C | 0 |
| Decoy072 | O1N=C(N[C@H]1C(=O)NC(CC)(CO)CO)C=1C=2[C@@H](N=CC=2)C=CC=1 | 0 |
| Decoy073 | O1[C@@H](C[C@@H]2CO[C@@H](C\C(=C\C(OCCCCCCCC)=O)\C)[C@H](O)[C@@H]2O)[C@@H]1[C@H]([C@@H](O)C)C | 0 |
| Decoy074 | s1c(ncc1C[NH+]1CCC[C@@H]1c1nc(ccc1)C)-c1sccc1 | 0 |
| Decoy075 | Brc1ccc(cc1Cl)[C@@H]([NH2+]N)[C@H]1C[C@@H]1c1ccccc1 | 0 |
| Decoy076 | s1c(nnc1S(=O)(=O)N[C@@]1(C2=C(NC1=O)N(CCc1cc(OC)c(OC)cc1)C(=O)NC2=O)C(F)(F)F)NC(=O)C | 0 |
| Decoy077 | O(C(=O)C[C@H]([NH2+][C@H](C(C)C)C(=O)NC(C)(C)C)C(=O)[O-])C[C@H](C(C)C)C(=O)NC(C)(C)C | 0 |
| Decoy078 | S1(=O)(=O)C[C@@H](N\C=C\2/c3c(cccc3)C(=O)N(c3cccc(C)c3C)C/2=O)CC1 | 0 |
| Decoy079 | O(C(=O)CCC(=O)N([C@@H](C(=O)c1c(C)c(n(C)c1C)C(OC)=O)C)CC=C)CC | 0 |
| Decoy080 | Cl[C@H](C)c1[nH+]c2cc(ccc2n1[C@@H](C(C)C)COC)C | 0 |
| Decoy081 | Brc1ccc(cc1F)C(=O)N(Cc1oc(cc1)C)C(C)C | 0 |
| Decoy082 | S(=O)(=O)(N1CCCCC1)c1cc(C(=O)[O-])c(NCC[NH+]2CCOCC2)cc1 | 0 |
| Decoy083 | Brc1c(cc(F)cc1S(=O)(=O)N)C(=O)N1[C@@H]2CC[C@@H](C2)C1 | 0 |
| Decoy084 | S(Cc1ncccc1)c1[nH+]cc(n1CC(F)(F)F)-c1ccccc1 | 0 |
| Decoy085 | O(C)c1ccc(cc1)-c1ncc(cn1)C[NH+]1C[C@@H](CCC)[C@H](N(C)C)C1 | 0 |
| Decoy086 | O=C(N([C@@H](C(=O)NC1CCCCC1)c1ccc(cc1)C)CCCCCC)[C@@H]1C[C@H]2C[C@@H]([C@H]1C)C2(C)C | 0 |
| Decoy087 | BrCC(CC)(CC)C[NH+]1CCCC[C@H]1CC | 0 |
| Decoy088 | Brc1ccc(SC[C@H](N[NH3+])c2ccc(F)c(F)c2F)cc1 | 0 |
| Decoy089 | S(CC(=O)N\N=C\c1cccc(OC)c1[O-])c1nnc(n1N)-c1cc([N+](=O)[O-])cc([N+](=O)[O-])c1 | 0 |
| Decoy090 | Brc1c(n(nc1C)-c1nc2SC=Cn2c1CO)C | 0 |
| Decoy091 | O(C)c1ccc(cc1)Cc1c([nH+]c2n(Cc3ncccc3)c(C)c(c2c1N)C)C | 0 |
| Decoy092 | s1cc(nc1-c1ccc(F)cc1)C(Oc1cc2[nH]ncc2cc1)=O | 0 |
| Decoy093 | O=C1N=C(N[C@@H]2N(C=N[C@H]12)CO[C@@H](COC(=O)[C@@H]([NH3+])C(C)C)CO)[NH-] | 0 |
| Decoy094 | S1CCC2(OCC[C@H](C2)[C@@H](O)c2nn(cc2)C)CC1 | 0 |
| Decoy095 | Brc1cccnc1SCC[C@@H]1CCC[C@]1([NH2+]C)C(=O)[O-] | 0 |
| Decoy096 | Fc1cc2c(CC[C@@H]2NC(=O)Nc2cc(ccc2C)-c2occn2)cc1 | 0 |
| Decoy097 | Clc1ccc(SC2=C3C(=[NH+][C@@H]2C(=O)NC[C@H]2OCCC2)C=C(C=C3)C)cc1 | 0 |
| Decoy098 | S(C1c2c(-c3c1cccc3)cccc2)c1nnc(n1CC=C)C | 0 |
| Decoy099 | Clc1ccccc1Cn1cccc1CN(C(=O)CN(S(=O)(=O)C)CCCOCC)CC(C)C | 0 |
| Decoy100 | Clc1ccc(cc1)[C@@H](NC(=O)N)CC(OCC(=O)NC(=O)NCC=C)=O | 0 |
| Decoy101 | S1CCSC[C@H]1[C@H](O)[C@H]1CC2(OCC1)CCOCC2 | 0 |
| Decoy102 | s1c(ccc1S(=O)(=O)N1Cc2c3[C@@H]([NH+](CCc3ccc2)C)C1)C | 0 |
| Decoy103 | O1c2c(ccc(NC(=O)[C@@H](NC(=O)CNC(=O)[C@@H](NC(OC(C)(C)C)=O)CCC(=O)N)CCCNC(=[NH2+])N)c2)C(=CC1=O)C | 0 |
| Decoy104 | S([C@@H](C)c1oc(nn1)-c1ccc(F)cc1)c1nnc(n1Cc1ccccc1)N1CCOCC1 | 0 |
| Decoy105 | s1ccc(C(=O)N)c1NC(=O)COC(=O)c1ccccc1N=S(=O)([O-])C | 0 |
| Decoy106 | Fc1ccc(cc1)[C@H]1n2c3c(nc2NC(C)=C1C(OCC=C)=O)cccc3 | 0 |
| Decoy107 | S(C)C1=Nc2c(n(Cc3ccc(cc3)C)c(c2)C)C(=O)N1CCC(C)C | 0 |
| Decoy108 | Clc1ccc(cc1)[C@H]1N2C(S\C(=C\c3cc4c(cc3)ccnc4)\C2=O)=NC2=C1C(=O)CCC2 | 0 |
| Decoy109 | Brc1ccc(cc1)[C@@H](N1C[C@@H]([NH2+]C[C@H]1CC)C(C)C)C | 0 |
| Decoy110 | Fc1cc(ccc1)\C=C\1/Oc2c(cc3c(OC[NH+](C3)[C@H](C)c3ccccc3)c2C)C/1=O | 0 |
| Decoy111 | Clc1cc2OC(=O)N(c2cc1)CCC(OCC=1N=C2SC=3CCCCC=3N2C(=O)C=1)=O | 0 |
| Decoy112 | S1SC[C@H]2NC(=O)[C@H](NC(=O)[C@@H](NC(=O)[C@H](NC(=O)[C@@H](NC2=O)C1)C(C)C)CC(C)C)[C@@H](CC)C | 0 |
| Decoy113 | S1C[C@@H]2NC(=O)N[C@@H]2[C@@H]1CCCCC(=O)NCCCCCC(=O)NCCCCCNC(=O)[C@@H]([NH3+])CCCNC(=S)N | 0 |
| Decoy114 | Clc1ccc(cc1)-c1oc(cc1)\C=C/1\C(C)=C(C#N)C(=O)N(C)C\1=O | 0 |
| Decoy115 | s1ccc(C#N)c1NC(=O)C[NH+](CCCc1[nH]nc(N)c1C#N)C | 0 |
| Decoy116 | O=C1c2c(N(C)C(=O)C1=C1NNC([O-])=C1\C=N\c1ncccc1)cccc2 | 0 |
| Decoy117 | O=C1N(CC(=O)N(Cc2ccccc2)C=2C(=O)NC(=O)N(CCCC)C=2N)C(=O)NC12CCCC2 | 0 |
| Decoy118 | Ic1ccc(cc1)CN1C[C@@H]([NH2+]C[C@@H]1CCC)C | 0 |
| Decoy119 | S(C)c1ccc(cc1)[C@H]1Oc2c([C@@H]3N1N=C(C3)c1ccc(F)cc1)cccc2OC | 0 |
| Decoy120 | Fc1cc(ccc1NC(=O)[C@H](Nc1cc2nc(oc2cc1)[O-])C)C | 0 |
| Decoy121 | Fc1c(N2CC([NH+]3CC[NH2+]CC3)C2)c(F)c(F)c(F)c1F | 0 |
| Decoy122 | Brc1cc(ccc1)[C@H]1N(C(=O)C([O-])=C1C(=O)c1sc(nc1C)C)c1sccn1 | 0 |
| Decoy123 | Clc1ccc(S(=O)([O-])=Nc2c3c(c4oc(C)c(c4c2)C(=O)C)cccc3)cc1C | 0 |
| Decoy124 | S(Cc1ccccc1)C=1NC(=O)C2=C(NC(=N)[C@H](C#N)[C@]23c2c(N(Cc4ccccc4F)C3=O)cccc2)N=1 | 0 |
| Decoy125 | Brc1cc(NS(=O)(=O)c2ccc(N3C(=O)c4c(cccc4)C3=O)cc2)ccc1 | 0 |
| Decoy126 | s1ccc(N=S(=O)([O-])c2c3c(ccc2)cccc3)c1-c1onc(n1)C1CC1 | 0 |
| Decoy127 | S(=O)([O-])(=Nc1cc(ccc1N1CC[NH2+]CC1)C(=O)NCCC)c1ccccc1F | 0 |
| Decoy128 | S1c2c(ccc(F)c2)C(=O)[C@@H](c2cc(S(=O)(=O)N(C)C)ccc12)Cc1cc(S(=O)(=O)N(C)C)ccc1Sc1ccccc1 | 0 |
| Decoy129 | Clc1c(Cl)c(nc(Cl)c1Cl)C(=O)Nc1sc2cc(S(=O)(=O)C)ccc2n1 | 0 |
| Decoy130 | s1c(nnc1SCCCCCCCCCCCCCCCCCC)N | 0 |
| Decoy131 | S(=O)(=O)(c1cnc(SCC(=O)N)nc1[O-])c1ccc(cc1)C(C)C | 0 |
| Decoy132 | O(CC1CC1)CCN(C(=O)/C(/[O-])=N/c1cnc(nc1)C(C)(C)C)C | 0 |
| Decoy133 | Oc1ccc(N2C3=C([C@H](CC2=O)c2ccc([N+](=O)[O-])cc2)C(=O)CC(C3)(C)C)cc1 | 0 |
| Decoy134 | Clc1cc(NC(=O)CSC=2NC(C)=C(C(=O)Nc3c(cccc3C)CC)[C@H](C=2C#N)c2occc2)ccc1C | 0 |
| Decoy135 | S([C@H](C)c1cc(F)c(F)cc1)c1[nH+]c2c(n1CCOC)cccc2 | 0 |
| Decoy136 | s1c2c(CC([NH2+]C2(C)C)(C)C)c(C(OCC)=O)c1NC(=O)c1ccc(S(=O)(=O)N(C[C@@H]2OCCC2)C)cc1 | 0 |
| Decoy137 | S(=O)(=O)(N)c1ccc(\N=C(/[O-])\C[NH+]([C@@H](C)c2occc2)C2CC2)cc1 | 0 |
| Decoy138 | Fc1ccccc1C(=O)N(CCCOCC)CC(=O)N(Cc1oc(cc1)C)CCc1ccccc1 | 0 |
| Decoy139 | Brc1cc(cc2OCCCOc12)[C@@H]([NH+]([C@@H](CC)C)C)CN | 0 |
| Decoy140 | Brc1ccc(S(=O)(=O)c2nc(oc2SCC(=O)NC[C@H]2OCCC2)-c2ccc(cc2)C)cc1 | 0 |
| Decoy141 | s1c(ccc1C[NH+]1[C@@H]2CC[C@@H](CN(S(=O)(=O)C)C2)C1)-c1ccccc1 | 0 |
| Decoy142 | O=C(NCCC[NH+](CC)CC)C=1N=C([C@@H]2[NH+]=C3C(=C2C=1)C=CC=C3)c1ccc(cc1)C | 0 |
| Decoy143 | Brc1ccc(cc1)Cn1nccc1NC(=O)[C@@H](SCCCC)C | 0 |
| Decoy144 | S(=O)(=O)(NCc1ccccc1)c1ccc(S(=O)(=O)NCCc2ccncc2)cc1 | 0 |
| Decoy145 | Clc1ccccc1C(=O)Nc1nc2c(cn1)C(=O)C[C@H](C2)c1ccc(cc1)C(C)C | 0 |
| Decoy146 | o1nc(nc1CCC(=O)Nc1cc2c([nH]nc2C(=O)N)cc1)C(C)C | 0 |
| Decoy147 | Brc1c(n(nc1C)C)C[NH+]1C[C@H](O[C@H](C1)C)C | 0 |
| Decoy148 | s1c2C[C@@H](CCc2c2c1N=C(NC2=O)C[NH+](CCOC)C[C@H](O)COCC)C | 0 |
| Decoy149 | Brc1cc2nc(n(c2nc1)[C@H](Cc1ccsc1)C)N | 0 |
| Decoy150 | O1[C@H]2[C@H](C[C@@H]3[C@](CCCC3=C)(C2)C)[C@@H](C[NH+](CCCCCCCCCC)CCCCCCCCCC)C1=O | 0 |
| Decoy151 | O1C[C@@H](OC(=O)C)[C@H](OC(=O)[O-])[C@@H](OC(=O)C)[C@H]1Oc1ccc([N+](=O)[O-])cc1 | 0 |
| Decoy152 | Clc1ccccc1N1C(=O)/C(/SC1=S)=C\c1ccc(OC)c(OC)c1C(=O)[O-] | 0 |
| Decoy153 | Brc1cc2NC(=S)N(c2nc1)[C@@H]1CCS(=O)(=O)C1 | 0 |
| Decoy154 | S(=O)(=O)(N1CCCCC1)c1c[nH+]ccc1N1CCN(N(C(=O)CC)C(=O)CC)CC1 | 0 |
| Decoy155 | S(=O)(=O)(N)c1c(n[nH]c1CC)C(=O)NC[C@H]1CCOC1 | 0 |
| Decoy156 | Brc1cc2nc(n(c2cc1F)C[C@@H]([NH+](C)C)C)CCCl | 0 |
| Decoy157 | s1nc(C(=O)N)c(N)c1C(=O)N([C@H](C(=O)NCCOC)c1ccc(OC)cc1)c1ccc(cc1C)C | 0 |
| Decoy158 | Brc1ccc(S(=O)([O-])=Nc2cc(ccc2Cl)C(=O)N)cc1 | 0 |
| Decoy159 | Brc1ccc(S[C@H](C[C@]([NH2+]C2CC2)(C(=O)[O-])C)C)nc1 | 0 |
| Decoy160 | S(C(=O)C)[C@H]1[C@H]2[C@H]3CC[C@]4(OC(=O)CC4)[C@@]3(CC[C@H]2[C@@]2(C(C1)=C[C@H](OC(=O)C)CC2)C)C | 0 |
| Decoy161 | Ic1ccc(cc1)-c1oc(cc1)[C@H]([NH2+]C)CC | 0 |
| Decoy162 | s1ccnc1\N=C/1\S\C(=C\c2ccc(O[C@H](C(=O)[O-])C)cc2)\C(=O)N\1C | 0 |
| Decoy163 | s1c2c([nH+]c1N(CC(=O)N(CC)CC)C)c(ccc2C)C | 0 |
| Decoy164 | Clc1ccc(cc1)Cc1nc(on1)[C@]1(CCC[NH2+]C1)CC | 0 |
| Decoy165 | O(Cc1ccccc1CCOC(=O)CCCOC)C(=O)CCCOC | 0 |
| Decoy166 | S1C2=NC(=NC(=O)C2=C(C)[C@H]1C)[C@H](OC(=O)c1ccc(N2C(=O)c3c(cccc3)C2=O)cc1)C | 0 |
| Decoy167 | o1nc(-c2nc3c(n2CC(=O)Nc2nn(nc2C(=O)N)-c2ccccc2)cccc3)c(n1)N | 0 |
| Decoy168 | Clc1ccc(N2CCN(S(=O)(=O)c3cc4OCC(=O)Nc4cc3C)CC2)cc1 | 0 |
| Decoy169 | Brc1c(n(nc1CC)C)C[NH+]1C[C@@H](CCC1)CCCl | 0 |
| Decoy170 | Brc1ccc(nc1)C(=O)Nc1sc(cn1)C | 0 |
| Decoy171 | O(C)c1ccc(cc1)C[C@H]([NH+](Cc1nc2n(c1)C(=CC(=N2)C)C)CC)C | 0 |
| Decoy172 | O=C1N=C([O-])NC=C1C(OCC(=O)N1N=C/2[C@@H](CCC\C\2=C\c2ccccc2)[C@H]1c1ccccc1)=O | 0 |
| Decoy173 | S(CC(=O)NCc1ccc(F)cc1)c1nnc(n1N)N\N=C\c1ccccc1OC | 0 |
| Decoy174 | S(C(F)F)c1ccc(cc1)C(OCc1onc(n1)-c1ccc[nH+]c1)=O | 0 |
| Decoy175 | S(CC(=O)NCC(=O)[O-])c1nc2c(C[C@@H](CC2)CC)c(C(F)(F)F)c1C#N | 0 |
| Decoy176 | S=C(\N=C(\Nc1nc(cc(n1)C)C)/NCc1cccnc1)Nc1ccccc1C | 0 |
| Decoy177 | O=C1NC(=O)NC(=O)C1(Cc1ccccc1CC1(C(C)(C)C)C(=O)NC(=O)NC1=O)C(C)(C)C | 0 |
| Decoy178 | O=C1N(C[C@H](C1)c1[nH+]c(c[nH]1)-c1ccccc1C)C1CCCC1 | 0 |
| Decoy179 | S\1\C(=C/c2ccccc2)\C(=O)N/C/1=N\N=C\c1ccccc1 | 0 |
| Decoy180 | FC(F)(F)c1ccc(cc1)-c1[nH+]c2n(C=CC=C2)c1NC(CC(C)(C)C)(C)C | 0 |
| Decoy181 | Clc1cc(ccc1N)-c1onc(n1)-c1cc(F)cc(F)c1 | 0 |
| Decoy182 | Fc1ccccc1NC(=O)Cn1c2c([nH+]c1CCNC(=O)[C@H]1OCCC1)cccc2 | 0 |
| Decoy183 | S(=O)(=O)(NC1CC([O-])C1)c1cc(n(c1)CCC)C(=O)[O-] | 0 |
| Decoy184 | S(CC(=O)C=1[C@H]([NH3+])N(CCC)C(=O)NC=1[O-])[C@H]1n2c3c(nc2N=N1)cccc3 | 0 |
| Decoy185 | O=C1N(N=C(C=C1)c1ccccc1)C[NH+]1C[C@@H](c2c(C1)cccc2)c1ccccc1 | 0 |
| Decoy186 | S(=O)(=O)(N1CCCC[C@@H]1C(=O)[O-])c1ccc(cc1)/C(=N/[N-]C(=S)Nc1ccccc1C)/C | 0 |
| Decoy187 | S\1c2cc(S(=O)(=O)C)ccc2N(C)/C/1=N/C(=O)c1ccccc1F | 0 |
| Decoy188 | Clc1cc(N2C(=O)[C@H]3[C@H](C4c5c(cccc5)C3(c3c4cccc3)C(OC)OC)C2=O)ccc1 | 0 |
| Decoy189 | O=C/1Nc2c(cccc2)\C\1=N/[N-]c1[nH+]c2N(C)C(=O)N(C)C(=O)c2n1C[C@@H](O)COC(C)C | 0 |
| Decoy190 | Brc1ncn(n1)C12CC3(C[C@@H](C1)C[C@H](C3)C2)C(=O)N1CCc2c1cccc2 | 0 |
| Decoy191 | S(CCOc1ccccc1C)c1nnc(n1C1CC1)-c1ccncc1 | 0 |
| Decoy192 | s1cccc1-c1nc2n(nc(c2c(c1)C(=O)N[N-]C(=O)NCC)C)C | 0 |
| Decoy193 | S(=O)(CC(=O)NCCc1ccccc1C)c1nc(n[n-]1)CC | 0 |
| Decoy194 | O=C(Nc1ncnc(N2CCN(CC2)c2ccccc2)c1)c1cc2c([nH]cc2)cc1 | 0 |
| Decoy195 | O=C(N\N=C/c1ccc(cc1)C(=O)[O-])c1c(C)c([nH]c1C)C(=O)N\N=C/c1ccc(cc1)C(=O)[O-] | 0 |
| Decoy196 | ClC(Cl)(Cl)C1=[NH+][C@@H]2[C@H](N1)CS(=O)(=O)C2 | 0 |
| Decoy197 | [O-]c1nc2n(n1)C(=NC(CCC)=C2Cc1ccc(cc1)-c1ccccc1-c1nnn[n-]1)C | 0 |
| Decoy198 | P(OCCCCCC[NH2+]C(c1ccc(OC)cc1)(c1ccccc1)c1ccccc1)(OCCC#N)N(C(C)C)C(C)C | 0 |
| Decoy199 | S([C@H](/C(/[O-])=N/C=1C(=O)N(N(C)C=1C)c1ccccc1)C)CC(=O)N(CC)CC | 0 |
| Decoy200 | Clc1ccc(N2C(=N)c3c(n[nH]c3C)N=C2S[C@@H](CC)C(=O)NCc2occc2)cc1C | 0 |
| Decoy201 | s1ccc(C)c1C[NH+](CN1N=C(c2c(cccc2)C1=O)c1cc(ccc1C)C)C | 0 |
| Decoy202 | s1c2c(c3c1N[C@H](N=C3O)c1ccccc1[O-])CC[NH+](C2)C | 0 |
| Decoy203 | O=C1N2C(=NC(=C1)C[NH+](Cc1ccc(cc1)C)CC)C(=CC=C2)C | 0 |
| Decoy204 | S(CCCC)c1ccccc1NC(=O)CSc1nnc(n1N)N\N=C\c1c2c(ccc1[O-])cccc2 | 0 |
| Decoy205 | FC(C(F)(F)C(F)(F)C(F)(F)C(F)(F)C(F)(F)C(F)(F)C[C@H](O)COC(=O)C=C)(C(F)(F)F)C(F)(F)F | 0 |
| Decoy206 | S1(=O)(=O)C[C@H](NC(=O)Nc2cn(nc2)CC(=O)[O-])C=C1 | 0 |
| Decoy207 | s1cc(nc1\N=C\1/S\C(=C\c2ccccc2OCC(=O)[O-])\C(=O)N/1CC)C | 0 |
| Decoy208 | Fc1cccc(F)c1N1CC[C@@H](NC(=O)NC2CCC(CC2)C)C1=O | 0 |
| Decoy209 | Brc1ccc(S(=O)([O-])=Nc2cc(S(=O)(=O)N3CCCCC3)ccc2)cc1C | 0 |
| Decoy210 | S(=O)(=O)(\N=C(\SCCC[NH+](CC)CC)/[NH-])c1ccc(N)cc1 | 0 |
| Decoy211 | Brc1ccc(cc1)\C=C\1/Sc2n(nc(n2)\C=C\c2sccc2)C/1=O | 0 |
| Decoy212 | s1cccc1[C@H]1N(Cc2cccnc2)C(=O)C([O-])=C1C(=O)c1cc2C[C@H](Oc2cc1)C | 0 |
| Decoy213 | S1(=O)(=O)N=C(N[C@H](C(=O)N(CCCc2ccccc2)CC[NH+](C)C)C)c2c1cccc2 | 0 |
| Decoy214 | S(CC(=O)Nc1ccccc1C#N)C=1NC(=O)C(Cc2ccccc2)=C([O-])N=1 | 0 |
| Decoy215 | Ic1c(nc(nc1Cl)[C@@H]1CCCc2c1cccc2)C(C)C | 0 |
| Decoy216 | ClC=1C=CC2=[NH+]CC(=C2C=1)CCNC(=O)c1ccccc1NC(=O)[C@H]1NSc2c1cccc2 | 0 |
| Decoy217 | s1c2c(CC[C@@H](C2)C(C)(C)C)c(C(=O)N)c1NC(=O)CC(C)C | 0 |
| Decoy218 | Clc1ccc(-n2nnnc2SCC#CCOC(=O)C2=Cc3c(OC2=O)cccc3)cc1 | 0 |
| Decoy219 | Brc1cc(N2CC([NH2+]C[C@H]2C(C)C)(CC)CC)c(F)cc1 | 0 |
| Decoy220 | Clc1cc(ccc1F)[C@H](Nc1nc(nc(Cl)c1)N)C | 0 |
| Decoy221 | O1[C@H]2N=NC(=C2[C@@H](C(C#N)=C1N)c1ccc(cc1)C)CCC | 0 |
| Decoy222 | s1cccc1[C@@H]([NH2+]CC=1N=C2N(C=C(C=C2)C)C(=O)C=1)c1ccc(F)cc1 | 0 |
| Decoy223 | S(Cc1ccccc1F)c1[nH+]cc(n1CCC(c1ccccc1)c1ccccc1)CO | 0 |
| Decoy224 | Fc1ccccc1C(=O)NCCCCCc1[nH+]c2c(n1[C@H](C)c1ccccc1)cccc2 | 0 |
| Decoy225 | Ic1ccccc1C(=O)\N=C\1/Sc2c(N/1CC#C)c(F)ccc2 | 0 |
| Decoy226 | S(=O)(=O)(N1C[C@H](CC[C@@H]1C)C(=O)N)c1ccc(OCCCCC)cc1 | 0 |
| Decoy227 | s1cccc1C(=O)N(C(=O)c1sccc1)c1nc(c(nn1)-c1occc1)-c1occc1 | 0 |
| Decoy228 | S=C(Nc1ccc(cc1C)C)N1C[C@H](CCC1)c1[nH]c2cc(C)c(cc2n1)C | 0 |
| Decoy229 | S1\C(=C\c2oc(cc2)-c2ccccc2C#N)\C(=O)N(c2c3c(ccc2)cccc3)C1=S | 0 |
| Decoy230 | Brc1cc(ccc1Nc1n(nc(C)c1N)C)C | 0 |
| Decoy231 | S(=O)(=O)(CCCCCCCC)CCSCCS(=O)(=O)CCCCCCCC | 0 |
| Decoy232 | Brc1cc(Cl)c(N[C@@H]2C[C@H](CCC2)C(F)(F)F)cc1 | 0 |
| Decoy233 | Clc1cnccc1\N=C\1/S\C(=C/c2cc(F)ccc2)\C(=O)N/1C | 0 |
| Decoy234 | S(C)c1ccccc1N1[C@H]2C[C@H]([NH+](C2)C\C(=C\c2ccccc2)\C)C1=O | 0 |
| Decoy235 | Brc1sc(cc1)[C@H]([NH+](Cc1ccsc1)C1CC1)[C@H](N)CC | 0 |
| Decoy236 | Brc1cc(Nc2sc(C(=O)CC(C)C)c(n2)N)ccc1 | 0 |
| Decoy237 | s1c2nc(cc(c2c(N)c1C(=O)N[C@@H]1CCC=CC1)C)C | 0 |
| Decoy238 | [NH2+]1CCc2nc[nH]c2[C@H]1c1cc2c3c(n(c2cc1)CC)cccc3 | 0 |
| Decoy239 | s1cccc1[C@@H]1CC(=O)C2=C(NC(=O)C[C@@H]2c2cc([N+](=O)[O-])ccc2)C1 | 0 |
| Decoy240 | Clc1ccc(cc1)/C(/Cl)=C/[C@@H]1C(C#N)C(=NC(C)=C1C#N)C | 0 |
| Decoy241 | OCCN(C(=O)c1n[nH]c(c1)-c1ccccc1[O-])C\C=C\c1ccccc1 | 0 |
| Decoy242 | Clc1ccc(\N=C/C23[C@H]4[C@H](C(c5c2cccc5)c2c3cccc2)C(=O)N(c2ccccc2)C4=O)cc1 | 0 |
| Decoy243 | Ic1cc(N)c(NCCC[NH+]2CCCC[C@@H]2C)cc1F | 0 |
| Decoy244 | s1c(ccc1C)CN(C(C)C)C(=O)Nc1ccccc1S[C@@H](CC#N)C | 0 |
| Decoy245 | s1c2CCCc2c2c1nc(nc2N1CCn2c(ccc2)[C@@H]1C)C | 0 |
| Decoy246 | Brc1cc(cc(OC)c1OCCC(F)(F)F)C[C@@H]([NH3+])CC | 0 |
| Decoy247 | Brc1sc(cc1)CNS(=O)(=O)c1cc(sc1)C[NH2+]CCC | 0 |
| Decoy248 | s1cccc1-c1[nH+]c(S[C@@H](C#N)C)c(C#N)c(c1)C(F)(F)F | 0 |
| Decoy249 | Clc1c2c(n(nc2C[NH+]2C[C@]3(N(CC2)C)CCC(=O)NCC3)C)ccc1 | 0 |
| Decoy250 | S1CC(=O)Nc2cc(NC(=O)Cc3cc(n(c3C)-c3ccc(cc3)C)C)ccc12 | 0 |
| Decoy251 | O1C(=Cc2c(C1=O)c(O)c1c(OC(=O)[C@@H](OC)C[C@H]1O[C@@H](O)[C@@H](O)[C@@H](O)[C@@H](O)[C@@H](O)CO)c2)C | 0 |
| Decoy252 | O(C)c1nc2c(cc1[C@@H]1CC(=O)NCc3nc4n(C=C(C=C4)C)c13)cccc2 | 0 |
| Decoy253 | s1c2c(CC(OC2)(C)C)c2c1nc(SCC(C)=C)nc2N | 0 |
| Decoy254 | S1c2c(Nc3c1cccc3)cc(cc2)/C(=N\Nc1ncc(cc1)C(F)(F)F)/C | 0 |
| Decoy255 | Ic1cc(Oc2nc3n(C=CC=C3)c2C[NH2+]CC)ccc1 | 0 |
| Decoy256 | O(C(=O)C[NH+](CC(=O)N(CC=C)CC=C)CC(OC)=O)C | 0 |
| Decoy257 | S(CC(=O)N(CC)C1CC1)c1nc2CCCCc2cc1C#N | 0 |
| Decoy258 | S(=O)([O-])(=NNC(=O)c1n(ccc1)C)c1cc2oc([O-])nc2cc1 | 0 |
| Decoy259 | S(=O)(=O)(N(CC)CC)c1cc2c(nc([O-])cc2/C(/O)=N/C[C@@H]2OCCC2)cc1 | 0 |
| Decoy260 | o1cccc1C=1C=CC(=O)N(C=1)Cc1ccccc1-c1ccccc1 | 0 |
| Decoy261 | Fc1cc(NC(=O)C[C@@H](CC(C)(C)C)C)c(cc1)C(=O)[O-] | 0 |
| Decoy262 | o1cccc1[C@@H]1[C@H](N2[C@H](c3c(C=C2)cccc3)C1(C#N)C#N)C(=O)N | 0 |
| Decoy263 | Fc1ccc(N2CCN(CC2)C(=O)C2CCN(CC2)c2nnc(-n3ncnc3)cc2)cc1 | 0 |
| Decoy264 | S(=O)(=O)(N(CCC)CCC)N1CC[NH+](CC1)CCC(=O)[O-] | 0 |

**Table S3.** Structures of the 435 Compounds (in SMILE format) from the training set together with their activities (1 or 0) for H_2_O_2_-induced models.

| ChEMBL ID | Structure | Activity |
| --- | --- | --- |
| CHEMBL1076243 | Clc1cc2sc(cc2cc1)CC(=O)NC(CCC(=O)NCCC1CC[NH+](CC1)Cc1ccccc1)C(OCCCCCC)=O | 1 |
| CHEMBL1163571 | Clc1ccc(cc1)-c1nc(SCC)nnc1-c1ccc(Cl)cc1 | 1 |
| CHEMBL1782123 | O1c2c(OC1)cc1c(-c3c(cc(OC)c(OC)c3OC)[C@H](OC(=O)\C(=C\C)\C)[C@@H](C)[C@@H](C)[C@H]1O)c2OC | 1 |
| CHEMBL451738 | O=C1C2=C(NC(N)=C(C#N)C2c2ccc(cc2)C)CC(C1)(C)C | 1 |
| CHEMBL512096 | O=C1C2=C(Nc3[nH+]c4c(CCCC4)c(N)c3C2c2ccccc2)CC(C1)(C)C | 1 |
| CHEMBL436302 | O(C)c1ccccc1C1c2c([nH+]c3c(CCCC3)c2N)NC(C)=C1C(OCC)=O | 1 |
| CHEMBL508313 | O(C(=O)C=1C(C(C#N)=C(NC=1C)N)c1cccnc1)CC | 1 |
| CHEMBL1428 | O(C(=O)C=1C(C(C(OCCOC)=O)=C(NC=1C)C)c1cc([N+](=O)[O-])ccc1)C(C)C | 1 |
| CHEMBL1214707 | O1[C@H]([C@H](O)[C@@H](O)[C@H](O)[C@H]1CO)c1c(O)c(c2Oc3c(cc(O)c(O)c3)C(=O)c2c1O)-c1cc(O)c2Oc3c(C(=O)c2c1O)c(O)cc(O)c3 | 1 |
| CHEMBL220294 | O(C(=O)C=1C(c2c([nH+]c3c(CCCC3)c2N)NC=1C)c1cc([N+](=O)[O-])ccc1)CC | 1 |
| CHEMBL219172 | Fc1ccc(cc1)C1c2c([nH+]c3c(CCCC3)c2N)NC(C)=C1C(OCC)=O | 1 |
| CHEMBL1163601 | S(CC)c1nc(c(nn1)-c1ccccc1)-c1ccccc1 | 1 |
| CHEMBL506792 | O1c2c(OC1)cc1c(-c3c(cc(OC)c(OC)c3OC)C[C@@H](C)[C@@H](C)[C@H]1O)c2OC | 1 |
| CHEMBL468001 | O(C)c1ccc(cc1)C1C2=C(Nc3[nH+]c4c(CCCC4)c(N)c13)CC(CC2=O)(C)C | 1 |
| CHEMBL1938456 | Clc1cc2N(NC(=O)C[NH+]3CCCCC3)c3c(Sc2cc1)cccc3 | 1 |
| CHEMBL1080631 | O(CCCCCC)C(=O)[C@@H](NC(=O)c1ccccc1)CCC(=O)NCCC1CC[NH+](CC1)Cc1ccccc1 | 1 |
| CHEMBL1938458 | S1c2c(N(NC(=O)C[NH+]3CCCC3)c3c1cccc3)cccc2 | 1 |
| CHEMBL468000 | Fc1ccc(cc1)C1C2=C(Nc3[nH+]c4c(CCCC4)c(N)c13)CC(CC2=O)(C)C | 1 |
| CHEMBL1938455 | Clc1c2N(NC(=O)C[NH+]3CCCCC3)c3c(Sc2ccc1)cccc3 | 1 |
| CHEMBL2333074 | OC1=C2[C@](CCCC2(C)C)(c2c(c(C3=C4C=C(O)C(=O)C=C4[C@]4([C@@H](C3=O)C(CCC4)(C)C)C)c(O)c(O)c2)C1=O)C | 1 |
| CHEMBL491360 | O(C(=O)C=1C(c2c([nH+]c3c(CCCC3)c2N)NC=1C)c1ccncc1)CC | 1 |
| CHEMBL509467 | O(C(=O)C=1C(C(C#N)=C(NC=1C)N)c1ccc(cc1)-c1ccccc1)CC | 1 |
| CHEMBL1938463 | Clc1cc2Sc3c(N(NC(=O)CC[NH+]4CCCC4)c2cc1)cccc3 | 1 |
| CHEMBL448447 | s1c2CCCCc2nc1NC(=O)CCC(OCC)=O | 1 |
| CHEMBL1938453 | Clc1cc2Sc3c(N(NC(=O)C[NH+](C)C)c2cc1)cccc3 | 1 |
| CHEMBL1957559 | O(C)c1ccccc1\C=C\C(=O)[C@H]1CC[C@H]2[C@H]3[C@H](CC[C@]12C)[C@@]1(C(C[C@@H](O)CC1)=CC3)C | 1 |
| CHEMBL1782119 | O1c2c(OC1)cc1c(-c3c(cc(OC)c(OC)c3OC)[C@H](OC(=O)C)[C@@H](C)[C@@H](C)[C@H]1OC(=O)c1ccccc1)c2OC | 1 |
| CHEMBL374184 | O(C)c1ccc(cc1)C1c2c([nH+]c3c(CCCC3)c2N)NC(C)=C1C(OCC)=O | 1 |
| CHEMBL1938397 | Clc1cc2Sc3c(N(NC(=O)CC[NH+]4CC[NH+](CC4)C)c2cc1)cccc3 | 1 |
| CHEMBL1938465 | S1c2c(N(NC(=O)CC[NH+]3CC[NH+](CC3)C)c3c1cccc3)cccc2 | 1 |
| CHEMBL218940 | O(C)c1cc(ccc1)C1c2c([nH+]c3c(CCCC3)c2N)NC(C)=C1C(OCC)=O | 1 |
| CHEMBL1214705 | O1[C@H](CO)[C@@H](O)[C@H](O)[C@@H](O)[C@@H]1O[C@@H]1C2=C(Oc3c(c(O)c(-c4cc(O)c5Oc6c(C(=O)c5c4O)c(O)cc(O)c6)c(O)c3)C2=O)[C@H](O)CC1 | 1 |
| CHEMBL512565 | O(C(=O)C=1C(C(C#N)=C(NC=1C)N)c1ccc(cc1)C)CC | 1 |
| CHEMBL512226 | OC1=C2[C@@](CCCC2(C)C)(c2c(cc(O)c(O)c2)C1=O)C | 1 |
| CHEMBL510656 | O(C)c1ccc(cc1)C1C(C(OCC)=O)=C(NC(N)=C1C#N)C | 1 |
| CHEMBL2180484 | O[C@]1(C[C@@H](O)[C@H]2[C@](CCC[C@@]2(CO)C)(C)[C@@H]1CCC(C=C)=C)C | 1 |
| CHEMBL1782111 | O1c2c(OC1)cc1c(-c3c(cc(OC)c(OC)c3OC)[C@@H](O)[C@@H](C)[C@@H](C)[C@H]1O)c2OC | 1 |
| CHEMBL218939 | O(C(=O)C=1C(c2c([nH+]c3c(CCCC3)c2N)NC=1C)c1ccccc1)CC | 1 |
| CHEMBL451743 | O=C1C2=C(NC(N)=C(C#N)C2c2ccncc2)CC(C1)(C)C | 1 |
| CHEMBL1782120 | O1c2c(OC1)cc1c(-c3c(cc(OC)c(OC)c3OC)[C@H](OC(=O)\C(=C\C)\C)[C@@H](C)[C@@H](C)[C@H]1OC(=O)C)c2OC | 1 |
| CHEMBL468316 | O1c2c(c(OC)c(O)c(OC)c2)C(=O)[C@H](O)[C@H]1c1ccc(OC)cc1 | 1 |
| CHEMBL559796 | O1c2c(OC1)cc1c(-c3c(cc(OC)c(OC)c3OC)C[C@@H](C)[C@@H](C)[C@H]1OC(=O)C)c2OC | 1 |
| CHEMBL1688934 | O1C[C@H]2[C@H](CO[C@@H]2c2cc3OCOc3cc2)[C@H]1c1cc(O)c(O)cc1 | 1 |
| CHEMBL452007 | O(C)c1ccc(cc1)C1C2=C(NC(N)=C1C#N)CC(CC2=O)(C)C | 1 |
| CHEMBL1076260 | s1c2ncccc2cc1C(=O)N[C@@H](CCC(=O)NCCC1CC[NH+](CC1)Cc1ccccc1)C(OCCCCCC)=O | 1 |
| CHEMBL460669 | O(C)c1cc(ccc1)C1C(C(OCC)=O)=C(NC(N)=C1C#N)C | 1 |
| CHEMBL1782117 | O1c2c(OC1)cc1c(-c3c(cc(OC)c(OC)c3OC)[C@H](OC(=O)C)[C@@H](C)[C@@H](C)[C@H]1OC(=O)CCC)c2OC | 1 |
| CHEMBL538371 | O1c2c(OC1)cc1c(-c3c(cc(OC)c(OC)c3OC)[C@H](C(=O)\C(=C\C)\C)[C@H](C)[C@@H](C)[C@H]1OC(=O)CC)c2OC | 1 |
| CHEMBL1938461 | Clc1cc2Sc3c(N(NC(=O)C)c2cc1)cccc3 | 1 |
| CHEMBL2012548 | O1c2c(OC1)cc1c(-c3c(cc(OC)c(OC)c3OC)C[C@@H](C)[C@@H](C)[C@H]1OC(=O)\C=C/c1ccccc1)c2OC | 1 |
| CHEMBL1782113 | O1c2c(OC1)cc1c(-c3c(cc(OC)c(OC)c3OC)[C@H](OC(=O)C)[C@@H](C)[C@@H](C)[C@H]1O)c2OC | 1 |
| CHEMBL1782114 | O1c2c(OC1)cc1c(-c3c(cc(OC)c(OC)c3OC)[C@H](OC(=O)C)[C@@H](C)[C@@H](C)[C@H]1OC(=O)C)c2OC | 1 |
| CHEMBL2036263 | Clc1cc2[nH+]c3c(CCCC3)c(NCCCCCCNC(=O)CCS)c2cc1 | 1 |
| CHEMBL468002 | O=C1C2=C(Nc3[nH+]c4c(CCCC4)c(N)c3C2c2ccncc2)CC(C1)(C)C | 1 |
| CHEMBL1076259 | s1c2c(cc1C(=O)N[C@@H](CCC(=O)NCCC1CC[NH+](CC1)Cc1ccccc1)C(OCCCCCC)=O)cccc2 | 1 |
| CHEMBL1782112 | O1c2c(OC1)cc1c(-c3c(cc(OC)c(OC)c3OC)[C@@H](O)[C@@H](C)[C@@H](C)[C@H]1OC(=O)C)c2OC | 1 |
| CHEMBL467247 | O1c2c(c(OC)c(OC)c(OC)c2)C(=O)[C@H](O)[C@H]1c1cc(O)c(O)cc1 | 1 |
| CHEMBL1080813 | s1cccc1C(=O)N[C@@H](CCC(=O)NCCC1CC[NH+](CC1)Cc1ccccc1)C(OCCCCCC)=O | 1 |
| CHEMBL460668 | O(C)c1ccccc1C1C(C(OCC)=O)=C(NC(N)=C1C#N)C | 1 |
| CHEMBL1782110 | O1c2c(OC1)cc1c(-c3c(cc(OC)c(OC)c3OC)C(=O)[C@@H](C)[C@H](C)[C@@H]1OC(=O)C)c2OC | 1 |
| CHEMBL480452 | s1cc(nc1\N=C\c1c2c([nH]c1)cccc2)-c1ccc(OCC)cc1OCC | 1 |
| CHEMBL501290 | s1cc(nc1NC(=O)\C=C\c1cc(OC)cc(OC)c1)-c1ccccc1 | 1 |
| CHEMBL460868 | O(C(=O)C=1C(C(C#N)=C(NC=1C)N)c1cc([N+](=O)[O-])ccc1)CC | 1 |
| CHEMBL501443 | O1c2c(OC1)cc1c(-c3c(cc(OC)c(OC)c3OC)C[C@@H](C)[C@@H](C)[C@H]1OC(=O)\C=C\c1ccccc1)c2OC | 1 |
| CHEMBL2180482 | O[C@@H]1C[C@@](C[C@@H]2CC=C3[C@H](CCCC3(C)C)[C@@]12C)([C@@H](O)CO)C | 1 |
| CHEMBL455119 | O(C)c1cc(ccc1OC)C1C(C(OCC)=O)=C(NC(N)=C1C#N)C | 1 |
| CHEMBL1938462 | S1c2c(N(NC(=O)CC[NH+]3CCCC3)c3c1cccc3)cccc2 | 1 |
| CHEMBL558251 | O(C)c1c(OC)c2-c3c(cc(OC)c(OC)c3OC(=O)\C(=C\C)\C)C[C@@H](C)[C@@H](C)[C@@H](O)c2cc1OC | 1 |
| CHEMBL1163570 | Clc1ccc(cc1)-c1nc(SC)nnc1-c1ccc(Cl)cc1 | 1 |
| CHEMBL2012549 | O(C)c1c(OC(=O)\C(=C/C)\C)c2-c3c(cc(OC)c(OC)c3OC)[C@H](O)[C@H](C)[C@H](C)[C@@H](OC(=O)\C(=C/C)\C)c2cc1OC | 1 |
| CHEMBL2012544 | O1c2c(OC1)cc1c(-c3c(cc(OC)c(OC)c3OC)[C@H](OC(=O)\C(=C/C)\C)[C@@H](C)[C@@H](C)[C@H]1OC(=O)\C(=C/C)\C)c2OC | 1 |
| CHEMBL1080274 | s1c2sccc2cc1C(=O)N[C@@H](CCC(=O)NCCC1CC[NH+](CC1)Cc1ccccc1)C(OCCCCCC)=O | 1 |
| CHEMBL2180483 | O[C@@H]1C[C@@H]2[C@]3(C[C@@]1(C=C3)CO)[C@@H](O)C[C@H]1[C@]2(CCCC1(C)C)C | 1 |
| CHEMBL219400 | O(C(=O)C=1C(c2c([nH+]c3c(CCCC3)c2N)NC=1C)c1ccccc1[N+](=O)[O-])CC | 1 |
| CHEMBL452158 | s1cc(nc1NC(=O)CCC(OCC)=O)-c1ccccc1 | 1 |
| CHEMBL513161 | O=C1C2=C(Nc3[nH+]c4c(CCCC4)c(N)c3C2c2ccc(cc2)C)CC(C1)(C)C | 1 |
| CHEMBL450611 | O(C(=O)C=1C(C(C#N)=C(NC=1C)N)c1ccncc1)CC | 1 |
| CHEMBL1163577 | Clc1ccc(cc1)-c1nc(SCCCC)nnc1-c1ccc(Cl)cc1 | 1 |
| CHEMBL2012546 | O1c2c(OC1)cc1c(-c3c(cc(OC)c(OC)c3OC)[C@H](OC(=O)\C(=C/C)\C)[C@@H](C)[C@@H](C)[C@H]1OC(=O)\C(=C\C)\C)c2OC | 1 |
| CHEMBL1782116 | O1c2c(OC1)cc1c(-c3c(cc(OC)c(OC)c3OC)[C@H](OC(=O)C)[C@@H](C)[C@@H](C)[C@H]1OC(=O)C(C)C)c2OC | 1 |
| CHEMBL455640 | O=C1C2=C(NC(N)=C(C#N)C2c2ccccc2)CC(C1)(C)C | 1 |
| CHEMBL219406 | O(C(=O)C=1C(c2c([nH+]c3c(CCCC3)c2N)NC=1C)c1ccc(cc1)C)CC | 1 |
| CHEMBL1214706 | O1[C@H](CO)[C@@H](O)[C@H](O)[C@@H](O)[C@@H]1Oc1c2c(Oc3c(c(O)c(-c4cc(O)c5Oc6c(C(=O)c5c4O)c(O)cc(O)c6)c(O)c3)C2=O)c(O)cc1 | 1 |
| CHEMBL491359 | O(C(=O)C=1C(c2c([nH+]c3c(CCCC3)c2N)NC=1C)c1cccnc1)CC | 1 |
| CHEMBL1782118 | O1c2c(OC1)cc1c(-c3c(cc(OC)c(OC)c3OC)[C@H](OC(=O)C)[C@@H](C)[C@@H](C)[C@H]1OC(=O)C(CC)C)c2OC | 1 |
| CHEMBL461077 | FC(F)(F)c1ccccc1C1C(C(OCC)=O)=C(NC(N)=C1C#N)C | 1 |
| CHEMBL2012547 | O1c2c(OC1)cc1c(-c3c(cc(OC)c(OC)c3OC)[C@H](OC(=O)\C=C\c3ccccc3)[C@@H](C)[C@@H](C)[C@H]1OC(=O)\C(=C/C)\C)c2OC | 1 |
| Decoy001 | S(=O)(=O)(Nc1ccc(cc1)C1[NH+]=C2C(=C1)C=C(F)C=C2)c1cc(ccc1)C | 0 |
| Decoy002 | s1c(ccc1[C@@H](Nc1cc2OC(Oc2cc1)(C)C)C)C | 0 |
| Decoy003 | Clc1ccccc1-c1noc([N-]C(=O)c2nc3n(n2)C(=CC(=N3)c2occc2)C(F)(F)F)c1 | 0 |
| Decoy004 | O1CCC[C@H]1COc1cc(ccc1NC(=O)N[C@H](C)c1[nH+]c2c([nH]1)cccc2)C | 0 |
| Decoy005 | O(CCC)c1cc(C)c(cc1)C(=O)C=1[C@H](N(CC[NH+](CC)CC)C(=O)C=1[O-])c1ccc(OCCCCC)cc1 | 0 |
| Decoy006 | Clc1cc(Cl)ccc1-c1nnc(SCc2cc([N+](=O)[O-])cc3c2OCOC3)n1CC(C)C | 0 |
| Decoy007 | Clc1ccc(cc1)[C@H]1N=C2[NH+]=CNN2[C@H](C1)c1c2c(ccc1)cccc2 | 0 |
| Decoy008 | Clc1ccc(cc1)CSc1sc(nn1)N1[C@@H](C(C(=O)c2cc3C[C@H](Oc3cc2)C)=C([O-])C1=O)c1cc(OCC)c(OCCC)cc1 | 0 |
| Decoy009 | Brc1cc(C[NH2+]C[C@@H]2N(CCC2)CC)c(OCc2ccc(cc2)C)cc1 | 0 |
| Decoy010 | Clc1cc(cnc1N1CC[NH+](CC1)[C@@H](C)c1nc(nc(n1)N)N(C)C)C(F)(F)F | 0 |
| Decoy011 | Clc1c2NC(=CC(=O)c2ccc1)CS[C@H]1[NH+](C)[C@H](NN1)c1cc(ccc1)C | 0 |
| Decoy012 | Brc1cc2c([nH+]cc(N)c2N2CC(CC2)(CC)CC)cc1 | 0 |
| Decoy013 | Clc1cc(Cl)c(F)cc1C(=O)Nc1ccc(nc1)-n1cc[nH+]c1 | 0 |
| Decoy014 | O=C1Nc2cc(C)c(cc2C=C1[C@@H]([NH+]1CCN(CC1)c1cccc(C)c1C)c1nnnn1Cc1ccccc1)C | 0 |
| Decoy015 | Brc1cc(ccc1)CC(=O)N1C[C@H](CCC1)/C(=N\O)/N | 0 |
| Decoy016 | O=C1Nc2cc(N[C@@H]3CC[NH+](C3)C3CC3)ccc2NC1=O | 0 |
| Decoy017 | FC(F)(F)c1ccc(cc1)[C@H](N(CCC)C(=O)\C=C\CCCCC)C(=O)NC(CC(C)(C)C)(C)C | 0 |
| Decoy018 | O1CC[NH+](CC1)C[C@@H]([C@@](O)(CCc1ccccc1)c1ccccc1)c1ccccc1 | 0 |
| Decoy019 | O=C(NCc1[nH]nc(c1)C)[C@@](Nc1ccc(cc1)C)(C)c1ccccc1 | 0 |
| Decoy020 | o1c2c(cc1C(=O)N1CCc3[nH]c4c(cc(cc4)C(OC)=O)c3C1)cccc2OC | 0 |
| Decoy021 | Clc1cc(S(=O)(=O)N2CC(O[C@H](C2)C)(C)C)cnc1NN | 0 |
| Decoy022 | O=C(NC1[C@H]2[C@H]1CCCC2)CCC=1C(=Nc2n(nc(c2)C)C=1C)C | 0 |
| Decoy023 | S1C=C(N2C1=NC=C(C(=O)Nc1cc(cc(c1)C(OC)=O)C(OC)=O)C2=O)c1ccc(F)cc1 | 0 |
| Decoy024 | Fc1cc(c2nc[nH]c2c1)C(=O)Nc1cc(NC(=O)[C@H](OC)C)ccc1 | 0 |
| Decoy025 | Clc1cccc(F)c1[C@@H]1n2nc[nH+]c2N[C@@H](C1)c1cc(C)c(cc1)C | 0 |
| Decoy026 | OCc1cc(C)c(Nc2nc([nH+]cc2)Nc2ccc(cc2)C#N)c(c1)C | 0 |
| Decoy027 | o1cccc1C(=O)Nc1cc2[nH+]c3n(CCN(C3)[C@@H](C)c3ccccc3)c2cc1 | 0 |
| Decoy028 | Fc1cc2nc([nH]c2cc1F)-c1c(cccc1N)C | 0 |
| Decoy029 | Brc1cc(F)c(N[C@H]2CCCC[C@H]2[C@H]2[NH2+]CCC2)cc1 | 0 |
| Decoy030 | O=C(N[C@@H](Cc1ccc[nH+]c1)C)c1c2c(nc(c1)-c1cn(nc1)C)cc(cc2C)C | 0 |
| Decoy031 | O1c2c(C(=O)C=C1c1cc(O)c(O)cc1)c(O)cc([O-])c2[C@@H]1O[C@H](CO)[C@@H](O)[C@H](O)[C@H]1O[C@@H]1OC[C@@H](O)[C@H](O)[C@H]1O | 0 |
| Decoy032 | O1C2=C([C@H](N(C2=O)c2ccc(OCC)cc2)c2cc(OCC)c(OCCCC)cc2)C(=O)c2cc(ccc12)C | 0 |
| Decoy033 | O=C/1N(c2c(cccc2)\C\1=C/c1c2c(n(c1)CC(C)C)cccc2)c1ccccc1 | 0 |
| Decoy034 | Clc1ccc(cc1)Cn1ncc(NS(=O)(=O)c2ccc(cc2)C(C)C)c1 | 0 |
| Decoy035 | Oc1ccc(cc1)[C@H](N(CCCCCC)C(=O)\C=C\CCCCC)C(=O)NC1CCCCC1 | 0 |
| Decoy036 | o1cccc1[C@@H](NC(=O)c1cnn(c1C)-c1nc2-c3c(CCCc2cn1)cccc3)C | 0 |
| Decoy037 | S(=O)(=O)(NC)c1oc(cc1)CNc1ccc(C)c(O)c1C | 0 |
| Decoy038 | Clc1ccc(NC(=O)[C@H](n2cnc(C)c2C)C)cc1N | 0 |
| Decoy039 | Fc1cc(ccc1C)[C@H](NC(=O)N([C@@H](C)c1[nH+]cccc1)C)C | 0 |
| Decoy040 | O1c2c(C=C(C(=O)Nc3ccc(OCc4ccccc4)cc3)C1=O)c(cnc2C)COC(=O)C(C)C | 0 |
| Decoy041 | s1cccc1S(=O)([O-])=Nc1scc([nH+]1)-c1ccc(N)cc1 | 0 |
| Decoy042 | Brc1cc2cc(oc2cc1)[C@H]([NH3+])c1cc(F)c(cc1F)C | 0 |
| Decoy043 | S(=O)(=O)(N)c1cc(N[C@H]2CCS(=O)(=O)C2)ccc1 | 0 |
| Decoy044 | O1[C@H](CO)[C@@H](O)[C@H](O)[C@@H](O[C@@H]2OC[C@](O)(CO)[C@H]2O)[C@@H]1O[C@H]1CC[C@@]23[C@H]([C@@H](O)C[C@@H]4[C@]2(CC[C@@]2(C)[C@]4(C[C@H](O)C2[C@@]2(O[C@@H](CC2)C(O)(C)C)C)C)C3)C1(C)C | 0 |
| Decoy045 | S(Cc1[nH+]c2c([nH]1)cccc2)[C@H]1[NH+](Cc2occc2)[C@@H](NN1)c1ccc(cc1)C | 0 |
| Decoy046 | Fc1ccc(cc1)[C@@H](NC(=O)COC(=O)c1cc2c(cc1)C(=O)N(Cc1occc1)C2=O)C | 0 |
| Decoy047 | o1cccc1[C@H]1c2c(OC=3N=Cn4nc(nc4C1=3)CO\N=C(\C)/c1ccccc1)n(nc2C)-c1ccccc1 | 0 |
| Decoy048 | [NH2+]1C[C@H](CCC1)c1cc(nc(N)c1C#N)-c1ccc(N2CCCCC2)cc1 | 0 |
| Decoy049 | Clc1sc(S(=O)(=O)N2C[C@@H](CCC2)C(=O)Nc2oc(nn2)-c2cc(cc(c2)C)C)cc1 | 0 |
| Decoy050 | O(CCCCn1c2c(nc1CCNC(=O)c1ccccc1C)cccc2)c1ccc(cc1OC)CC=C | 0 |
| Decoy051 | Clc1ccc(cc1)-c1nnc(N2C[C@@H](CCC2)C(=O)Nc2ncnc(N3CCC(CC3)C)c2)cc1 | 0 |
| Decoy052 | O(CCCC)c1ccc(cc1)[C@H]1N(CCC[NH+](CC)CC)C(=O)C(=O)C1=C([O-])c1ccc(OCCCC)cc1 | 0 |
| Decoy053 | O(CCCC)c1cc(ccc1)C(=O)C=1[C@H](N(CCC[NH+](CC)CC)C(=O)C=1[O-])c1ccc(OCCCC)cc1 | 0 |
| Decoy054 | Brc1cc2cc(oc2cc1)[C@H](Nc1c(n([nH+]c1C)C)C)C | 0 |
| Decoy055 | O=C1Nc2c(cccc2)C(=C1)CNC(=O)Nc1c(cccc1C)CC | 0 |
| Decoy056 | S(=O)(=O)(N(CC)c1ccccc1)c1cc([N+](=O)[O-])c(NC[C@@H]2OCCC2)cc1 | 0 |
| Decoy057 | Clc1ccccc1N(S(=O)(=O)c1cc([N+](=O)[O-])c(Cl)cc1)S(=O)(=O)\C=C/c1ccccc1 | 0 |
| Decoy058 | Fc1cc(-n2c3c(nc2[C@@H](O)C)cc(N)cc3)ccc1F | 0 |
| Decoy059 | O=C(N[C@@H](CCC)C)[C@H]1CCCN(C1)C(=O)NC1CC([NH2+]C(C1)(C)C)(C)C | 0 |
| Decoy060 | S=C(NC(C)C)N1CCC2([NH2+][C@H]([C@H](CC)C)C(=O)N2C[C@@H](CC)C)CC1 | 0 |
| Decoy061 | O1[C@H]2[C@]34CC[NH+]([C@H](Cc5c3c1c(OC(=O)CCCCCCCCCCCCCC)cc5)[C@]4(O)CCC2=O)CC=C | 0 |
| Decoy062 | s1c(ccc1NC(=O)Nc1ccccc1)C(=O)Nc1ccc(-n2cc[nH+]c2)cc1 | 0 |
| Decoy063 | Clc1ccccc1OS(=O)(=O)c1cc(ccc1C)C | 0 |
| Decoy064 | OC[C@H]1CCC[NH+](C1)CC(=O)NC(=O)NC1CCCCC1 | 0 |
| Decoy065 | Clc1ccc(cc1)C=1N(CC=C)/C(/SC=1)=N/c1ccccc1OC | 0 |
| Decoy066 | o1cccc1\C=C\CN1CCC=2[C@H]([NH+]=C3C=2C=CC=C3)[C@H]1c1cc(O)ccc1 | 0 |
| Decoy067 | Brc1cc(-n2c3ncnc(OC)c3nc2N)c(F)cc1 | 0 |
| Decoy068 | Ic1cn(nc1)CCCC(/C(=N/O)/N)(C)C | 0 |
| Decoy069 | Clc1c(cccc1Cl)\C=C\C=1OC(=O)/C(/N=1)=C\c1ccc(F)cc1 | 0 |
| Decoy070 | Fc1ccccc1NC(=O)Nc1ccc(Nc2nc([nH+]c(c2)C)N2CCOCC2)cc1 | 0 |
| Decoy071 | Clc1c(n(nc1C)CCNc1nc(nc(N2CCOCC2)c1)N)C | 0 |
| Decoy072 | O1[C@H](CO)[C@@H](O)[C@H](O)[C@@H](O)[C@@H]1O[C@H]1CC[C@]2([C@@H](CC[C@@]3([C@@H]2CC=C2[C@@H]4CC(CC[C@]4(C(O[C@@H]4O[C@H](CO)[C@@H](O)[C@H](O)[C@H]4O)=O)[C@H](O)C[C@]23C)(C)C)C)C1(C)C)C | 0 |
| Decoy073 | S=C(Nc1ccccc1OCCOc1ccccc1NC(=S)NCCCC)NCCCC | 0 |
| Decoy074 | Clc1cc2c(NC(=O)[C@@H]2O)cc1N(CC(=O)NC(C)C)C | 0 |
| Decoy075 | O1CCN(CC1)c1nc(nc(n1)N\N=C\c1ccc(OCc2ccccc2)cc1)N(c1ccccc1)c1ccccc1 | 0 |
| Decoy076 | Fc1ccc(F)cc1-n1c2c(nc1[C@@H](O)C)cc(N)cc2 | 0 |
| Decoy077 | Clc1cc(ccc1)C[C@]1(CCCN(C1)c1[nH+]ccc(n1)N)CO | 0 |
| Decoy078 | Brc1cc(ccc1F)[C@@H](N1[C@@H]2[C@H](CCCC2)CCC1)C[NH3+] | 0 |
| Decoy079 | s1cnc(C2CC2)c1C(=O)N[C@H]1CCC[NH+](C1)c1ccc(cc1)C | 0 |
| Decoy080 | [NH+]1(CCc2c([nH]c3c2cccc3)[C@@H]1C1CCCCC1)Cc1cnn(C)c1C | 0 |
| Decoy081 | Clc1ccc(OCCN2C(=O)[C@]3(NC2=O)CCCC[C@@H]3C)cc1 | 0 |
| Decoy082 | S\1\C(=C/c2cc(OCC)c(OCc3cc4OCOc4cc3)cc2)\C(=O)N(C)/C/1=N\c1ccccc1CC | 0 |
| Decoy083 | O1CCOC12CCC([NH2+]Cc1ccc(N3CCCCCC3)cc1)CC2 | 0 |
| Decoy084 | Brc1cc\2c(NC(=O)/C/2=N\N/C(/[S-])=N/C=2C(=O)N(N(C)C=2C)c2ccccc2)cc1 | 0 |
| Decoy085 | Clc1cc(cc2OCCOc12)C[NH2+][C@H](C)c1oc(cc1)C | 0 |
| Decoy086 | Brc1cc(F)c(NCC=2[NH+]=C3N(C=C(C=C3)C)C(=O)C=2)cc1 | 0 |
| Decoy087 | Brc1cc(Br)cc(C[NH2+][C@H]2C[C@@H](C)[C@H](CC2)C)c1OCC | 0 |
| Decoy088 | S(=O)(=O)(N(Cc1cc([N+](=O)[O-])cc2c1OCOC2)Cc1ccccc1)c1cc(ccc1)C(F)(F)F | 0 |
| Decoy089 | Cl\C(=C\c1ccccc1)\C=C\1/N=C(OC/1=O)c1c2c(ccc1)cccc2 | 0 |
| Decoy090 | S(=O)(=O)(N1[C@H](C[NH2+]C[C@@H]1C)C)c1c(C)c(cc(C)c1C)C | 0 |
| Decoy091 | Clc1cc(ccc1Cl)CC1(CCN(CC1)c1ncnc2[nH]ccc12)C[NH3+] | 0 |
| Decoy092 | [NH2+]([C@@H](C)c1n2c(nn1)C=CC=C2)[C@@H]1CCCc2c1cc(cc2)C(C)C | 0 |
| Decoy093 | Clc1ccc(Nc2[nH+]cnc(N3CCCCC3)c2N)cc1C(F)(F)F | 0 |
| Decoy094 | O=C(N[C@@H](C(=O)N1CC[NH+](CC1)C)C12CC3CC(C1)CC(C2)C3)C1CCCCC1 | 0 |
| Decoy095 | O1CCOc2c1cc(cc2)-c1nn(cc1C(=O)Nc1cc([N+](=O)[O-])c(cc1)C)-c1ccccc1 | 0 |
| Decoy096 | O=C(Nc1ccc(cc1C)C)c1[nH]c[nH+]c1C(=O)Nc1ccc(cc1)C | 0 |
| Decoy097 | s1cccc1[C@@H]1CC=2N(c3ccc(F)cc3F)C(=O)C[C@H](C=2C(=O)[C@@H]1C(OCC)=O)c1cc(OC)c(OC)c(OC)c1 | 0 |
| Decoy098 | [S-]C1=[NH+]N[C@@H](N1\N=C\c1cc(O)ccc1)[C@H]1N=NC2=C1CCC2 | 0 |
| Decoy099 | O(Cc1ncccc1)c1ccc(cc1)C[NH2+]Cc1c[nH]nc1-c1ccccc1 | 0 |
| Decoy100 | Fc1c(F)cc(N[C@@H](C(=O)NC(=O)N)C)cc1F | 0 |
| Decoy101 | Fc1ccccc1Cn1cccc1C[NH+](CC1CCCCC1)C[C@@H](O)CC | 0 |
| Decoy102 | Clc1nc(nc(C)c1CC)-c1oc(cc1)[C@@H]1C[C@@H]1C | 0 |
| Decoy103 | S\1c2c3c(ccc2N(CCOC)/C/1=N/C(=O)c1ccc(S(=O)(=O)N(CCC)CCC)cc1)cccc3 | 0 |
| Decoy104 | O([C@@H]1CC(C[C@@H](C1)C)(C)C)c1cc(ncc1)/C(=N/O)/N | 0 |
| Decoy105 | O=C1N2C(=NN1CC(=O)Nc1cc(C)c(cc1)C)C(=NC=C2)N1CCN(CC1)c1ccc(cc1)C | 0 |
| Decoy106 | Brc1cc(Br)cc(Br)c1NCc1[nH+]cc[nH]1 | 0 |
| Decoy107 | O1CC(=O)Nc2cc(NC(=O)c3[nH]c(C)c(C(=O)C)c3C)ccc12 | 0 |
| Decoy108 | O=C(N[C@H](C)c1[nH+]cccc1)c1c2c(nc(c1)C(C)C)n(nc2C)C(C)(C)C | 0 |
| Decoy109 | o1cccc1[C@H]1OC[C@@H]2O[C@@H](Oc3ccccc3C)[C@H](O)[C@H](O)[C@H]2O1 | 0 |
| Decoy110 | S(=O)(=O)(C(F)(F)F)c1cc([N+](=O)[O-])c(S(=O)CC(Oc2ccc(cc2)C2CCCCC2)=O)cc1 | 0 |
| Decoy111 | Brc1cc2[nH]c([nH+]c2nc1)COc1ccc(cc1C)C | 0 |
| Decoy112 | Ic1c([nH+]n([C@H]2CC[C@@H](CCC2)C(C)(C)C)c1N)C | 0 |
| Decoy113 | S1(=O)(=O)C[C@H](NC(=O)[N-]\N=C\2/C[C@H](CC[C@@H]/2C(C)C)C)CC1 | 0 |
| Decoy114 | Brc1ccccc1[C@H](O)CC[NH+](C)c1ccc(O)cc1 | 0 |
| Decoy115 | Clc1cc(ccc1NCC=1[NH+]=C2N(C=CC=C2)C(=O)C=1)C(F)(F)F | 0 |
| Decoy116 | Fc1cc2CCN(Cc2cc1)C(=O)N[C@@H](C)c1ccc(cc1)-c1ccc[nH+]c1 | 0 |
| Decoy117 | O1[C@H]2[C@@H](CC1)[C@H](N\C(=[NH+]/CC)\NCc1ccccc1OCC1CC1)C2(C)C | 0 |
| Decoy118 | Clc1ccc(N2CC(=O)Nc3c2c(F)c(F)cc3)cc1 | 0 |
| Decoy119 | S1(=O)(=O)C[C@@H](NC(=O)Nc2ccc(cc2)[C@@H](O)C)CC1 | 0 |
| Decoy120 | Fc1ccccc1NC(=O)N1C[C@H](CCC1)C(OC1CCCC1)=O | 0 |
| Decoy121 | S1CCN(CC1)c1nccnc1-c1cc(NC(=O)Nc2cc3OCCOc3cc2)ccc1 | 0 |
| Decoy122 | S(=O)(=O)(N1[C@@H]2[C@@H](C[C@H]1C)C[NH2+]C2)c1c(cc(cc1C)C)C | 0 |
| Decoy123 | S\1c2c(N(CCC)/C/1=N/C(=O)c1ccc(S(=O)(=O)N3CCCC[C@@H]3CC)cc1)cc1OCOc1c2 | 0 |
| Decoy124 | Brc1c(n(nc1C)CC)C[C@@H]1C[C@@H](CC[C@@H]1[NH3+])C(C)(C)C | 0 |
| Decoy125 | Clc1ccc(Cl)nc1C(=O)Nc1n(nc(c1)C(C)(C)C)[C@@H]1CCS(=O)(=O)C1 | 0 |
| Decoy126 | s1cccc1-c1nn2c(c1)C(=O)N(N=C2)Cc1onc(n1)-c1ccc(OCc2ccccc2)cc1 | 0 |
| Decoy127 | S(=O)(=O)(N1CCC(CC1)(CC)C)c1ccc(nc1)NN | 0 |
| Decoy128 | O1c2c([C@H]3N(N=C(C3)c3ccccc3)[C@H]1C1CCCCC1)cccc2 | 0 |
| Decoy129 | O=C(NC(C)(C)C)[C@@H](N(CCCCCC)C(=O)[C@H](CCCC)CC)c1ccc(N(C)C)cc1 | 0 |
| Decoy130 | o1c(C)c(cc1C)[C@H](NC(=O)Nc1cc2c(cc1)C(=O)NC2=O)C | 0 |
| Decoy131 | Fc1ccccc1[C@H]1[NH+]=NC(=CN1O)c1ccc(F)cc1 | 0 |
| Decoy132 | O(CC)c1cc(ccc1OCCCC)[C@@H]1N(CCC[NH+](C)C)C(=O)C([O-])=C1C(=O)\C=C\c1ccccc1 | 0 |
| Decoy133 | Clc1cc2c(OC(=O)C3=C2CCCC3)c(C[NH+]2CCCC[C@@H]2CC)c1O | 0 |
| Decoy134 | Brc1cc(Oc2nnc(cc2)C[NH2+]C(C)(C)C)c(Cl)cc1 | 0 |
| Decoy135 | Clc1nc2c(cc1CN(CCOC)C(=O)NC1CCCCC1)cccc2C | 0 |
| Decoy136 | O(CC(C)C)c1ccc(cc1)C(=O)C=1[C@@H](N(CC[NH+](C)C)C(=O)C=1[O-])c1ccc(OCCCCC)cc1 | 0 |
| Decoy137 | O1CC[NH+](CC1)CCCN1[C@H](C2=C(Oc3c(ccc4c3cccc4)C2=O)C1=O)c1ccc([N+](=O)[O-])cc1 | 0 |
| Decoy138 | Fc1cc(F)ccc1Oc1n(nc(C)c1C[NH+](C[C@@H]1OCCC1)C[C@H](O)COCC(C)C)-c1ccccc1 | 0 |
| Decoy139 | s1c2N=C(NC(=O)c2c(C)c1C)[C@H](Sc1nnc(n1Cc1occc1)-c1cc(OC)ccc1)C | 0 |
| Decoy140 | O=C(N(Cc1[nH]ncn1)C)C1(CCCC1)CC(C)C | 0 |
| Decoy141 | s1c2c(nc(SC)nc2SCC(=O)N(CC)CC)c2c3CC(OCc3c(nc12)N1CCOCC1)(C)C | 0 |
| Decoy142 | O1CC[NH+](CC1(C)C)[C@@H](C(=O)NC(=O)Nc1ccc(cc1C)C)C | 0 |
| Decoy143 | o1cc(nc1-c1ccc(OC)cc1)CN1C(=O)[C@@](NC1=O)(CCc1ccc(OC)cc1)C | 0 |
| Decoy144 | Ic1cc(cc(N)c1OCc1nonc1C)C#N | 0 |
| Decoy145 | Brc1cc(\C=C(\C#N)/C2[NH+]=C3C(=[NH+]2)C=CC=C3)c([O-])cc1 | 0 |
| Decoy146 | Clc1ccc(cc1[N+](=O)[O-])C(=O)Nc1ccc(-n2nc(OCC(C)C)nc2-c2cc(ccc2)C(F)(F)F)cc1 | 0 |
| Decoy147 | Brc1cc(OCC(OC)=O)c(OC)cc1\C=N\NC(=O)CC[C@@H](O)c1ccc(OCC)cc1 | 0 |
| Decoy148 | o1nc(cc1C(=O)N1C[C@H](CCC1)c1[nH]nc(c1)C(=O)N)[C@@H](CC)C | 0 |
| Decoy149 | Oc1ccc(NC(=O)C)cc1C(=O)N[C@H]1C[C@H](CCC1)C | 0 |
| Decoy150 | S\1c2n(nc(n2)-c2cc(OC)c(OC)cc2)C(=O)/C/1=C/c1cc(OC)c(OCCCCCC)cc1 | 0 |
| Decoy151 | Clc1ccc(N2O[C@H]3[C@H]([C@H]2c2ccc(OC(=O)c4ccccc4)cc2)C(=O)N(c2ccc(F)cc2)C3=O)cc1 | 0 |
| Decoy152 | Fc1ccc(cc1)C=1C2=NC(=O)N(CC(=O)N3CCN(CC3)c3ccccc3)C(=O)C2=NC=1 | 0 |
| Decoy153 | s1c(ccc1C[NH+]1CC(=O)NCCC1)C#CC[NH3+] | 0 |
| Decoy154 | S(CC)c1nc(N2CCCC2)c2c(n1)n(nc2)CCNC(=O)c1cc(OCC)c(OCC)c(OCC)c1 | 0 |
| Decoy155 | Brc1cc(ccc1)[C@H]1[NH+]=C2N(NC=N2)[C@@H](C1)c1ccc(Cl)cc1 | 0 |
| Decoy156 | BrC=1C=Cc2nc([NH2+][C@@H]3CCC=CC3)nn2C=1 | 0 |
| Decoy157 | FC(F)(F)Oc1cc2c([nH+]cc(C#N)c2NN)cc1 | 0 |
| Decoy158 | Brc1ccc(cc1)C(=O)C=1[C@H](N(CCC[NH+](CCCC)CCCC)C(=O)C=1[O-])c1ccc(cc1)CC | 0 |
| Decoy159 | Brc1ccc(O)cc1\C=N/NC(=O)[C@@H](O)c1ccccc1 | 0 |
| Decoy160 | o1cccc1C1=NN([C@@H](C(=O)N2CCc3c2cccc3)C)C(=O)C(N)=C1 | 0 |
| Decoy161 | S([C@H](C(C)C)C(=O)NC(=O)N)c1oc(nn1)-c1ccoc1C | 0 |
| Decoy162 | Brc1cc(OCc2nnsc2NN)cnc1 | 0 |
| Decoy163 | S([C@H](C(=O)Nc1cc2OCOc2cc1)C)c1nnc(n1Cc1occc1)N1CCCCC1 | 0 |
| Decoy164 | Clc1n(nc(C)c1\C=C/C(OCC1=NC(=O)c2oc3c(c2N1)cccc3)=O)Cc1ccc(cc1)C | 0 |
| Decoy165 | Brc1cc(ccc1)[C@@H]1[NH+]=C2N(NC=N2)[C@H](C1)c1ccc(Cl)cc1 | 0 |
| Decoy166 | Brc1ccc(cc1F)[C@@H](N[NH3+])c1oc2c(c1)cccc2Cl | 0 |
| Decoy167 | S([C@@H](C)c1nnnn1-c1ccccc1)c1nnc(n1-c1ccc(cc1)C)-c1ccc(OC)cc1 | 0 |
| Decoy168 | Fc1ccc(cc1)CN(C(=O)CN(CCCOC)C(=O)[C@@H](CCCC)CC)CCc1c2c([nH]c1)cccc2 | 0 |
| Decoy169 | S(=O)(=O)(N([C@H](CC(C)C)C)C)c1c[nH+]ccc1NC | 0 |
| Decoy170 | O(c1n(nc(C)c1C[NH+]([C@@H](CC)C)C[C@H](O)COCC=C)-c1ccccc1)c1cc(OC)ccc1 | 0 |
| Decoy171 | S([C@@H](C(=O)c1ccc(cc1C(C)C)C(C)C)C)c1nc2c(n1C(C)C)cccc2 | 0 |
| Decoy172 | s1c(C=O)c(nc1N1CCc2sccc2C1)C(C)(C)C | 0 |
| Decoy173 | s1c2c(nc1N(C(=O)c1ccc(S(=O)(=O)N(CCCC)C)cc1)C[C@@H]1OCCC1)c(OC)ccc2 | 0 |
| Decoy174 | Fc1ccc(cc1)[C@@H]1N(CCc2c1cc(OCc1occ(n1)C(=O)NCc1occc1)cc2)C(=O)C(C)C | 0 |
| Decoy175 | O(CCCC)c1ccc(cc1)\C=C/1\c2nc3c(cccc3)c(c2C[NH+](C\1)C)C(=O)[O-] | 0 |
| Decoy176 | Clc1nnc(NCc2cn(nc2)Cc2ccccc2)c(c1)C(=O)N | 0 |
| Decoy177 | Brc1cc(oc1Br)[C@H](N(C)c1cc(F)ccc1)C[NH3+] | 0 |
| Decoy178 | s1cc(nc1N1N=C2[C@@H](CCCC2)[C@@]1(O)C(F)(F)F)-c1[nH+]cccc1 | 0 |
| Decoy179 | S=C1N(C)C(=N[N-]1)CNC(=O)Nc1cc2oc(nc2cc1)C1CC1 | 0 |
| Decoy180 | Fc1cc(ccc1F)[C@@H](O)[C@@H](NC(=O)NCC(C)(C)c1cc[nH+]cc1)C | 0 |
| Decoy181 | S1(=O)(=O)N(c2ccc(F)cc2)C(=O)N(c2c1cccc2)Cc1onc(n1)-c1cccc(OC)c1OC | 0 |
| Decoy182 | O=C(NC1CC1)c1cc(NC(=O)N[C@@H]2C[C@H](CCC2)CC)c(cc1)C | 0 |
| Decoy183 | Brc1cc(ccc1Oc1nc2SC=Cn2c1C[NH2+]C)C | 0 |
| Decoy184 | Clc1ccc(cc1S(=O)(=O)N1C[C@H](CCC1)C(=O)Nc1cc(C)c(cc1)C)-c1nc(on1)C | 0 |
| Decoy185 | O=C1N(C[C@H]2[C@]13[NH+](CCC3)[C@@H](C2)c1[nH]c2c(cccc2)c1C)C1CCCC1 | 0 |
| Decoy186 | O(CC)c1cc2C=C([C@H]([NH+]3CC[NH+](CC3)C3CCCCC3)c3nnnn3C(C)(C)C)C(=O)Nc2cc1 | 0 |
| Decoy187 | S(CC(=O)N\N=C/c1c(n(nc1C)-c1ccccc1)C)c1nnc(n1-c1ccc(cc1)C)-c1ccc(cc1)C(C)(C)C | 0 |
| Decoy188 | O=C(N1C[C@@H](CCC1)c1[nH+]c(cc(c1)-c1ccc(nc1)N)C)c1n[nH]c2c1cc(cc2)C | 0 |
| Decoy189 | O(c1ncnc(N2CC[NH+](CC2)C(c2ccccc2)c2ccccc2)c1[N+](=O)[O-])c1ccc(cc1)C(C)C | 0 |
| Decoy190 | Fc1cc(ccc1)-c1nc(nc2[nH+]n(Cc3ccccc3)c(N)c12)NC[C@H]1OCCC1 | 0 |
| Decoy191 | Fc1cc(F)cc(F)c1[C@@H](NN)[C@H]1C[C@H](C)[C@@H](CC1)C | 0 |
| Decoy192 | O=C(NC(C)(C)C)[C@H](NC(=O)Nc1ccc(nc1)-n1nccc1)C | 0 |
| Decoy193 | Fc1cc(-n2nc(c(-c3ccc(NC(=O)c4occc4)cc3)c2OC(=O)C)-c2ccccc2OC)ccc1 | 0 |
| Decoy194 | O(C(C)C)c1nc(nc(N2C[C@@]3(CCC(=O)N(C3)CCO)CCC2)c1)N | 0 |
| Decoy195 | Brc1cc2cc(oc2cc1)-c1[nH+]nc(NCC)cc1 | 0 |
| Decoy196 | O=C1Nc2n(nc(c2C=C1C[NH+]1C[C@@H](CCC1)C)-c1ccccc1)-c1ccccc1 | 0 |
| Decoy197 | S1C=CC[C@H]1[C@H](C[NH2+][C@@H](C(=O)N[C@@H](C)c1ccccc1)C)C=1C[NH+]=C2C=1C=CC=C2 | 0 |
| Decoy198 | O[C@H](C)c1cc(Nc2[nH+]c3c(cccc3)c(n2)N[C@@H](C)c2ccccc2)ccc1 | 0 |
| Decoy199 | s1c2N=Cn3nc(nc3-c2c(C)c1C(OC)=O)-c1cc(ccc1)COc1ccc(cc1[N+](=O)[O-])C | 0 |
| Decoy200 | s1cc(N)cc1S(=O)(=O)NC=1C=CC(=O)N(C=1)CC | 0 |
| Decoy201 | O(C(=O)c1ccc(cc1)[C@H](N(C(=O)Cn1nnc2c1cccc2)c1cc2c(nc1)cccc2)C(=O)NC1CCCCC1)C | 0 |
| Decoy202 | Brc1cc(cc(F)c1)-c1nc(on1)[C@@]1(CC[NH2+]C1)C | 0 |
| Decoy203 | O1[C@@H]2[C@@H]([NH+](CC1)[C@@H]1C[C@H](CC[C@H]1C#N)CCC)CCCC2 | 0 |
| Decoy204 | Ic1cc2[nH+]c(n(c2cc1)[C@@H](C)c1sccc1)N | 0 |
| Decoy205 | Brc1c2c(ccc1OCC(=O)N\N=C\c1cc(OC)c(OC(=O)c3ccc(OCCCC)cc3)cc1)cccc2 | 0 |
| Decoy206 | S(Oc1cc(N(CC)CC)ccc1CN(CC(C)C)C(=O)Nc1ccccc1OC)(=O)(=O)c1ccc(OC)cc1 | 0 |
| Decoy207 | O(CC)c1cc(ccc1OCCCC)[C@@H]1N(CCCn2cc[nH+]c2)C(=O)C([O-])=C1C(=O)\C=C\c1ccccc1 | 0 |
| Decoy208 | O=Cc1ccc(N2N=C(C[C@H]2c2c3c(ccc2)cccc3)c2ccccc2)cc1 | 0 |
| Decoy209 | Brc1ccc(cc1)CN1C(=O)/C(/SC1=O)=C/c1cc([N+](=O)[O-])c(O)cc1 | 0 |
| Decoy210 | Brc1ccc(NC(=O)c2nn3c(N[C@@H](C[C@H]3C(F)(F)F)c3ccccc3)c2Cl)cc1 | 0 |
| Decoy211 | O1CC(=CC1=O)[C@H]1CC[C@]2(O)[C@H]3[C@H](CC[C@]12C)[C@]1(CC[C@H](O[C@@H]2O[C@@H](C)[C@@H](O[C@H]4O[C@H](CO)[C@@H](O)[C@H](O)[C@H]4O)[C@@H](O)[C@H]2O)C[C@@]1(O)CC3)C=O | 0 |
| Decoy212 | O1C=2N(Cc3ccccc3)C(=C3[C@H](N(N(C3=O)c3ccccc3)c3ccccc3)C=2[C@@H](CC1=O)C(=O)c1ccccc1)C | 0 |
| Decoy213 | O=C1Nc2c(C=C1[C@@H]([NH+]1CCC(CC1)C)c1nnnn1C(C)(C)C)cc(cc2)C | 0 |
| Decoy214 | Brc1ccc(\[NH+]=C\2/Oc3c(C=C/2C(=S)N)cccc3)cc1 | 0 |
| Decoy215 | S=C(N(Cc1cc2cc(ccc2nc1O)CC)Cc1ccc[nH+]c1)NCC(C)C | 0 |
| Decoy216 | Clc1ccc2scnc2c1NC(=O)[C@H]1C[C@@H]([NH3+])C=C1 | 0 |
| Decoy217 | S1CCN(CC12CCCCC2)C(=O)N[C@@H]1C[C@H]([NH+](C)C)CC1 | 0 |
| Decoy218 | s1c(C(=O)N(C)C)c(C)c(C(OC)=O)c1NC(=O)CSc1nnc(n1CC=C)[C@@H](Oc1ccc(cc1)CC)C | 0 |
| Decoy219 | S(Cc1ccccc1)C=1n2ncnc2C=C(N=1)N(S(=O)(=O)c1ccccc1)S(=O)(=O)c1ccccc1 | 0 |
| Decoy220 | Fc1ccc(cc1)C1=NN([C@H](C1)c1occc1)c1ccccc1 | 0 |
| Decoy221 | S\1C=2N(CCCN=2)C(=O)/C/1=C\c1oc(cc1)CNC(=O)C(=O)Nc1ccc(cc1)C | 0 |
| Decoy222 | Oc1c(cccc1O)CNc1ccccc1-n1nccc1 | 0 |
| Decoy223 | O1[C@H](CO)[C@@H](O[C@@H]2O[C@H](CO)[C@H](O)[C@H](O)[C@H]2O)[C@H](O)[C@@H](O)[C@@H]1O[C@@H]1CC[C@]2([C@H](CC[C@@]3([C@@H]2CC=C2[C@@H]4CC(CC[C@@]4(CC[C@]23C)C(=O)[O-])(C)C)C)C1(C)C)C | 0 |
| Decoy224 | O1C/C(/CC1=O)=C\1/CC[C@]2(O)[C@H]3[C@H](CC[C@]/12C)[C@]1(C(C[C@@H](O[C@H]2O[C@@H](CO)[C@@H](O)[C@@H](O[C@H]4O[C@@H](CO)[C@@H](O)[C@@H](O)[C@H]4O)[C@H]2O)CC1)=CC3)C | 0 |
| Decoy225 | Brc1cc2c(nc1[C@@H]([NH3+])c1cc(F)ccc1Cl)cccc2 | 0 |
| Decoy226 | Fc1cc(ccc1F)-c1[nH]c([nH+]c1)C1CCN(CC1)C(=O)C1CCCCC1 | 0 |
| Decoy227 | Brc1sc(cc1)[C@H](N1[C@@H]2[C@H](CCCC2)CCC1)[C@@H]([NH3+])C | 0 |
| Decoy228 | Clc1cc(Nc2[nH+]cnc(N(CC(C)C)CC(C)C)c2N)ccc1F | 0 |
| Decoy229 | S\1C=2N(N=C(c3ccccc3)C(=O)N=2)C(=O)/C/1=C/c1cn(nc1-c1cc(C)c(OCC=C)cc1)-c1ccccc1 | 0 |
| Decoy230 | Brc1cc(F)c(NC(=O)NCc2[nH]ccn2)cc1F | 0 |
| Decoy231 | S(C)c1nccn1-c1ccc(cc1)C(=O)N1CCC[C@H]1c1[nH+]c2c([nH]1)cc(cc2)C | 0 |
| Decoy232 | Brc1sc(cc1)[C@@H](SC1=N[N-]C(=O)N1C(C)C)[C@@H]([NH3+])CC | 0 |
| Decoy233 | Brc1ccc(Oc2nnc(Cl)c(C)c2C)cc1F | 0 |
| Decoy234 | O1[C@H](CO)[C@@H](O)[C@H](O)[C@@H](O)[C@@H]1OC(=O)[C@]12[C@H](C3=CC[C@@H]4[C@@](CC[C@@H]5[C@](C(=O)[O-])(CO)[C@H](O)[C@H](O)C[C@@]45C)(C)[C@@]3(CC1)C)[C@@](O)(C)[C@@H](CC2)C | 0 |
| Decoy235 | Clc1ccc(N(CCCCN2C(=O)c3c(cccc3)C2=O)C(=O)\C=C\c2ccc([N+](=O)[O-])cc2)cc1 | 0 |
| Decoy236 | O(CCCn1c2c(nc1CCCCCNC(=O)C(C)(C)C)cccc2)c1ccc(cc1OC)CC=C | 0 |
| Decoy237 | S(CC(=O)Nc1ccc(cc1)C(=O)N)c1nnc(n1C[C@@H](CCCCC)C)-c1ccc(cc1)C(C)(C)C | 0 |
| Decoy238 | Brc1c2oc(cc2ccc1)[C@@H]([NH2+]N)Cc1n(nc(c1)C)C | 0 |
| Decoy239 | Brc1c2oc(cc2ccc1)[C@H]([NH2+]C)c1cc(OC)cnc1 | 0 |
| Decoy240 | O1CC[NH+](CC1)CCn1c2c(nc1-n1nc(cc1N)C)cccc2 | 0 |
| Decoy241 | S(=O)(=O)(Nc1cn(nc1)C(C)C)c1c(N)cccc1F | 0 |
| Decoy242 | Brc1cnc(nc1)N[C@@H]1CC[C@H](C[C@H]1C)C | 0 |
| Decoy243 | s1ccc(C)c1[C@@H](NN)[C@@H]1CC2(OCC1)CCCC2 | 0 |
| Decoy244 | O1C[C@H]2O[C@@H](OC(=O)c3cc(O)c(O)c(O)c3)[C@@H](O)[C@@H](OC(=O)c3c(-c4c(cc(O)c(O)c4O)C1=O)c(O)c(O)c(O)c3)[C@@H]2O | 0 |
| Decoy245 | O=C1N(C=C(NCc2cn(nc2)C(C)(C)C)C=C1)CC | 0 |
| Decoy246 | s1c2N=C(NC(=O)c2c(C[C@H](CC)C)c1C)c1cc[nH+]cc1 | 0 |
| Decoy247 | [O-]C=1n2nc(cc2N=C2C=1C[NH+](CC2)Cc1ccc(-n2cc[nH+]c2C)cc1)C(C)(C)C | 0 |
| Decoy248 | S(CC(=O)N\N=C/c1cccc(OC)c1OC)C1=Nc2c(cccc2)C(=O)N1c1ccc(cc1)C | 0 |
| Decoy249 | Clc1cc(ccc1Cl)[C@@H]1N([C@@H]2CCS(=O)(=O)C2)C(=O)c2[nH]nc(c12)-c1cc(C)c(cc1O)C | 0 |
| Decoy250 | O=C1Nc2c(C=C1[C@H]([NH+]1CCN(CC1)c1cc(ccc1C)C)c1nnnn1C1CCCC1)cc(cc2)CC | 0 |
| Decoy251 | O1[C@H]([C@H](O)[C@@H](O)[C@H](O)[C@H]1CO[C@@H]1OC[C@](O)(CO)[C@H]1O)c1c(O)c2c(O[C@H](CC2=O)c2ccc(O)cc2)cc1O | 0 |
| Decoy252 | Clc1cc(NC(=O)[NH+]=C(NC2CCCC2)Nc2nc(cc(n2)C)C)ccc1Cl | 0 |
| Decoy253 | Brc1cc(F)cc(N)c1OCc1cc2c([nH+]ccc2)cc1 | 0 |
| Decoy254 | Fc1cc(NC(=O)N2C[C@@H](CC[C@@H]2C)C(=O)N)ccc1CC | 0 |
| Decoy255 | Clc1cc(N)c(S(=O)(=O)NO)cc1Cl | 0 |
| Decoy256 | Cl[C@@H]1Cc2c(cccc2)[C@H]1Nc1nc([nH+]cc1)N(C)C | 0 |
| Decoy257 | s1cccc1C1=NN(C(=O)CSc2nnc(n2-c2ccc(F)cc2)CNC(=O)c2cc(ccc2)C)[C@@H](C1)c1ccc(cc1)C | 0 |
| Decoy258 | Clc1cc(ccc1Cl)COC[C@@H](OCc1cc(Cl)c(Cl)cc1)Cn1c2c(nc1)N(C)C(=O)N(C)C2=O | 0 |
| Decoy259 | o1c2c(cc1[C@@H]1N=NC=C1C[NH2+][C@H]1CCCc3c1cccc3)cccc2 | 0 |
| Decoy260 | Clc1cc(Oc2cnc(nc2CCl)C(C)C)ccc1Cl | 0 |
| Decoy261 | O=C(NCC(C)C)[C@@H]1CCCN(C1)c1nnc(cc1)-c1ccc(cc1)C | 0 |
| Decoy262 | s1cccc1S(=O)(=O)N1CCC[C@@H]1C(=O)\N=C\1/Sc2cc(C)c(cc2N/1CC(OCC)=O)C | 0 |
| Decoy263 | Oc1c2c(C[C@@H]3C(=C([O-])[C@@]4(O)[C@@H](C3)[C@H]([NH+](C)C)C(=O)C(C(=O)N)=C4[O-])C2=O)c(N(C)C)cc1NC(=O)C[NH2+]C(C)(C)C | 0 |
| Decoy264 | Brc1c(nn(Cc2cc(ccc2)C)c1O)CC | 0 |
| Decoy265 | O1CC[C@](c2c1cccc2)([C@@H](O)C1CCCCCC1)C#N | 0 |
| Decoy266 | o1c(ccc1C)CN([C@H](\C=C\CCC)C(=O)NC(CC(C)(C)C)(C)C)C(=O)\C=C\CCCCC | 0 |
| Decoy267 | o1nc(nc1-c1cc(ccc1NC(=O)NCc1[nH]ccn1)C)C1CC1 | 0 |
| Decoy268 | s1c2c(ncnc2S[C@@H](C(OC)=O)C)c2c3CC(OCc3c(nc12)N1CCOCC1)(C)C | 0 |
| Decoy269 | Brc1cc(S(=O)(=O)NC[C@@H]2[NH+](CCCC2)C)c(N)cc1 | 0 |
| Decoy270 | s1c2c(CC[C@@H](C2)C)c(C(OC)=O)c1NC(=O)CSc1nnc(n1CC=C)-c1ccoc1C | 0 |
| Decoy271 | FC(F)(F)c1nc(-n2ccnc2/C(=N\O)/N)ccc1 | 0 |
| Decoy272 | Brc1ccccc1-c1c(noc1N)-c1[nH]ncc1 | 0 |
| Decoy273 | O=C(Nc1cccnc1-n1nccc1)[C@@H]1C[C@@H]([NH3+])C=C1 | 0 |
| Decoy274 | O1c2c(cc(cc2)C(=O)C=2[C@H](N(CCC[NH+](CC)CC)C(=O)C=2[O-])c2ccc(OCCCCC)cc2)C[C@@H]1C | 0 |
| Decoy275 | Brc1oc(cc1)[C@H](N[NH3+])Cc1n(nc(CC)c1Cl)CC | 0 |
| Decoy276 | Brc1cc([C@@H]([NH3+])c2c(F)cc(F)cc2F)c(OC)cc1C | 0 |
| Decoy277 | s1c2c(nc1N(Cc1occc1)C(=O)[C@H]1CCCN(S(=O)(=O)c3ccc(cc3)C)C1)c(OC)ccc2 | 0 |
| Decoy278 | S([C@H](C(=O)N1c2c(NC(=O)C1(C)C)cccc2)C)c1nnc(n1C)-c1ccccc1C | 0 |
| Decoy279 | FC(F)(F)c1c2nc(cc(c2ccc1)[C@H](O)[C@@H]1[NH2+]CCCC1)C(F)(F)F | 0 |
| Decoy280 | Oc1ccc(cc1)[C@@H](O)[C@@H]1NC(=O)CNC(=O)[C@@H](NC(=O)CNC(=O)[C@@H]2N(CCC2)C(=O)[C@H](NC(=O)CNC1=O)Cc1c2c([nH]c1)cccc2)C(C)C | 0 |
| Decoy281 | Brc1c([O-])c(Br)cc(\C=N/c2ccc(N3CC[NH+](CC3)CC)cc2)c1O | 0 |
| Decoy282 | o1cccc1-c1nc2cc(NC(=O)N3CCC(CC3)C)ccc2nc1-c1occc1 | 0 |
| Decoy283 | O1C[C@H](CC1)C[NH+]1CCC(CC1)C(=O)Nc1cc(ccc1)[C@@H]1[NH+]=C2C(=C1)C=CC=C2 | 0 |
| Decoy284 | S(=O)(=O)(N[C@@H]1C[C@H](CCC1)C)c1cc2c(NC(=O)NC2=O)cc1 | 0 |
| Decoy285 | O(CCCC)c1ccc(cc1)C(=O)N1CCN(CC1)c1nc(nc2n(ncc12)-c1ccccc1)CC(C)C | 0 |
| Decoy286 | S1\C(=C/c2cn(nc2-c2cc([N+](=O)[O-])ccc2)-c2ccccc2)\C(=O)N(C[C@H](CCCC)CC)C1=S | 0 |
| Decoy287 | O(CCCC)c1ccc(cc1)C(=O)N1CCN(CC1)c1nc(nc2n(ncc12)-c1ccccc1)[C@H](CCC)C | 0 |
| Decoy288 | O(Cc1nc2c(n1CCc1ccccc1)cccc2)c1c2c(ccc1)cccc2 | 0 |
| Decoy289 | S\1c2n(nc(n2)[C@@H]2Oc3c(OC2)cccc3)C(=O)/C/1=C/c1oc(cc1)-c1ccc(cc1)C(=O)C | 0 |
| Decoy290 | Ic1ccc(cc1NC(=O)c1sc(Cl)cc1)C(F)(F)F | 0 |
| Decoy291 | S=C1NC(=O)C(=CNc2ccc(cc2)C(C)(C)C)C(=O)N1 | 0 |
| Decoy292 | S(=O)(=O)(Nc1cc(N)ccc1C)N1C[C@H](CCC1)C | 0 |
| Decoy293 | S1C2=C(CCCC2)[C@H](C(=O)N)[C@@H]1NC(=O)CCC=1C[NH+]=C2C=1C=CC=C2 | 0 |
| Decoy294 | O1Cc2cc(NC(=O)NC[C@H](O)c3ccc(cc3)C(C)(C)C)ccc2C1 | 0 |
| Decoy295 | S(=O)(=O)(NNC(=O)C1=NN(C(C)C)C(=O)c2c1cccc2)c1cc(ccc1C)C | 0 |
| Decoy296 | FC(F)(F)c1cc(ccc1)C(=O)n1c2c([nH+]c1[C@@H](O)C)cccc2 | 0 |
| Decoy297 | Ic1c(nc(nc1NCC)CC[NH+](CC)CC)C(C)(C)C | 0 |
| Decoy298 | Brc1cc2[nH]cc(c2cc1)C(=O)c1cc(N)ccc1F | 0 |
| Decoy299 | Oc1cc(ccc1)C=Nn1cnnc1N\N=C\c1cc(O)ccc1 | 0 |
| Decoy300 | Clc1cc(F)ccc1NC(=O)[C@H](Sc1[nH+]cc2n1C=CC=C2)C | 0 |
| Decoy301 | FC(F)(C(F)(F)O[C@](F)(C(F)(F)O[C@](F)(C(=O)NCCCCCCCCCC)C(F)(F)F)C(F)(F)F)C(F)(F)F | 0 |
| Decoy302 | Fc1cc(-c2ncnc(N3CCN(CC3)C(=O)Nc3cc(C)c(cc3)C)c2)c(OC)cc1 | 0 |
| Decoy303 | Clc1c(S(=O)(=O)N2CCN(CC2)C=2n3ncnc3N=C(C=2)C(F)(F)F)cccc1Cl | 0 |
| Decoy304 | Clc1cc2nc(n(c2cc1F)C1CCC(O)CC1)N | 0 |
| Decoy305 | [NH2+]1C[C@H]([NH+](C[C@H]1CC(C)C)Cc1ncnn1C(C)C)CC | 0 |
| Decoy306 | Fc1ccc(F)cc1NC(=O)Nc1ccc(cc1)[C@H]1[NH+]=C2C(C=C(F)C=C2)=C1 | 0 |
| Decoy307 | O=C(N([C@H](C)c1cc2c(cc1)cccc2)C1CC1)N[C@@H](C)c1cc[nH+]cc1 | 0 |
| Decoy308 | O=C(NCCNc1nnc(Nc2[nH+]cc(cc2)C)cc1)c1ccc(cc1C)C | 0 |
| Decoy309 | S(CC(=O)Nc1c2c(ccc1)C(O)=NNC2=O)c1ncccc1 | 0 |
| Decoy310 | O[C@H](C)c1nc2cc(N)ccc2n1Cc1nc(ncc1)C | 0 |
| Decoy311 | O1[C@H](CO)[C@@H](O)[C@H](O)[C@@H](O[C@@H]2O[C@H](CO)[C@@H](O)[C@H](O)[C@H]2O)[C@@H]1O[C@H]1CC[C@@]2([C@@H]3[C@](CC[C@H]2C1(C)C)(C)[C@@]1(CC[C@@]2([C@H](C1=CC3=O)C[C@](CC2)(C(=O)[O-])C)C)C)C | 0 |
| Decoy312 | S(C)c1cc2nc(OCC(C)C)c(cc2cc1)C[NH2+][C@H](C)c1occc1 | 0 |
| Decoy313 | Brc1cn(nc1NS(=O)(=O)c1cc([N+](=O)[O-])ccc1)Cc1ccc(Cl)cc1Cl | 0 |
| Decoy314 | O1[C@H](CO[C@@H]2O[C@@H](C)[C@H](O)[C@@H](O)[C@H]2O)[C@@H](O)[C@H](O)[C@@H](O)[C@@H]1OC[C@@]1(Oc2c(C=C1)c(O)c1c(OC=C(C1=O)c1ccc(O)cc1)c2)C | 0 |
| Decoy315 | FC(F)(F)c1ccc(Nc2ncnc(Oc3c4[nH+]c(ccc4ccc3)C)c2N)cc1 | 0 |
| Decoy316 | Brc1ccsc1C(=O)N[C@@H]1[C@H]2OCC[C@@H]2[C@@H]1[NH3+] | 0 |
| Decoy317 | O(Cc1nc-2n(n1)C=Nc1n(CCO)c(c(c1-2)-c1ccccc1)-c1ccccc1)c1ccccc1OC | 0 |
| Decoy318 | Brc1oc(cc1)-c1[nH+][nH]c(N)c1-c1cc(C)c(cc1)C | 0 |
| Decoy319 | O=C1N(CCC[C@@]1(O)C[NH+](CCc1ccccc1)C)Cc1ccc(cc1)C(C)C | 0 |
| Decoy320 | O=C(N1C[C@@]2(CCC[NH+](C2)CC2CCCCC2)CC1)CCc1[nH]nc(C)c1C | 0 |
| Decoy321 | Brc1c(n[nH]c1C(=O)NNc1nc(cc(n1)C)C)C | 0 |
| Decoy322 | Clc1cc(ccc1Cl)CSC=1NC(=O)C=C(N=1)C | 0 |
| Decoy323 | S(CC(=O)Nc1cc2OC3(Oc2cc1)CCCCC3)c1[nH+]c(N)c(cn1)C#N | 0 |
| Decoy324 | S\1c2c(N(CC(OCC)=O)/C/1=N/C(=O)c1ccc(S(=O)(=O)N(CC=C)CC=C)cc1)cc(cc2C)C | 0 |
| Decoy325 | S(=O)(=O)(C)c1cc(NC(=O)N2C[C@H](CCC2)CCC)ccc1C | 0 |
| Decoy326 | S(=O)(=O)(CNC(=O)[C@@H](N(CCCCCC)C(=O)\C=C/CCCCC)\C=C\CCC)c1ccc(cc1)C | 0 |
| Decoy327 | Brc1nc(sc1)N[C@@H]1[C@H]2OCCC[C@@H]2[C@H]1[NH3+] | 0 |
| Decoy328 | s1c(nnc1SCc1ccccc1F)N1[C@H](C(C(=O)c2ccc(OCCCC)cc2)=C([O-])C1=O)c1cc(OCC)c(OCCCC)cc1 | 0 |
| Decoy329 | [NH2+]([C@H](Cn1ncc(c1)C)C)Cc1ccc(-n2nccc2)cc1 | 0 |
| Decoy330 | s1c(C)c(cc1C)[C@H](NC(=O)N[C@@H](C)c1[nH+]c2c([nH]1)cccc2)C | 0 |
| Decoy331 | Clc1cc(cc(OCC)c1OCC)C[NH2+]CC=1NC=C(C)C(=O)C=1C | 0 |
| Decoy332 | Ic1cc2nc(n(c2cc1F)CC([NH+](C)C)(C)C)N | 0 |
| Decoy333 | ClC=1C=Cc2[nH+]c(cn2C=1)CNC(=O)Nc1ccc(cc1)C(=O)N[C@H](CC)C | 0 |
| Decoy334 | Brc1cc(cc(OCC)c1OCc1ccccc1F)C[NH2+]C[C@H](O)C | 0 |
| Decoy335 | O=C(N1C[C@@H]2[NH+](C[C@@H]1C)CCCC2)[C@@H](/C(=N\O)/N)CCC | 0 |
| Decoy336 | O(C)c1cc(OC)c(OC)cc1C[NH2+]CC(N[C@@H](C)c1ccccc1)(C)C | 0 |
| Decoy337 | S(OC[C@]1(O[C@@]2(OC(O[C@]2(C)[C@@]2(OC(O[C@@]12C)(C)C)C)(C)C)C)C)(=O)(=O)c1ccc(cc1)C | 0 |
| Decoy338 | s1cccc1-c1nn(nn1)CC(=O)N([C@@H](C(=O)NC1CCCC1)c1ccc(N(C)C)cc1)c1cccc(C)c1C | 0 |
| Decoy339 | s1c(ccc1C)[C@@H]1[NH+]=C([O-])c2c3CC[C@H](Cc3sc2N1)C(C)(C)C | 0 |
| Decoy340 | O=C1N(CC(C)C)C(=O)c2c1c(c1c(c2)cccc1)-c1ccccc1 | 0 |
| Decoy341 | S(CC(=O)Nc1ccccc1C(=O)Nc1ccccc1)c1[nH+]c2c([nH]1)cc(cc2)C | 0 |
| Decoy342 | [nH+]1c2c(n(c1)-c1ncnc(Nc3ccc(cc3C)C)c1N)cccc2 | 0 |
| Decoy343 | o1ccc(C(=O)NN)c1C[NH+]1CC[NH+]2[C@H](CCCC2)C1 | 0 |
| Decoy344 | s1c2c(CC[C@@H](C2)C)c(C(OC(C)C)=O)c1NC(=O)c1nn2c(N=C(C=C2C(F)(F)F)c2ccc(OC)cc2)c1 | 0 |
| Decoy345 | Ic1ccc(cc1)C[C@H]([NH2+]CCC)C[C@@H](COC)C | 0 |
| Decoy346 | O=C(N[C@H]1CC[C@H]1[NH2+]C1Cc2c(C1)cccc2)C=1C=Nc2n(ncn2)C=1[O-] | 0 |
| Decoy347 | Fc1cc(ccc1F)[C@@H](NC(=O)c1cc2c(nc1)NC(=O)NC2=O)C | 0 |
| Decoy348 | s1ccc(C)c1[C@@H](Nc1cc2OC(F)(F)Oc2cc1)C | 0 |

**Table S4.** Structures of the 145 Compounds (in SMILE format) from the test set together with their activities (1 or 0) for H_2_O_2_-induced models.

| ChEMBL ID | Structure | Activity |
| --- | --- | --- |
| CHEMBL1938467 | S1c2cc([N+](=O)[O-])ccc2N(NC(=O)CC[NH+]2CC[NH+](CC2)C)c2c1cccc2 | 1 |
| CHEMBL2036260 | S(C(=O)C)CCC(=O)NCCCCNc1c2CCCCc2[nH+]c2c1cccc2 | 1 |
| CHEMBL1938451 | S1c2c(N(NC(=O)C[NH+](C)C)c3c1cccc3)cccc2 | 1 |
| CHEMBL2012545 | O1c2c(OC1)cc1c(-c3c(cc(OC)c(OC)c3OC)[C@H](OC(=O)C)[C@@H](C)[C@@H](C)[C@H]1OC(=O)\C(=C\C)\C)c2OC | 1 |
| CHEMBL482012 | s1cc(nc1NC(=O)c1ccncc1)-c1ccccc1 | 1 |
| CHEMBL1782122 | O1c2c(OC1)cc1c(-c3c(cc(OC)c(OC)c3OC)[C@H](OC(=O)\C(=C/C)\C)[C@@H](C)[C@@H](C)[C@H]1OC(=O)CCC)c2OC | 1 |
| CHEMBL1080814 | Brc1cc(sc1)C(=O)N[C@@H](CCC(=O)NCCC1CC[NH+](CC1)Cc1ccccc1)C(OCCCCCC)=O | 1 |
| CHEMBL516766 | s1c2CCCCc2nc1NC(=O)\C=C\c1cc(OC)cc(OC)c1 | 1 |
| CHEMBL453572 | Fc1ccc(cc1)C1C2=C(NC(N)=C1C#N)CC(CC2=O)(C)C | 1 |
| CHEMBL1082082 | O(CCCCCC)C(=O)[C@@H](NC(OCc1ccccc1)=O)CCC(=O)NCCC1CC[NH+](CC1)Cc1ccccc1 | 1 |
| CHEMBL1938460 | Clc1cc2Sc3c(N(NC(=O)C[NH+]4CCCC4)c2cc1)cccc3 | 1 |
| CHEMBL1938466 | S1c2cc(OC)ccc2N(NC(=O)CC[NH+]2CC[NH+](CC2)C)c2c1cccc2 | 1 |
| CHEMBL219405 | O(C(=O)C=1C(c2c([nH+]c3c(CCCC3)c2N)NC=1C)c1ccc([N+](=O)[O-])cc1)CC | 1 |
| CHEMBL1938452 | Clc1cc2N(NC(=O)C[NH+](C)C)c3c(Sc2cc1)cccc3 | 1 |
| CHEMBL2036258 | S(C(=O)C)CCC(=O)NCCCNc1c2CCCCc2[nH+]c2c1cccc2 | 1 |
| CHEMBL461076 | Fc1ccc(cc1)C1C(C(OCC)=O)=C(NC(N)=C1C#N)C | 1 |
| CHEMBL1782124 | O1c2c(OC1)cc1c(-c3c(cc(OC)c(OC)c3OC)C[C@H](C)[C@H](C)[C@H]1O)c2OC | 1 |
| CHEMBL1082081 | O(CCCCCC)C(=O)[C@@H](NC(OC(C)(C)C)=O)CCC(=O)NCCC1CC[NH+](CC1)Cc1ccccc1 | 1 |
| CHEMBL1782121 | O1c2c(OC1)cc1c(-c3c(cc(OC)c(OC)c3OC)[C@H](OC(=O)\C(=C/C)\C)[C@@H](C)[C@@H](C)[C@H]1OC(=O)C(C)C)c2OC | 1 |
| CHEMBL50 | O1c2c(C(=O)C(O)=C1c1cc(O)c(O)cc1)c(O)cc(O)c2 | 1 |
| CHEMBL452100 | s1cc(nc1NC(=O)\C=C\c1ccccc1)-c1ccccc1 | 1 |
| CHEMBL1938459 | Clc1cc2N(NC(=O)C[NH+]3CCCC3)c3c(Sc2cc1)cccc3 | 1 |
| CHEMBL461075 | O(C(=O)C=1C(C(C#N)=C(NC=1C)N)c1ccccc1)CC | 1 |
| CHEMBL516955 | O(C(=O)C=1C(C(C#N)=C(NC=1C)N)c1ccccc1[N+](=O)[O-])CC | 1 |
| CHEMBL1163575 | Clc1ccc(cc1)-c1nc(SCCC)nnc1-c1ccc(Cl)cc1 | 1 |
| CHEMBL1782115 | O1c2c(OC1)cc1c(-c3c(cc(OC)c(OC)c3OC)[C@H](OC(=O)C)[C@@H](C)[C@@H](C)[C@H]1OC(=O)CC)c2OC | 1 |
| CHEMBL1938457 | Clc1cc2Sc3c(N(NC(=O)C[NH+]4CCCCC4)c2cc1)cccc3 | 1 |
| CHEMBL488100 | O1c2c(OC1)cc1c(-c3c(cc(OC)c(OC)c3OC)[C@H](OC(=O)\C(=C/C)\C)[C@@H](C)[C@@H](C)[C@H]1OC(=O)C)c2OC | 1 |
| CHEMBL460667 | O(C(=O)C=1C(C(C#N)=C(NC=1C)N)c1ccc([N+](=O)[O-])cc1)CC | 1 |
| Decoy001 | O1CC[C@](CC1C(C)C)(CCC(C)C)CC[NH2+]Cc1ccc(N(C)C)cc1 | 0 |
| Decoy002 | O1CCN(CC1)c1ccc(cc1)CNCC([NH2+][C@@H](C)c1ccccc1)(C)C | 0 |
| Decoy003 | Clc1ccc(OP(=S)(N2CCOCC2)c2ccccc2)cc1 | 0 |
| Decoy004 | Clc1cc(ccc1)C=1C2=NC=C3[C@H](N2NC=1C)C=CN(C3=O)c1nc(SC)[nH]n1 | 0 |
| Decoy005 | s1c2cc(OCC)ccc2nc1N1[C@@H](C(C(=O)c2cc3C[C@H](Oc3cc2)C)=C([O-])C1=O)c1cc(OCCC)ccc1 | 0 |
| Decoy006 | S(=O)(=O)(c1c(nn(c1OC(=O)c1cc(OCC)c(OCC)cc1)-c1ccccc1)C)c1ccc(cc1)C | 0 |
| Decoy007 | Brc1cc(Cl)c(S(=O)([O-])=N[NH3+])cc1 | 0 |
| Decoy008 | Brc1cc(F)c([C@@H](NN)c2cc(ccc2)C)c(F)c1 | 0 |
| Decoy009 | FC(F)(F)c1ccc(cc1)[C@H](N(C(=O)C=C(C)C)CCCCCC)C(=O)NC(CC(C)(C)C)(C)C | 0 |
| Decoy010 | O(C(=O)C=1[C@H](C)C(=NC=1C)\C=N\c1ccc(cc1)Cc1ccc(\N=C\C=2N=C(C)[C@@H](C(OC)=O)C=2C)cc1)C | 0 |
| Decoy011 | S(=O)(=O)([O-])c1cc(\N=C(/[O-])\C2=C\C(=N/c3ccc([NH+](CC)CC)cc3)\c3c(cccc3)C2=O)ccc1CCCCCCCC | 0 |
| Decoy012 | Brc1cc(oc1N1CCCC1)\C=[NH+]\c1ccc(Cl)cc1O | 0 |
| Decoy013 | O(C(C)C)C[C@@H](O)C[NH+](CCC)CC(=O)N(Cc1ccc(cc1)C(C)(C)C)CCc1c2c([nH]c1)cccc2 | 0 |
| Decoy014 | O1CC[NH+](CC1)C1(CCCC1)CC(=O)Nc1cc2c([nH]nc2C)nc1 | 0 |
| Decoy015 | O(Cc1ccc(cc1)C)c1c(cc(cc1OCC)\C=N\NC(=O)CC[C@@H](O)c1ccc(OCC)cc1)CC=C | 0 |
| Decoy016 | Clc1cc(Cl)ccc1[C@@H]1N2NC=[NH+]C2=NC(=C1)c1ccc(cc1)C | 0 |
| Decoy017 | s1c2c(nc1N(C(=O)c1ccc(S(=O)(=O)N(CCC)CCC)cc1)C[C@H]1OCCC1)cc(OC)cc2 | 0 |
| Decoy018 | S(CC(=O)Nc1ccc(cc1)-c1[nH+]c2n(c1)C=CC=C2)c1ccc(F)cc1 | 0 |
| Decoy019 | s1cc(nc1N1CCC[NH+](CC1)Cc1cn[nH]c1-c1cc(ccc1)C)C | 0 |
| Decoy020 | S(=O)(=O)(N1CCN(CC1)c1ccccc1F)C=1c2c(cccc2)C(=O)N(C=1)CC(=O)N(CC)c1cc(C)c(cc1)C | 0 |
| Decoy021 | s1c2CCCCc2c2c1N=C(SCCC(C)C)N(C1CCCCC1)C2=O | 0 |
| Decoy022 | S\1C=2N([C@@H](C(C(OC)=O)=C(N=2)C)c2ccc(OC(=O)c3occc3)cc2)C(=O)/C/1=C/1\c2c(N(CC)C\1=O)cccc2 | 0 |
| Decoy023 | FC(F)(F)c1nc(N[C@@H]2[C@H]3OCC[C@@H]3[C@@H]2[NH3+])ccc1 | 0 |
| Decoy024 | Clc1cc(c2OCOCc2c1)COC(=O)CNc1ccc(cc1[N+](=O)[O-])C(F)(F)F | 0 |
| Decoy025 | s1c2cc(OC)ccc2nc1N(C(=O)c1ccc(S(=O)(=O)N2CCC(CC2)C)cc1)C[C@H]1OCCC1 | 0 |
| Decoy026 | Clc1ccccc1NC(=O)c1sc2[nH+]c3cc(ccc3cc2c1N)C | 0 |
| Decoy027 | O(c1ccccc1OC)c1n(nc(CC)c1C[NH+](CCC)C[C@@H](O)COCC)-c1ccc(OC)cc1 | 0 |
| Decoy028 | FC(F)(F)[C@]([NH3+])(C)c1[nH]c2ccc(nc2n1)N(C)C | 0 |
| Decoy029 | s1cccc1-c1nc2S\C(=C\c3cn(nc3-c3ccc(OCCC(C)C)cc3)-c3ccccc3)\C(=O)n2n1 | 0 |
| Decoy030 | o1nc(nc1CN1C(=O)[C@](NC1=O)(c1cc(C)c(cc1)C)c1ccccc1)C(C)C | 0 |
| Decoy031 | s1c2CCCCCc2c2c1nc(nc2N)CSc1nnc(n1C)N1CCOCC1 | 0 |
| Decoy032 | Clc1cc([N+](=O)[O-])ccc1C=1OC(=O)/C(/N=1)=C/c1ccc(OCc2ccccc2)cc1OCc1ccccc1 | 0 |
| Decoy033 | FC(F)(F)c1ccc(Nc2[nH+]cnc(N3CCCCCC3)c2N)cc1 | 0 |
| Decoy034 | s1c2c(cc1)[C@H](N(CC2)Cc1[nH+]c(nc(n1)N)Nc1ccc(F)cc1)CC | 0 |
| Decoy035 | Clc1cc(Nc2[nH+]cnc(N3C[C@@H](CCC3)C)c2N)ccc1F | 0 |
| Decoy036 | O(C[C@@H](O)Cn1c2c([nH+]c1NCc1ccccc1)cccc2)c1ccccc1 | 0 |
| Decoy037 | O(c1n(nc(C)c1C[NH+](CCOC)C[C@H](O)COC(C)(C)C)-c1ccccc1)c1ccc(OC)cc1 | 0 |
| Decoy038 | Brc1cc2NC(=O)Nc2cc1[C@@H]([NH2+]CCC)[C@@H](CC)C | 0 |
| Decoy039 | O(C(C)C)c1ccc(cc1)CNC(=O)C1CCN(CC1)C1=Nn2c(nnc2-c2cc(ccc2)C)C=C1 | 0 |
| Decoy040 | O=C(N(C)C)c1cc2[nH+]c(n(c2cc1)C)CN(C(=O)Nc1cc(ccc1C)C)C | 0 |
| Decoy041 | S(=O)([O-])(=Nc1cc2c(cc1)cccc2)c1cc([nH+]cc1)N | 0 |
| Decoy042 | s1cccc1-c1nn(nn1)CC(=O)N([C@@H](C(=O)NC1CCCC1)c1ccc(OCC)cc1)c1c2CCCCc2ccc1 | 0 |
| Decoy043 | s1c2c(nc1N(C(=O)C1CCN(S(=O)(=O)c3ccc(OC)cc3)CC1)CC[NH+](C)C)c(cc(c2)C)C | 0 |
| Decoy044 | Clc1c2NC(=CC(=O)c2ccc1)CS[C@@H]1[NH+](C)[C@H](NN1)c1cc(ccc1)C | 0 |
| Decoy045 | S\1c2n(nc(n2)\C=C\c2cc(OC)c(OCC)cc2)C(=O)/C/1=C/c1oc(cc1)-c1cc([N+](=O)[O-])ccc1 | 0 |
| Decoy046 | Clc1c(n(nc1C)C)CC1(CCC(CC1)C(C)C)C#N | 0 |
| Decoy047 | O(CC)c1ccccc1NC(=O)CN1c2c(n(c3c2cc(cc3)C)C)C(=O)N(c2cc(ccc2)C)C1=O | 0 |
| Decoy048 | O=C(Nc1ccc(cc1)C(C)C)[C@H]1[NH2+]Cc2nc[nH]c2C1 | 0 |
| Decoy049 | Clc1cc(NC(=O)c2cc(S(=O)(=O)N)oc2C)ccc1Cl | 0 |
| Decoy050 | Clc1cccc(NC(=O)NC[C@H](c2c3c([nH]c2)cccc3)c2ccc[nH+]c2)c1C | 0 |
| Decoy051 | s1c(ccc1CNC(=O)[C@@H]1CCOC1)C#CC[NH3+] | 0 |
| Decoy052 | Ic1cc([NH3+])ccc1N[C@H]1CCOc2c1cccc2 | 0 |
| Decoy053 | o1c2c(cc1[C@H]1N=NC=C1C[NH2+][C@@H]1CCc3c1cccc3)cccc2 | 0 |
| Decoy054 | O1c2c(cc(cc2)C(=O)C=2[C@@H](N(CC[NH+]3CCOCC3)C(=O)C=2[O-])c2cc(OCC)c(OCCCCC)cc2)C[C@@H]1C | 0 |
| Decoy055 | S(=O)(=O)(N[C@H](CCCC)C(Oc1cc2OC(=O)C=C(c2cc1)c1ccc(OC)cc1)=O)c1ccc(cc1)C | 0 |
| Decoy056 | Brc1ccc(cc1)C1=NN(Cc2onc(n2)-c2cc(OC)c(OC)c(OC)c2)C(=O)C=C1 | 0 |
| Decoy057 | S(=O)(=O)(Nc1ccccc1-c1nnn[n-]1)NCc1ccccc1 | 0 |
| Decoy058 | Clc1cccc(Cl)c1CO\N=C\c1c2c(ccc1OCC=C)cccc2 | 0 |
| Decoy059 | Ic1cc([NH3+])c(N[C@H]2CCC[C@H]2OC)cc1F | 0 |
| Decoy060 | Fc1cccc(F)c1N1C[C@@H]([NH2+]Cc2n3c(nc2)C(=CC=C3)C)CC1 | 0 |
| Decoy061 | Brc1cc\2c(NC(=O)/C/2=N\Nc2nc(cc(n2)C)C)cc1 | 0 |
| Decoy062 | S(=O)(=O)(N(CC(=O)N\N=C\c1ccc(cc1)C(C)(C)C)c1ccc(F)cc1)c1cc2OCCOc2cc1 | 0 |
| Decoy063 | S(=O)(=O)(Nc1cc(N)c(cc1)C)N1C[C@@H](C[C@H](C1)C)C | 0 |
| Decoy064 | S([C@H](C(=O)NC=1C(=O)N(N(C)C=1C)c1ccccc1)C)c1oc(nn1)-c1ccc(cc1)C(C)(C)C | 0 |
| Decoy065 | S(=O)(=O)(Nc1cc(ccc1)CC)c1cn(nc1N)CC | 0 |
| Decoy066 | O(CCCC)c1ccc(cc1)C(=O)[C@@H](CC[C@@H](C(=O)c1ccc(OCCCC)cc1)C[NH2+]CC)C[NH2+]CC | 0 |
| Decoy067 | Clc1c(n(nc1C)C)CN1C[C@@]([NH2+]C[C@]1(CC)C)(CC)C | 0 |
| Decoy068 | O1C[C@@H](CC1)C[NH+]1Cc2c([nH]nc2C(c2ccccc2)c2ccccc2)CC1 | 0 |
| Decoy069 | O([C@@H]([C@@H](C\C=C\C=C)C)[C@H]([NH+](C)C)C(=O)CCCCCCC(=O)[C@@H](NC(=O)[C@@H]([NH2+]C)CC(C)C)C(C)C)C(=O)C | 0 |
| Decoy070 | Clc1cc(N(S(=O)(=O)c2ccccc2)C(=O)CCCN2C(=O)c3c(cccc3)C2=O)ccc1OC | 0 |
| Decoy071 | Clc1c(n(nc1C)C)C[C@@H]1C[C@H](CC[C@@H]1O)C(C)(C)C | 0 |
| Decoy072 | o1cccc1[C@H]([NH+](C)C)CNC[C@@H](O)COc1ccc(cc1C)C(C)(C)C | 0 |
| Decoy073 | s1c(C)c(nc1Nc1ncnc(Sc2ccc(cc2)C)c1N)C | 0 |
| Decoy074 | O1CC(=CC1=O)[C@H]1CC[C@@]2(O)[C@H]3[C@H](CC[C@@]12C)[C@@]1([C@H](C[C@@H](O[C@H]2O[C@@H](CO[C@H]4O[C@@H](CO)[C@@H](O)[C@@H](O)[C@H]4O)[C@@H](O)[C@@H](O)[C@H]2O)CC1)CC3)C | 0 |
| Decoy075 | Clc1cc(N)c(cc1)[C@@H](O)[C@@H]1CC2(OCC1)CCC2 | 0 |
| Decoy076 | Ic1ccc(Br)cc1C(=O)Nc1ccc(F)c(F)c1F | 0 |
| Decoy077 | Fc1ccccc1[C@H](NC(=O)[C@H]1[C@H](C=C(C)C)C1(C)C)c1[nH+]ccn1C | 0 |
| Decoy078 | O1CCC(NC(=O)N2C[C@@H](CCC2)C(=O)NC2CCCCC2)CC1 | 0 |
| Decoy079 | Clc1cc(NC(=O)[N-]NC(=O)c2cc3[nH+]c(n(c3cc2)CC)C)ccc1Cl | 0 |
| Decoy080 | S(Cc1ccc(cc1)C)c1nc2N(C)C(=O)NC(=O)c2n1CCCCCCCCCCCC | 0 |
| Decoy081 | O=C1N(c2n(-c3c1cccc3)c(nn2)CCC(=O)N1C[C@@H](N(CC1)c1cc(ccc1)C)C)CCc1ccccc1 | 0 |
| Decoy082 | s1c2cc(ccc2nc1N(C(=O)c1cc([N+](=O)[O-])c(N2CCOCC2)cc1)C[C@@H]1OCCC1)C | 0 |
| Decoy083 | [nH]1c2c(nc1[C@H]1CCCN(C1)c1ncnc3[nH]ccc13)c(ccc2)C | 0 |
| Decoy084 | Clc1cc(N)c(cc1)[C@H](O)[C@@H]1CCc2c1nccc2 | 0 |
| Decoy085 | Fc1cc(ccc1C)[C@@H](NC(=O)Nc1cccc(NC(=O)C)c1C)C | 0 |
| Decoy086 | O1CC[C@@H](CC12CCCC2)[C@H](NN)C1C(C)(C)C1(C)C | 0 |
| Decoy087 | S=C(N)C1(CCCC1)C(=O)N[C@H]1CC[C@H](C[C@H]1C)C | 0 |
| Decoy088 | S(=O)(=O)(Nc1cc(C)c([NH+]2CCCC2)cc1)c1ccc(F)cc1C | 0 |
| Decoy089 | O=C1Nc2cc(N[C@H](C)c3nnnn3-c3ccccc3)ccc2CC1 | 0 |
| Decoy090 | Fc1c2c(N[C@@H](N(C2=O)c2ccccc2)[C@@H](NC2=[NH+]C=N[C@@H]3N=CN=C23)CC)ccc1 | 0 |
| Decoy091 | s1c2N=C(NC(=O)c2c(-c2ccc(cc2)C)c1C)CSc1[nH+]cccc1 | 0 |
| Decoy092 | Brc1sc(cc1)[C@H](NN)c1ccc(OCCC)cc1 | 0 |
| Decoy093 | S=P1(OCC(CO1)(C)C)O[C@H]1[C@@H]2OC[C@H](OP3(=S)OCC(CO3)(C)C)[C@@H]2OC1 | 0 |
| Decoy094 | ClC=1C=CC2=[NH+]CC(=C2C=1)CCNC(=O)C(=O)Nc1c2c(ccc1)c(O)ccc2 | 0 |
| Decoy095 | S(CC(=O)N1CCc2c1cccc2)C1=NC(=O)c2c(n(nc2)-c2cc(C)c(cc2)C)N1 | 0 |
| Decoy096 | O1c2c(c(O)c([C@@H]3O[C@H](C)[C@@H](O)C(=O)[C@@H]3O[C@@H]3O[C@H](C)[C@H](O)[C@H](O)[C@@H]3O)c(O)c2)C(=O)C=C1c1cc(O)c(O)cc1 | 0 |
| Decoy097 | O=C1N(CCC(=O)NC1(C)C)[C@@H]1CC[C@H](CC)[C@H]1C | 0 |
| Decoy098 | o1c(C)c(cc1C)[C@@H](NC(=O)N[C@H](C(=O)NCc1occc1)C)C | 0 |
| Decoy099 | s1c(nnc1N1[C@@H](C2=C(Oc3c(cccc3)C2=O)C1=O)c1cc(OCC)c(OCCC(C)C)cc1)CC | 0 |
| Decoy100 | S=C1N(C(=N[N-]1)CNC(=O)Nc1c(cc(cc1C)C)C)C1CC1 | 0 |
| Decoy101 | Clc1ccccc1COc1ccc(cc1OCC)\C=C\1/N=C(OC/1=O)c1cc([N+](=O)[O-])c(Cl)cc1 | 0 |
| Decoy102 | Ic1cc(N)c(N[C@@H]2CCCOC2)cc1 | 0 |
| Decoy103 | s1c2c(S[C@H]3[C@@H]([C@H]4[C@H]5[C@H]([C@@H]3C4)C(=O)N(c3ccc(cc3)C)C5=O)[C@@H]2c2ccc(O)cc2)nc1[S-] | 0 |
| Decoy104 | Fc1cc(ccc1)[C@H]1N(CCC1)C(=O)Nc1ccc([NH+]2CCOCC2)cc1C | 0 |
| Decoy105 | [O-]c1c2c(ccc1[C@@H]([NH+]1C[C@@H](C[C@H](N)C1)C)C)cccc2 | 0 |
| Decoy106 | S(=O)(=O)(CNC(=O)[C@H](N(C(=O)CC(C)C)CCCCCC)C(CC)CC)c1ccc(cc1)C | 0 |
| Decoy107 | O1CC[NH+](CC1)CCCN1[C@@H](C2=C(Oc3c(cc(cc3)C)C2=O)C1=O)c1cc(OCC)c(OCCCC)cc1 | 0 |
| Decoy108 | Oc1cc(ccc1)[C@H](CC(=O)N1CCc2[nH]ncc2C1)c1ccc(O)cc1 | 0 |
| Decoy109 | Clc1cc(N(S(=O)(=O)c2cc(OC)c(OC)cc2)CC(=O)N2CCN(CC2)c2cc(Cl)ccc2)c(OC)cc1 | 0 |
| Decoy110 | O(CCCCn1c2c(nc1CCCNC(=O)C(CC)CC)cccc2)c1cc(ccc1C(C)C)C | 0 |
| Decoy111 | BrC=1C=Cc2[nH+]cc(n2C=1)C[NH+]1C[C@@H](Nc2ccccc2)CC1 | 0 |
| Decoy112 | S1C=2N(N=C1N1CCC(CC1)C(=O)N1CC[NH+](CC1)C1CCCCC1)C(=O)C=C(N=2)CCC | 0 |
| Decoy113 | O(CCCn1c2c(nc1-c1ccc(cc1)C)cccc2)c1c(cccc1C)C | 0 |
| Decoy114 | Brc1cc(F)c(cc1)[C@H]1[NH+]=C(N(CC(C)C)C1=[NH2+])C | 0 |
| Decoy115 | Clc1nc(C)c(nc1NCCS(=O)(=O)N)C | 0 |
| Decoy116 | S(=O)(=O)(Nc1cc(ccc1)[C@H](NC(=O)c1c2[nH]ncc2ccc1)C)C | 0 |

**Table S5. Molecular descriptors used in this work.**

| No. | Descriptor class | Number of descriptors | Descriptors |
| --- | --- | --- | --- |
| 1 | DS 2D | 26 | ES_Count_aaS, ES_Count_dsN, ES_Count_sBr, ES_Count_sOH, ES_Count_ssC_2_, ES_Count_ssssC, ES_Sum_aaN, ES_Sum_ssO, IsChiral, SAscore, SAscore_Complexity, Num_AliphaticSingleBonds, Num_AtomClasses, Num_ExplicitHydrogens, Num_H_Acceptors, Num_H_Donors, Num_RingAssemblies, Num_RingBonds, Num_RingFusionBonds, Num_Rings5, Num_Rings6, CHI_2, CIC, IAC_Mean, JY, Kappa_2_AM |
| 2 | MOE 2D | 50 | ast_violation, a_donacc, a_hyd, a_ICM, a_nBr, a_nN, a_nO, a_nS, BCUT_SLOGP_0, BCUT_SLOGP_1, BCUT_SLOGP_3, BCUT_SMR_0, BCUT_SMR_1, b_max1len, b_single, chi0v_C, density, GCUT_SLOGP_1, GCUT_SLOGP_2, GCUT_SLOGP_3, GCUT_SMR_1, Kier2, KierA1, KierA3, opr_brigid, opr_nrot, PEOE_PC+, PEOE_PC-, PEOE_RPC+, PEOE_RPC-, PEOE_VSA+1, PEOE_VSA+2, PEOE_VSA+4, PEOE_VSA-3, PEOE_VSA-4, PEOE_VSA-6, PEOE_VSA_FPOS, PEOE_VSA_POS, rsynth, SlogP_VSA0, SlogP_VSA1, SlogP_VSA3, SlogP_VSA9, SMR_VSA3, SMR_VSA5, SMR_VSA6, VAdjEq, VDistEq, vsa_acc, vsa_hyd |
| 3 | DS 2D and  MOE 2D | 65 | ast_violation, a_donacc, a_hyd, a_ICM, a_nN, a_nO, a_nS, BCUT_SLOGP_0, BCUT_SLOGP_1, BCUT_SLOGP_3, BCUT_SMR_0, BCUT_SMR_1, b_max1len, chi0v_C, chi1v, chiral_u, density, GCUT_SLOGP_1, GCUT_SLOGP_2, GCUT_SLOGP_3, GCUT_SMR_1, KierA1, PEOE_PC+, PEOE_PC-, PEOE_RPC+, PEOE_RPC-, PEOE_VSA+1, PEOE_VSA+2, PEOE_VSA+4, PEOE_VSA-3, PEOE_VSA-4, PEOE_VSA-6, PEOE_VSA_FNEG, PEOE_VSA_POS, rsynth, SlogP_VSA0, SlogP_VSA1, SlogP_VSA3, SlogP_VSA9, SMR_VSA3, SMR_VSA5, SMR_VSA6, VAdjEq, VDistEq, vsa_acc, vsa_hyd, ES_Count_dsN, ES_Count_ssssC, ES_Sum_aaN, ES_Sum_ssO, IsChiral, SAscore, SAscore_Complexity, Num_AliphaticSingleBonds, Num_H_Acceptors, Num_H_Donors, Num_RingAssemblies, Num_RingBonds, Num_RingFusionBonds, Num_Rings5, Num_Rings6, CIC, JY, Kappa_2_AM, SC_3_P |
| 4 | DS 2D | 32 | ES_Count_aaaC, ES_Count_aaCH, ES_Count_aaN, ES_Count_aasC, ES_Count_dssC, ES_Count_sCH3, ES_Count_sF, ES_Count_ssO, ES_Count_sssN, ES_Sum_aasC, ES_Sum_dssC, ES_Sum_sN_2_, IsChiral, QED_AROM, QED_HBA, QED_HBD, SAscore_Complexity, SAscore_Fragments, HBA_Count, Num_AliphaticDoubleBonds, Num_AromaticRings, Num_H_Acceptors, Num_Hydrogens, Num_RingAssemblies, Num_RingFusionBonds, Num_Rings5, Num_Rings6, Num_StereoBonds, Num_UnknownPseudoStereoAtoms, IAC_Mean, IC, JX |
| 5 | MOE 2D | 53 | a_acc, a_ICM, a_nN, a_nO, balabanJ, BCUT_PEOE_1, BCUT_PEOE_2, BCUT_SLOGP_2, BCUT_SLOGP_3, BCUT_SMR_0, b_1rotR, b_double, density, GCUT_PEOE_0, GCUT_PEOE_2, GCUT_SLOGP_0, GCUT_SLOGP_2, GCUT_SMR_2, PEOE_RPC+, PEOE_RPC-, PEOE_VSA+0, PEOE_VSA+1, PEOE_VSA+2, PEOE_VSA+3, PEOE_VSA+5, PEOE_VSA+6, PEOE_VSA-0, PEOE_VSA-2, PEOE_VSA-4, PEOE_VSA-5, PEOE_VSA-6, PEOE_VSA_FPOS, PEOE_VSA_NEG, PEOE_VSA_POS, petitjean, Q_RPC+, Q_VSA_FPOL, Q_VSA_FPOS, Q_VSA_FPPOS, Q_VSA_NEG, Q_VSA_POL, Q_VSA_PPOS, rsynth, SlogP_VSA0, SlogP_VSA1, SlogP_VSA2, SlogP_VSA5, SlogP_VSA6, SlogP_VSA7, SMR_VSA3, SMR_VSA5, SMR_VSA7, TPSA |
| 6 | DS 2D and  MOE 2D | 79 | a_nN, a_nO, BCUT_PEOE_1, BCUT_PEOE_2, BCUT_SLOGP_2, BCUT_SLOGP_3, BCUT_SMR_0, b_1rotN, b_double, density, GCUT_PEOE_0, GCUT_PEOE_2, GCUT_SLOGP_0, GCUT_SLOGP_2, GCUT_SMR_2, PEOE_RPC+, PEOE_RPC-, PEOE_VSA+0, PEOE_VSA+1, PEOE_VSA+2, PEOE_VSA+3, PEOE_VSA+5, PEOE_VSA+6, PEOE_VSA-0, PEOE_VSA-2, PEOE_VSA-4, PEOE_VSA-5, PEOE_VSA-6, PEOE_VSA_FNEG, PEOE_VSA_NEG, PEOE_VSA_POS, petitjean, Q_RPC+, Q_VSA_FHYD, Q_VSA_FPOS, Q_VSA_FPPOS, Q_VSA_NEG, Q_VSA_POL, Q_VSA_PPOS, rsynth, SlogP_VSA0, SlogP_VSA1, SlogP_VSA2, SlogP_VSA5, SlogP_VSA6, SMR_VSA3, SMR_VSA5, SMR_VSA7, TPSA, VAdjEq, ES_Count_aaaC, ES_Count_aaN, ES_Count_aasC, ES_Count_dssC, ES_Count_sCH3, ES_Count_sF, ES_Count_ssO, ES_Count_sssN, ES_Sum_aaaC, ES_Sum_dssC, ES_Sum_sN_2_, IsChiral, QED_AROM, QED_HBA, QED_HBD, SAscore_Complexity, SAscore_Fragments, HBA_Count, Num_AromaticRings, Num_H_Acceptors, Num_RingAssemblies, Num_RingFusionBonds, Num_Rings5, Num_Rings6, Num_StereoBonds, Num_UnknownPseudoStereoAtoms, IAC_Mean, IC, JX |
| 1–3#: neuroprotective models against ischemia-induced neurotoxicity (NIN models). | | | |
| 4–6#: neuroprotective models against H_2_O_2_ -induced neurotoxicity (NHN models) | | | |

**Table S6.** Performance of the 26 Bayesian classification models for the training set and test set using different combinational of output probabilities and fingerprints

| No. | Model | 5-Fold Cross-Validation Result | | | | | Validation Result Using External Test Set | | | | |
| --- | --- | --- | --- | --- | --- | --- | --- | --- | --- | --- | --- |
|  |  | SE | SP | PPV | MCC | AUC | SE | SP | PPV | MCC | AUC |
| 1 | NB-1 | 0.848 | 0.942 | 0.923 | 0.767 | 0.932 | 0.818 | 0.902 | 0.885 | 0.672 | 0.913 |
| 2 | NB-1-ECFP4 | 0.954 | 0.933 | 0.937 | 0.826 | 0.964 | 0.833 | 0.958 | 0.933 | 0.792 | 0.952 |
| 3 | NB-1-ECFP6 | 0.980 | 0.957 | 0.962 | 0.890 | 0.967 | 0.833 | 0.962 | 0.936 | 0.800 | 0.956 |
| 4 | NB-1-EPFP4 | 0.858 | 0.948 | 0.930 | 0.787 | 0.948 | 0.818 | 0.939 | 0.915 | 0.741 | 0.944 |
| 5 | NB-1-EPFP6 | 0.919 | 0.939 | 0.935 | 0.813 | 0.953 | 0.864 | 0.947 | 0.930 | 0.789 | 0.955 |
| 6 | NB-1-FCFP4 | 0.924 | 0.944 | 0.940 | 0.826 | 0.960 | 0.833 | 0.958 | 0.933 | 0.792 | 0.947 |
| 7 | NB-1-FCFP6 | 0.954 | 0.957 | 0.957 | 0.872 | 0.965 | 0.833 | 0.943 | 0.921 | 0.760 | 0.95 |
| 8 | NB-1-FPFP4 | 0.904 | 0.908 | 0.907 | 0.745 | 0.946 | 0.864 | 0.936 | 0.921 | 0.767 | 0.956 |
| 9 | NB-1-FPFP6 | 0.944 | 0.907 | 0.914 | 0.772 | 0.947 | 0.909 | 0.943 | 0.936 | 0.813 | 0.963 |
| 10 | NB-1-LCFP4 | 0.959 | 0.943 | 0.946 | 0.848 | 0.964 | 0.833 | 0.939 | 0.918 | 0.752 | 0.951 |
| 11 | NB-1-LCFP6 | 0.980 | 0.962 | 0.966 | 0.900 | 0.967 | 0.818 | 0.970 | 0.939 | 0.807 | 0.954 |
| 12 | NB-1-LPFP4 | 0.970 | 0.976 | 0.975 | 0.923 | 0.969 | 0.894 | 0.955 | 0.942 | 0.826 | 0.964 |
| 13 | NB-1-LPFP6 | 0.975 | 0.994 | 0.990 | 0.968 | 0.973 | 0.864 | 0.985 | 0.961 | 0.874 | 0.961 |
| 14 | NB-2 | 0.931 | 0.934 | 0.933 | 0.811 | 0.976 | 0.931 | 0.905 | 0.910 | 0.761 | 0.935 |
| 15 | NB-2-ECFP4 | 0.920 | 0.986 | 0.972 | 0.913 | 0.991 | 0.931 | 0.966 | 0.959 | 0.875 | 0.992 |
| 16 | NB-2-ECFP6 | 1.000 | 0.974 | 0.979 | 0.940 | 0.993 | 0.931 | 0.966 | 0.959 | 0.875 | 0.996 |
| 17 | NB-2-EPFP4 | 0.920 | 0.951 | 0.945 | 0.837 | 0.983 | 0.897 | 0.931 | 0.924 | 0.781 | 0.981 |
| 18 | NB-2-EPFP6 | 0.931 | 0.971 | 0.963 | 0.887 | 0.991 | 1.000 | 0.897 | 0.917 | 0.796 | 0.994 |
| 19 | NB-2-FCFP4 | 0.966 | 0.945 | 0.949 | 0.857 | 0.991 | 0.931 | 0.957 | 0.952 | 0.856 | 0.983 |
| 20 | NB-2-FCFP6 | 1.000 | 0.948 | 0.959 | 0.886 | 0.992 | 0.931 | 0.957 | 0.952 | 0.856 | 0.994 |
| 21 | NB-2-FPFP4 | 0.920 | 0.974 | 0.963 | 0.886 | 0.984 | 0.931 | 0.922 | 0.924 | 0.790 | 0.982 |
| 22 | NB-2-FPFP6 | 0.908 | 0.989 | 0.972 | 0.913 | 0.991 | 0.966 | 0.940 | 0.945 | 0.846 | 0.993 |
| 23 | NB-2-LCFP4 | 1.000 | 0.954 | 0.963 | 0.898 | 0.993 | 0.931 | 0.948 | 0.945 | 0.839 | 0.994 |
| 24 | NB-2-LCFP6 | 1.000 | 0.966 | 0.972 | 0.921 | 0.994 | 0.931 | 0.983 | 0.972 | 0.914 | 0.997 |
| 25 | NB-2-LPFP4 | 1.000 | 0.997 | 0.998 | 0.993 | 0.994 | 1.000 | 0.974 | 0.979 | 0.940 | 1.000 |
| 26 | NB-2-LPFP6 | 1.000 | 0.997 | 0.998 | 0.993 | 0.992 | 1.000 | 0.983 | 0.986 | 0.959 | 1.000 |
| 1-13: neuroprotective models against ischemia-induced neurotoxicity (NIN models). | | | | | | | | | | | |
| 14-26: neuroprotective models against H2O2-induced neurotoxicity (NHN models). | | | | | | | | | | | |

**Table** **S7** Detailed prediction results of the 398 compounds predicted active by two phenotypic screening models

| molecule | source | NB-1-LPFP6 | | NB-2-LPFP6 | |
| --- | --- | --- | --- | --- | --- |
|  |  | EstPGood | Prediction | EstPGood | Prediction |
| 1-Palmitoylglycerol | Chuan xiong | 0.423949 | TRUE | 0.165346 | TRUE |
| 9,12-octadecadienoic acid ethylester | Chuan xiong | 0.318933 | TRUE | 0.140082 | TRUE |
| 9,12-octadecadienoic acid methylester | Chuan xiong | 0.275752 | TRUE | 0.102272 | TRUE |
| beta-asarone | Chuan xiong | 0.286439 | TRUE | 0.138253 | TRUE |
| Coniferylfcrulate | Chuan xiong | 0.837697 | TRUE | 0.182951 | TRUE |
| Ethyl margarate [14010-23-2] | Chuan xiong | 0.17055 | TRUE | 0.170444 | TRUE |
| Ethylisoheptadecanoate | Chuan xiong | 0.155447 | TRUE | 0.168051 | TRUE |
| Ethylisooctadecanoate | Chuan xiong | 0.161412 | TRUE | 0.165202 | TRUE |
| Ethyloctadecanoate,Ethyl stearate | Chuan xiong | 0.176434 | TRUE | 0.176191 | TRUE |
| Ethylpentadecanoate | Chuan xiong | 0.155708 | TRUE | 0.171069 | TRUE |
| Exceparl M-OL | Chuan xiong | 0.220705 | TRUE | 0.108796 | TRUE |
| Mandenol | Chuan xiong | 0.318933 | TRUE | 0.140082 | TRUE |
| Methyl linoleate | Chuan xiong | 0.21779 | TRUE | 0.105482 | TRUE |
| Myricanone | Chuan xiong | 0.32608 | TRUE | 0.189831 | TRUE |
| palmitic acid ethylester | Chuan xiong | 0.165393 | TRUE | 0.168503 | TRUE |
| PLO | Chuan xiong | 0.421352 | TRUE | 0.978439 | TRUE |
| sinapic acid | Chuan xiong | 0.229496 | TRUE | 0.169523 | TRUE |
| Sitogluside | Chuan xiong | 0.97605 | TRUE | 0.441895 | TRUE |
| sitosterol | Chuan xiong | 0.88894 | TRUE | 0.886617 | TRUE |
| Vitamin A | Chuan xiong | 0.425533 | TRUE | 0.120933 | TRUE |
| (2R,3R)-3-(4-hydroxy-3-methoxy-phenyl)-5-methoxy-2-methylol-2,3-dihydropyrano[5,6-h][1,4]benzodioxin-9-one | Fang feng | 0.809237 | TRUE | 0.165498 | TRUE |
| (2S)-4-methoxy-7-methyl-2-[1-methyl-1-[(2S,3R,4S,5S,6R)-3,4,5-trihydroxy-6-methylol-tetrahydropyran-2-yl]oxy-ethyl]-2,3-dihydrofuro[3,2-g]chromen-5-one | Fang feng | 0.993204 | TRUE | 0.133496 | TRUE |
| 11,14-Eicosadienoic acid, methyl ester | Fang feng | 0.29417 | TRUE | 0.104123 | TRUE |
| 11-hydroxy-sec-o-beta-d-glucosylhamaudol | Fang feng | 0.949716 | TRUE | 0.132686 | TRUE |
| 11-hydroxy-sec-o-beta-d-glucosylhamaudol_qt | Fang feng | 0.894679 | TRUE | 0.145411 | TRUE |
| 3-O-Acetylhamaudol | Fang feng | 0.873223 | TRUE | 0.23528 | TRUE |
| 3-O-Angeloylhamaudol | Fang feng | 0.825158 | TRUE | 0.432519 | TRUE |
| 4''-O-β-Glucopyranosyl-5-O-methylvisamminol | Fang feng | 0.993204 | TRUE | 0.133496 | TRUE |
| 5-O-Methylvisamminol | Fang feng | 0.851338 | TRUE | 0.199439 | TRUE |
| 5-O-Methylvisammioside | Fang feng | 0.99352 | TRUE | 0.133496 | TRUE |
| 9-Octadecenoic acid, ethyl ester | Fang feng | 0.248456 | TRUE | 0.152655 | TRUE |
| anomalin | Fang feng | 0.226671 | TRUE | 0.225525 | TRUE |
| Cimifugin | Fang feng | 0.881195 | TRUE | 0.183048 | TRUE |
| Daucosterol | Fang feng | 0.97605 | TRUE | 0.441895 | TRUE |
| divaricatacid | Fang feng | 0.849822 | TRUE | 0.178037 | TRUE |
| divaricataester,b | Fang feng | 0.425418 | TRUE | 0.242513 | TRUE |
| divaricatol | Fang feng | 0.914989 | TRUE | 0.217386 | TRUE |
| fraxidin | Fang feng | 0.222085 | TRUE | 0.169283 | TRUE |
| Hamaudol | Fang feng | 0.887326 | TRUE | 0.158828 | TRUE |
| isofraxidin | Fang feng | 0.183808 | TRUE | 0.17197 | TRUE |
| Ledebouliellol | Fang feng | 0.90113 | TRUE | 0.404303 | TRUE |
| Ledebouriellol | Fang feng | 0.872421 | TRUE | 0.404303 | TRUE |
| Myristicin | Fang feng | 0.337785 | TRUE | 0.492529 | TRUE |
| prim-o-beta-d-glucosylcimifugin | Fang feng | 0.921134 | TRUE | 0.172741 | TRUE |
| Prim-O-glucosylcimifugin | Fang feng | 0.996003 | TRUE | 0.101146 | TRUE |
| scopoletin | Fang feng | 0.218894 | TRUE | 0.10087 | TRUE |
| sec-o-beta-d-glucosylhamaudol | Fang feng | 0.943881 | TRUE | 0.140985 | TRUE |
| Undulatoside A | Fang feng | 0.999255 | TRUE | 0.289606 | TRUE |
| (＋)-2-N-Methyltetrandrine | Fang ji | 0.758181 | TRUE | 0.813927 | TRUE |
| (+)-Limacine | Fang ji | 0.882246 | TRUE | 0.727953 | TRUE |
| 1, 3, 4-Tridehydrofangchinolium hydroxide | Fang ji | 0.910303 | TRUE | 0.83052 | TRUE |
| 2’, 2’－N,N-Dichloromethyltetrandrine | Fang ji | 0.64938 | TRUE | 0.535131 | TRUE |
| 7-Hydroxy-aristolochic acid A | Fang ji | 0.924697 | TRUE | 0.438956 | TRUE |
| Aristolochic acid | Fang ji | 0.925122 | TRUE | 0.345127 | TRUE |
| Aristololactum | Fang ji | 0.936845 | TRUE | 0.138782 | TRUE |
| Berbamine | Fang ji | 0.878705 | TRUE | 0.856478 | TRUE |
| Betaine | Fang ji | 0.925122 | TRUE | 0.345127 | TRUE |
| beta-sitosterol | Fang ji | 0.88894 | TRUE | 0.886617 | TRUE |
| Cissamine | Fang ji | 0.259138 | TRUE | 0.178076 | TRUE |
| Cissampareine | Fang ji | 0.645009 | TRUE | 0.837354 | TRUE |
| Coccutrine | Fang ji | 0.362902 | TRUE | 0.168685 | TRUE |
| Coclobine | Fang ji | 0.853205 | TRUE | 0.784192 | TRUE |
| Cocsulinine | Fang ji | 0.761033 | TRUE | 0.634239 | TRUE |
| Curine | Fang ji | 0.879416 | TRUE | 0.690602 | TRUE |
| Cyclanoline | Fang ji | 0.266539 | TRUE | 0.178076 | TRUE |
| Cycleaneonine | Fang ji | 0.567693 | TRUE | 0.806242 | TRUE |
| Dichotomitin | Fang ji | 0.968046 | TRUE | 0.851705 | TRUE |
| Epistephanine | Fang ji | 0.83535 | TRUE | 0.873981 | TRUE |
| Fangchinoline | Fang ji | 0.827676 | TRUE | 0.727455 | TRUE |
| Fenfangjine A | Fang ji | 0.811279 | TRUE | 0.77399 | TRUE |
| Fenfangjine D | Fang ji | 0.706 | TRUE | 0.544155 | TRUE |
| Hesperetin | Fang ji | 0.858894 | TRUE | 0.293062 | TRUE |
| Isotrilobine | Fang ji | 0.800787 | TRUE | 0.655446 | TRUE |
| Isotrilobine-N-2-oxide | Fang ji | 0.81997 | TRUE | 0.67035 | TRUE |
| Jatrorrhizine | Fang ji | 0.498585 | TRUE | 0.190656 | TRUE |
| Magnoflorine | Fang ji | 0.206585 | TRUE | 0.599692 | TRUE |
| Menisarine | Fang ji | 0.858367 | TRUE | 0.910912 | TRUE |
| Menisine | Fang ji | 0.819445 | TRUE | 0.787697 | TRUE |
| menisperine | Fang ji | 0.380035 | TRUE | 0.895325 | TRUE |
| Normenisarine | Fang ji | 0.790194 | TRUE | 0.910038 | TRUE |
| Oxofangchirine | Fang ji | 0.63688 | TRUE | 0.675461 | TRUE |
| Stephanthrine | Fang ji | 0.935987 | TRUE | 0.235569 | TRUE |
| STOCK1N-53032 | Fang ji | 0.152955 | TRUE | 0.514891 | TRUE |
| Tetrandrine | Fang ji | 0.754324 | TRUE | 0.787275 | TRUE |
| Tiliacorine | Fang ji | 0.644059 | TRUE | 0.595115 | TRUE |
| TNP00326 | Fang ji | 0.819445 | TRUE | 0.787697 | TRUE |
| Trilobamine | Fang ji | 0.84857 | TRUE | 0.718363 | TRUE |
| Trilobine | Fang ji | 0.891373 | TRUE | 0.560483 | TRUE |
| fuziline | Fu zi | 0.355335 | TRUE | 0.859289 | TRUE |
| fuzitine | Fu zi | 0.355335 | TRUE | 0.859289 | TRUE |
| Neokadsuranic acid B | Fu zi | 0.208282 | TRUE | 0.17565 | TRUE |
| penduline | Fu zi | 0.768996 | TRUE | 0.857304 | TRUE |
| Tamarixinol | Fu zi | 0.150213 | TRUE | 0.108703 | TRUE |
| TMPEA | Fu zi | 0.3398 | TRUE | 0.115483 | TRUE |
| (-)-Medicocarpin | Gan cao | 0.919386 | TRUE | 0.151387 | TRUE |
| (2R)-2-[3,4-dihydroxy-5-(3-methylbut-2-enyl)phenyl]-5,7-dihydroxy-8-(3-methylbut-2-enyl)chroman-4-one | Gan cao | 0.425922 | TRUE | 0.164273 | TRUE |
| 1,3-dihydroxy-8,9-dimethoxy-6-benzofurano[3,2-c]chromenone | Gan cao | 0.63828 | TRUE | 0.160144 | TRUE |
| 1-Methoxyficifolinol | Gan cao | 0.403987 | TRUE | 0.225425 | TRUE |
| 1-Methoxyphaseollidin | Gan cao | 0.607143 | TRUE | 0.309502 | TRUE |
| 2-(3,4-dihydroxyphenyl)-5,7-dihydroxy-6-(3-methylbut-2-enyl)chromone | Gan cao | 0.999978 | TRUE | 0.126894 | TRUE |
| 2',7-Dihydroxy-4'-methoxyisoflavan-7-O-β-d-glucopyranoside | Gan cao | 0.78297 | TRUE | 0.138091 | TRUE |
| 3-(3,4-dihydroxyphenyl)-5,7-dihydroxy-8-(3-methylbut-2-enyl)chromone | Gan cao | 0.655709 | TRUE | 0.129361 | TRUE |
| 3'(γ,γ-dimethylallyl)-kievitone | Gan cao | 0.365513 | TRUE | 0.281988 | TRUE |
| 3,3’－Dimethylquercetin | Gan cao | 1 | TRUE | 0.293579 | TRUE |
| 3-[4,6-dihydroxy-2-methoxy-3-(3-methylbut-2-enyl)phenyl]-7-hydroxy-chromone | Gan cao | 0.449843 | TRUE | 0.170487 | TRUE |
| 3'-Hydroxy-4'-O-Methylglabridin | Gan cao | 0.477683 | TRUE | 0.117533 | TRUE |
| 3-Hydroxyglabrol | Gan cao | 0.530973 | TRUE | 0.32821 | TRUE |
| 3-Hydroxyglabrol Ⅰ | Gan cao | 0.669935 | TRUE | 0.295032 | TRUE |
| 3-Hydroxyglabrol Ⅱ | Gan cao | 0.530973 | TRUE | 0.324281 | TRUE |
| 4H-1-Benzopyran-4-one, 2-(4-(beta-D-glucopyranosyloxy)phenyl)-2,3-dihydro-5,7-dihydroxy-, (2S)- | Gan cao | 0.979227 | TRUE | 0.201906 | TRUE |
| 5,7,3'',4''-4-tetrahydroxy-3-methoxy-5''-isoprenyl flavone | Gan cao | 0.999892 | TRUE | 0.265257 | TRUE |
| 5,7-dihydroxy-3-(2-hydroxy-4-methoxy-phenyl)-6-(3-methylbut-2-enyl)chromone | Gan cao | 0.439523 | TRUE | 0.192407 | TRUE |
| 5,7-dihydroxy-3-(4-methoxyphenyl)-8-(3-methylbut-2-enyl)chromone | Gan cao | 0.332376 | TRUE | 0.143383 | TRUE |
| 5-O-Methyl licoricidin | Gan cao | 0.274176 | TRUE | 0.248131 | TRUE |
| 6,5'-dipernylluteolin | Gan cao | 0.997915 | TRUE | 0.135091 | TRUE |
| 6-prenylated eriodictyol | Gan cao | 0.784103 | TRUE | 0.204105 | TRUE |
| 7-Acetoxy-2-methylisoflavone | Gan cao | 0.262387 | TRUE | 0.156288 | TRUE |
| 7-hydroxy-2-methyl-3-phenyl-chromone | Gan cao | 0.26427 | TRUE | 0.126953 | TRUE |
| 7-Methoxy-2-methyl isoflavone | Gan cao | 0.29518 | TRUE | 0.166946 | TRUE |
| 7-Methoxy-2-methylisoflavone | Gan cao | 0.29518 | TRUE | 0.166946 | TRUE |
| 7-Methoxy-4''-hydroxyflavonol | Gan cao | 0.999962 | TRUE | 0.112118 | TRUE |
| 8-prenylated eriodictyol | Gan cao | 0.743768 | TRUE | 0.155055 | TRUE |
| Artonin E | Gan cao | 0.890203 | TRUE | 0.20626 | TRUE |
| astragalin | Gan cao | 1 | TRUE | 0.201149 | TRUE |
| Castanin | Gan cao | 0.451371 | TRUE | 0.143922 | TRUE |
| dehydroglyasperins C | Gan cao | 0.212463 | TRUE | 0.100941 | TRUE |
| Docosyl caffeate | Gan cao | 0.518117 | TRUE | 0.180605 | TRUE |
| Gancaonin A | Gan cao | 0.363795 | TRUE | 0.158715 | TRUE |
| Gancaonin B | Gan cao | 0.782946 | TRUE | 0.193543 | TRUE |
| Gancaonin C | Gan cao | 0.342253 | TRUE | 0.110796 | TRUE |
| Gancaonin D | Gan cao | 0.775265 | TRUE | 0.169419 | TRUE |
| Gancaonin E | Gan cao | 0.425922 | TRUE | 0.164273 | TRUE |
| Gancaonin G | Gan cao | 0.436215 | TRUE | 0.150421 | TRUE |
| Gancaonin P | Gan cao | 1 | TRUE | 0.272742 | TRUE |
| Gancaonin P-3′-methylether | Gan cao | 1 | TRUE | 0.29679 | TRUE |
| Gancaonin U | Gan cao | 0.309061 | TRUE | 0.383388 | TRUE |
| Gancaonin V | Gan cao | 0.390557 | TRUE | 0.340345 | TRUE |
| Genkwanin | Gan cao | 0.998924 | TRUE | 0.141444 | TRUE |
| Glabranin | Gan cao | 0.50335 | TRUE | 0.153905 | TRUE |
| Glabranine | Gan cao | 0.50335 | TRUE | 0.153905 | TRUE |
| Glepidotin A | Gan cao | 0.99846 | TRUE | 0.263691 | TRUE |
| Glepidotin B | Gan cao | 0.517451 | TRUE | 0.844624 | TRUE |
| Glisoflavanone | Gan cao | 0.405824 | TRUE | 0.319835 | TRUE |
| Glyasperin A | Gan cao | 0.999766 | TRUE | 0.116231 | TRUE |
| glyasperin B | Gan cao | 0.489599 | TRUE | 0.398738 | TRUE |
| Glyasperin C | Gan cao | 0.278036 | TRUE | 0.22097 | TRUE |
| glyasperin E | Gan cao | 0.27908 | TRUE | 0.139359 | TRUE |
| glyasperin F | Gan cao | 0.664921 | TRUE | 0.186331 | TRUE |
| glyasperins D | Gan cao | 0.297963 | TRUE | 0.251085 | TRUE |
| Glyasperins K | Gan cao | 0.489575 | TRUE | 0.198976 | TRUE |
| Glyasperins M | Gan cao | 0.744147 | TRUE | 0.252196 | TRUE |
| glyasperins Z | Gan cao | 0.302547 | TRUE | 0.118758 | TRUE |
| Glycyrrhiso flavanone | Gan cao | 0.772687 | TRUE | 0.268789 | TRUE |
| Glycyrrhisoflavone | Gan cao | 0.443662 | TRUE | 0.165346 | TRUE |
| Glyzaglabrin | Gan cao | 0.708566 | TRUE | 0.302033 | TRUE |
| Hirsutrin | Gan cao | 1 | TRUE | 0.195365 | TRUE |
| Hispaglabridin A | Gan cao | 0.619722 | TRUE | 0.117793 | TRUE |
| Inermine | Gan cao | 0.644108 | TRUE | 0.293958 | TRUE |
| Isolicoflavonol | Gan cao | 0.999893 | TRUE | 0.127943 | TRUE |
| isoquercitrin | Gan cao | 1 | TRUE | 0.195365 | TRUE |
| isorhamnetin | Gan cao | 1 | TRUE | 0.306401 | TRUE |
| Isoschaftoside | Gan cao | 0.997939 | TRUE | 0.132539 | TRUE |
| Isotrffoliol | Gan cao | 0.426804 | TRUE | 0.102815 | TRUE |
| Isotrifoliol | Gan cao | 0.426804 | TRUE | 0.102815 | TRUE |
| Isoviolanthin | Gan cao | 0.998504 | TRUE | 0.142842 | TRUE |
| Jaranol | Gan cao | 0.999851 | TRUE | 0.283173 | TRUE |
| Kaempferol | Gan cao | 0.999883 | TRUE | 0.239225 | TRUE |
| Kaempferol-3-O-glucoside | Gan cao | 1 | TRUE | 0.201149 | TRUE |
| Kanzonol F | Gan cao | 0.52976 | TRUE | 0.123693 | TRUE |
| Kanzonol H | Gan cao | 0.646351 | TRUE | 0.172636 | TRUE |
| Kanzonol Z | Gan cao | 0.672336 | TRUE | 0.156626 | TRUE |
| kanzonols K | Gan cao | 0.457985 | TRUE | 0.167765 | TRUE |
| kanzonols L | Gan cao | 0.21286 | TRUE | 0.121161 | TRUE |
| kanzonols T | Gan cao | 0.385873 | TRUE | 0.113969 | TRUE |
| kanzonols X | Gan cao | 0.218207 | TRUE | 0.120551 | TRUE |
| Licoagrocarpin | Gan cao | 0.388273 | TRUE | 0.172778 | TRUE |
| Licoagroside D | Gan cao | 0.963419 | TRUE | 0.240317 | TRUE |
| Licoagroside E | Gan cao | 0.977478 | TRUE | 0.115318 | TRUE |
| Licochaleone B | Gan cao | 0.515992 | TRUE | 0.110168 | TRUE |
| Licocoumarone | Gan cao | 0.406739 | TRUE | 0.154507 | TRUE |
| Licoflavonol | Gan cao | 0.999855 | TRUE | 0.267385 | TRUE |
| licoisoflavanone | Gan cao | 0.638684 | TRUE | 0.166448 | TRUE |
| Licoisoflavone | Gan cao | 0.472679 | TRUE | 0.166393 | TRUE |
| Licoricidin | Gan cao | 0.308727 | TRUE | 0.221159 | TRUE |
| Licoricone | Gan cao | 0.493148 | TRUE | 0.201023 | TRUE |
| Licoriisoflavan A | Gan cao | 0.308216 | TRUE | 0.248131 | TRUE |
| lsolicoflavonol | Gan cao | 0.999893 | TRUE | 0.127943 | TRUE |
| lsoquercitrin | Gan cao | 1 | TRUE | 0.195365 | TRUE |
| Lupiwighteone | Gan cao | 0.342253 | TRUE | 0.110796 | TRUE |
| Medicarpin | Gan cao | 0.27263 | TRUE | 0.148064 | TRUE |
| Medicarpin-3-O-glucoside | Gan cao | 0.919386 | TRUE | 0.151387 | TRUE |
| Morusin | Gan cao | 0.848035 | TRUE | 0.125422 | TRUE |
| naringenin | Gan cao | 0.542831 | TRUE | 0.162267 | TRUE |
| Neouralenol | Gan cao | 0.999985 | TRUE | 0.308725 | TRUE |
| Nortangeretin | Gan cao | 0.987727 | TRUE | 0.104824 | TRUE |
| Odoratin | Gan cao | 0.784403 | TRUE | 0.144526 | TRUE |
| Pinocembrin | Gan cao | 0.498947 | TRUE | 0.20215 | TRUE |
| Prunetin | Gan cao | 0.4455 | TRUE | 0.135701 | TRUE |
| quercetin | Gan cao | 1 | TRUE | 0.250269 | TRUE |
| Quercetin der. | Gan cao | 1 | TRUE | 0.293579 | TRUE |
| schaftoside | Gan cao | 0.996858 | TRUE | 0.138165 | TRUE |
| Scopoletol | Gan cao | 0.218894 | TRUE | 0.10087 | TRUE |
| Semilicoisoflavone B | Gan cao | 0.628643 | TRUE | 0.108083 | TRUE |
| sigmoidin B | Gan cao | 0.542921 | TRUE | 0.219224 | TRUE |
| Sigmoidin-B | Gan cao | 0.566929 | TRUE | 0.219224 | TRUE |
| TAXIFOLIN | Gan cao | 0.816776 | TRUE | 0.964602 | TRUE |
| trans-Sinapic acid methylester | Gan cao | 0.279853 | TRUE | 0.273905 | TRUE |
| Uralene | Gan cao | 0.999903 | TRUE | 0.310612 | TRUE |
| Uralenin | Gan cao | 0.998768 | TRUE | 0.153482 | TRUE |
| Uralenol | Gan cao | 0.999933 | TRUE | 0.286916 | TRUE |
| Uralenol-3-methylether | Gan cao | 0.999892 | TRUE | 0.265257 | TRUE |
| Vestitol | Gan cao | 0.213488 | TRUE | 0.113521 | TRUE |
| vicenin-2 | Gan cao | 0.997567 | TRUE | 0.160936 | TRUE |
| violanthin | Gan cao | 0.998504 | TRUE | 0.146109 | TRUE |
| vitexin | Gan cao | 0.999571 | TRUE | 0.135484 | TRUE |
| (-)-Epicatechin | Gui zhi | 0.679789 | TRUE | 0.43339 | TRUE |
| (-)-taxifolin | Gui zhi | 0.832164 | TRUE | 0.964602 | TRUE |
| (+)-catechin | Gui zhi | 0.699418 | TRUE | 0.429462 | TRUE |
| 5-epicatechin | Gui zhi | 0.663147 | TRUE | 0.705004 | TRUE |
| copaene | Gui zhi | 0.163011 | TRUE | 0.262619 | TRUE |
| ent-Epicatechin | Gui zhi | 0.699418 | TRUE | 0.429462 | TRUE |
| Procyanidin B2 | Gui zhi | 0.706185 | TRUE | 0.677054 | TRUE |
| SYRINGIC ACID | Gui zhi | 0.216186 | TRUE | 0.120147 | TRUE |
| (+)-Syringaresinol | Huang qin | 0.226396 | TRUE | 0.625743 | TRUE |
| (2R)-7-hydroxy-5-methoxy-2-phenylchroman-4-one | Huang qin | 0.641995 | TRUE | 0.312892 | TRUE |
| (2R,3R)-2'',3,5,7-tetrahydroxyflavanone | Huang qin | 0.607248 | TRUE | 0.880358 | TRUE |
| (2S)-2'',5,6''-Trihydroxy-7-methoxyflavanone | Huang qin | 0.455011 | TRUE | 0.280235 | TRUE |
| (2S)-2'',5,6''-trihydroxy-7-methoxyflavanone-2''-O-β-D-glucopyranoside | Huang qin | 0.97101 | TRUE | 0.349082 | TRUE |
| (2S)-5,7,2',5'-Tetrahydroxyflavanone | Huang qin | 0.429454 | TRUE | 0.119194 | TRUE |
| (2S)-5,7,2',6'-tetrahydroxyflavanone | Huang qin | 0.386723 | TRUE | 0.208231 | TRUE |
| (2S)-5,7-Dihydroxy-6-methoxyflavanone-7-O-β-D-glucopyranoside | Huang qin | 0.985265 | TRUE | 0.51284 | TRUE |
| (2S)-7,2',6'-trihydroxy-5-methoxyflavanone | Huang qin | 0.501775 | TRUE | 0.327909 | TRUE |
| 2-(2,6-dihydroxyphenyl)-3,5,7-trihydroxy-chromone | Huang qin | 0.994931 | TRUE | 0.347166 | TRUE |
| 2,6,2'',4''-Tetrahydroxy-6''-methoxyehalcone | Huang qin | 0.37686 | TRUE | 0.245669 | TRUE |
| 3,5,7,2'',6''-Pentahydroxy flavonol | Huang qin | 0.994931 | TRUE | 0.347166 | TRUE |
| 3,5,7,2',6'-pentahydroxyflavanone | Huang qin | 0.42064 | TRUE | 0.873525 | TRUE |
| 3,5,7,2',6'-Pentahydroxyflavone-2'-O-β-D-glucopyranoside | Huang qin | 0.999938 | TRUE | 0.26384 | TRUE |
| 3,5,7,2''6''-Pentahydroxy flavanonol | Huang qin | 0.42064 | TRUE | 0.873525 | TRUE |
| 5, 2'', 5''-Trihydroxy-6, 7, 8-trimethoxyflavone | Huang qin | 0.998998 | TRUE | 0.330034 | TRUE |
| 5, 2'', 6''-Trihydroxy-7, 8-dimethoxyflavone | Huang qin | 0.984593 | TRUE | 0.679383 | TRUE |
| 5, 7, 2''-Trihydroxy-6-methoxyflavone | Huang qin | 0.999445 | TRUE | 0.381894 | TRUE |
| 5, 7, 2''-Trihydroxy-8, 6''-dimethoxyflavone | Huang qin | 0.9866 | TRUE | 0.574061 | TRUE |
| 5, 7, 2''-Trihydroxyflavone | Huang qin | 0.997332 | TRUE | 0.133788 | TRUE |
| 5, 7, 4''-Trihydroxy-6-C-glucoside-8-C-arabinoside flavone | Huang qin | 0.68437 | TRUE | 0.501633 | TRUE |
| 5, 7, 4''-Trihydroxy-8-methoxyflavone | Huang qin | 0.99832 | TRUE | 0.294308 | TRUE |
| 5, 8, 2''-Trihydroxy-6, 7-dimethoxyflavone | Huang qin | 0.99442 | TRUE | 0.258388 | TRUE |
| 5, 8, 2''-Trihydroxy-7-methoxyflavone | Huang qin | 0.999236 | TRUE | 0.480576 | TRUE |
| 5,2',6'-Trihydroxy-6,7,8-trimethoxyflavone-2'-O-β-D-glucopyranoside | Huang qin | 0.997661 | TRUE | 0.253276 | TRUE |
| 5,2',6'-Trihydroxy-6,7-dimethoxyflavone-2'-O-β-D-glucopyranoside | Huang qin | 0.999921 | TRUE | 0.548851 | TRUE |
| 5,2',6'-trihydroxy-7,8-dimethoxyflavone-2'-O-β-D-glucopyranoside | Huang qin | 0.999562 | TRUE | 0.513071 | TRUE |
| 5,2’－ Dihydroxy－6,7,8－trimethoxyflavone | Huang qin | 0.994415 | TRUE | 0.264974 | TRUE |
| 5,2''-Dihydroxy-6,7,8,6''-tetamethoxyflavone | Huang qin | 0.973091 | TRUE | 0.465454 | TRUE |
| 5,6－Dihydroxy－7－O-glucoside－flavone | Huang qin | 1 | TRUE | 0.506809 | TRUE |
| 5,7,2',3'-Tetrahydroxyflavone | Huang qin | 0.998345 | TRUE | 0.11404 | TRUE |
| 5,7,2'',5''-Tetrahydroxy-8,6''-dimethoxy flavone | Huang qin | 0.979875 | TRUE | 0.68099 | TRUE |
| 5,7,2',6'-Tetrahydroxyflavone | Huang qin | 0.968049 | TRUE | 0.202267 | TRUE |
| 5,7,2'''',6''''-Tetrahydroxyflavone | Huang qin | 0.968049 | TRUE | 0.202267 | TRUE |
| 5,7,2'-Trihydroxy-6,8-dimethoxyflavone | Huang qin | 0.9915 | TRUE | 0.17858 | TRUE |
| 5,7,2'-Trihydroxy-6'-methoxyflavone | Huang qin | 0.981667 | TRUE | 0.27874 | TRUE |
| 5,7,2'-Trihydroxy-6-methoxyflavone-7-O-β-D-glucuronide | Huang qin | 0.999978 | TRUE | 0.397822 | TRUE |
| 5,7,2'-Trihydroxy-8,6'-dimethoxyflavone | Huang qin | 0.9866 | TRUE | 0.574061 | TRUE |
| 5,7,2''-Trihydroxy-8-methoxyflavone | Huang qin | 0.997443 | TRUE | 0.333878 | TRUE |
| 5,7,2'-Trihydroxy-8-methoxyflavone | Huang qin | 0.997443 | TRUE | 0.333878 | TRUE |
| 5,7,2'-Trihydroxyflavone | Huang qin | 0.997332 | TRUE | 0.133788 | TRUE |
| 5,7,4'-trihydroxy-6-methoxyflavanone | Huang qin | 0.68437 | TRUE | 0.501633 | TRUE |
| 5,7,4'-Trihydroxy-8-methoxyflavone | Huang qin | 0.99832 | TRUE | 0.294308 | TRUE |
| 5,7－Dihydroxy－6,8,2’，3’－tetramethoxyflavone | Huang qin | 0.995956 | TRUE | 0.176789 | TRUE |
| 5,8,2'-Trihydroxy-6,7-dimethoxyflavone | Huang qin | 0.99442 | TRUE | 0.258388 | TRUE |
| 5,8,2'-Trihydroxy-7-methoxyflavone | Huang qin | 0.999236 | TRUE | 0.480576 | TRUE |
| 5，8－Dihydroxy－6,7－dimethoxyflavone | Huang qin | 0.98877 | TRUE | 0.3478 | TRUE |
| 5,8-dihydroxy-6,7-dimethoxyflavone | Huang qin | 0.98877 | TRUE | 0.3478 | TRUE |
| 5-hydroxy-7,8-dimethoxyflavone | Huang qin | 0.996976 | TRUE | 0.647894 | TRUE |
| 6,2'-Dihydroxy-5,7,8,6'-tetramethoxyflavone | Huang qin | 0.966526 | TRUE | 0.538493 | TRUE |
| 7, 2'', 6''-Trihydroxy-5-methoxychalcone | Huang qin | 0.37686 | TRUE | 0.245669 | TRUE |
| 7, 2''6''-Trihydroxy-5-methoxyflavanone | Huang qin | 0.501775 | TRUE | 0.327909 | TRUE |
| 7-Methoxybaicalein | Huang qin | 0.999708 | TRUE | 0.590535 | TRUE |
| 8-Methoxy-5-O-glucoside flavone | Huang qin | 0.999624 | TRUE | 0.437657 | TRUE |
| acacetin | Huang qin | 0.998782 | TRUE | 0.149053 | TRUE |
| apigenin | Huang qin | 0.998289 | TRUE | 0.123128 | TRUE |
| Baicalein | Huang qin | 0.999038 | TRUE | 0.348928 | TRUE |
| baicalein-7-o-β-D-glucopyranoside | Huang qin | 1 | TRUE | 0.506809 | TRUE |
| Baicalin | Huang qin | 0.999038 | TRUE | 0.348928 | TRUE |
| Campesterol | Huang qin | 0.900127 | TRUE | 0.899105 | TRUE |
| Carthamidin | Huang qin | 0.708406 | TRUE | 0.389219 | TRUE |
| Chrysin | Huang qin | 0.994452 | TRUE | 0.183073 | TRUE |
| Chrysin 6-C-beta-D-glucopyranoside | Huang qin | 0.99954 | TRUE | 0.327112 | TRUE |
| Chrysin 8-C-beta-D-glucopyranoside | Huang qin | 0.998739 | TRUE | 0.198109 | TRUE |
| Chrysin-6-C-α-L-arabinopyranosyl-8-C-β-D-glucopyranoside | Huang qin | 0.999991 | TRUE | 0.29593 | TRUE |
| chrysin-7-O-β-D-glucuronide | Huang qin | 1 | TRUE | 0.230497 | TRUE |
| Cosmetin | Huang qin | 0.999998 | TRUE | 0.140287 | TRUE |
| Dihydrobaicalin | Huang qin | 0.999794 | TRUE | 0.504601 | TRUE |
| Dihydrobaicalin_qt | Huang qin | 0.731901 | TRUE | 0.434152 | TRUE |
| DIHYDROOROXYLIN | Huang qin | 0.695758 | TRUE | 0.55245 | TRUE |
| dihydrooroxylin A | Huang qin | 0.566752 | TRUE | 0.235989 | TRUE |
| Dihydrooxylin A | Huang qin | 0.695758 | TRUE | 0.55245 | TRUE |
| Eriodictyol | Huang qin | 0.793187 | TRUE | 0.227224 | TRUE |
| Eriodyctiol (flavanone) | Huang qin | 0.793187 | TRUE | 0.227224 | TRUE |
| Ganhuangenin | Huang qin | 0.979875 | TRUE | 0.68099 | TRUE |
| Isoscutellarein | Huang qin | 0.998154 | TRUE | 0.228872 | TRUE |
| NEOBAICALEIN | Huang qin | 0.995995 | TRUE | 0.168671 | TRUE |
| Norwogonin | Huang qin | 0.994847 | TRUE | 0.328953 | TRUE |
| Oroxindin | Huang qin | 0.999999 | TRUE | 0.442716 | TRUE |
| Oroxylin | Huang qin | 0.993365 | TRUE | 0.17663 | TRUE |
| oroxylin-A | Huang qin | 0.998691 | TRUE | 0.4552 | TRUE |
| oroxylin-A-7-O-β-D-glucuronide | Huang qin | 1 | TRUE | 0.483444 | TRUE |
| Panicolin | Huang qin | 0.998743 | TRUE | 0.531266 | TRUE |
| Salvigenin | Huang qin | 0.999891 | TRUE | 0.573194 | TRUE |
| scutellarein | Huang qin | 0.999667 | TRUE | 0.257297 | TRUE |
| Scutellarin | Huang qin | 1 | TRUE | 0.288452 | TRUE |
| skullcapflavone I | Huang qin | 0.998743 | TRUE | 0.531266 | TRUE |
| skullcapflavone II | Huang qin | 0.973091 | TRUE | 0.465454 | TRUE |
| SkullcapflavoneⅠ | Huang qin | 0.998743 | TRUE | 0.54242 | TRUE |
| Tenaxin I | Huang qin | 0.994415 | TRUE | 0.264974 | TRUE |
| Viscidulin I | Huang qin | 0.994931 | TRUE | 0.347166 | TRUE |
| Viscidulin II | Huang qin | 0.984593 | TRUE | 0.679383 | TRUE |
| Viscidulin III | Huang qin | 0.999993 | TRUE | 0.663465 | TRUE |
| Wogonin | Huang qin | 0.993906 | TRUE | 0.416151 | TRUE |
| Wogonoside | Huang qin | 0.999999 | TRUE | 0.441317 | TRUE |
| 24-Methylcholest-5-en-3β-ol | Ma huang | 0.900127 | TRUE | 0.899105 | TRUE |
| 3-0-β-D-Glucopyranosyl-5,9,4''-trihydroxy-8-methoxyflavone | Ma huang | 0.999999 | TRUE | 0.226363 | TRUE |
| 3-Methoxyherbacetin | Ma huang | 0.999734 | TRUE | 0.446282 | TRUE |
| apigenin 7-O-glucoside | Ma huang | 0.999998 | TRUE | 0.140287 | TRUE |
| chlorogenic acid | Ma huang | 0.500288 | TRUE | 0.132781 | TRUE |
| Herbacetin | Ma huang | 0.999775 | TRUE | 0.432225 | TRUE |
| herbacetin 7-ghcoside | Ma huang | 1 | TRUE | 0.443564 | TRUE |
| herbacetin 7-methyl ether | Ma huang | 0.999949 | TRUE | 0.724141 | TRUE |
| herbacetin 8-methyl ether 3-glucoside | Ma huang | 1 | TRUE | 0.42373 | TRUE |
| Kaempferol 3-rhamnoside | Ma huang | 1 | TRUE | 0.15175 | TRUE |
| Kaempferol 5-rhamnoside | Ma huang | 1 | TRUE | 0.281003 | TRUE |
| Kaempferol 7-rhamnoside | Ma huang | 1 | TRUE | 0.192346 | TRUE |
| Kaempferol rhamnoside | Ma huang | 1 | TRUE | 0.15175 | TRUE |
| Leucodelphinidin | Ma huang | 0.462199 | TRUE | 0.471393 | TRUE |
| luceninⅢ | Ma huang | 0.999944 | TRUE | 0.118898 | TRUE |
| Mahuangnin A | Ma huang | 0.506443 | TRUE | 0.396144 | TRUE |
| Mahuangnin B | Ma huang | 0.594966 | TRUE | 0.256523 | TRUE |
| Mahuangnin C | Ma huang | 0.512372 | TRUE | 0.435228 | TRUE |
| Mahuangnin D | Ma huang | 0.507706 | TRUE | 0.364342 | TRUE |
| quercetin 3-0-galactoside | Ma huang | 1 | TRUE | 0.195365 | TRUE |
| quercetine 3-rhamnoside | Ma huang | 1 | TRUE | 0.14749 | TRUE |
| tricin | Ma huang | 0.999805 | TRUE | 0.339544 | TRUE |
| vicenin | Ma huang | 0.998041 | TRUE | 0.132539 | TRUE |
| 2,5－Dimethyl－7－hydroxy chromone | Ren shen | 0.860401 | TRUE | 0.121246 | TRUE |
| 3,5－Dimethoxy－4－glucosyloxy－phenyl－propenyl alcohol | Ren shen | 0.906419 | TRUE | 0.164729 | TRUE |
| 7,10-octadecadienoie acid,methyl ester | Ren shen | 0.268209 | TRUE | 0.106376 | TRUE |
| 7alpha-L-Rhamnosyl-6-methoxylutcolin | Ren shen | 1 | TRUE | 0.3062 | TRUE |
| alexandrin | Ren shen | 0.97605 | TRUE | 0.441895 | TRUE |
| alexandrin_qt | Ren shen | 0.88894 | TRUE | 0.886617 | TRUE |
| Campesteryl ferulate | Ren shen | 0.998374 | TRUE | 0.946343 | TRUE |
| Chrysanthemaxanthin | Ren shen | 0.229259 | TRUE | 0.124157 | TRUE |
| darutoside | Ren shen | 0.934906 | TRUE | 0.49037 | TRUE |
| Deoxygomisin A | Ren shen | 0.890687 | TRUE | 1 | TRUE |
| Deoxyharringtonine | Ren shen | 0.569115 | TRUE | 0.591242 | TRUE |
| Elemicin | Ren shen | 0.22451 | TRUE | 0.111609 | TRUE |
| Fumarine | Ren shen | 0.86306 | TRUE | 0.533915 | TRUE |
| Gomisin A | Ren shen | 0.891492 | TRUE | 1 | TRUE |
| Gomisin B | Ren shen | 0.489314 | TRUE | 0.999989 | TRUE |
| Inermin | Ren shen | 0.644108 | TRUE | 0.293958 | TRUE |
| Kaempferol-3-arabofuranoside | Ren shen | 1 | TRUE | 0.195684 | TRUE |
| methyl (Z)-icos-11-enoate | Ren shen | 0.228078 | TRUE | 0.112914 | TRUE |
| Methyl palmitelaidate | Ren shen | 0.197937 | TRUE | 0.108895 | TRUE |
| Methyl stearate | Ren shen | 0.153987 | TRUE | 0.125454 | TRUE |
| Methyl tricosanoate | Ren shen | 0.155728 | TRUE | 0.125238 | TRUE |
| Nepetin | Ren shen | 0.999997 | TRUE | 0.316387 | TRUE |
| Palmifin | Ren shen | 0.202978 | TRUE | 0.201647 | TRUE |
| Pancratistatin | Ren shen | 0.457336 | TRUE | 0.894244 | TRUE |
| Psuedohypericin | Ren shen | 0.430031 | TRUE | 0.170382 | TRUE |
| Stigmasterol | Ren shen | 0.759948 | TRUE | 0.905391 | TRUE |
| suchilactone | Ren shen | 0.908074 | TRUE | 0.162542 | TRUE |
| Trifolin | Ren shen | 1 | TRUE | 0.201149 | TRUE |
| Trifolirhizin | Ren shen | 0.992315 | TRUE | 0.329651 | TRUE |
| γ-sitosterol | Ren shen | 0.852963 | TRUE | 0.886617 | TRUE |
| Chalconaringenin | Shao yao | 0.40311 | TRUE | 0.11515 | TRUE |
| cis-ε-viniferin | Shao yao | 0.450677 | TRUE | 0.214505 | TRUE |
| dihydroapigenin | Shao yao | 0.542831 | TRUE | 0.162267 | TRUE |
| gnetin H | Shao yao | 0.277049 | TRUE | 0.195085 | TRUE |
| trans-ε-viniferin | Shao yao | 0.435359 | TRUE | 0.214505 | TRUE |
| β-Sitosterol | Shao yao | 0.893522 | TRUE | 0.886617 | TRUE |
| [6]-Gingerol monoacetate | Sheng jiang | 0.628504 | TRUE | 0.107754 | TRUE |
| 1-（4-Hydroxy-3-methoxyphenyl）-3,5-diacetoxyoctane | Sheng jiang | 0.646824 | TRUE | 0.108848 | TRUE |
| 1-Dehydro-[8]-gingerdione | Sheng jiang | 0.82813 | TRUE | 0.112434 | TRUE |
| 3，5－Diacetoxy-1-(4-hydroxy-3,5-dimethoxyphenyl)-7-(4-hydroxy-3-methoxyphenyl)heptane | Sheng jiang | 0.787729 | TRUE | 0.157881 | TRUE |
| 6-Gingediacetate | Sheng jiang | 0.678535 | TRUE | 0.11423 | TRUE |
| Dehydro(O)-paradol | Sheng jiang | 0.589798 | TRUE | 0.101002 | TRUE |
| lauric acid | Sheng jiang | 0.88894 | TRUE | 0.886617 | TRUE |
| Meso-3,5-diacetoxy-1,7-bis-(4-hydroxy-3-methoxyphenyl)heptanes | Sheng jiang | 0.674703 | TRUE | 0.112118 | TRUE |
| α-Copaene | Sheng jiang | 0.163011 | TRUE | 0.262619 | TRUE |
| 2-Hydroxy-hexadecanoic acid | Xing ren | 0.160872 | TRUE | 0.101187 | TRUE |
| Aromadendrin | Xing ren | 0.594549 | TRUE | 0.916251 | TRUE |
| CLR | Xing ren | 0.927732 | TRUE | 0.901984 | TRUE |
| Dihydroquercetin | Xing ren | 0.832164 | TRUE | 0.964602 | TRUE |
| ent-Epiafzelechin-(2α→O→7, 4α→8)-(-)-afzelechin | Xing ren | 0.506443 | TRUE | 0.396144 | TRUE |
| ent-Epiafzelechin-(2α→O→7, 4α→8)-(+)-afzelechin | Xing ren | 0.506443 | TRUE | 0.396144 | TRUE |
| ent-Epiafzelechin-(2α→O→7, 4α→8)-epiafzelechin | Xing ren | 0.506443 | TRUE | 0.396144 | TRUE |
| Flavoxanthin | Xing ren | 0.194535 | TRUE | 0.116237 | TRUE |
| Heriguard | Xing ren | 0.530054 | TRUE | 0.133243 | TRUE |
| Olein | Xing ren | 0.309667 | TRUE | 0.152173 | TRUE |
| Rubixanthin | Xing ren | 0.299503 | TRUE | 0.131246 | TRUE |
| terephthaldehyde | Xing ren | 0.530054 | TRUE | 0.133243 | TRUE |

**Table S8** Neuroprotective effects of compounds on H_2_O_2_ or Na_2_S_2_O_4_-induced neurotoxicity on SH-SY5Y cells

| Compound | H_2_O_2_ (200μM) | | | |
| --- | --- | --- | --- | --- |
|  | 0.3 | 1 | 3 | 10 |
| 5-O-methylvisammioside | 75.40±1.61 | 81.42±0.96 ^b^ | 83.68±6.14 ^b^ | 85.46±5.18 ^b^ |
| Chrysin | 72.33±10.19 | 76.52±11.93 | 82.24±6.75 ^a^ | 79.59±4.18 |
| Tetrandrine | 66.90±9.35 | 63.61±9.74 | 57.68±10.27 | 54.91±4.60 |
| Fangchinoline | 71.18±10.34 | 72.49±9.66 | 61.78±13.56 | 3.37±3.05 ^b^ |
| Baicalein | 78.06±4.14 ^b^ | 79.95±5.91 ^b^ | 84.89±7.92 ^b^ | 87.76±8.33 ^b^ |
| Baicalin | 84.84±3.88 ^b^ | 87.58±7.12 ^b^ | 86.10±5.23 ^b^ | 80.98±1.93 ^a^ |
| Wogonin | 64.56±3.20 | 69.81±6.71 | 64.31±3.73 | 62.56±1.46 |
| Wogonoside | 62.95±4.61 | 66.01±1.52 | 65.24±2.19 | 67.96±3.38 |
| Cimifugin | 71.29±7.35 ^a^ | 73.02±6.56 ^b^ | 80.35±12.22 ^b^ | 88.75±7.08 ^b^ |
| Prim-o-glucosylcimifugin | 72.32±7.59 ^a^ | 73.20±8.47 ^a^ | 83.08±6.49 ^b^ | 88.90±6.29 ^b^ |
| Compound | Na_2_S_2_O_4_ (8μM) | | | |
|  | 0.3 | 1 | 3 | 10 |
| 5-O-methylvisammioside | 71.13±1.95 ^c^ | 71.77±1.56 ^c^ | 72.33±2.42 ^c^ | 73.98±2.64 ^d^ |
| Chrysin | 69.28±2.78 | 72.35±2.41 ^c^ | 72.85±1.70 ^c^ | 74.79±3.08 ^c^ |
| Tetrandrine | 67.06±2.40 | 71.63±3.78 ^c^ | 68.60±4.09 | 66.86±2.69 |
| Fangchinoline | 67.96±1.58 | 67.05±1.26 | 69.70±1.76 | 71.16±2.93 |
| Baicalein | 78.13±2.97 ^d^ | 82.42±2.64 ^d^ | 83.32±2.48 ^d^ | 81.99±1.83 ^d^ |
| Baicalin | 79.21±1.80 d | 81.34±2.25 ^d^ | 82.11±2.01 ^d^ | 82.55±0.90 ^d^ |
| Wogonin | 67.74±0.57 | 70.98±2.65 | 69.17±1.88 | 65.17±3.56 |
| Wogonoside | 69.23±1.32 | 70.16±1.96 | 68.58±2.13 | 69.71±2.85 |
| Cimifugin | 66.30±4.31 | 64.68±2.42 | 71.82±2.35 | 58.96±1.55 |
| Prim-o-glucosylcimifugin | 65.52±5.98 | 67.04±10.34 | 72.68±2.24 ^c^ | 75.16±0.55 ^d^ |

The data (cell viability, measured by MTT assay) were normalized and expressed as a percentage of the control group, which was set to 100%. Degree of damage of H_2_O_2_ was 67.22±4.26 and degree of damage of Na_2_S_2_O_4_ was 68.78±1.09. Data expressed as means ±SEM. Three independent experiments were carried out. ^a^ P < 0.05. ^b^ P < 0.01 vs. H_2_O_2_ group. ^c^ P < 0.05. ^d^ P <0.01 vs. Na_2_S_2_O_4_ group.
